# Supplementary material for: Item-level analyses reveal genetic heterogeneity in neuroticism
Source: Nat Commun. 2018 Mar 2;9:905. doi: 10.1038/s41467-018-03242-8 (PMC5834468; doi:10.1038/s41467-018-03242-8)
Supplement: Supplementary file 1 — Description of Supplementary Data Files [file 41467_2018_3242_MOESM1_ESM.pdf]

Description of Supplementary Data Files

File Name: Supplementary Data 1
Description: Genetic correlations (r_g) between 15 Neuroticism phenotypes (12 items, sum-score, 2 clusters), calculated using LD score regression. Genetic correlations were computed separately in samples 1 and 2.

File Name: Supplementary Data 2
Description: Association *P* values for all genome-wide significant lead SNPs for the 12 individual items and the sum-score.

File Name: Supplementary Data 3
Description: Description of excluded loci. Position, association *P* values per sample, minor allele frequency in UKB, 1000 genomes and TOPMED data for 6 SNPs that were excluded from analyses.

File Name: Supplementary Data 4
Description: SNPs that are in LD (r^2>0.6) with lead SNPs in the excluded loci. For the loci listed in Supplementary Data 3 we checked whether there are SNPs in the 1000 genomes (1000G) data that are in high LD (r^2>06) with the lead SNPs. Subsequently, we determined the r^2 for these combinations of SNPs in the UKB data. If r^2 is very different depending on the dataset this might indicate genotyping errors.

File Name: Supplementary Data 5
Description: Summary statistics of all SNPs that were genome-wide significant in the meta-analysis of item IRR (UKB field ID: 1940). See Supplementary Table 1 for an explanation of item labels.

File Name: Supplementary Data 6
Description: Summary statistics of all SNPs that were genome-wide significant in the meta-analysis of item LONE (UKB field ID: 2020). See Supplementary Table 1 for an explanation of item labels.

File Name: Supplementary Data 7
Description: Summary statistics of all SNPs that were genome-wide significant in the meta-analysis of item MIS (UKB field ID: 1930). See Supplementary Table 1 for an explanation of item labels.

File Name: Supplementary Data 8
Description: Summary statistics of all SNPs that were genome-wide significant in the meta-analysis of item MOOD (UKB field ID: 1920). See Supplementary Table 1 for an explanation of item labels.

File Name: Supplementary Data 9
Description: Summary statistics of all SNPs that were genome-wide significant in the meta-analysis of item FED-UP (UKB field ID: 1960). See Supplementary Table 1 for an explanation of item labels.

File Name: Supplementary Data 10
Description: Summary statistics of all SNPs that were genome-wide significant in the meta-analysis of item NERV-FEEL (UKB field ID: 1970). See Supplementary Table 1 for an explanation of item labels.

File Name: Supplementary Data 11
Description: Summary statistics of all SNPs that were genome-wide significant in the meta-analysis of item WORRY (UKB field ID: 1980). See Supplementary Table 1 for an explanation of item labels.

File Name: Supplementary Data 12
Description: Summary statistics of all SNPs that were genome-wide significant in the meta-analysis of item TENSE (UKB field ID: 1990). See Supplementary Table 1 for an explanation of item labels.

File Name: Supplementary Data 13
Description: Summary statistics of all SNPs that were genome-wide significant in the meta-analysis of item SUF-NERV (UKB field ID: 2010). See Supplementary Table 1 for an explanation of item labels.

File Name: Supplementary Data 14
Description: Summary statistics of all SNPs that were genome-wide significant in the meta-analysis of item HURT (UKB field ID: 1950). See Supplementary Table 1 for an explanation of item labels.

File Name: Supplementary Data 15
Description: Summary statistics of all SNPs that were genome-wide significant in the meta-analysis of item WORR-EMB (UKB field ID: 2000). See Supplementary Table 1 for an explanation of item labels.

File Name: Supplementary Data 16
Description: Summary statistics of all SNPs that were genome-wide significant in the meta-analysis of item GUILT (UKB field ID: 2030). See Supplementary Table 1 for an explanation of item labels.

File Name: Supplementary Data 17
Description: Summary statistics of all SNPs that were genome-wide significant in the meta-analysis of the neuroticism sum-score.

File Name: Supplementary Data 18
Description: Genes implicated in SNP-based analysis (FUMA) of item IRR. See Supplementary Table 1 for an explanation of item labels.

File Name: Supplementary Data 19
Description: Genes implicated in SNP-based analysis (FUMA) of item LONE. See Supplementary Table 1 for an explanation of item labels.

File Name: Supplementary Data 20
Description: Genes implicated in SNP-based analysis (FUMA) of item MIS. See Supplementary Table 1 for an explanation of item labels.

File Name: Supplementary Data 21
Description: Genes implicated in SNP-based analysis (FUMA) of item MOOD. See Supplementary Table 1 for an explanation of item labels.

File Name: Supplementary Data 22
Description: Genes implicated in SNP-based analysis (FUMA) of item FED-UP. See Supplementary Table 1 for an explanation of item labels.

File Name: Supplementary Data 23
Description: Genes implicated in SNP-based analysis (FUMA) of item NERV-FEEL. See Supplementary Table 1 for an explanation of item labels.

File Name: Supplementary Data 24
Description: Genes implicated in SNP-based analysis (FUMA) of item WORRY. See Supplementary Table 1 for an explanation of item labels.

File Name: Supplementary Data 25
Description: Genes implicated in SNP-based analysis (FUMA) of item TENSE. See Supplementary Table 1 for an explanation of item labels.

File Name: Supplementary Data 26
Description: Genes implicated in SNP-based analysis (FUMA) of item SUF-NERV. See Supplementary Table 1 for an explanation of item labels.

File Name: Supplementary Data 27
Description: Genes implicated in SNP-based analysis (FUMA) of item HURT. See Supplementary Table 1 for an explanation of item labels.

File Name: Supplementary Data 28
Description: Genes implicated in SNP-based analysis (FUMA) of item WORR-EMB. See Supplementary Table 1 for an explanation of item labels.

File Name: Supplementary Data 29
Description: Genes implicated in SNP-based analysis (FUMA) of item GUILT. See Supplementary Table 1 for an explanation of item labels.

File Name: Supplementary Data 30
Description: Genes implicated in SNP-based analysis (FUMA) of the neuroticism sum-score.

File Name: Supplementary Data 31
Description: Results of the sign-concordance tests (for different *P* value thresholds). See the Methods and Supplementary Note 1 for detailed information on the sign-concordance tests.

File Name: Supplementary Data 32
Description: Results of the Fisher's exact tests (for different *P* value thresholds). See the Methods and Supplementary Note 1 for detailed information on the Fisher’s exact tests.

File Name: Supplementary Data 33
Description: Association *P* values for all genome-wide significant genes identified in gene-based analyses (MAGMA) for the 12 individual items and the sum-score.

File Name: Supplementary Data 34
Description: Genes implicated by gene-based analyses in MAGMA for item IRR. See Supplementary Table 1 for an explanation of item labels.

File Name: Supplementary Data 35
Description: Genes implicated by gene-based analyses in MAGMA for item LONE. See Supplementary Table 1 for an explanation of item labels.

File Name: Supplementary Data 36
Description: Genes implicated by gene-based analyses in MAGMA for item MIS. See Supplementary Table 1 for an explanation of item labels.

File Name: Supplementary Data 37
Description: Genes implicated by gene-based analyses in MAGMA for item MOOD. See Supplementary Table 1 for an explanation of item labels.

File Name: Supplementary Data 38
Description: Genes implicated by gene-based analyses in MAGMA for item FED-UP. See Supplementary Table 1 for an explanation of item labels.

File Name: Supplementary Data 39
Description: Genes implicated by gene-based analyses in MAGMA for item NERV-FEEL. See Supplementary Table 1 for an explanation of item labels.

File Name: Supplementary Data 40
Description: Genes implicated by gene-based analyses in MAGMA for item WORRY. See Supplementary Table 1 for an explanation of item labels.

File Name: Supplementary Data 41
Description: Genes implicated by gene-based analyses in MAGMA for item TENSE. See Supplementary Table 1 for an explanation of item labels.

File Name: Supplementary Data 42
Description: Genes implicated by gene-based analyses in MAGMA for item SUF-NERV. See Supplementary Table 1 for an explanation of item labels.

File Name: Supplementary Data 43

Description: Genes implicated by gene-based analyses in MAGMA for item HURT. See Supplementary Table 1 for an explanation of item labels.

File Name: Supplementary Data 44
Description: Genes implicated by gene-based analyses in MAGMA for item WORR-EMB. See Supplementary Table 1 for an explanation of item labels.

File Name: Supplementary Data 45
Description: Genes implicated by gene-based analyses in MAGMA for item GUILT. See Supplementary Table 1 for an explanation of item labels.

File Name: Supplementary Data 46
Description: Genes implicated by gene-based analyses in MAGMA for the neuroticism sum-score.

File Name: Supplementary Data 47
Description: Association *P* values of specific gene-sets that were significantly associated to at least one of the individual items or the neuroticism sum-score. Gene-sets were obtained from MsigDB (canonical pathways and gene ontology gene-sets) and from the GTEx portal (v6). In the gene-set analysis of the 53 tissue types of the GTEx data we conditioned on average expression of the gene within each tissue.

File Name: Supplementary Data 48
Description: Exonic non-synonymous (ExNS) variants in the genomic loci associated with individual neuroticism items or the sum-score and in LD (r^2>0.6) with one of the independent GWS SNPs. ExNS SNPs that occur more than once (typically indicating the SNP is identified in both the analysis of individual items as well as analysis of the sum-score) are marked green. Genes that occur more than once are marked yellow.

File Name: Supplementary Data 49
Description: Overview of traits tested for genetic correlation to 15 neuroticism phenotypes (12 items, sum-score, clusters *Depressed Affect* and *Worry*).

File Name: Supplementary Data 50
Description: Genetic correlations between the 12 neuroticism items, the neuroticism sum-score, the two item clusters and 33 other traits. For computing the genetic correlations, we used the summary statistics of the meta-analyses of the 15 Neuroticism phenotypes. Genetic correlations were calculated using LD score regression. None of the 95% confidence intervals ($CI_{95\%)}$ includes 1, indicating that all genetic correlations deviate from unity.
