# Supplementary material for: Item-level analyses reveal genetic heterogeneity in neuroticism
Source: Nat Commun. 2018 Mar 2;9:905. doi: 10.1038/s41467-018-03242-8 (PMC5834468; doi:10.1038/s41467-018-03242-8)
Supplement: Supplementary file 2 — Supplementary Information [file 41467_2018_3242_MOESM2_ESM.pdf]

Supplementary Information for

Item-level Analyses Reveal Genetic Heterogeneity in Neuroticism

Nagel et al.

Supplementary Note 1. Additional information on genetic overlap tests for top associated SNPs

Before conducting the sign concordance tests to establish whether signs of top associated SNPs were in the same direction across the 13 phenotypes, we used clumping (r^2 = 0.1 with window size 500 kb) based on the association *P* values to ensure that the most strongly associated variants were not lost. As clumping results in different SNP sets for different phenotypes, sign tests results are not symmetrical for pairs of phenotypes. To explicate the followed procedure:

1) Select two items I1 & I2
2) Retain only SNPs that survived clumping for item I1
3) Filter out all variants with P > threshold for phenotype I1
4) Of the selected SNPs, compute the number of SNPs that have the same sign (i.e., concordant) for both I1 and I2
5) The proportion we report is: $prop = \frac{N_{concordant}}{N_{total}}$ (both numbers in this equation only contain SNPs below the specified *P* threshold for item I1, mentioned in step 3)
6) Repeat for all combinations of items and sum-score

In addition, we repeat the procedure with the difference that clumping and *P* value filtering is based on item I2 instead of item I1. This results in heatmaps (visualizing the proportion of concordant sign SNPs) that are not symmetric, which can be seen from the number of SNPs reported in each cell.

Prior to performing Fisher's exact tests to examine the overlap in the top associated SNPs across the 13 neuroticism phenotypes, we applied LD-based pruning on the summary statistics from all items to ensure that SNPs were independent (a subset of 10,000 individuals from the UK biobank, the UK10K set, was used as a reference set; r^2 = 0.8). Using the SNPs that survived pruning, we then created (for each combination of items, and for each *P* value

threshold) a table like the one depicted below. The categories in the table below are exclusive, meaning that a SNP in cell **A** is not included in the count of cells **B** and **C**.

**A**<br>Number of SNPs < $P_{threshold}$ for both items	**B**<br>Number of SNPs < $P_{threshold}$ for item 2 & > $P_{threshold}$ for item 1
**C**<br>Number of SNPs < $P_{threshold}$ for item 1 & > $P_{threshold}$ for item 2	**D**<br>Number of SNPs > $P_{threshold}$ for both items

Supplementary Note 2. Diagnostic criteria major depressive disorder

Below we list the symptoms used in the diagnosis of major depressive disorder, as described more extensively in the Diagnostic and Statistical Manual (DSM-5[8]).

1. Depressed mood
2. Diminished interest or pleasure
3. Weight loss or weight gain, or decrease or increase in appetite
4. Insomnia or hypersomnia
5. Psychomotor agitation or retardation
6. Fatigue or loss of energy
7. Feelings of worthlessness or excessive or inappropriate
8. Diminished ability to think or concentrate, or indecisiveness
9. Recurrent thoughts of death, recurrent suicidal ideation

Supplementary Table 1. Content, corresponding abbreviations, *N*, and endorsement rates of the 12 EPQ-RS[1] neuroticism items and the sum-score in sample 1

		Female		Male		Total	
**Abbreviation**	**Item (UKB field code)**	**N**	**% yes**	**N**	**% yes**	**N**	**% yes**
IRR	Are you an irritable person? (1940)	55,729	0.27	50,628	0.31	106,357	0.29
	"Feeling irritable" (IDS)						
LONE	Do you often feel lonely? (2020)	57,295	0.21	51,867	0.15	109,162	0.18
	"I feel lonely" (YASR)						
MIS	Do you ever feel 'just miserable' for no reason? (1930)	57,301	0.49	51,746	0.36	109,047	0.43
	Depressed mood (HDRS)						
MOOD	Does your mood often go up and down? (1920)	56,935	0.48	51,416	0.43	108,351	0.45
FED-UP	Do you often feel 'fed-up'? (1960)	57,086	0.43	51,677	0.38	108,763	0.41
NERV-FEEL	Would you call yourself a nervous person? (1970)	56,639	0.27	51,624	0.19	108,263	0.23
	"I am nervous or tense" (YASR)						
WORRY	Are you a worrier? (1980)	56,852	0.64	51,371	0.47	108,223	0.56
	"I worry a lot" (YASR)						
TENSE	Would you call yourself tense or 'highly strung'? (1990)	56,398	0.20	51,285	0.15	107,683	0.18
	Feeling anxious or tense (IDS)						
SUF-NERV	Do you suffer from 'nerves'? (2010)	56,057	0.21	51,121	0.21	107,178	0.21
HURT	Are your feelings easily hurt? (1950)	56,713	0.64	51,190	0.45	107,903	0.55
	Interpersonal sensitivity (IDS)						
WORR-EMB	Do you worry too long after an embarrassing experience? (2000)	55,793	0.54	50,838	0.40	106,631	0.47
GUILT	Are you often troubled by feelings of guilt? (2030)	56,764	0.34	51,477	0.22	108,241	0.28
	"I feel too guilty" (YASR);						
	Feelings of guilt/worthlessness (BDI);						
	Feelings of guilt (HDRS)						

		Female		Male		Total	
	**Phenotype**	**N**	**M (SD)**	**N**	**M (SD)**	**N**	**M (SD)**
SUM-SCORE	Neuroticism sum-score	57,958	4.62 (3.20)	52,370	3.64 (3.16)	110,328	4.16 (3.22)

Descriptive statistics for the 12 neuroticism items and the sum-score in sample 1 (UKB data: first release). Below the neuroticism items (black font), highly similar items (content and source; grey font) from renowned depression/anxiety questionnaires are displayed. The neuroticism sum-score ranges from 0 to 12.

BDI = Beck Depression Inventory[2]; IDS = Inventory of Depressive Symptomatology[3]; HRDS = Hamilton Depression Rating Scale[4]; YASR = Young Adult Self Report scale[5].

Supplementary Table 2. Content, corresponding abbreviations, *N*, and endorsement rates of the 12 EPQ-RS[1] neuroticism items and the sum-score in sample 2

		Female		Male		Total	
**Abbreviation**	**Item (UKB field code)**	**N**	**% yes**	**N**	**% yes**	**N**	**% yes**
IRR	Are you an irritable person? (1940)	141,978	0.26	118,391	0.31	260,369	0.28
LONE	Do you often feel lonely? (2020)	145,829	0.20	121,361	0.15	267,190	0.18
MIS	Do you ever feel 'just miserable' for no reason? (1930)	145,908	0.49	121,142	0.35	267,050	0.43
MOOD	Does your mood often go up and down? (1920)	145,029	0.47	120,353	0.42	265,382	0.45
FED-UP	Do you often feel 'fed-up'? (1960)	145,371	0.42	120,837	0.38	266,208	0.4
NERV-FEEL	Would you call yourself a nervous person? (1970)	144,117	0.27	120,741	0.20	264,858	0.24
WORRY	Are you a worrier? (1980)	144,680	0.64	119,966	0.48	264,646	0.57
TENSE	Would you call yourself tense or 'highly strung'? (1990)	143,631	0.20	120,004	0.15	263,635	0.18
SUF-NERV	Do you suffer from 'nerves'? (2010)	142,635	0.21	119,686	0.22	262,321	0.21
HURT	Are your feelings easily hurt? (1950)	144,356	0.64	119,788	0.45	264,144	0.56
WORR-EMB	Do you worry too long after an embarrassing experience? (2000)	142,172	0.54	118,922	0.40	261,094	0.48
GUILT	Are you often troubled by feelings of guilt? (2030)	144,580	0.34	120,559	0.22	265,139	0.29
		**Female**		**Male**		**Total**	
	**Phenotype**	**N**	**M (SD)**	**N**	**M (SD)**	**N**	**M (SD)**
SUM-SCORE	Neuroticism sum-score	147,598	4.58 (3.18)	122,580	3.65 (3.16)	270,178	4.16 (3.21)

Descriptive statistics for the 12 neuroticism items and the sum-score in sample 2 (UKB data: first release). The neuroticism sum-score ranges from 0 to 12.

Supplementary Table 3. Overview of binary external variables used in phenotypic association analysis in sample 1

Item	*N*	Percentage yes	UKB field code
Binary variables			
Depressed mood ("Not at all" vs. other categories)	106,152	23.4%	2050
Unenthusiasm/disinterest ("Not at all" vs. other categories)	107,125	21.0%	2060
Tenseness/restlessness ("Not at all" vs. other categories)	106,825	26.5%	2070
Tiredness/lethargy ("Not at all" vs. other categories)	107,516	52.7%	2080
Visit psychiatrist for nerves/anxiety/tension/depression	109,965	11.8%	2100
Visit doctor (GP) for nerves/anxiety/tension/depression	109,729	34.5%	2090
Ever highly irritable/argumentative for 2 days	36,395	18.5%	4653
Ever depressed for a whole week	36,472	53.6%	4598
Ever manic/hyper for 2 days	36,255	6.0%	4642
Ever disinterested/unenthusiastic for a whole week	35,728	37.0%	4631
Bipolar disorder (both hypo- and hypermania)	110,328	0.3%	20122
Probable recurrent MDD (severe)	110,328	1.9%	20125
Probable recurrent MDD (moderate)	110,328	3.1%	20124
Insomnia/sleeplessness ("never/rarely"/"sometimes" vs. "usually")	110,255	28.5%	1200
Single episode of probable MDD	110,328	2.0%	20123
Risk taking	106,831	26.8%	2040
Sex (being male)	110,328	47.5%	31

Content, sample size (*N*) and endorsement rate (percentage of positive "yes" versus "no" or *NA* responses) for the binary external variables used in phenotypic association analysis in subsample 1 (UKB data: first release) as depicted in **Supplementary Figure 2**.

Supplementary Table 4. Overview of continuous external variables used in phenotypic association analysis in sample 1

**Item**	***N***	**M**	**SD**	**Range**	**UKB field code**
Continuous variables					
No. of depression episodes (duration at least one week)	14,269	3.7	4.4	1 - 20	4620
No. of disinterested/unenthusiastic episodes (duration at least one week)	9,431	4.1	4.8	1 - 20	5386
Longest period of depression	15,898	16.2	41.9	1 - 999	4609
Longest period of disinterest/unenthusiasm	10,151	16.0	44.9	1 - 999	5375
Townsend deprivation index (TDI)	110,183	-1.4	3.0	-6.3 - 10.6	189
Height	110,122	168.8	9.2	122 - 205	50
Age	110,328	56.7	8.0	39 - 70	21003
Financial satisfaction	36,944	4.3	1.0	1 - 6	4581
Friendships satisfaction	36,693	4.8	0.7	1 - 6	4570
Family satisfaction	36,707	4.8	0.9	1 - 6	4559
Health satisfaction	36,981	4.2	0.9	1 - 6	4548
Work satisfaction	25,554	4.4	0.9	1 - 6	4537
Happiness	36,970	4.5	0.7	1 - 6	4526

Content, sample size (*N*), mean (M) and standard deviation (SD) and item response range for the continuously measured external variables used in phenotypic association analyses in subsample 1 (UKB data: first release) as depicted in **Supplementary Figure 3**.

Supplementary Table 5. Content, corresponding abbreviations, *N*, and endorsement rates of the 12 EPQ-RS[1] neuroticism items and the sum-score in the full (meta-analyzed) sample

		Female		Male		Total	
**Abbreviation**	**Item (UKB field code)**	**N**	**% yes**	**N**	**% yes**	**N**	**% yes**
IRR	Are you an irritable person? (1940)	197,707	0.26	169,019	0.31	366,726	0.28
LONE	Do you often feel lonely? (2020)	203,124	0.21	173,228	0.15	376,352	0.18
MIS	Do you ever feel 'just miserable' for no reason? (1930)	203,209	0.49	172,888	0.35	376,097	0.43
MOOD	Does your mood often go up and down? (1920)	201,964	0.47	171,769	0.43	373,733	0.45
FED-UP	Do you often feel 'fed-up'? (1960)	202,457	0.43	172,514	0.38	374,971	0.4
NERV-FEEL	Would you call yourself a nervous person? (1970)	200,756	0.27	172,365	0.20	373,121	0.24
WORRY	Are you a worrier? (1980)	201,532	0.64	171,337	0.48	372,869	0.57
TENSE	Would you call yourself tense or 'highly strung'? (1990)	200,029	0.20	171,289	0.15	371,318	0.18
SUF-NERV	Do you suffer from 'nerves'? (2010)	198,692	0.21	170,807	0.22	369,499	0.21
HURT	Are your feelings easily hurt? (1950)	201,069	0.64	170,978	0.45	372,047	0.55
WORR-EMB	Do you worry too long after an embarrassing experience? (2000)	197,965	0.54	169,760	0.40	367,725	0.48
GUILT	Are you often troubled by feelings of guilt? (2030)	201,344	0.34	172,036	0.22	373,380	0.29
		**Female**		**Male**		**Total**	
	**Phenotype**	**N**	**M (SD)**	**N**	**M (SD)**	**N**	**M (SD)**
SUM-SCORE	Neuroticism sum-score	205,556	4.59 (3.19)	174,950	3.65 (3.16)	380,506	4.16 (3.21)

Descriptive statistics for the 12 neuroticism items and the sum-score in the full sample (UKB data: sample 1 and 2 combined). The neuroticism sum-score ranges from 0 to 12.

Supplementary Table 6. Functional consequences on genes of all SNPs in LD with an independent significant SNP in item- vs. sum-score analysis

	Item-level*	Sum-score
3'-UTR	322	175
5'-UTR	110	74
Exonic	286	151
Intergenic	9,693	5,141
Intronic	13,023	7,265
Non-coding RNA exonic	299	142
Non-coding RNA intronic	2,821	1,887
Non-coding RNA splicing	-	-
Splicing	1	-
Downstream	324	131
Upstream	313	109

Note: SNPs in the MHC region were excluded from annotation. A small portion of the SNPs could not be annotated by ANNOVAR.
*SNPs that were in LD with an independent significant SNP for at least one of the 12 neuroticism items.

Supplementary Table 7. Minimum chromatin state of all SNPs in LD with an independent significant SNP in item- vs. sum-score analysis

	**Item-level***	**Sum-score**
1: Active TSS	1,749	890
2: Flanking Active TSS	1,403	863
3: Transcr. at gene 5' & 3'	285	145
4: Strong transcription	5,230	3,080
5: Weak transcription	9,833	5,972
6: Genic enhancers	10	5
7: Enhancers	1,021	537
8: ZNF genes & repeats	787	201
9: Heterochromatin	5,085	2,596
12: Bivalent enhancer	8	3
13: Repressed PolyComb	368	157
14: Weak Repressed PolyComb	1,070	490
15: Quiescent/Low	364	221

Note: SNPs in the MHC region were excluded from annotation.
*SNPs that were in LD with an independent significant SNP for at least one of the 12 neuroticism items.

Supplementary Table 8. Regulome database score of all SNPs in LD with an independent significant SNP in item- vs. sum-score analysis

	**Item-level***	**Sum-score**
1a: eQTL + TF binding + matched TF motif + matched DNase Footpr	9	4
1b: eQTL + TF binding + any motif + DNase Footpr + DNase peak	71	35
1c: eQTL + TF binding + matched TF motif + DNase peak	4	4
1d: eQTL + TF binding + any motif + DNase peak	73	42
1e: eQTL + TF binding + matched TF motif	2	-
1f: eQTL + TF binding / DNase peak	866	534
2a: TF binding + match TF motif + matchDNase Footpr + DNase peak	43	24
2b: TF binding + any motif + DNase Footpr + DNase peak	385	214
2c: TF binding + matched TF motif + DNase peak	23	12
3a: TF binding + any motif + DNase peak	464	258
3b: TF binding + matched TF motif	11	6
4: TF binding + DNase peak	1,349	744
5: TF binding or DNase peak	4,532	2,829
6: other	7,355	3,990
7: not regulatory	8,520	4,589

Note: SNPs in the MHC region were excluded from annotation.
*SNPs that were in LD with an independent significant SNP for at least one of the 12 neuroticism items.

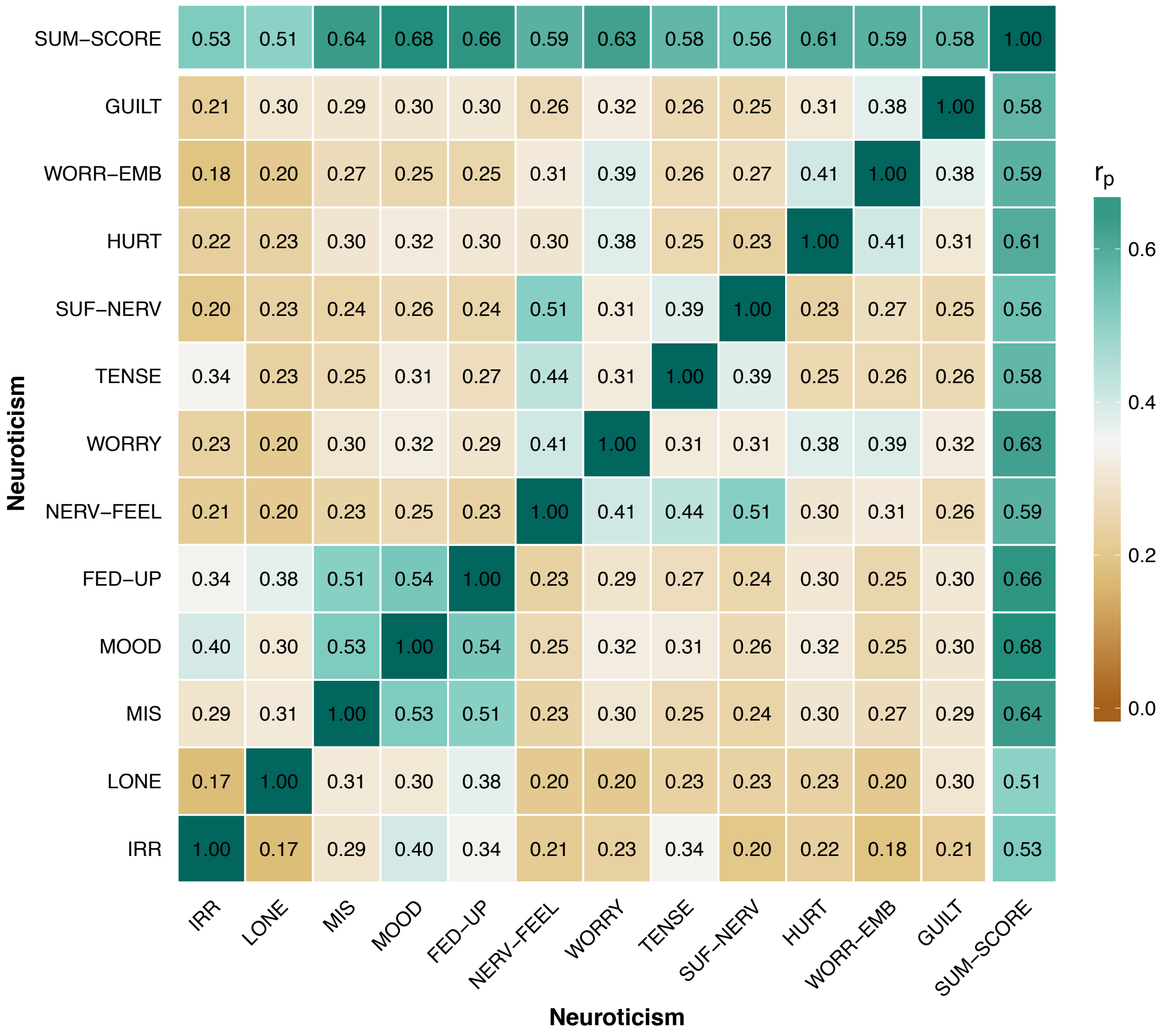

Supplementary Figure 1. Phenotypic correlations between all 12 neuroticism items and the neuroticism sum-score in sample 1.

The correlation heatmap is symmetric. See **Supplementary Table 1** for an explanation of item labels.

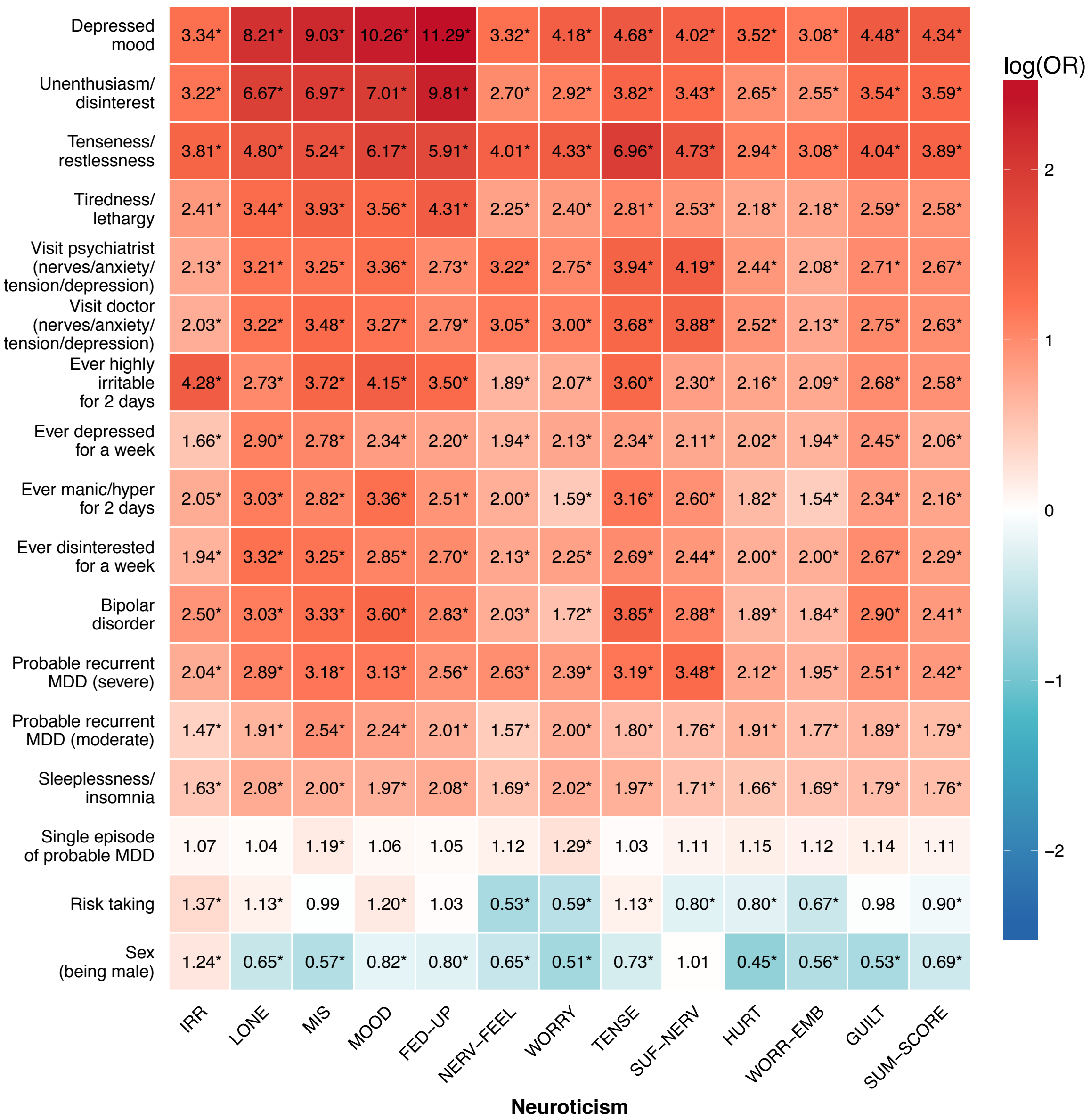

Supplementary Figure 2. Phenotypic associations of the 12 neuroticism items and the neuroticism sum-score (*x*-axis) with 17 dichotomous external variables (*y*-axis) in sample 1.

The numbers in the heatmap represent effect sizes expressed as odds ratios (coloring is based on log odds). See **Supplementary Table 1** for an explanation of neuroticism item labels. See **Supplementary Table 3** for descriptives on the external variables displayed in this figure.

*Bonferroni corrected *P* value threshold: $P < 1.28 \times 10^{-4}$ (0.05/((17+13) ×13).

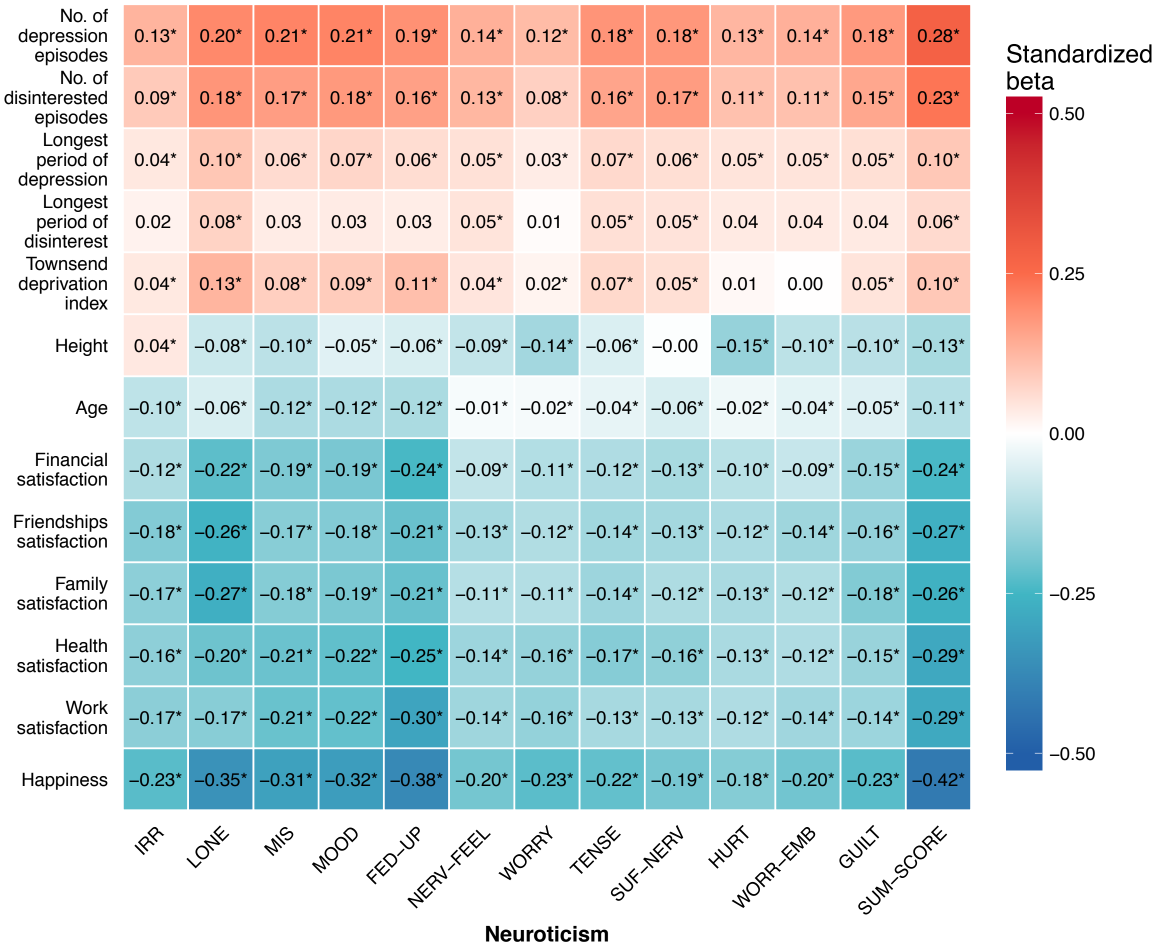

Supplementary Figure 3. Phenotypic associations of the 12 neuroticism items and the neuroticism sum-score (*x*-axis) with 13 continuous external variables (*y*-axis) in sample 1.

The numbers in the heatmap represent effect sizes expressed as standardized beta coefficients. See **Supplementary Table 1** for an explanation of neuroticism item labels. See **Supplementary Table 4** for descriptives on the external variables displayed in this figure.

*Bonferroni corrected *P* value threshold: $P < 1.28 \times 10^{-4}$ (0.05/((17+13) ×13).

IRR - Are you an irritable person?

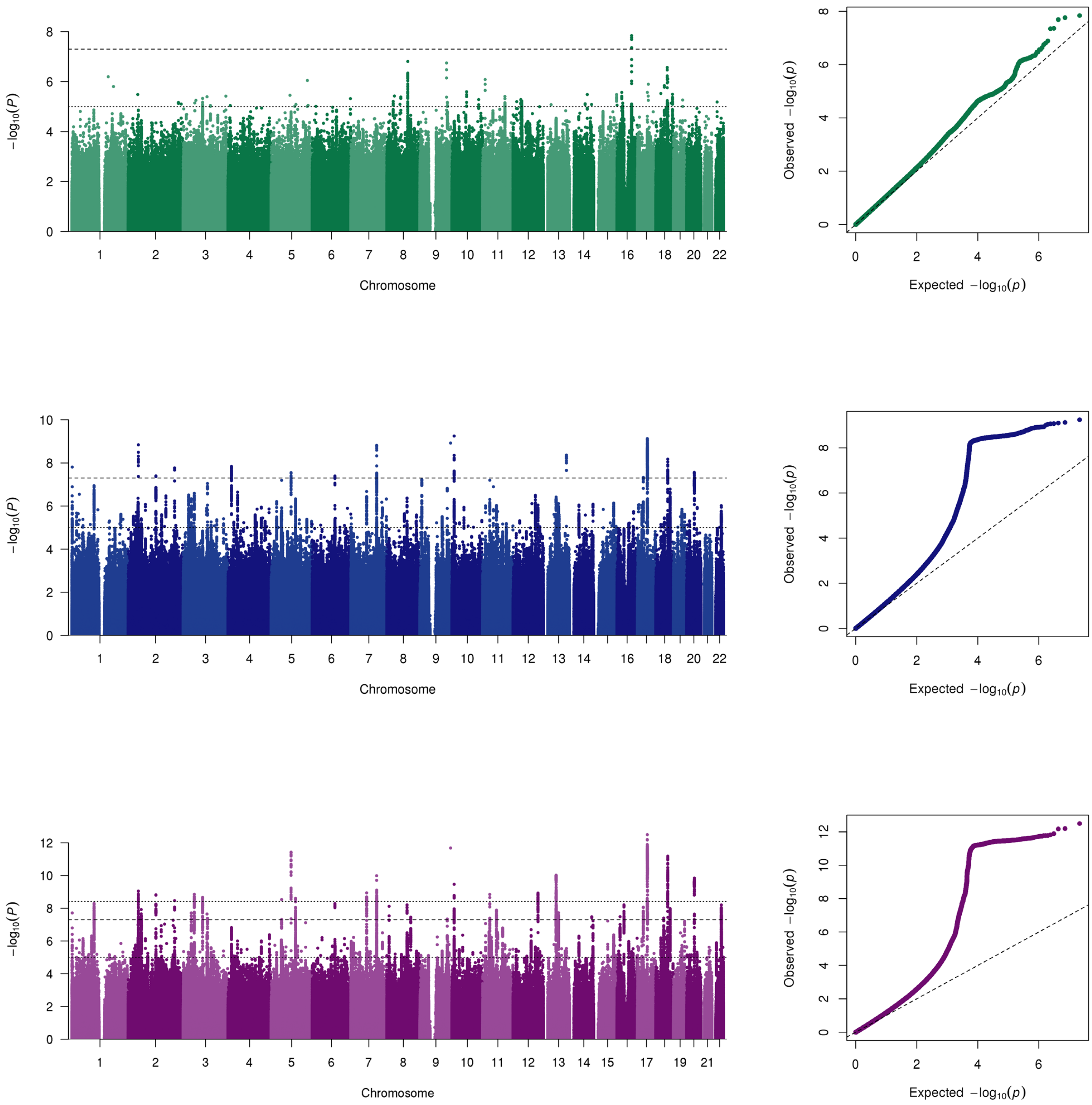

LONE - Do you often feel lonely?

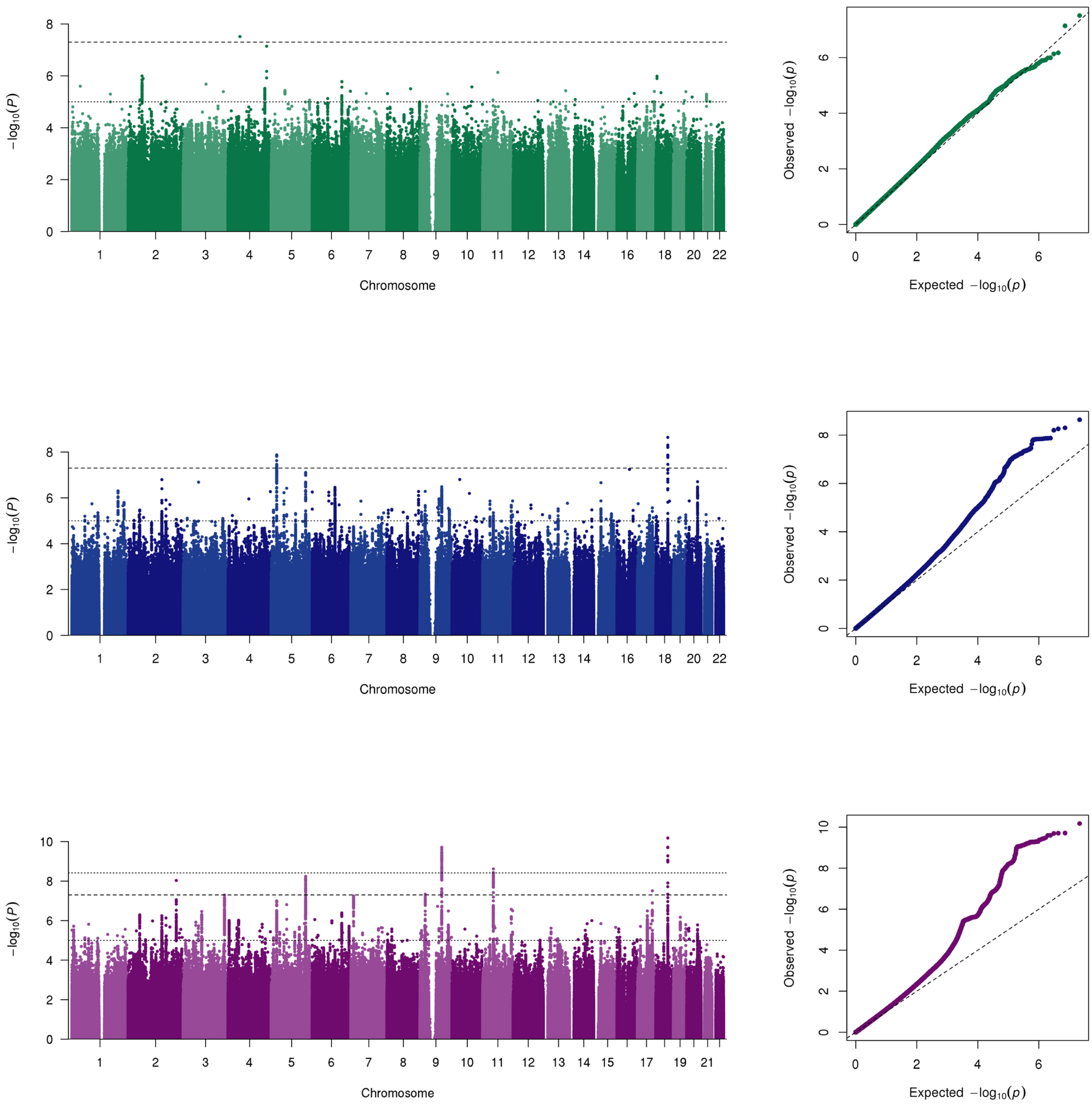

MIS - Do you ever feel ‘just miserable’ for no reason?

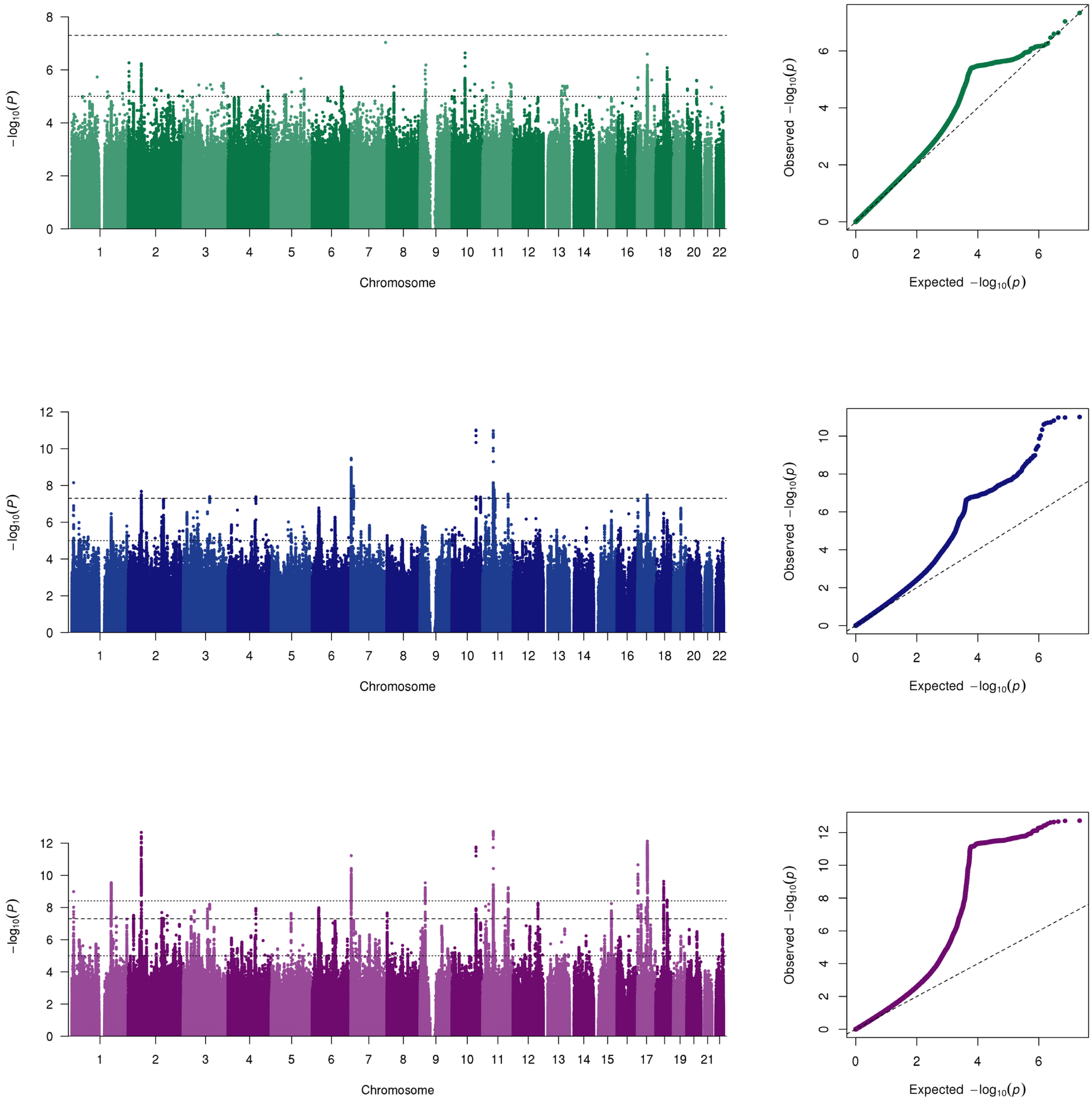

MOOD - Does your mood often go up and down?

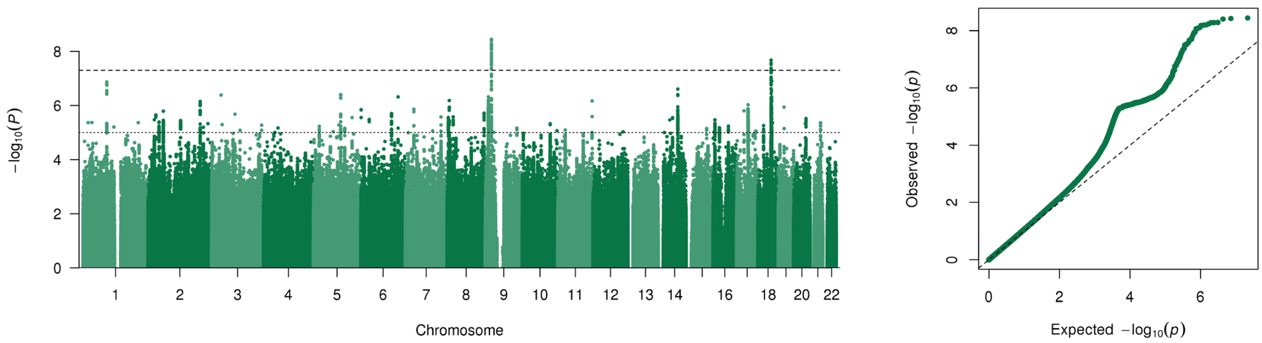

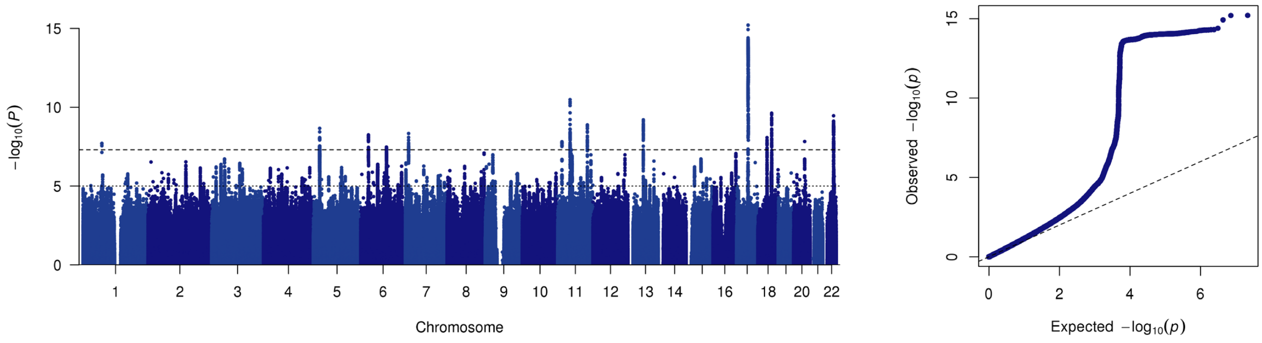

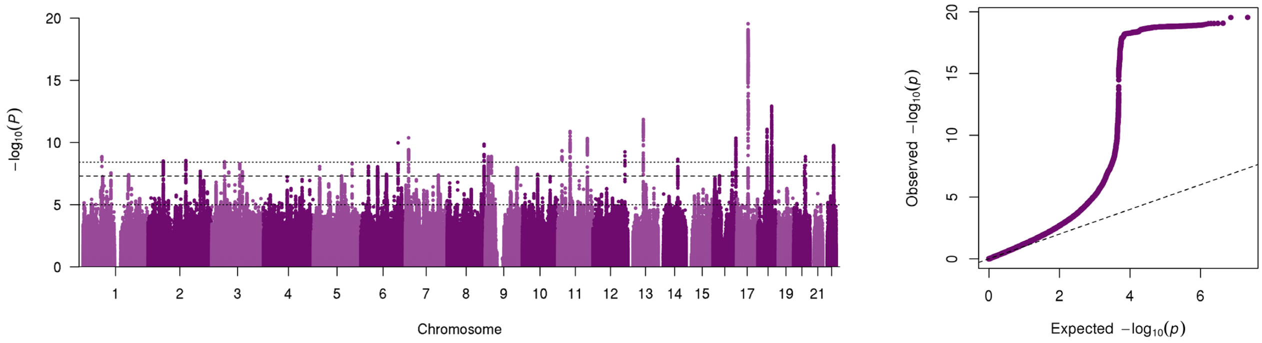

FED-UP - Do you often feel ‘fed-up’?

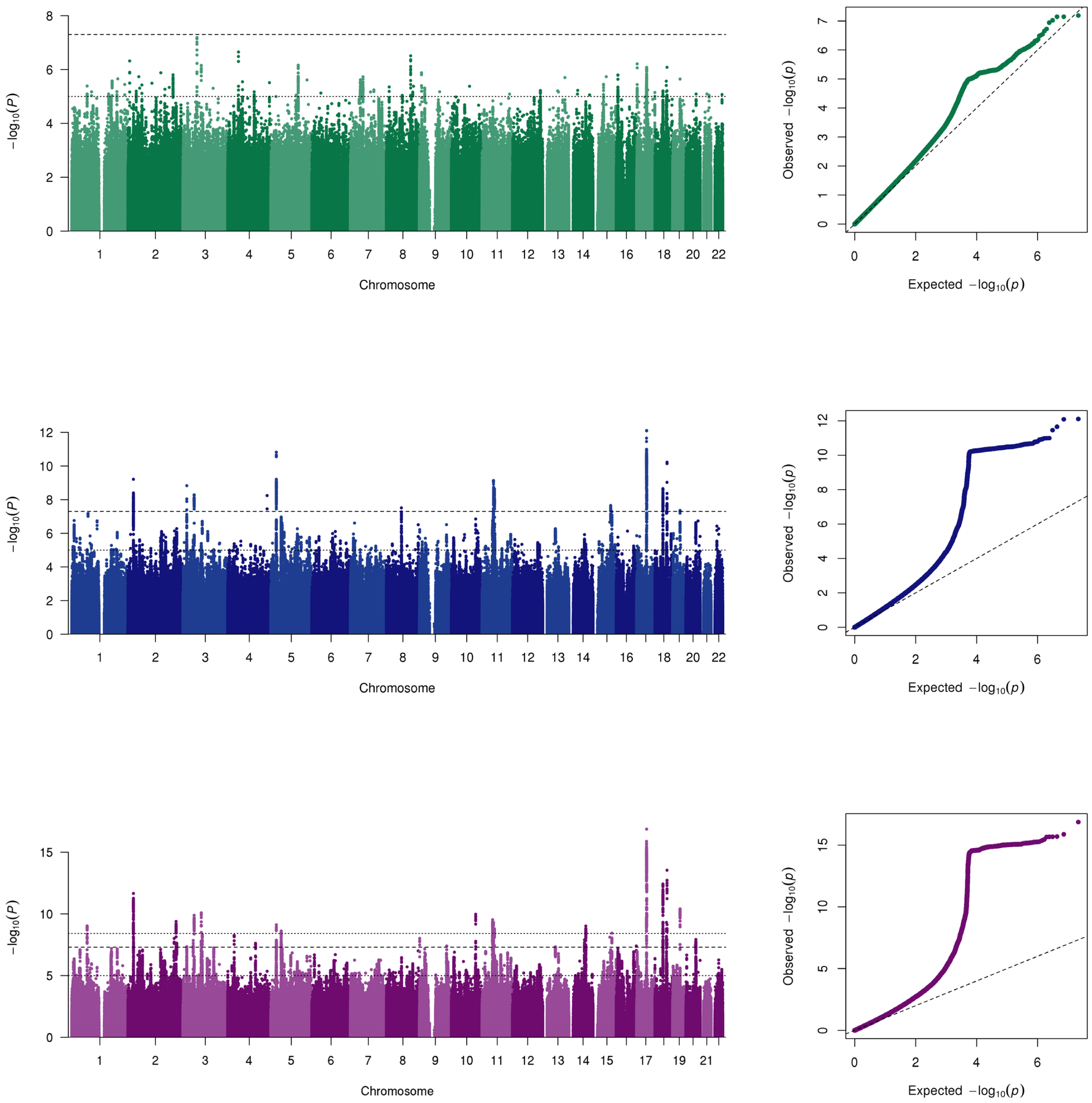

NERV-FEEL - Would you call yourself a nervous person?

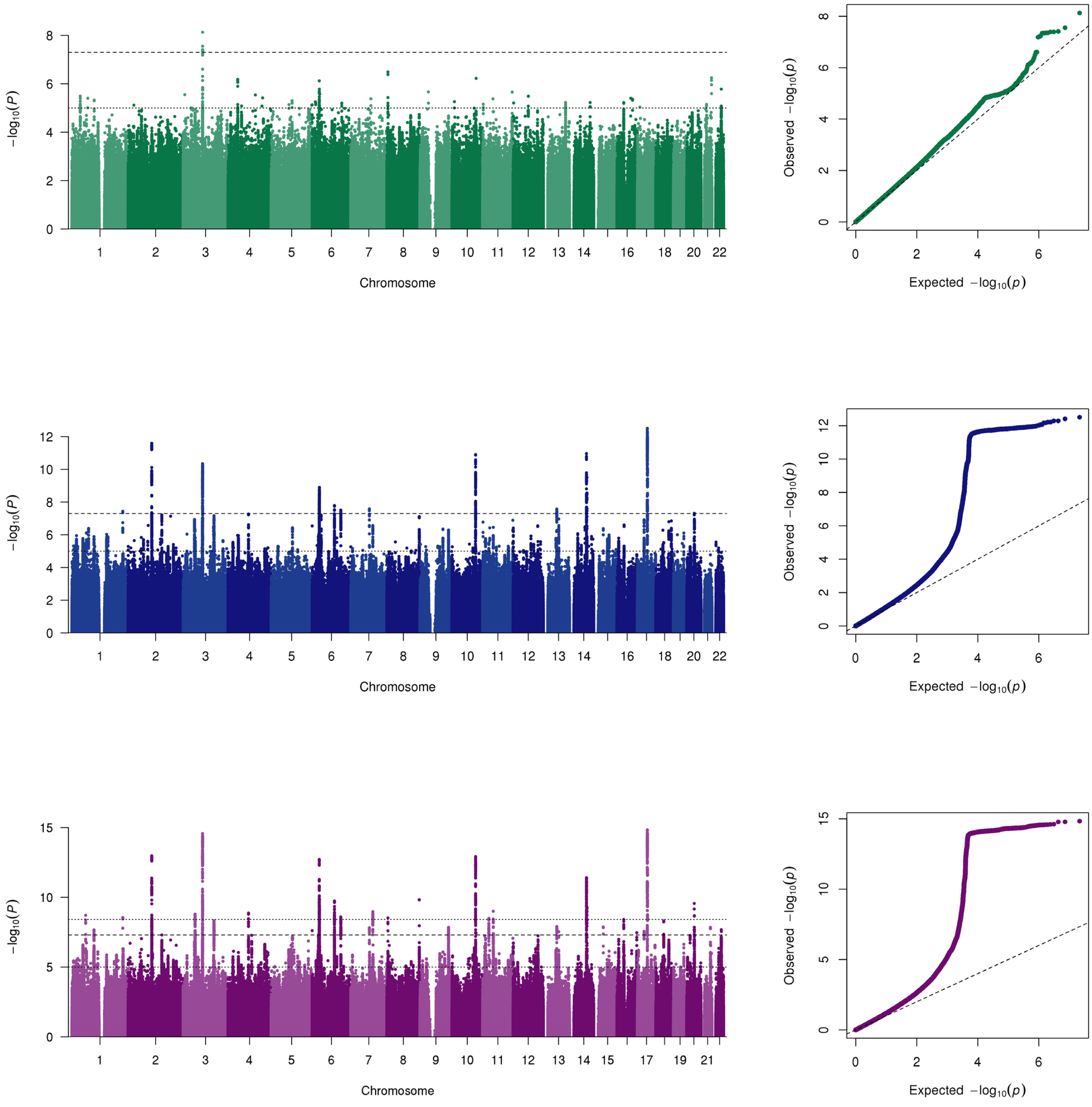

WORRY - Are you a worrier?

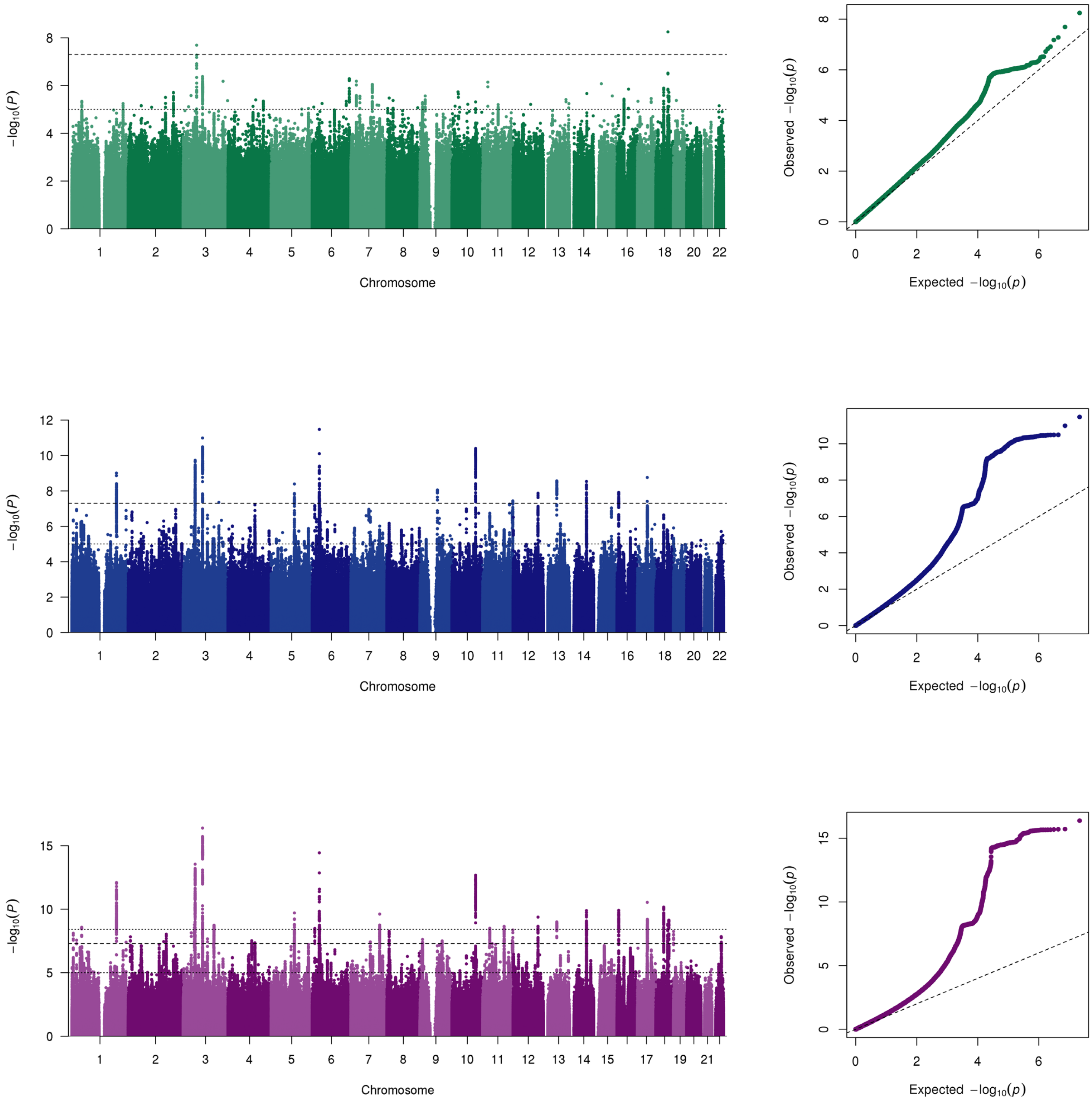

TENSE - Would you call yourself tense or ‘highly strung’?

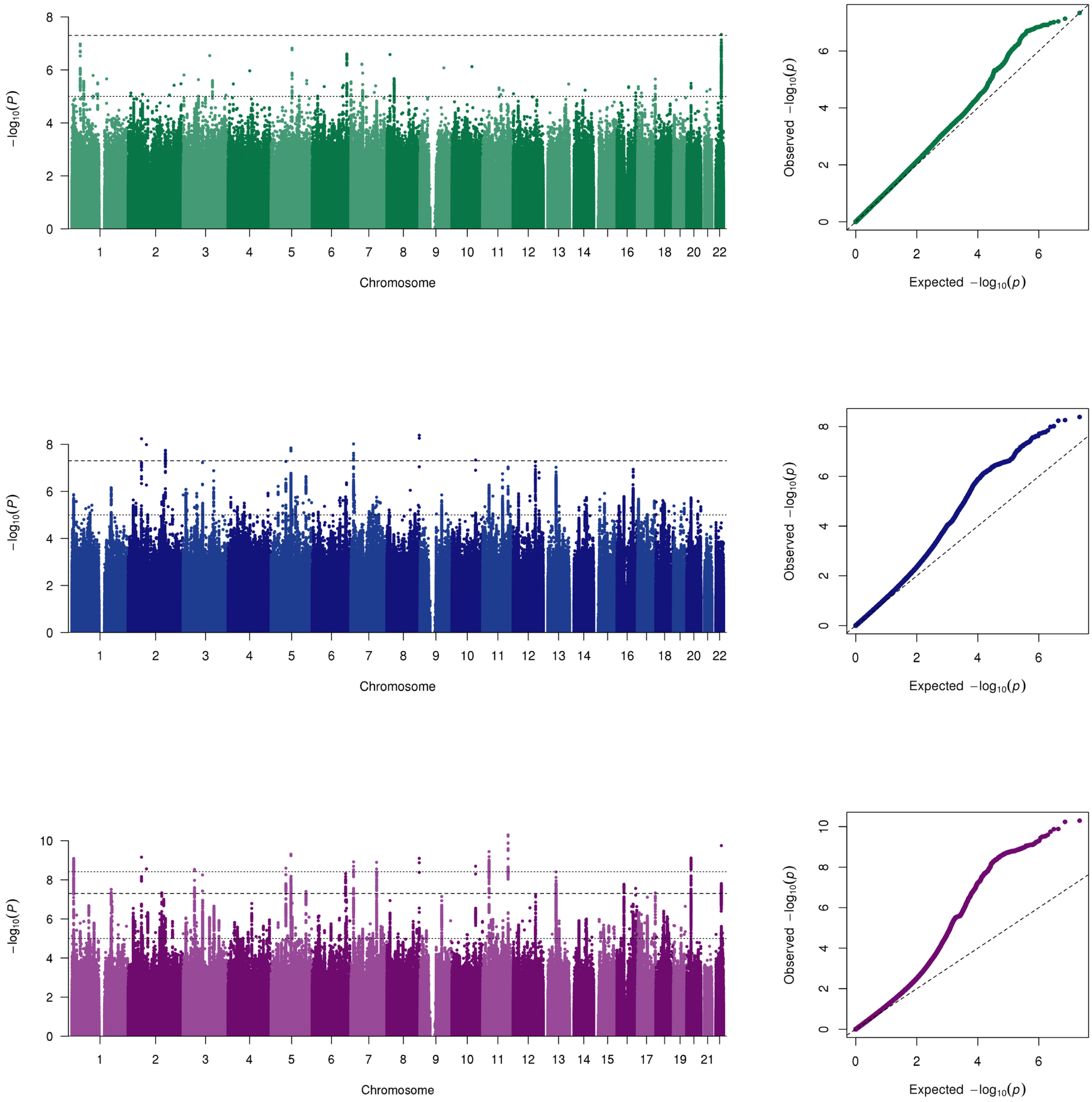

SUF-NERV - Do you suffer from ‘nerves’?

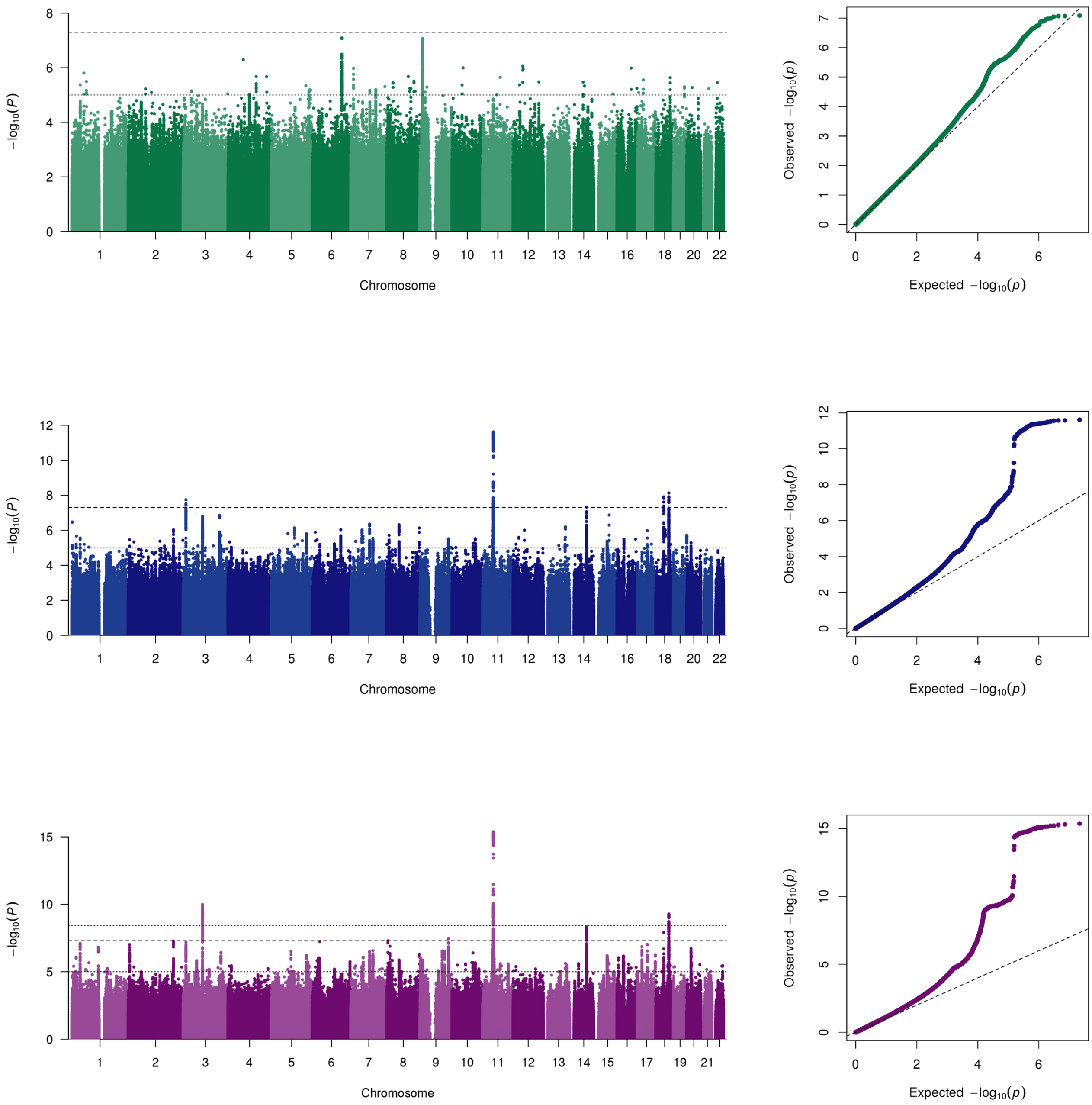

HURT - Are your feelings easily hurt?

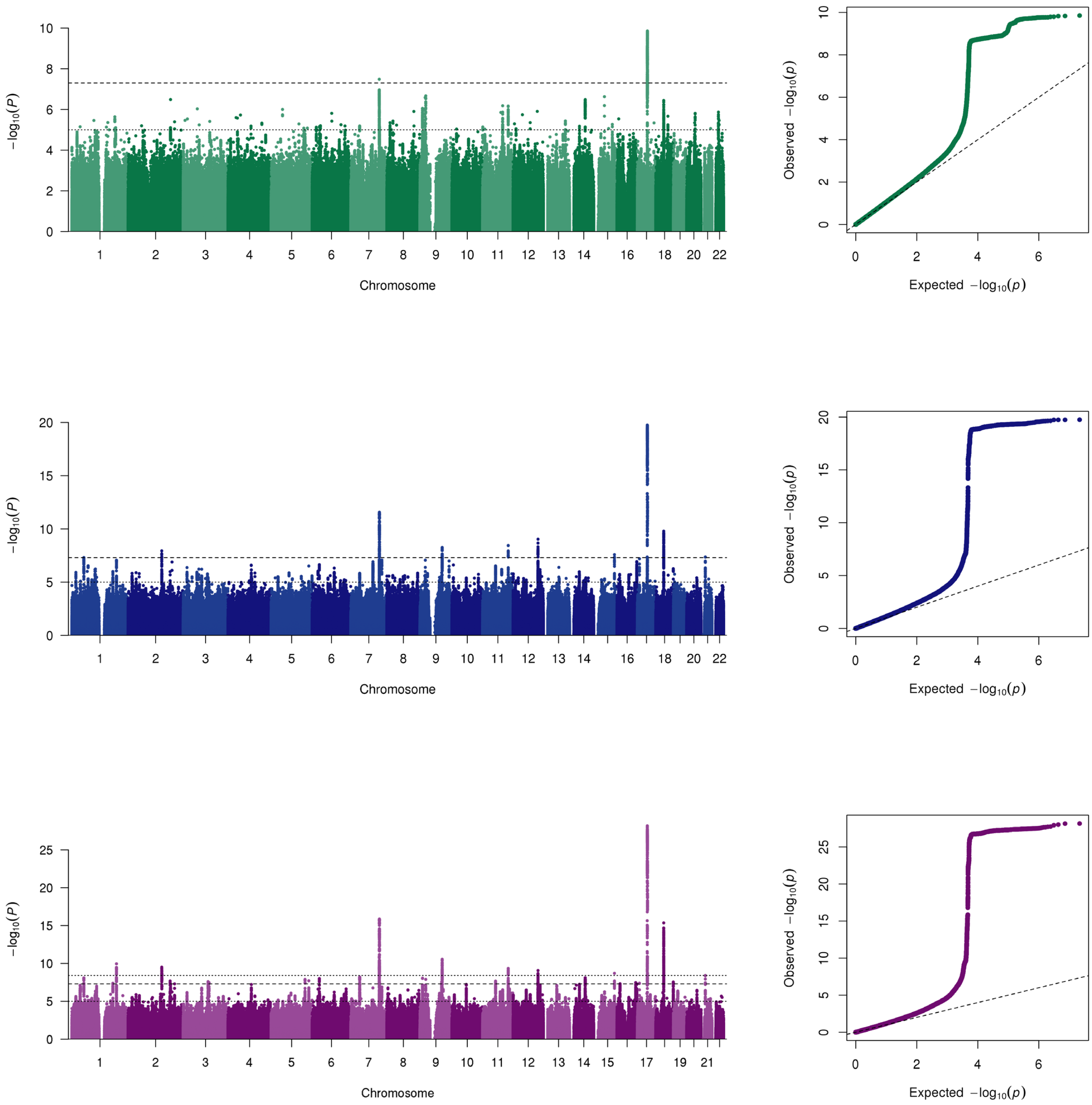

WORR-EMB - Do you worry too long after an embarrassing experience?

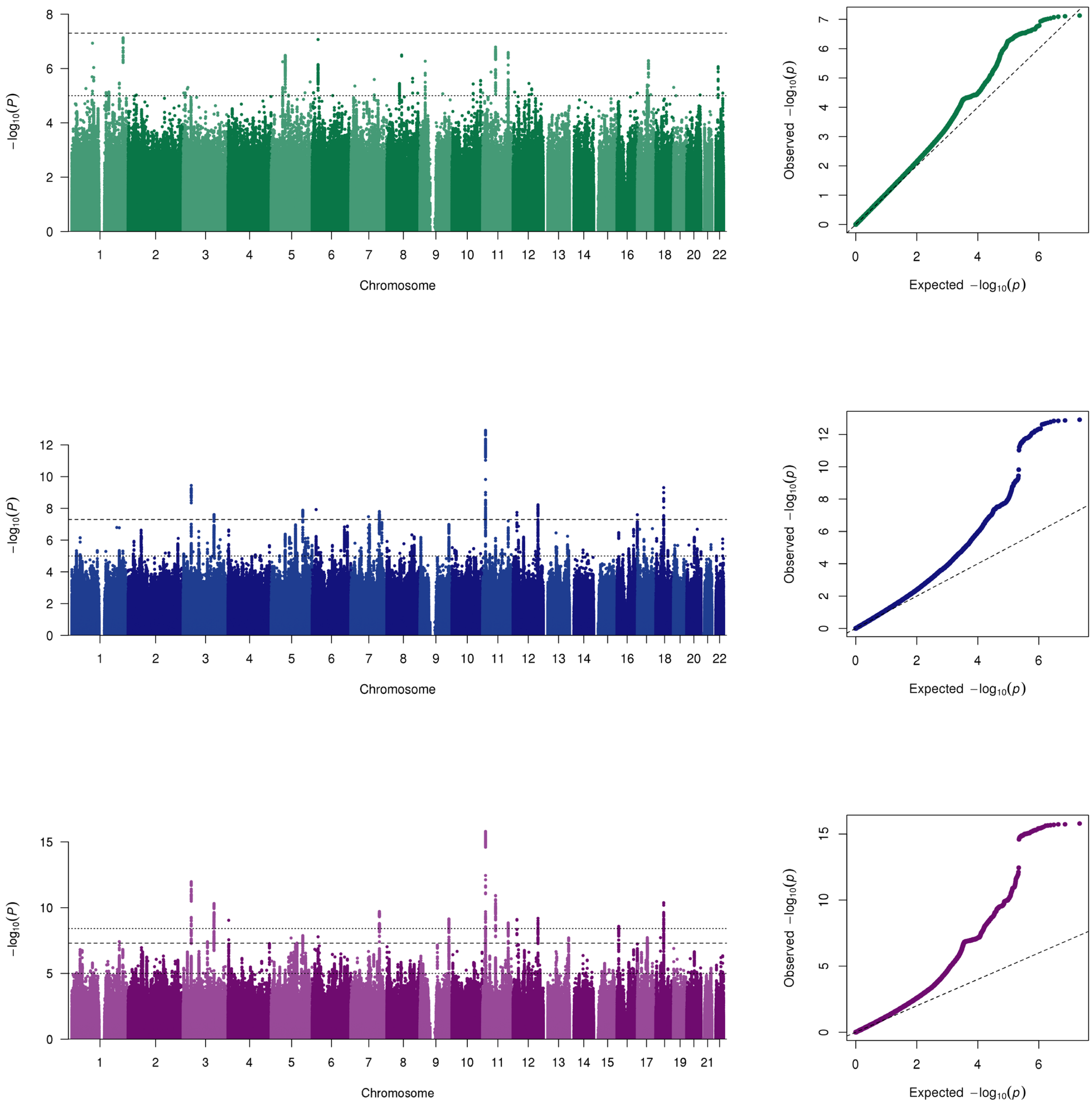

GUILT - Are you often troubled by feelings of guilt?

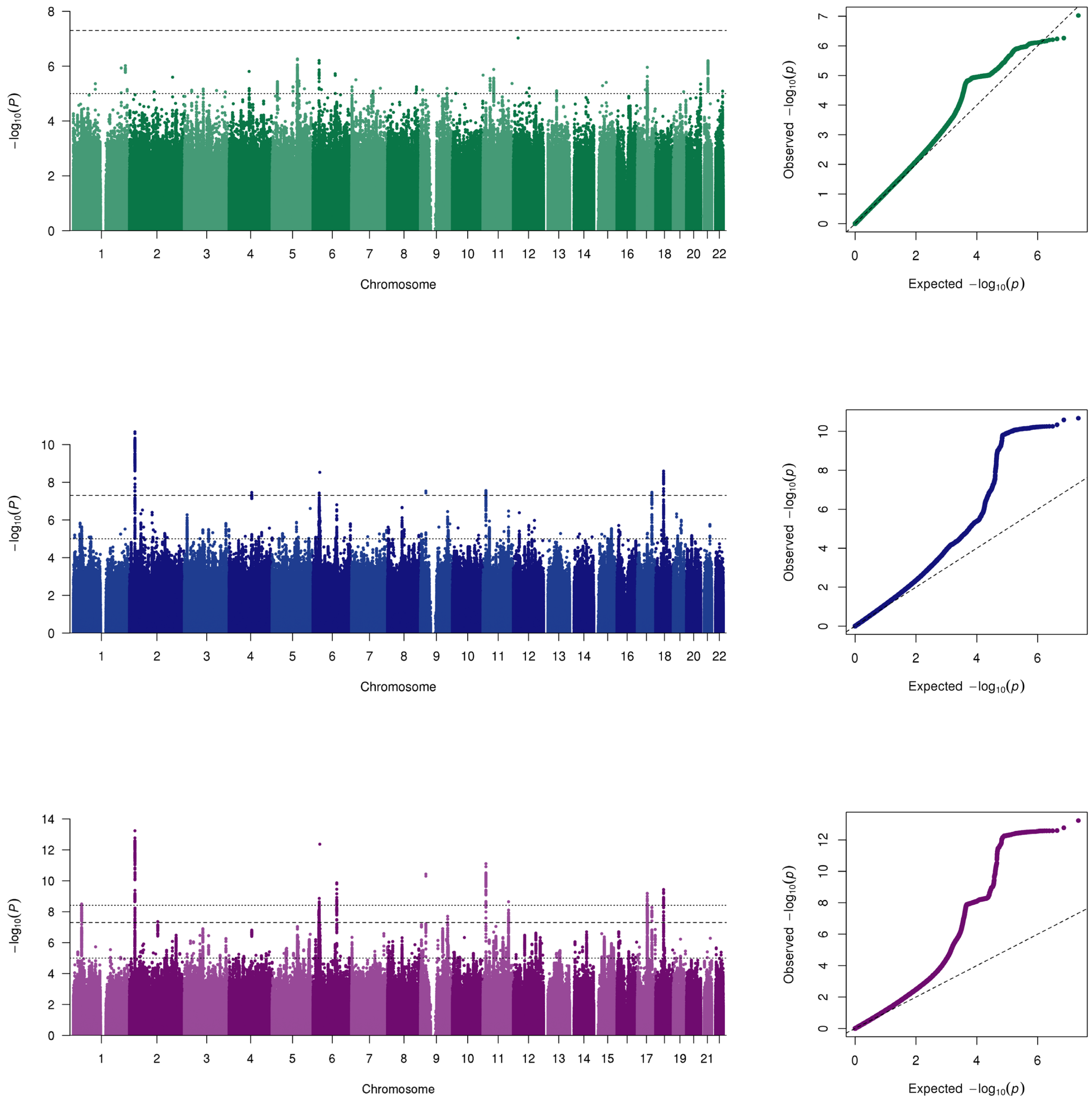

SUM-SCORE - Neuroticism sum-score

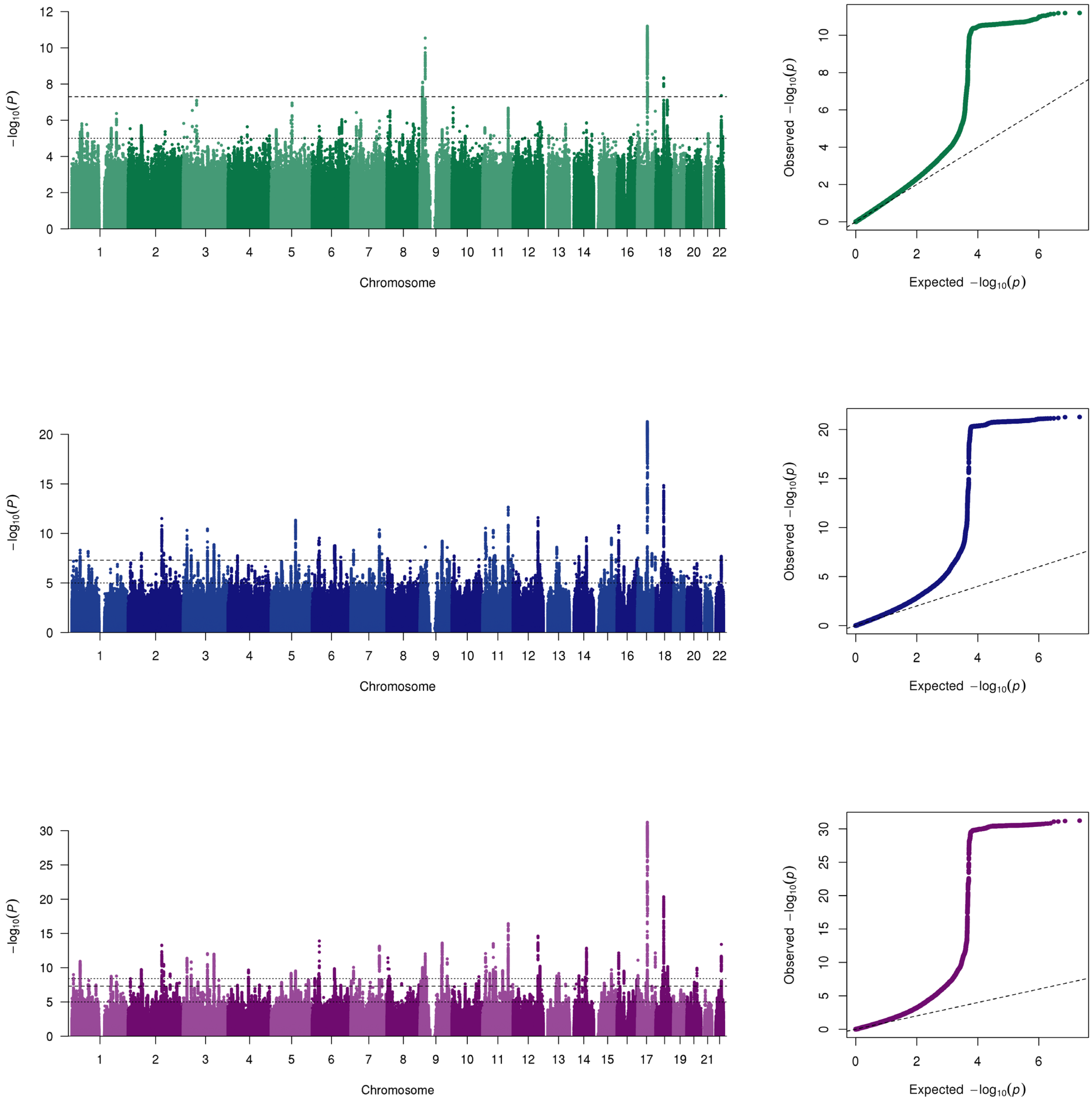

Supplementary Figure 4. Manhattan and quantile-quantile (Q-Q) plots of the SNP-based association results for the 12 individual items and the neuroticism sum-score.
SNP association results from the GWAS on the 12 neuroticism items and the sum-score in sample 1 (green), sample 2 (blue) and the meta-analysis (purple), respectively. Each dot represents a SNP. The *y*-axis shows the –log10 *P* values and the *x*-axis the chromosomal position of a given SNP. Dashed lines indicate genome-wide significance ($P < 5\times10^{-8}$) and dotted lines indicate the 'suggestive' significance threshold ($P < 1\times10^{-5}$). The upper dotted line in the Manhattan plots of the meta-analyses indicates a more stringent Bonferroni-corrected significance threshold, where we corrected for testing 13 phenotypes; $P < 5\times10^{-8}/13$ (3.85×10^{-9}). See **Supplementary Table 1** for an explanation of item labels.

rs7535528

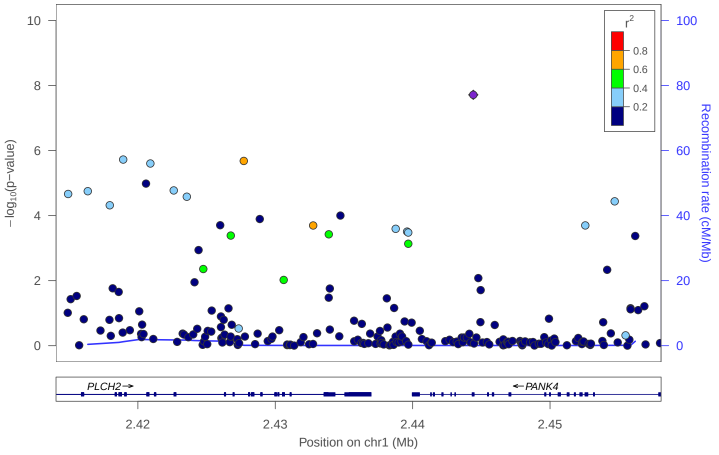

rs4411173

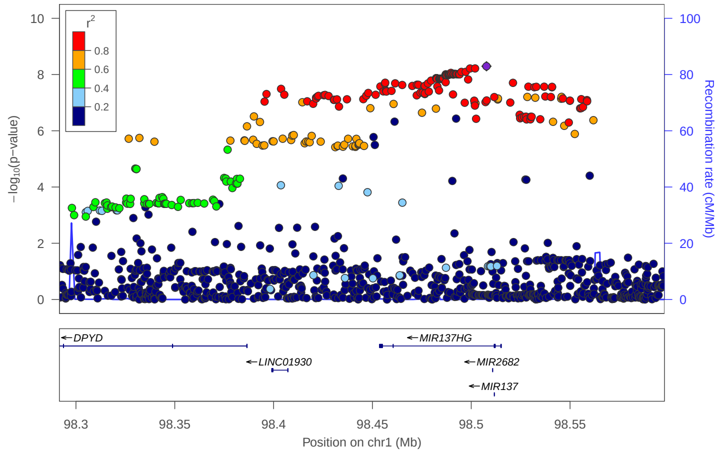

rs4953152

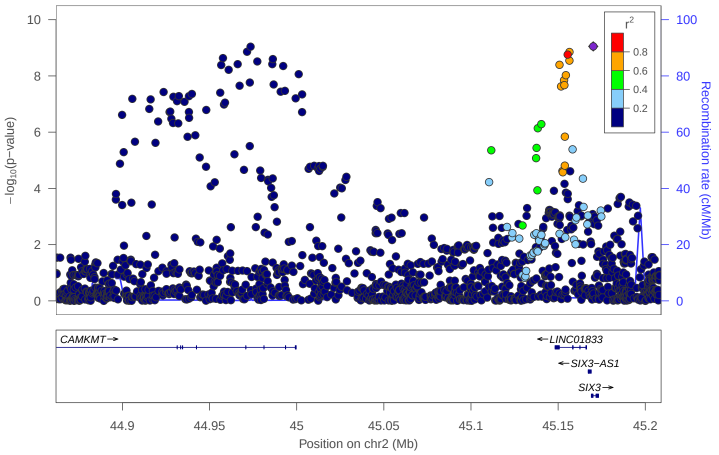

rs11682175

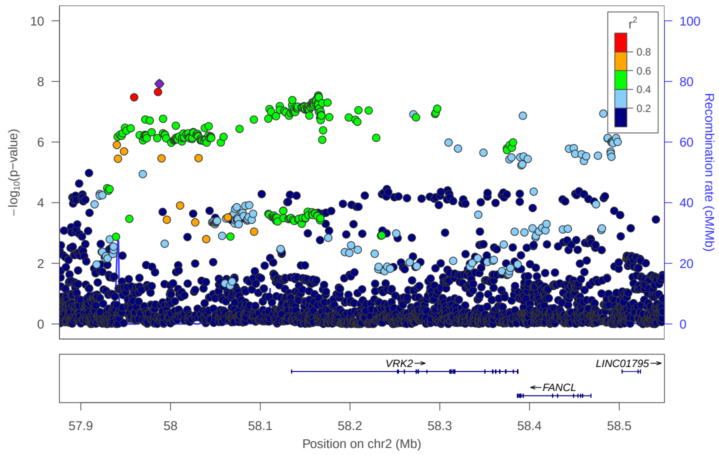

rs6718682

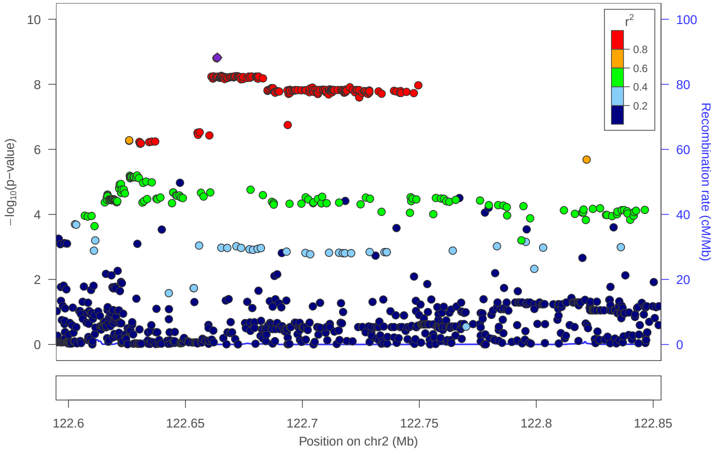

rs6711058

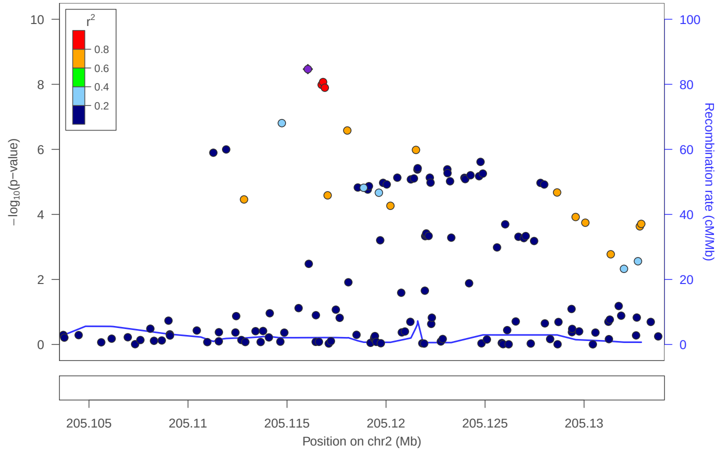

rs1542212

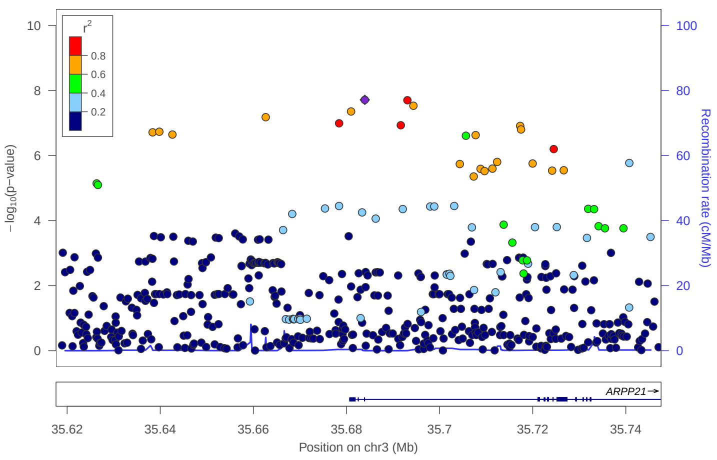

rs3774800

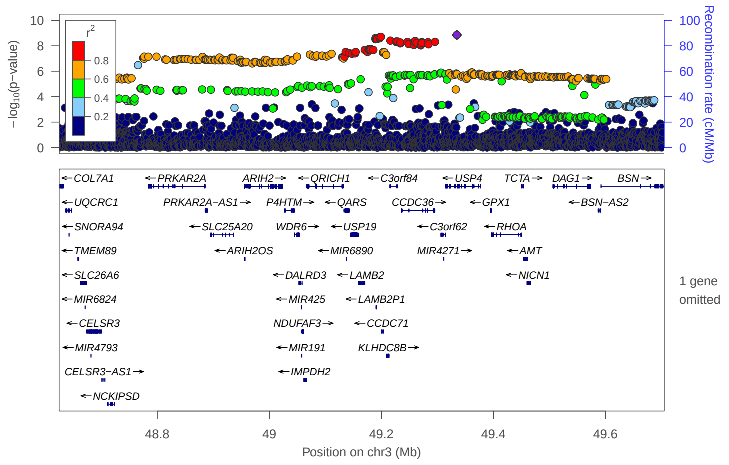

rs6549048

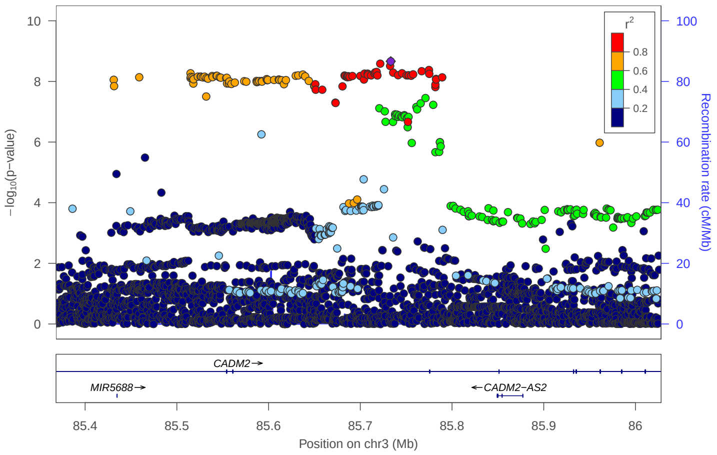

rs3772556

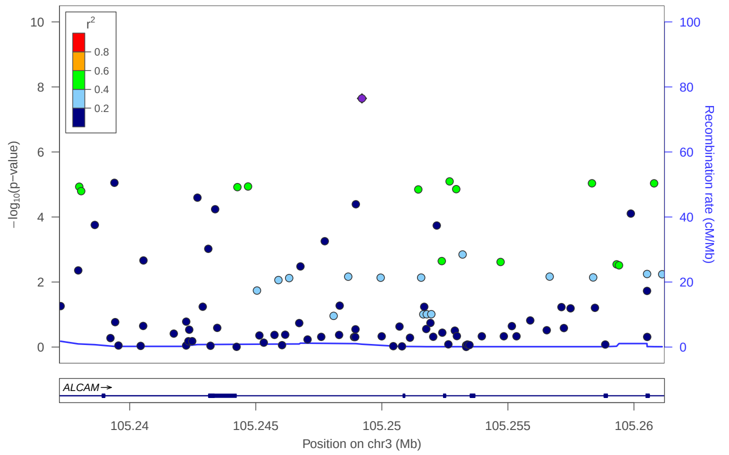

rs507078

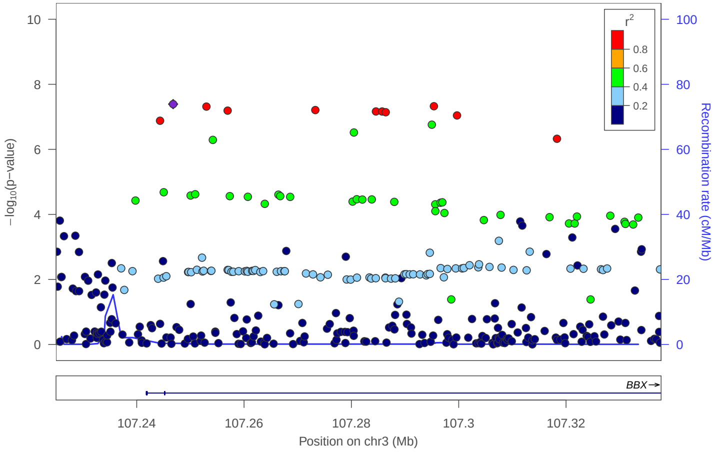

rs35255819

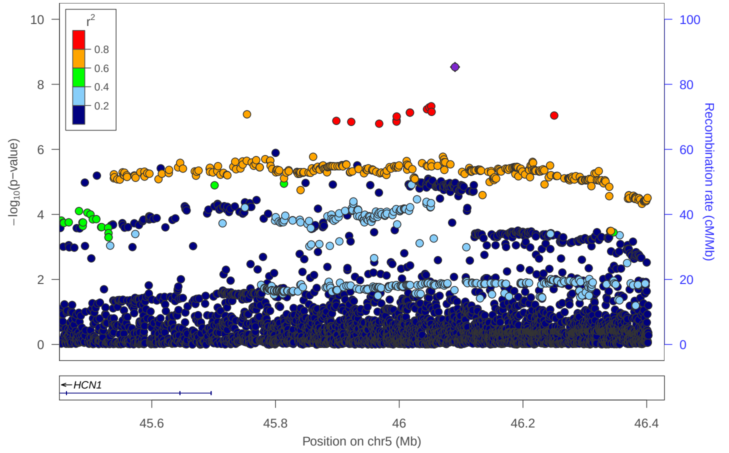

rs1422192

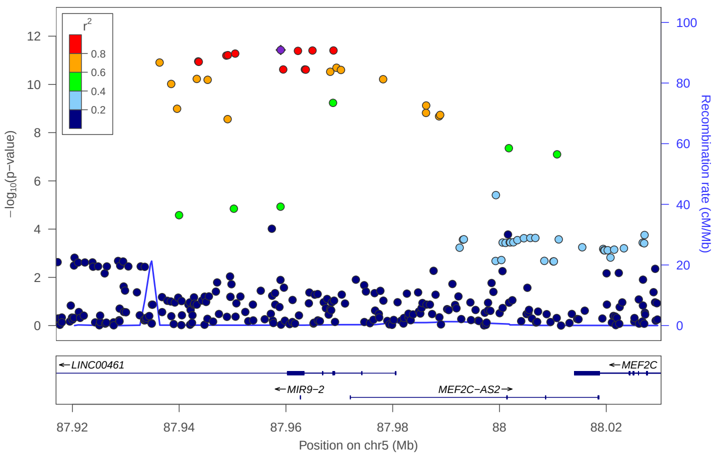

rs6596771

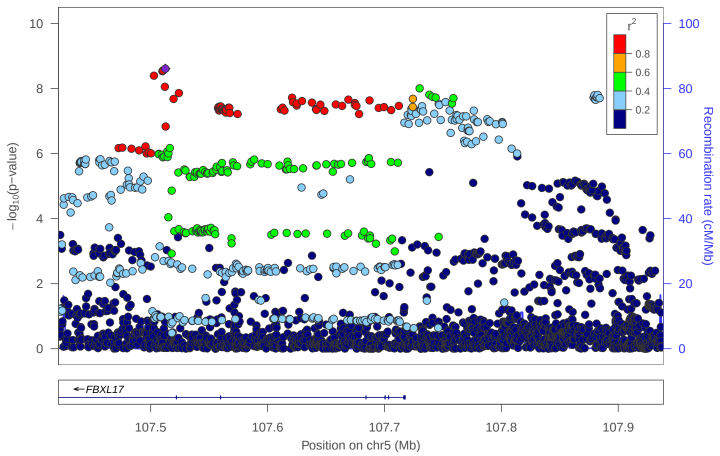

rs9403716

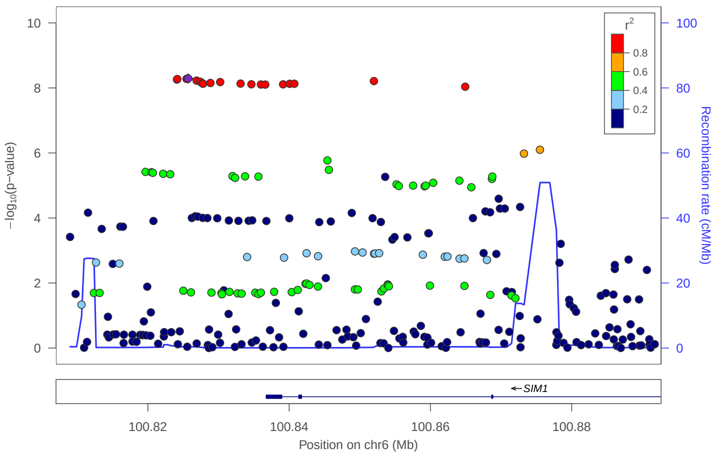

rs13223152

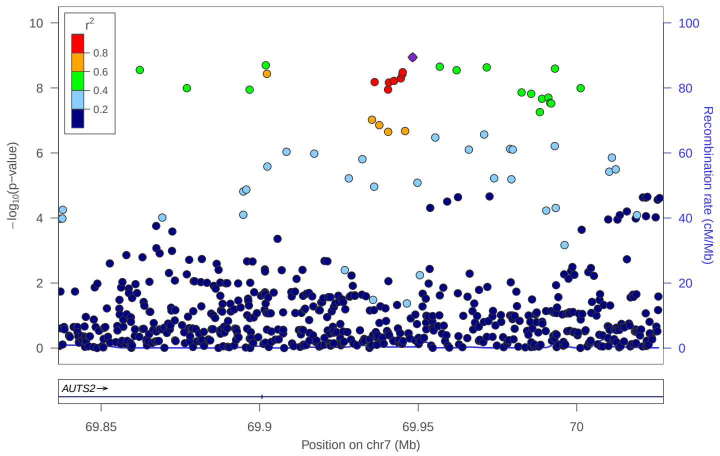

rs10228350

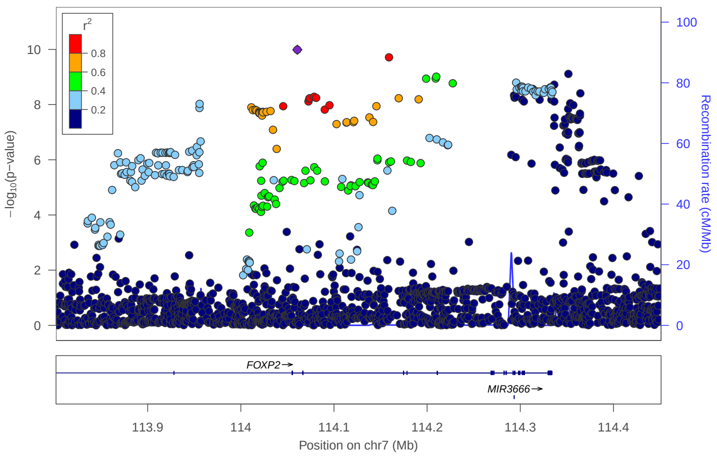

rs17151565

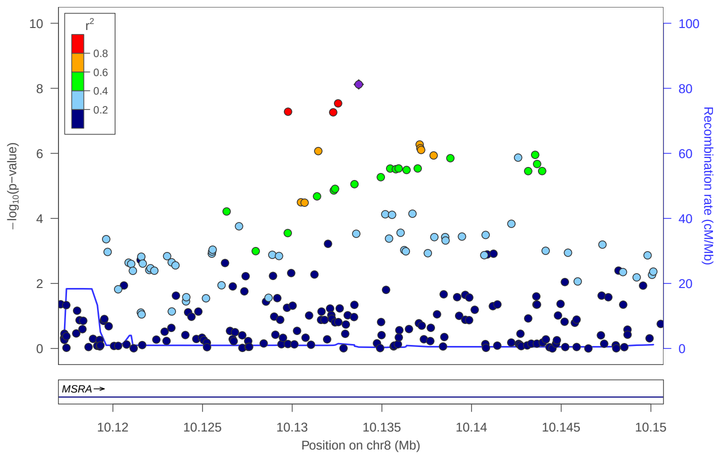

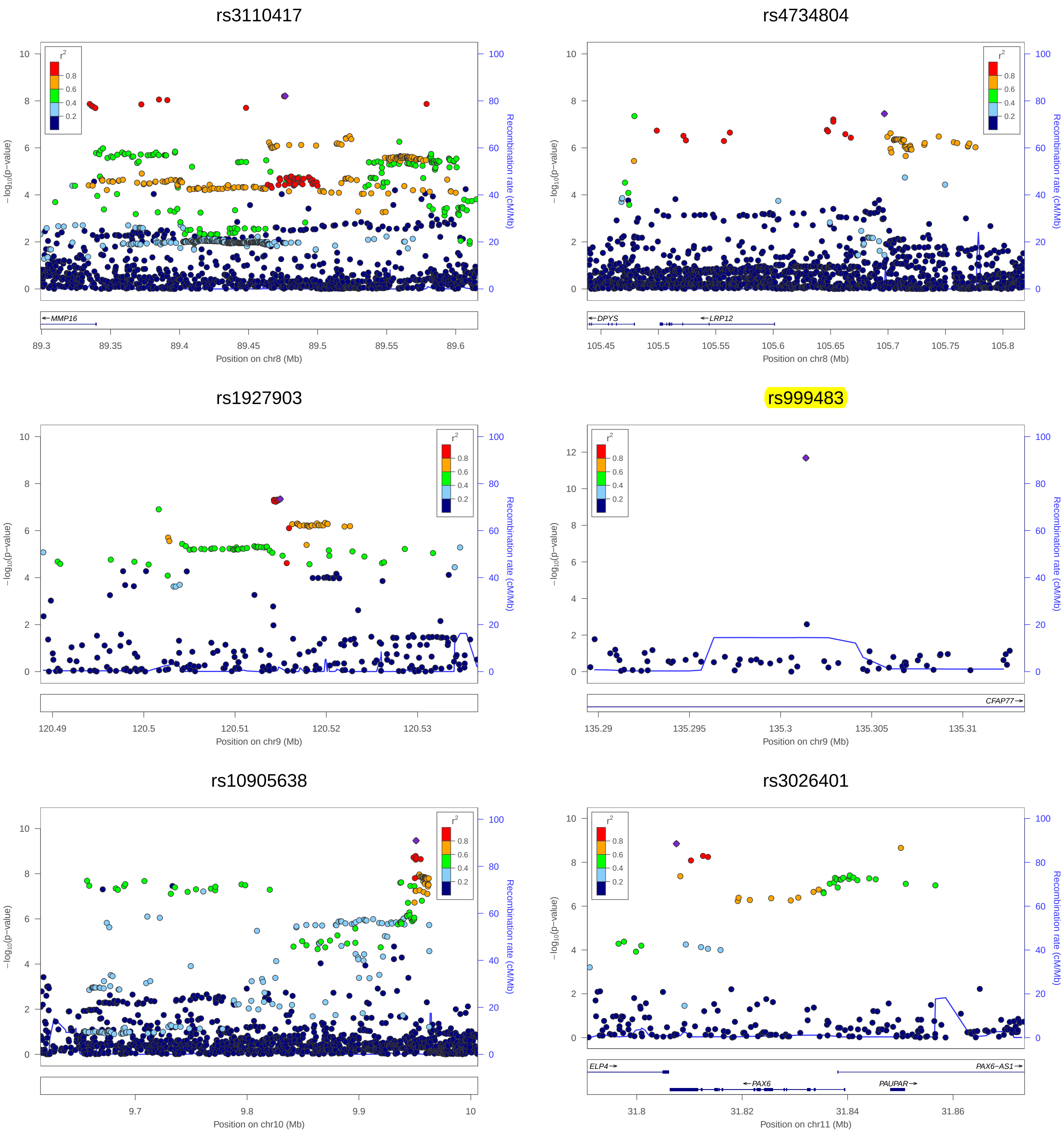
rs3110417
MMP16
Position on chr8 (Mb)
rs4734804
DPYS
LRP12
Position on chr8 (Mb)
rs1927903
Position on chr9 (Mb)
rs999483
CFAP77
Position on chr9 (Mb)
rs10905638
Position on chr10 (Mb)
rs3026401
ELP4
PAX6
PAUPAR
PAX6-AS1
Position on chr11 (Mb)
$-\log_{10}$(p-value)
Recombination rate (cM/Mb)
r^2

rs102275

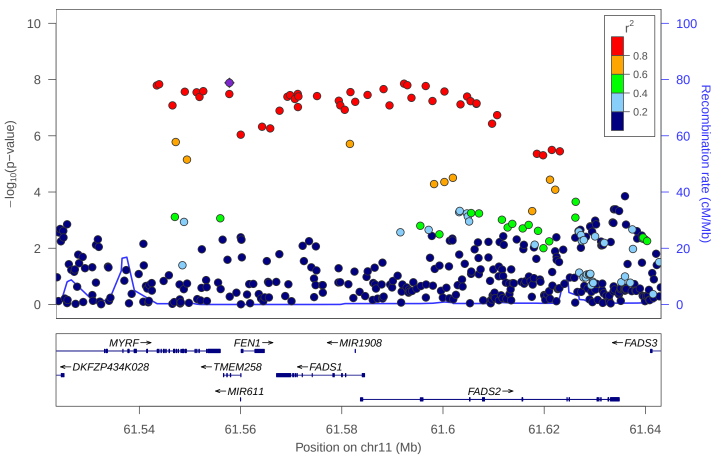

rs7973253

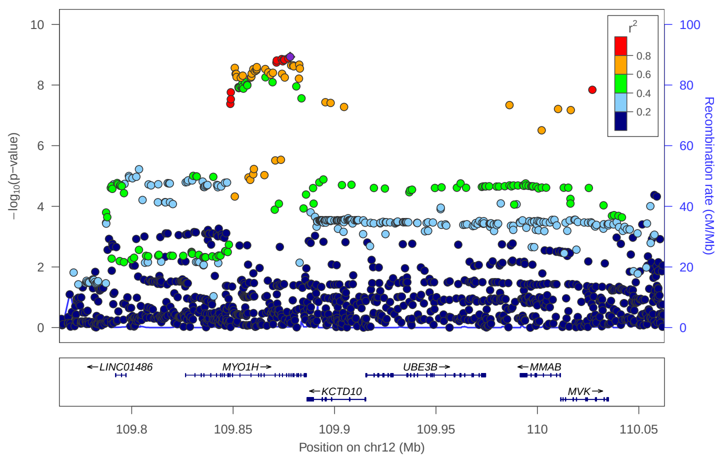

rs3124405

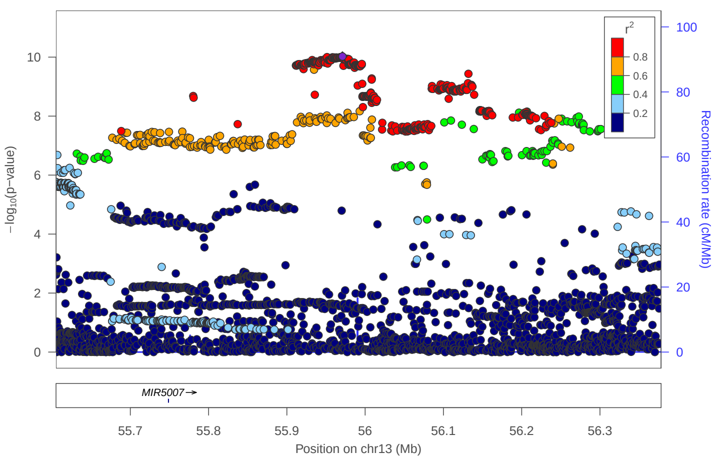

rs58446129

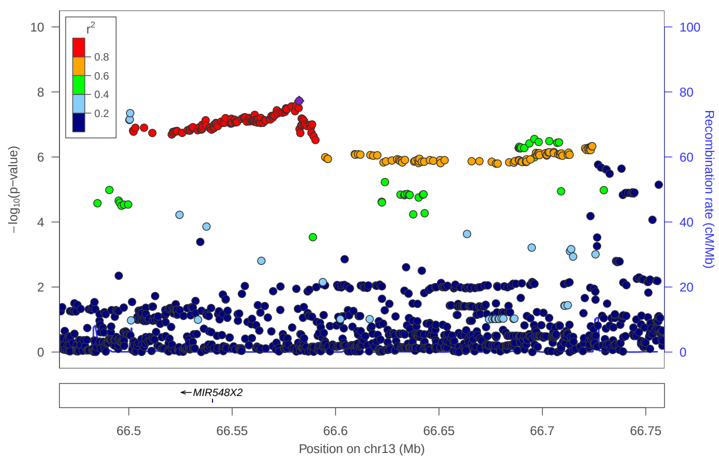

rs12886000

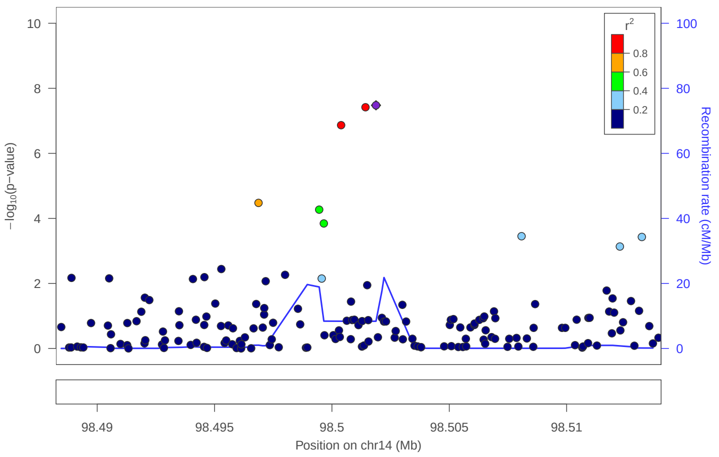

rs4781534

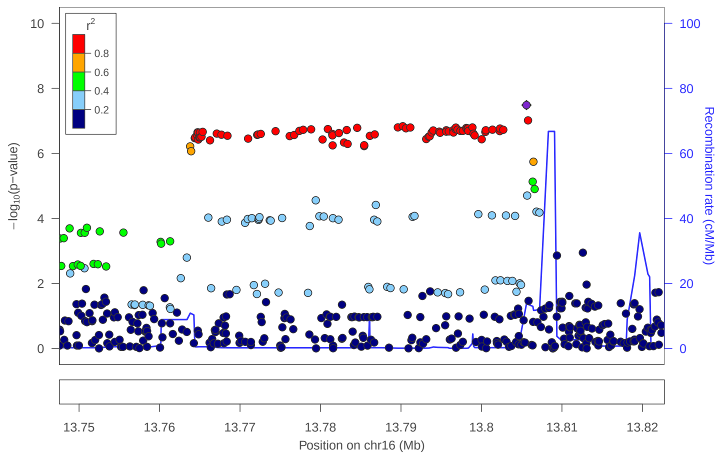

rs9938550

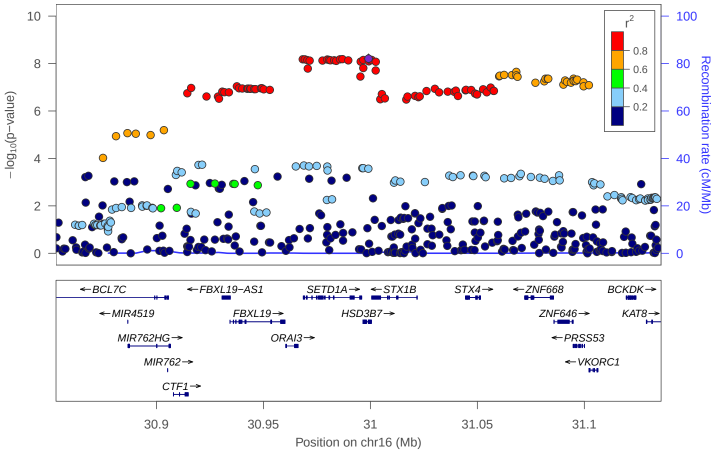

rs9630740

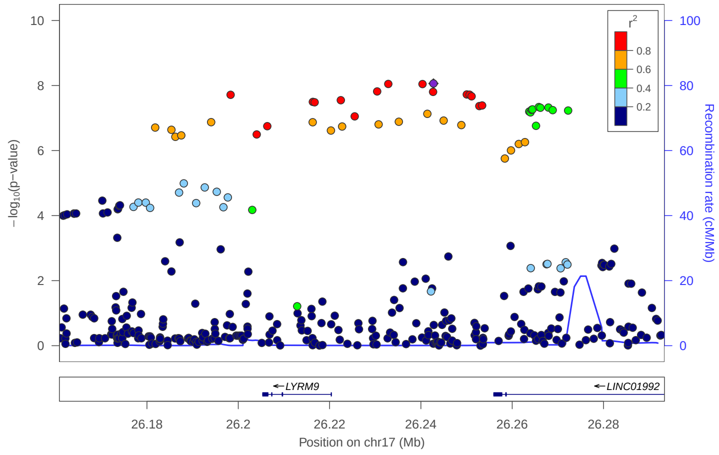

rs2106785

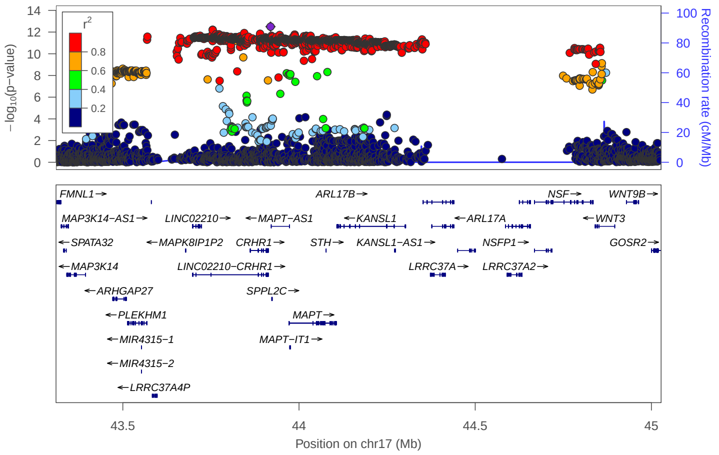

rs2217127

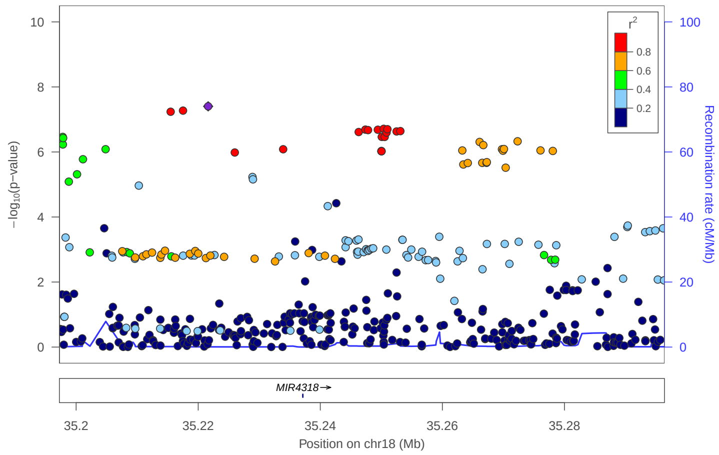

rs7231748

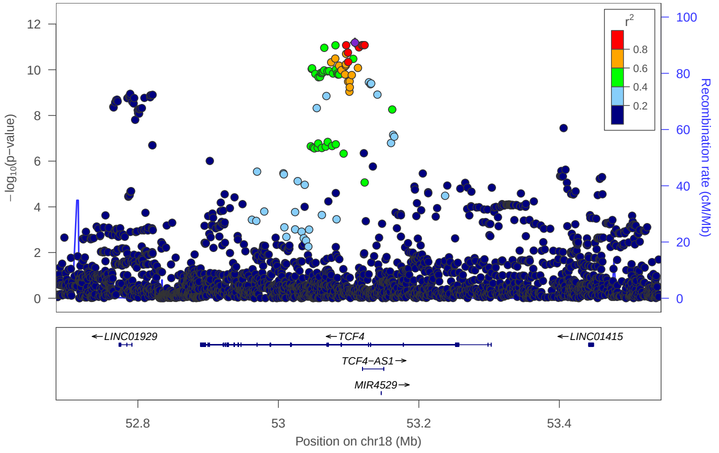

rs2587410

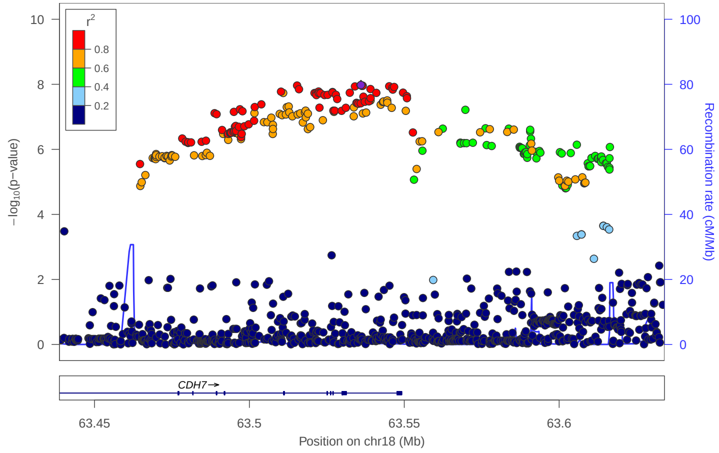

rs62211616

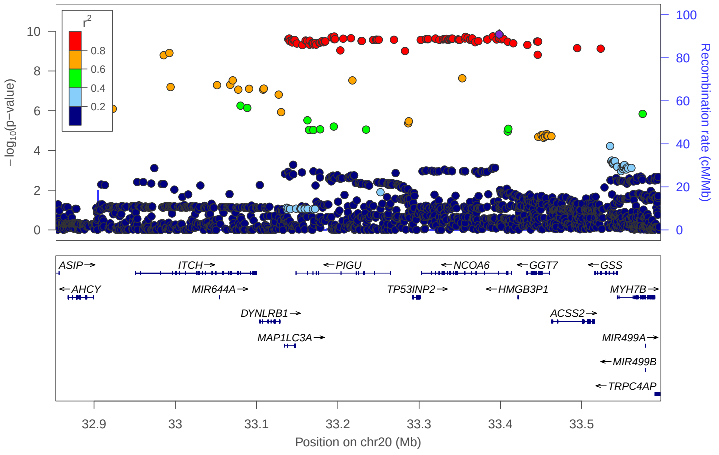

rs4820434

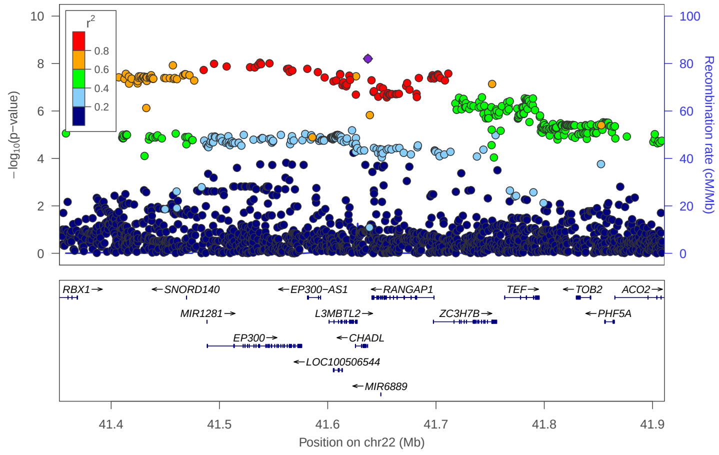

Supplementary Figure 5. Regional plots of GWA analyses of item IRR.

The *y*-axis represents the negative logarithm (base 10) of the SNP *P* value and the *x*-axis the position on the chromosome, with the name and location of genes in the UCSC Genome Browser in the bottom panel. The SNP with the lowest *P* value in the region is marked by a purple diamond. The colors of the other SNPs indicate the r^2 of these SNPs with the lead SNP, with grey color indicating SNPs below the user-defined r^2. Plots are generated with LocusZoom. See **Supplementary Data 2** for an overview of all lead SNPs for which a regional plot is presented above. For excluded loci (see **Methods; Supplementary Data 3-4**) the rsID's are marked yellow.

rs74338595

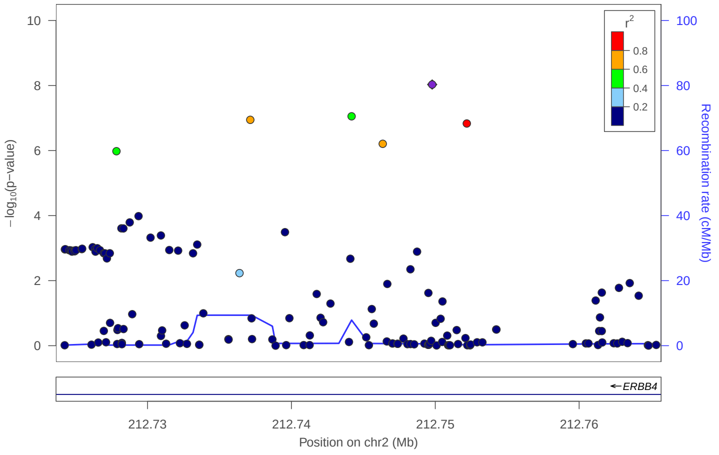

rs4958586

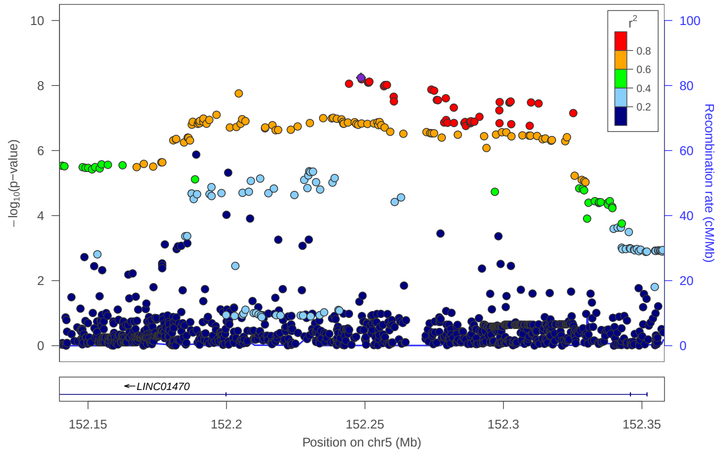

rs12554512

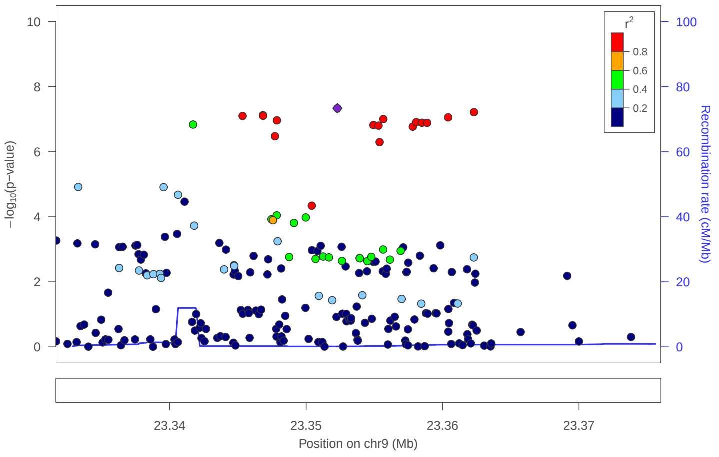

rs7044244

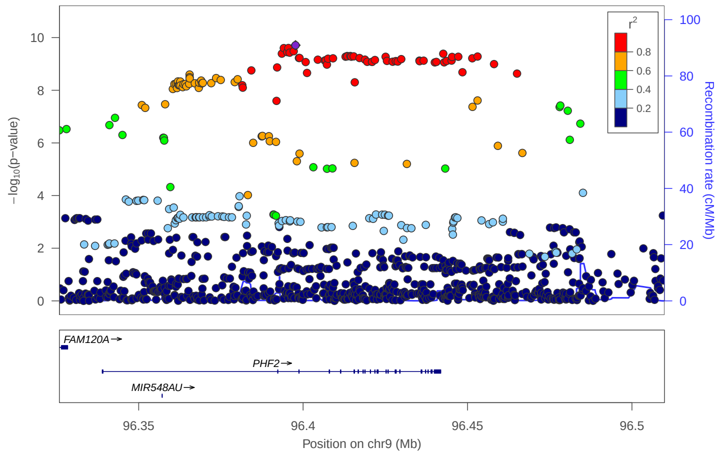

rs11039389

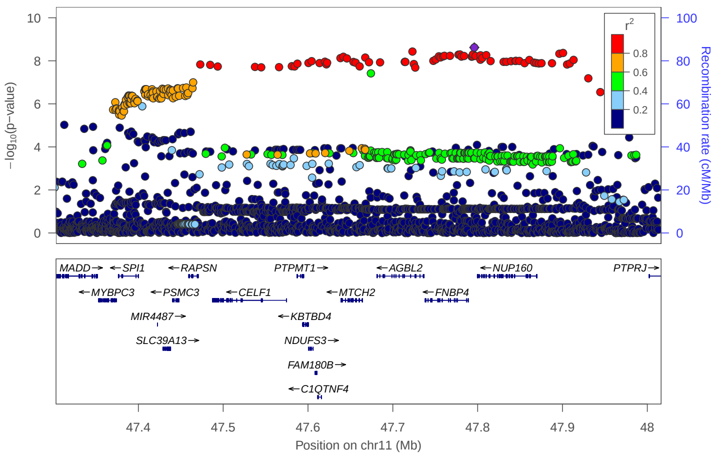

rs7209581

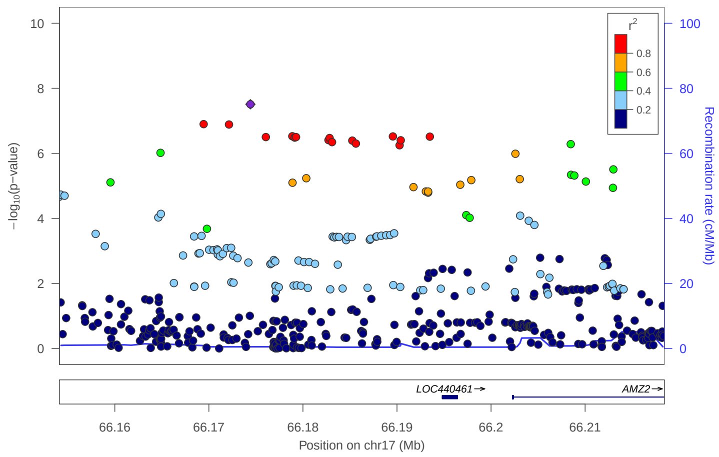

rs599550

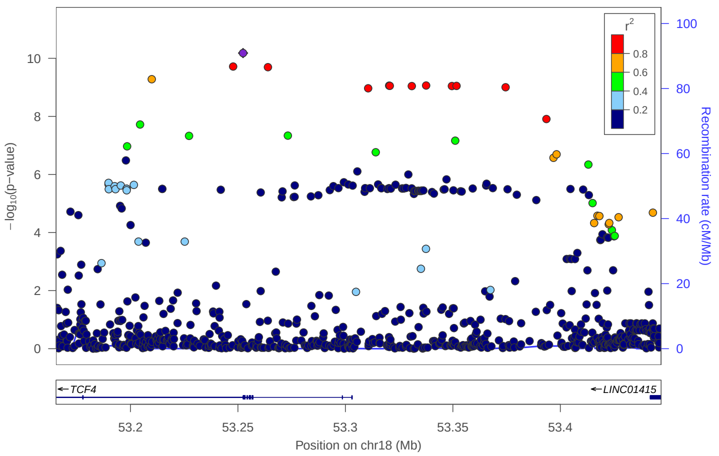

10
8
6
4
2
0
− log10(p−value)
100
80
60
40
20
0
Recombination rate (cM/Mb)
r2
0.8
0.6
0.4
0.2
TCF4
LINC01415
53.2
53.25
53.3
53.35
53.4
Position on chr18 (Mb)

Supplementary Figure 6. Regional plots of GWA analyses of item LONE.

The *y*-axis represents the negative logarithm (base 10) of the SNP *P* value and the *x*-axis the position on the chromosome, with the name and location of genes in the UCSC Genome Browser in the bottom panel. The SNP with the lowest *P* value in the region is marked by a purple diamond. The colors of the other SNPs indicate the r^2 of these SNPs with the lead SNP, with grey color indicating SNPs below the user-defined r^2. Plots are generated with LocusZoom. See **Supplementary Data 2** for an overview of all lead SNPs for which a regional plot is presented above.

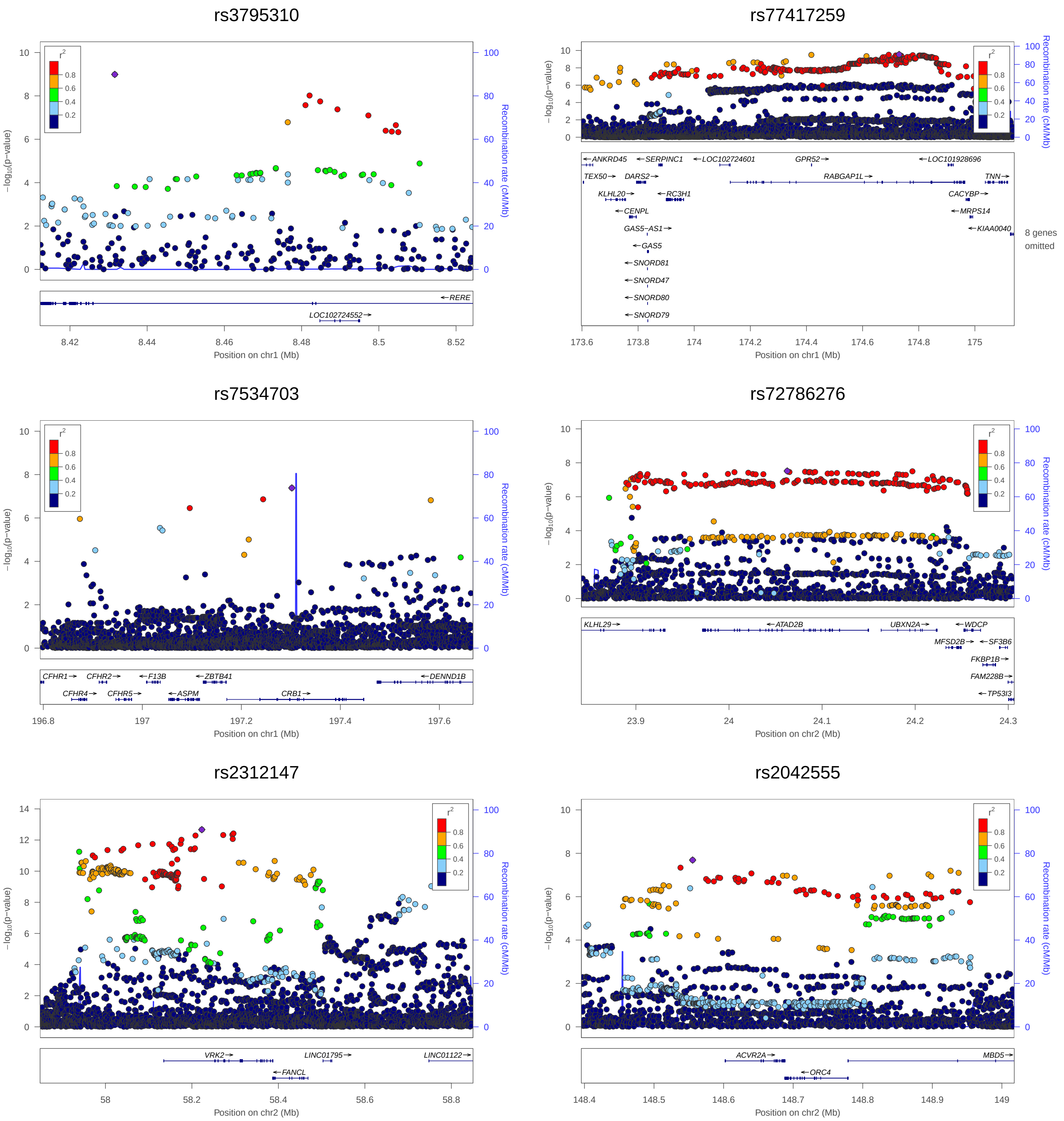

rs3795310
rs77417259
rs7534703
rs72786276
rs2312147
rs2042555
−log10(p−value)
Recombination rate (cM/Mb)
r2
0.8
0.6
0.4
0.2
RERE
LOC102724552
Position on chr1 (Mb)
ANKRD45
SERPINC1
LOC102724601
GPR52
LOC101928696
TEX50
DARS2
RABGAP1L
TNN
KLHL20
RC3H1
CACYBP
CENPL
MRPS14
GAS5−AS1
KIAA0040
GAS5
SNORD81
SNORD47
SNORD80
SNORD79
8 genes omitted
CFHR1
CFHR2
F13B
ZBTB41
DENND1B
CFHR4
CFHR5
ASPM
CRB1
KLHL29
ATAD2B
UBXN2A
WDCP
MFSD2B
SF3B6
FKBP1B
FAM228B
TP53I3
Position on chr2 (Mb)
VRK2
LINC01795
LINC01122
FANCL
ACVR2A
MBD5
ORC4

rs1994247

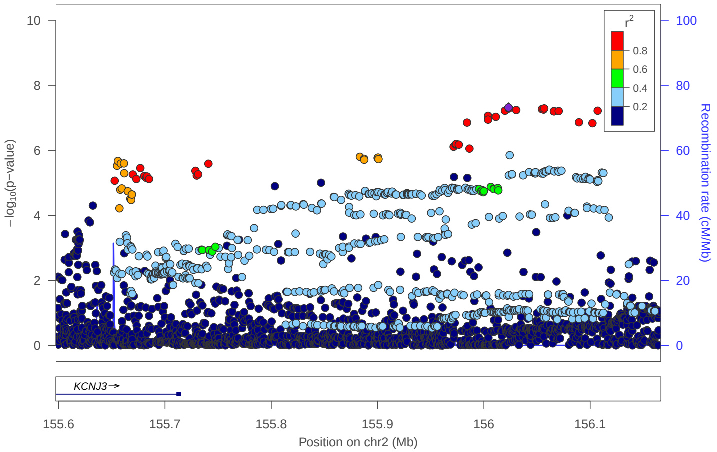

rs13018407

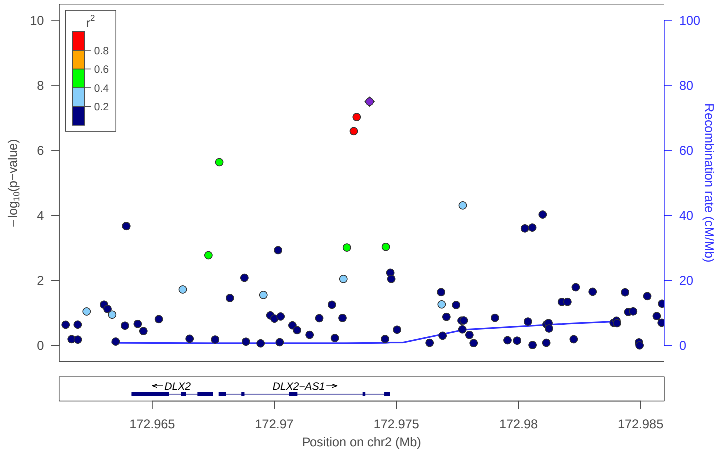

rs114306779

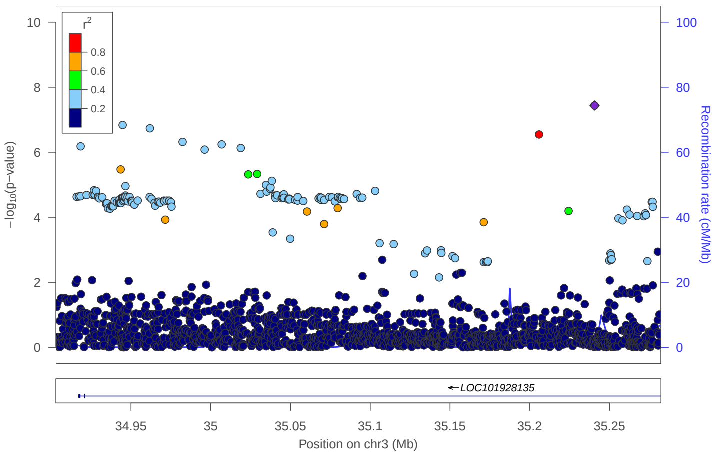

rs9586

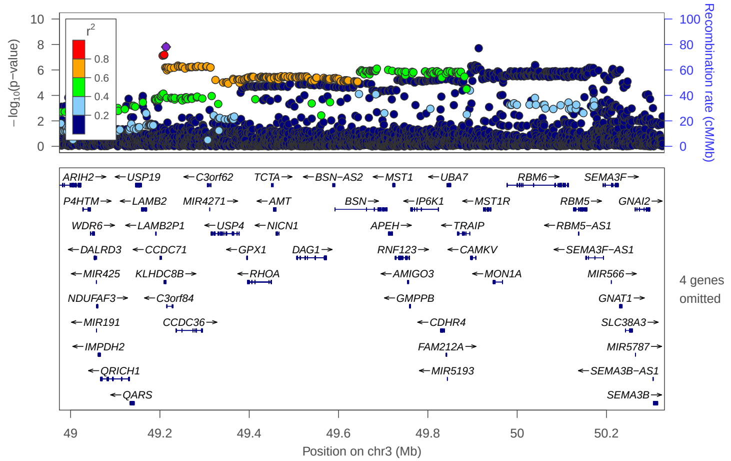

rs836927

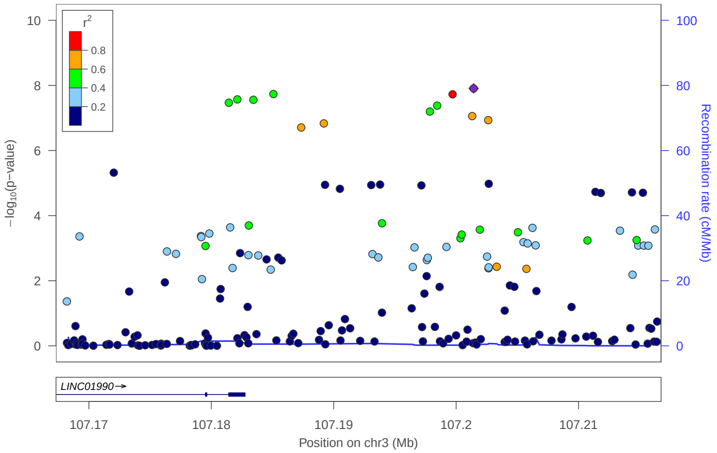

rs67932684

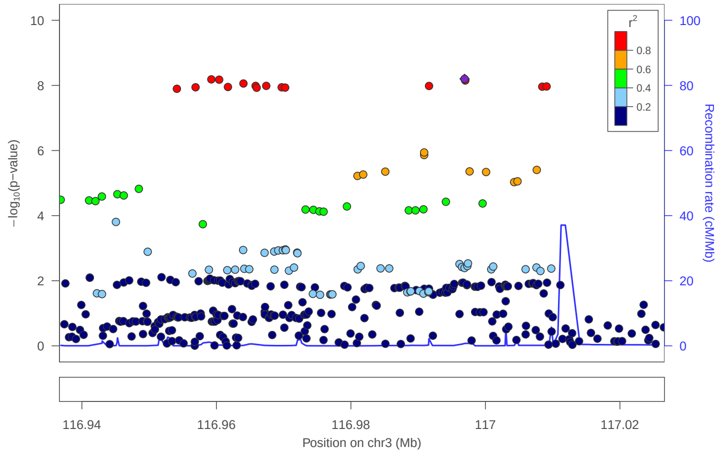

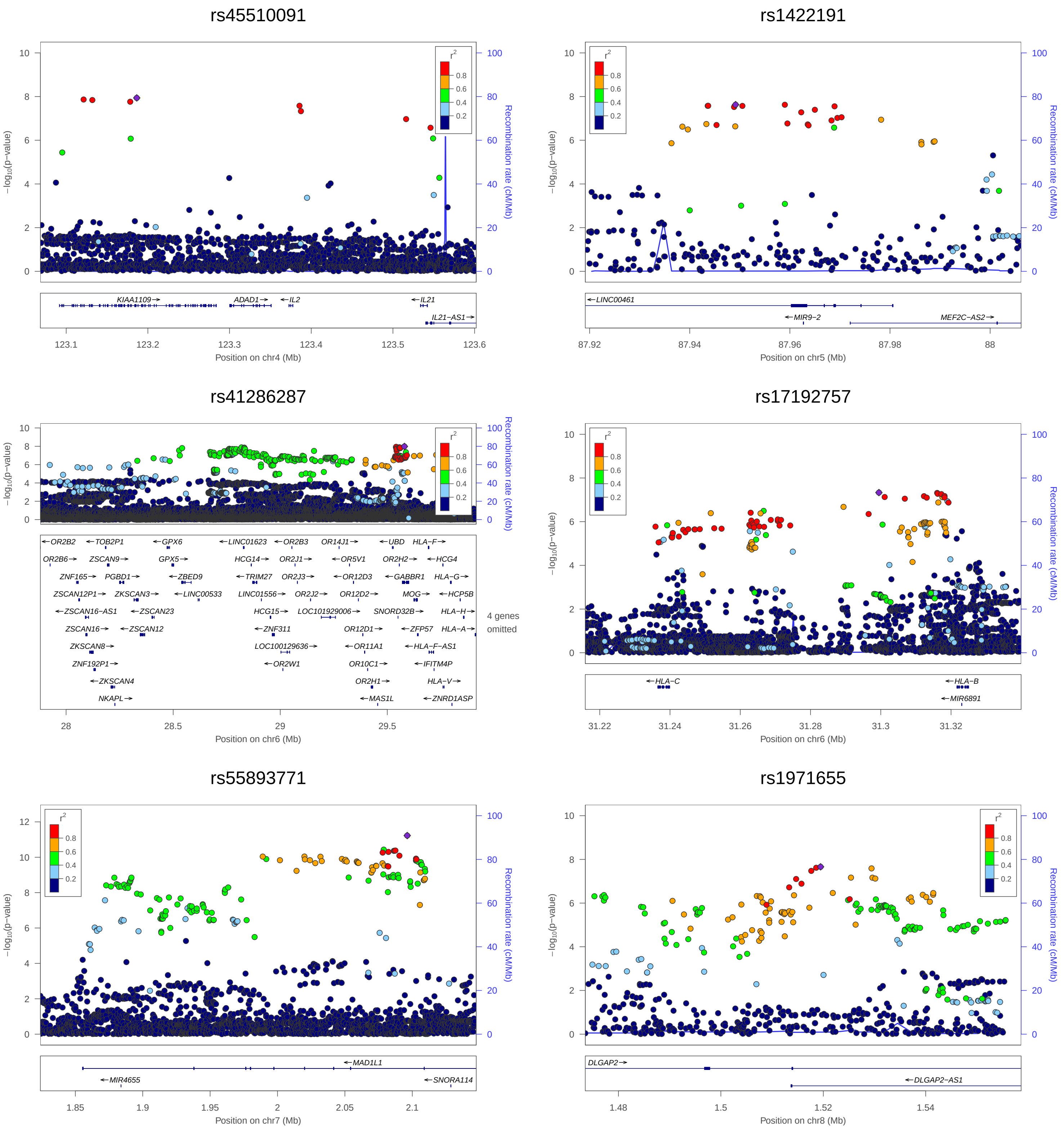

rs45510091
−log10(p−value)
Recombination rate (cM/Mb)
KIAA1109
ADAD1
IL2
IL21
IL21−AS1
Position on chr4 (Mb)
rs1422191
LINC00461
MIR9−2
MEF2C−AS2
Position on chr5 (Mb)
rs41286287
4 genes omitted
Position on chr6 (Mb)
rs17192757
HLA−C
HLA−B
MIR6891
Position on chr6 (Mb)
rs55893771
MAD1L1
MIR4655
SNORA114
Position on chr7 (Mb)
rs1971655
DLGAP2
DLGAP2−AS1
Position on chr8 (Mb)

rs10156548

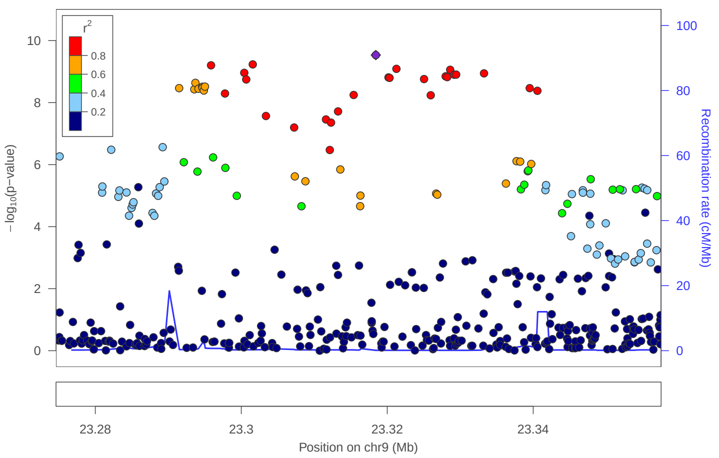

rs11599236

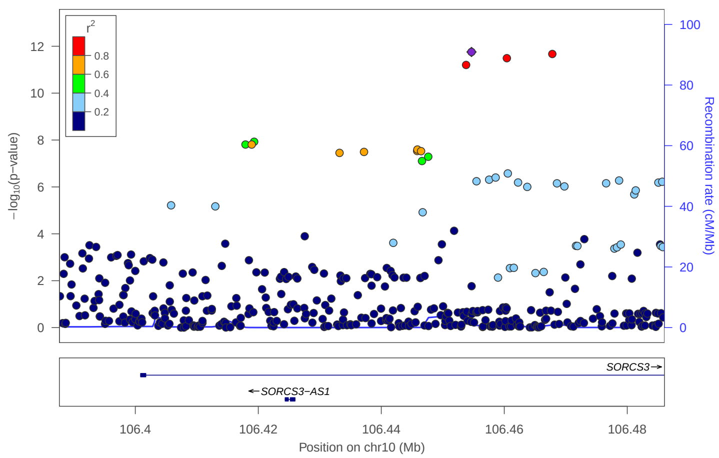

rs17805214

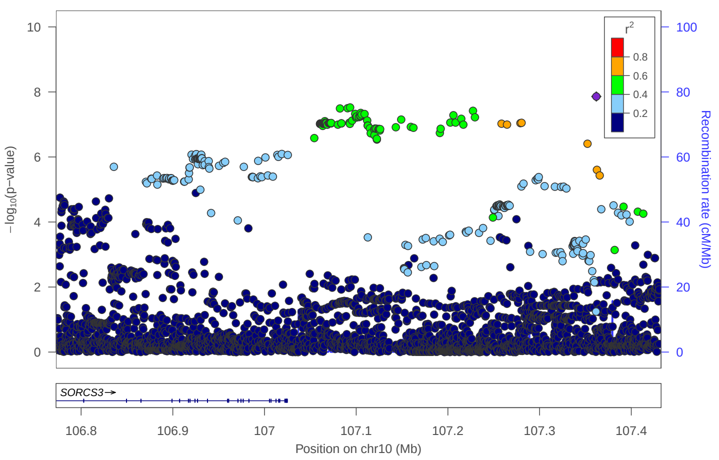

rs7912226

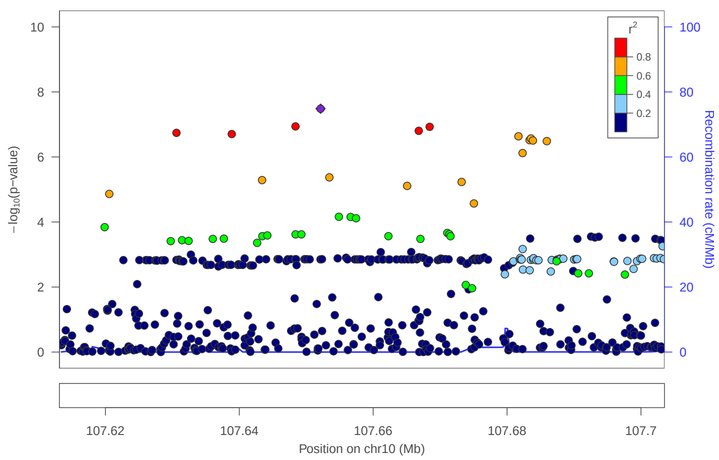

rs4757142

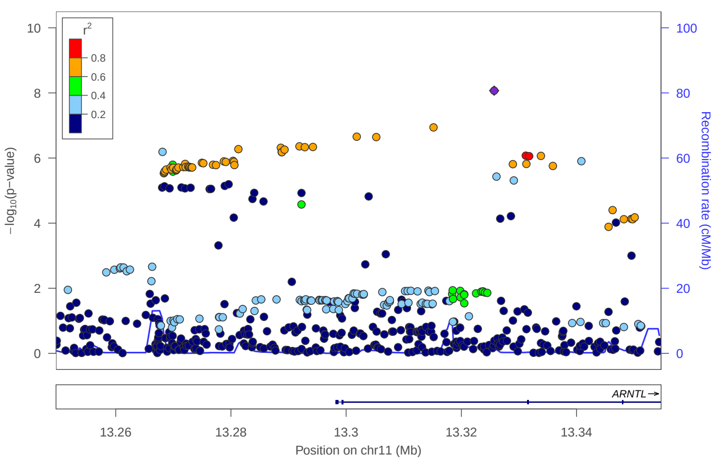

rs113829044

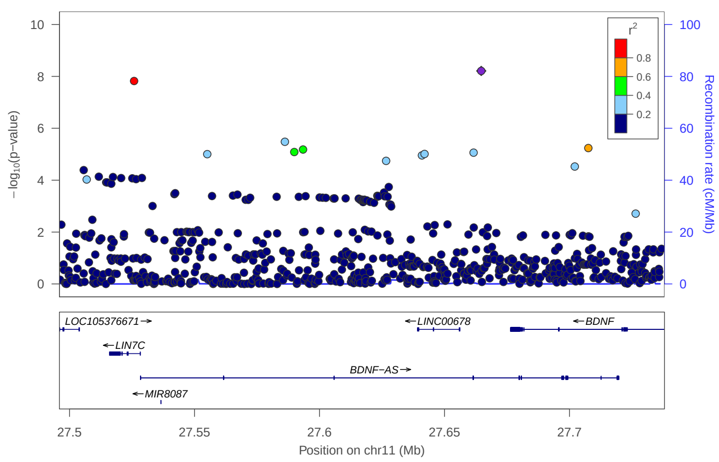

rs11039149

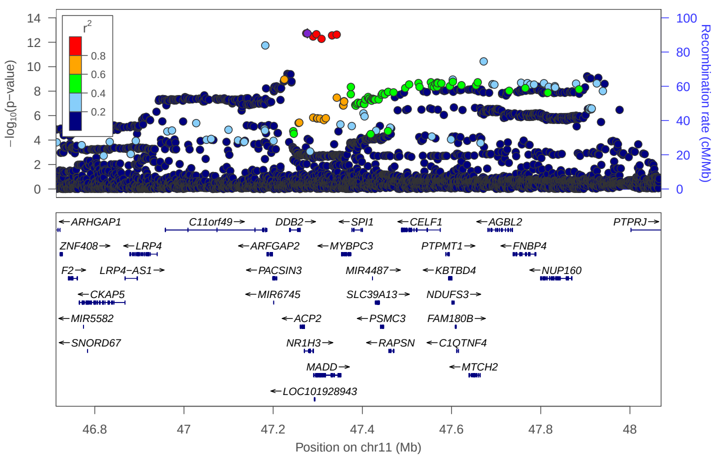

rs628246

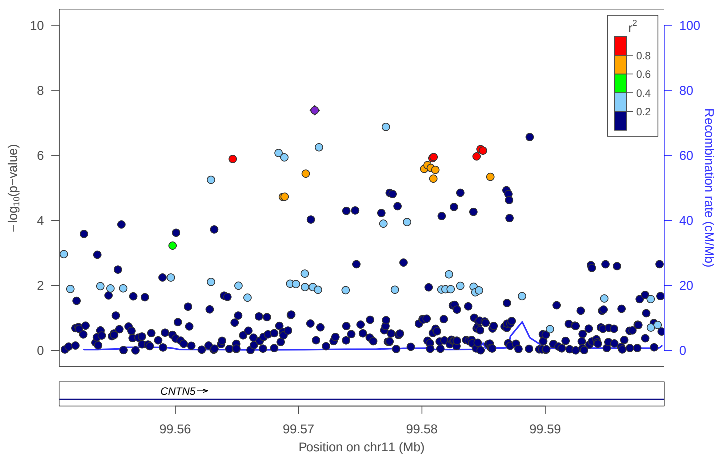

rs4424705

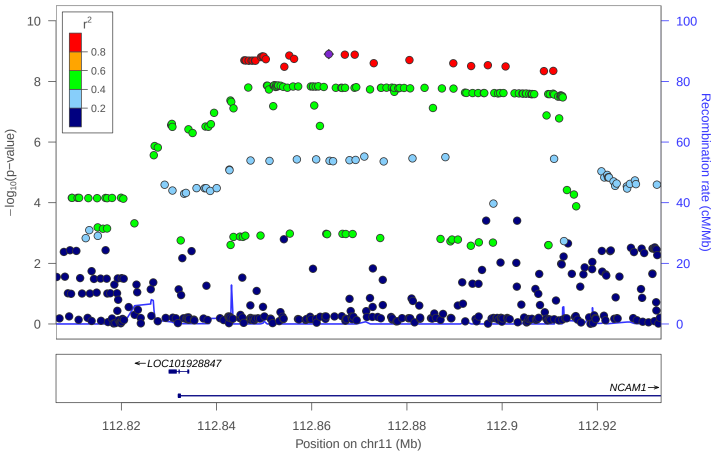

rs4245154

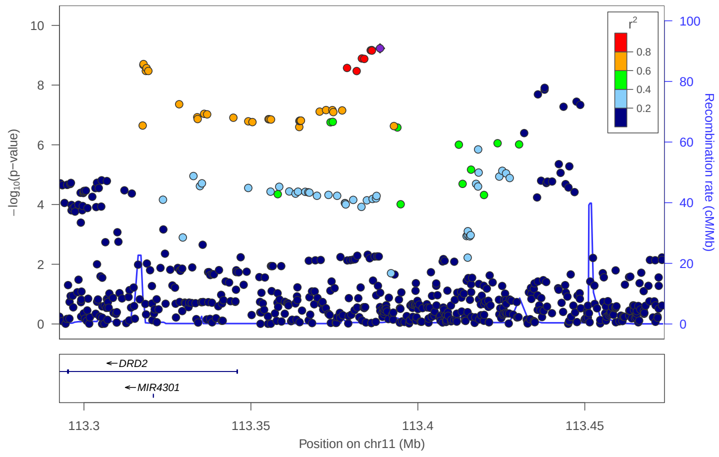

rs11608355

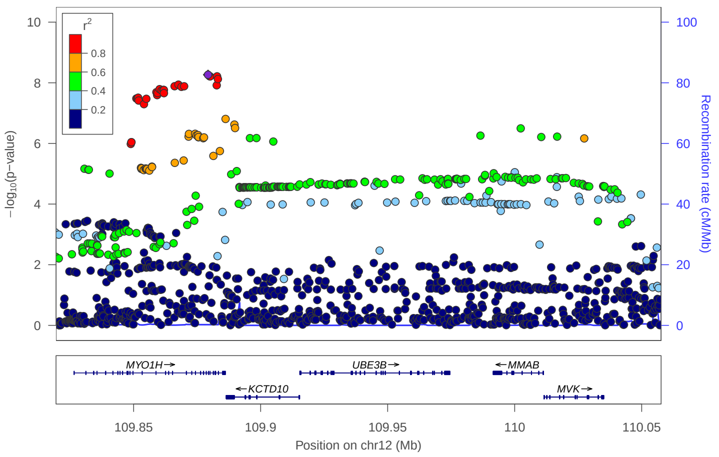

rs3936093

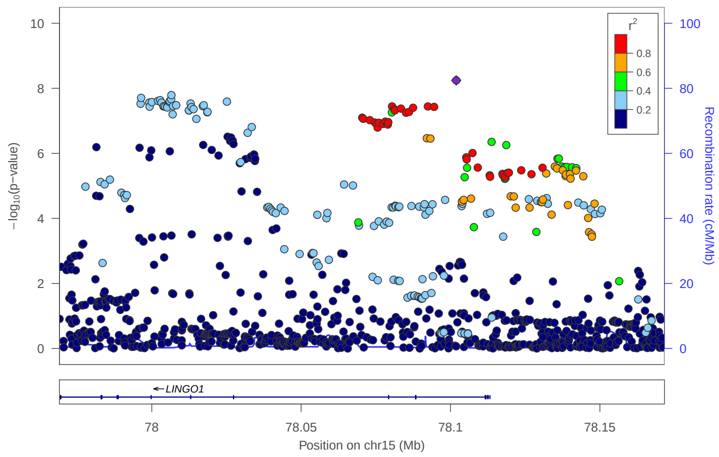

rs35994060

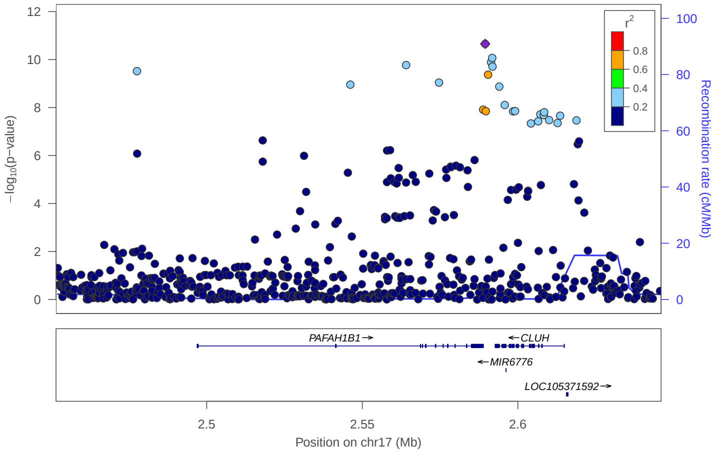

rs11650427

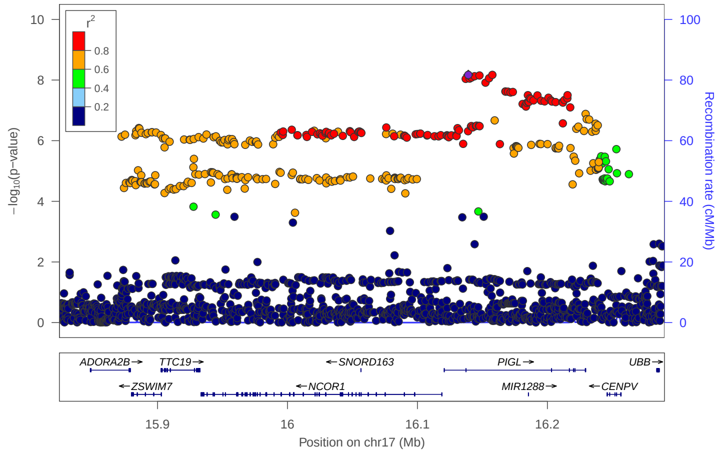

rs868150

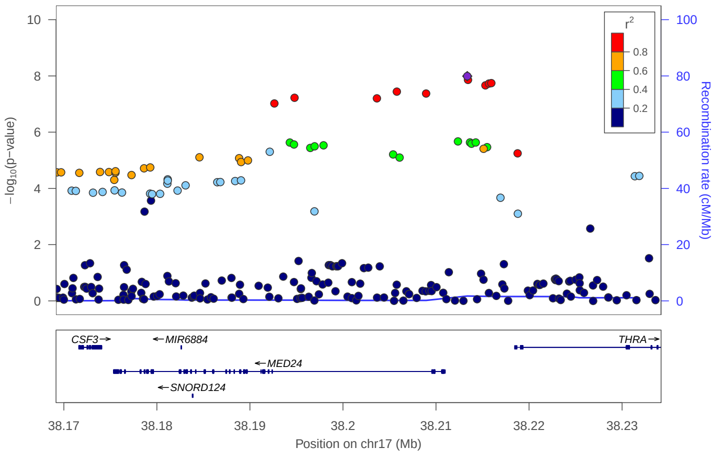

rs56280951

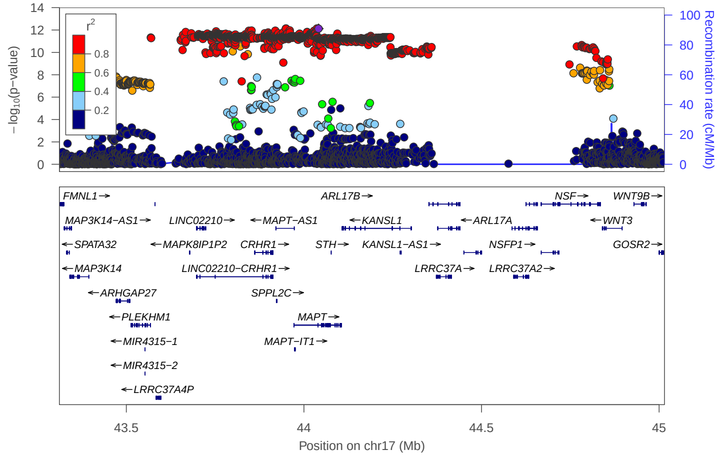

rs55731231

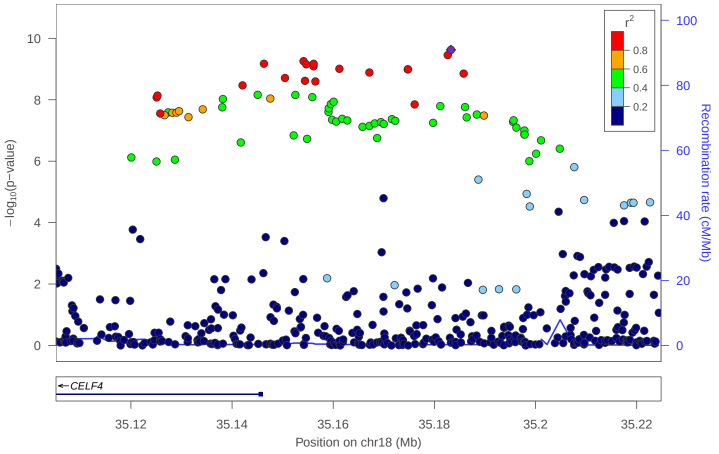

rs17681615

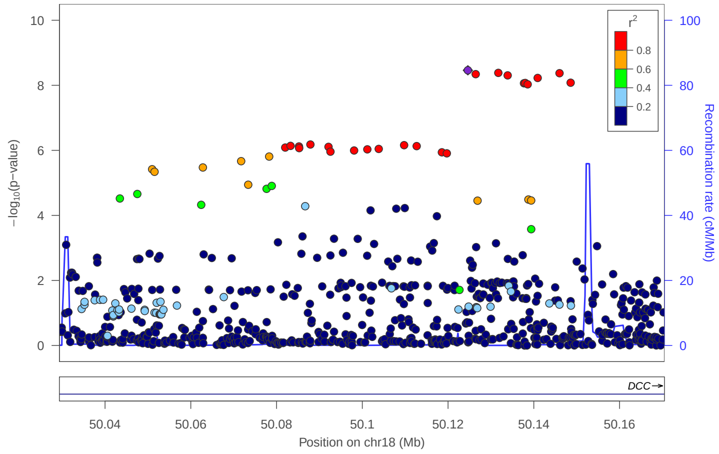

rs17501820

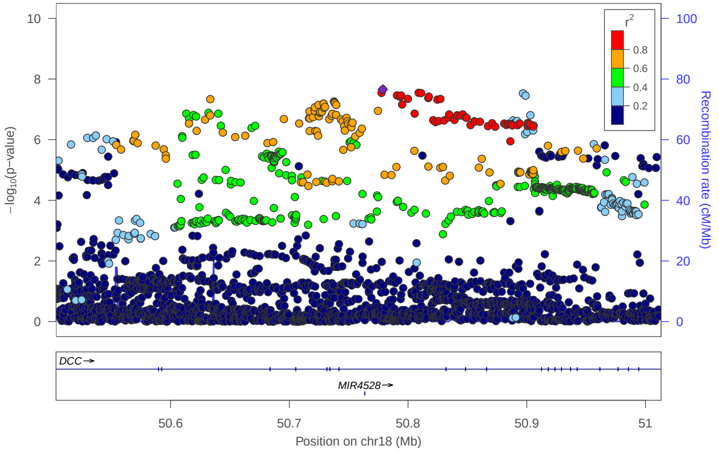

10
8
6
4
2
0
−log10(p−value)
100
80
60
40
20
0
Recombination rate (cM/Mb)
r2
0.8
0.6
0.4
0.2
DCC
MIR4528
50.6
50.7
50.8
50.9
51
Position on chr18 (Mb)

Supplementary Figure 7. Regional plots of GWA analyses of item MIS.

The *y*-axis represents the negative logarithm (base 10) of the SNP *P* value and the *x*-axis the position on the chromosome, with the name and location of genes in the UCSC Genome Browser in the bottom panel. The SNP with the lowest *P* value in the region is marked by a purple diamond. The colors of the other SNPs indicate the r^2 of these SNPs with the lead SNP, with grey color indicating SNPs below the user-defined r^2. Plots are generated with LocusZoom. See **Supplementary Data 2** for an overview of all lead SNPs for which a regional plot is presented above.

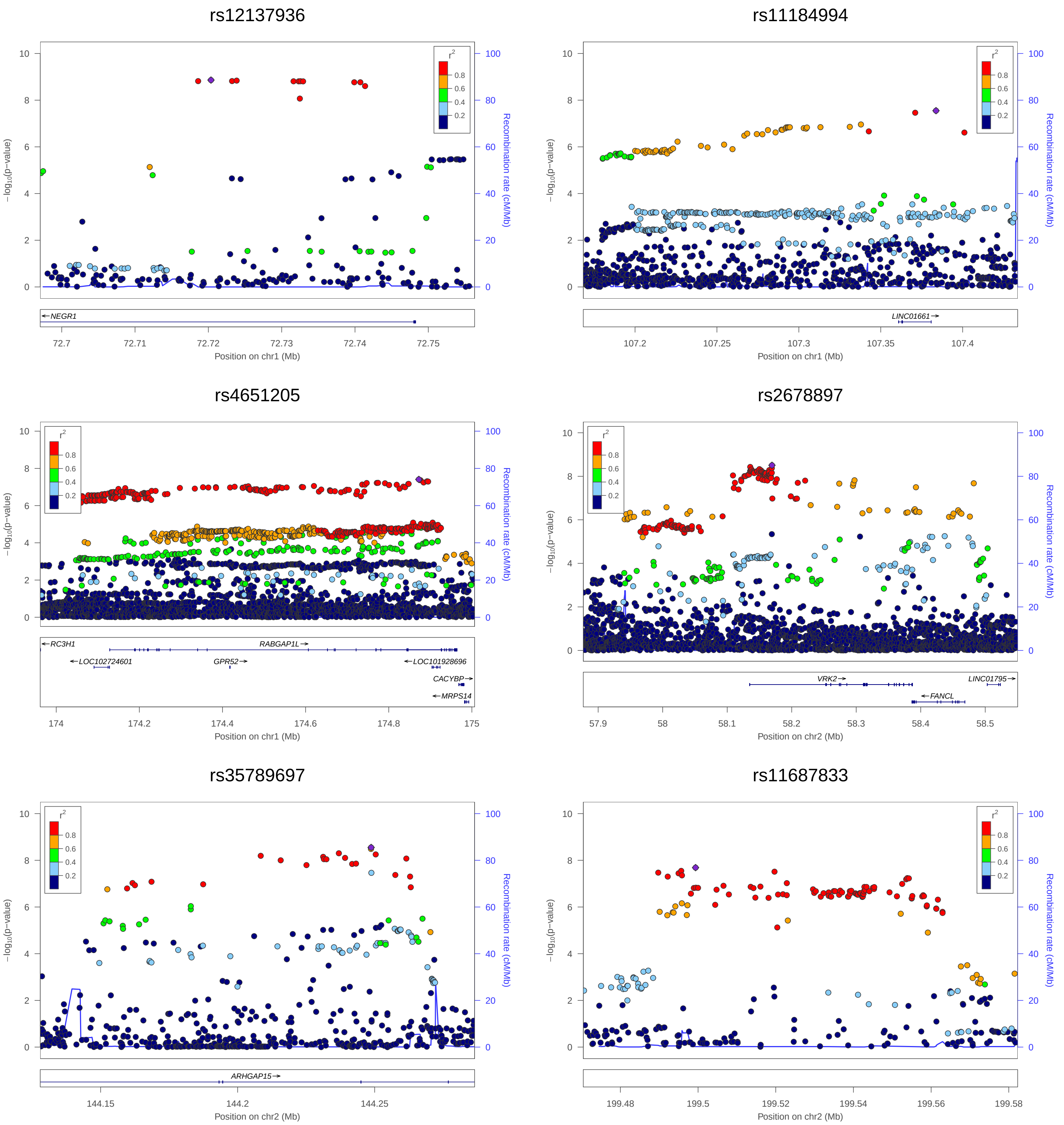
rs12137936
NEGR1
Position on chr1 (Mb)
rs11184994
LINC01661
Position on chr1 (Mb)
rs4651205
RC3H1
RABGAP1L
LOC102724601
GPR52
LOC101928696
CACYBP
MRPS14
Position on chr1 (Mb)
rs2678897
VRK2
LINC01795
FANCL
Position on chr2 (Mb)
rs35789697
ARHGAP15
Position on chr2 (Mb)
rs11687833
Position on chr2 (Mb)
−log10(p−value)
Recombination rate (cM/Mb)
r2

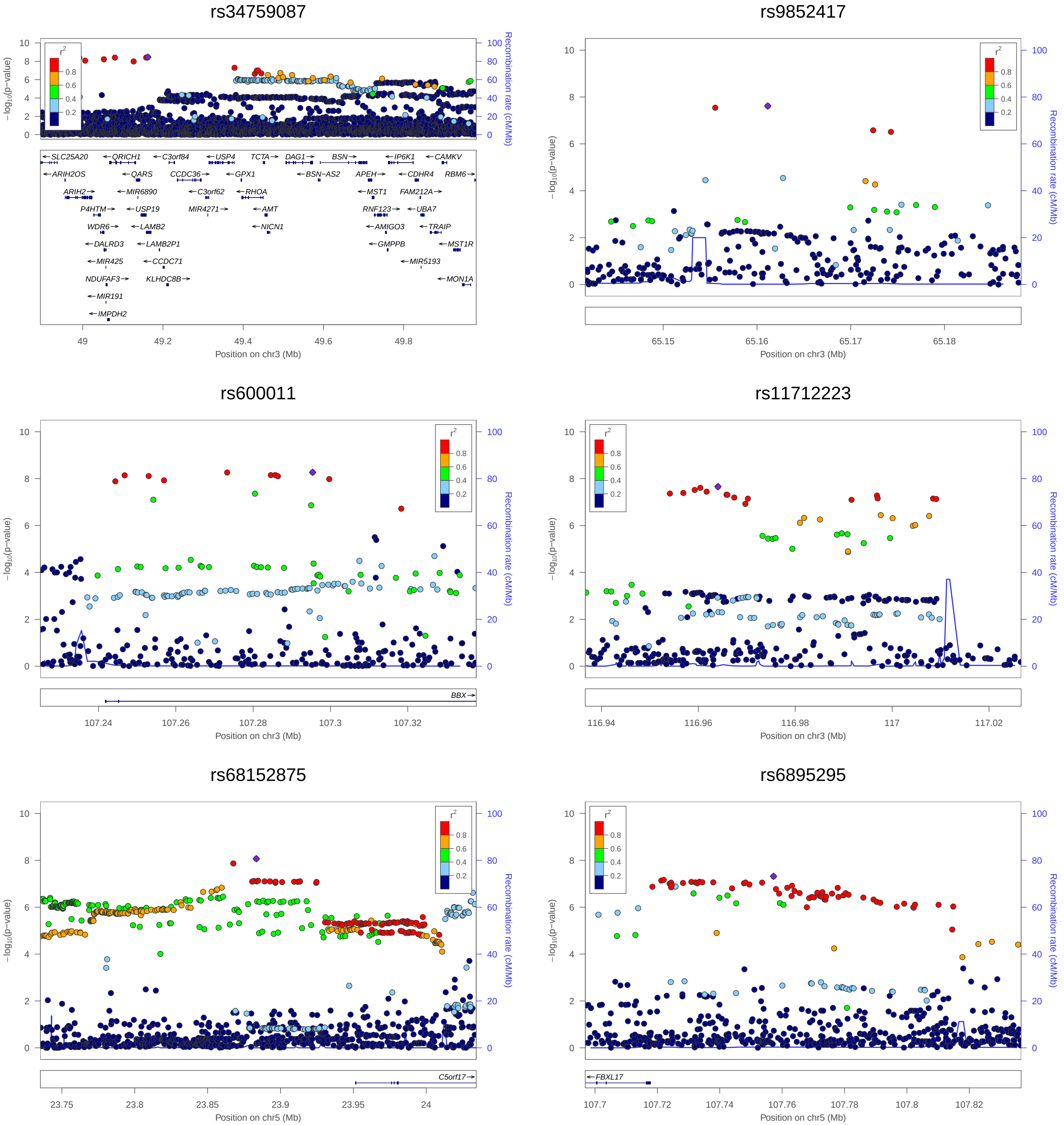

rs34759087
−log10(p−value)
Recombination rate (cM/Mb)
r²
SLC25A20
ARIH2OS
ARIH2
P4HTM
WDR6
DALRD3
MIR425
NDUFAF3
MIR191
IMPDH2
QRICH1
QARS
MIR6890
USP19
LAMB2
LAMB2P1
CCDC71
KLHDC8B
C3orf84
CCDC36
C3orf62
MIR4271
USP4
GPX1
RHOA
AMT
NICN1
TCTA
DAG1
BSN-AS2
BSN
APEH
MST1
RNF123
AMIGO3
GMPPB
IP6K1
CDHR4
FAM212A
UBA7
TRAIP
MIR5193
CAMKV
RBM6
MST1R
MON1A
Position on chr3 (Mb)
rs9852417
Position on chr3 (Mb)
rs600011
BBX
Position on chr3 (Mb)
rs11712223
Position on chr3 (Mb)
rs68152875
C5orf17
Position on chr5 (Mb)
rs6895295
FBXL17
Position on chr5 (Mb)

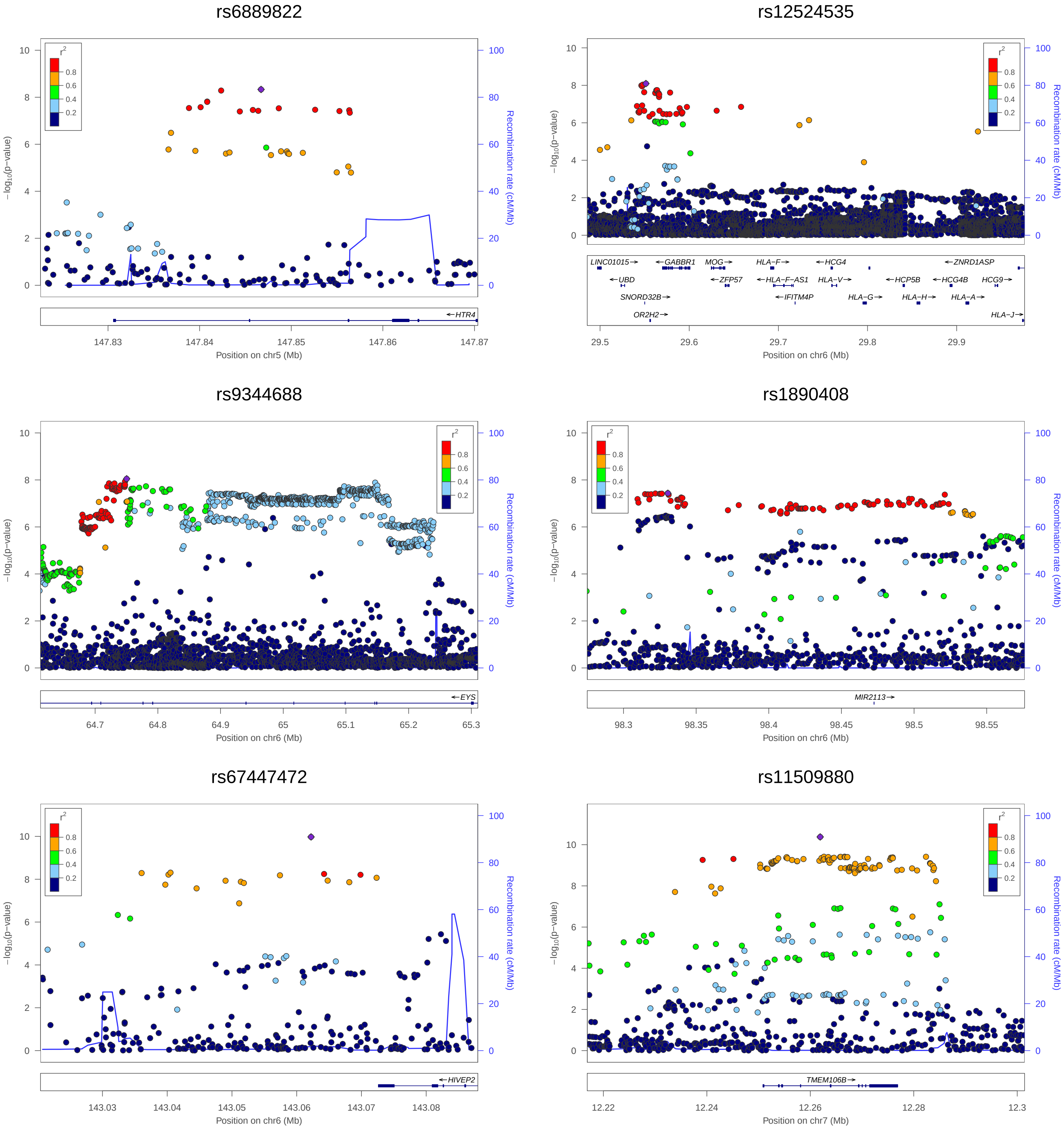
rs6889822
rs12524535
rs9344688
rs1890408
rs67447472
rs11509880
r2
0.8
0.6
0.4
0.2
−log10(p−value)
Recombination rate (cM/Mb)
HTR4
Position on chr5 (Mb)
147.83
147.84
147.85
147.86
147.87
LINC01015
GABBR1
MOG
HLA−F
HCG4
ZNRD1ASP
UBD
ZFP57
HLA−F−AS1
HLA−V
HCP5B
HCG4B
HCG9
SNORD32B
IFITM4P
HLA−G
HLA−H
HLA−A
OR2H2
HLA−J
Position on chr6 (Mb)
29.5
29.6
29.7
29.8
29.9
EYS
64.7
64.8
64.9
65
65.1
65.2
65.3
MIR2113
98.3
98.35
98.4
98.45
98.5
98.55
HIVEP2
143.03
143.04
143.05
143.06
143.07
143.08
TMEM106B
Position on chr7 (Mb)
12.22
12.24
12.26
12.28
12.3

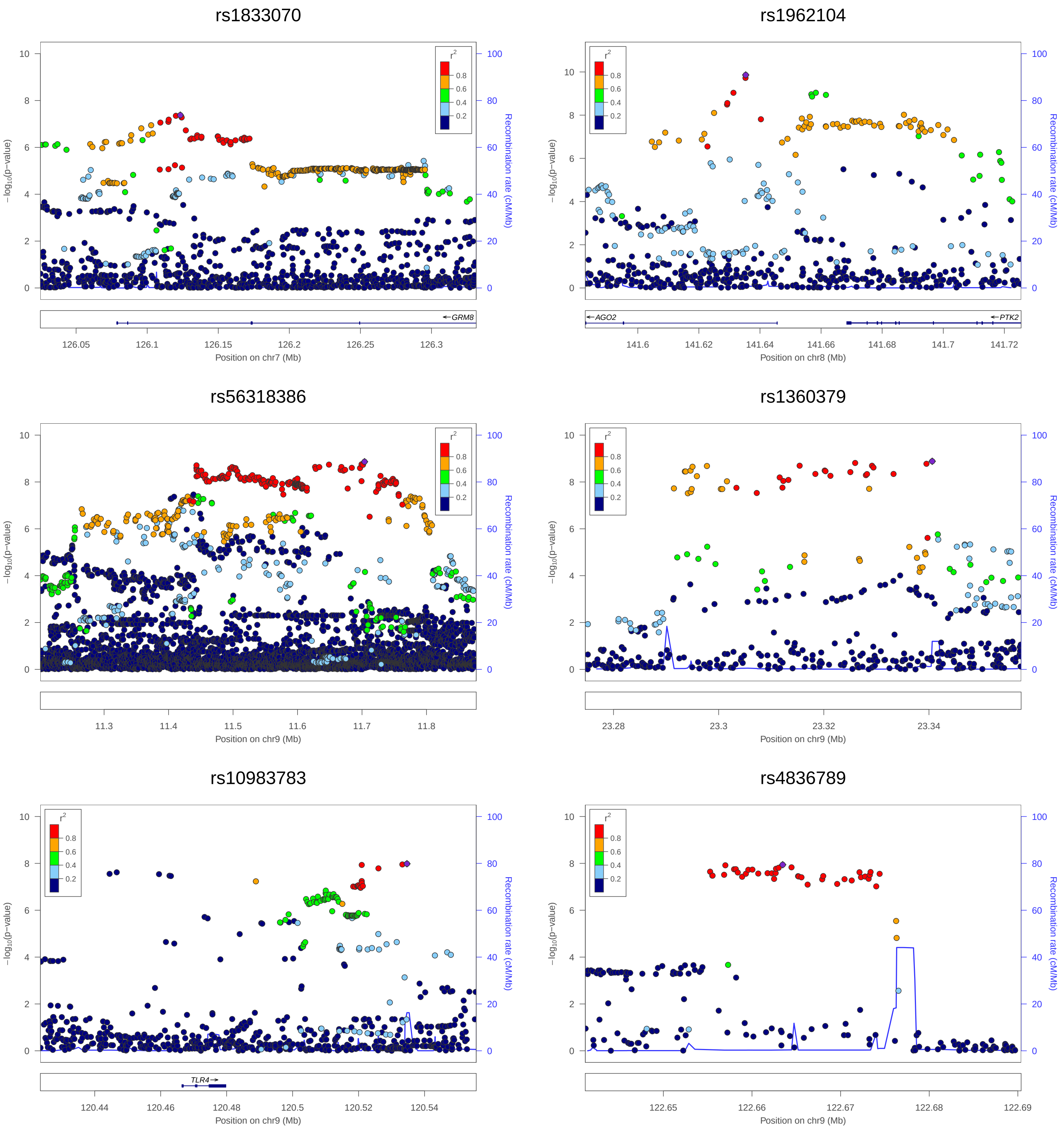
rs1833070
rs1962104
rs56318386
rs1360379
rs10983783
rs4836789
−log10(p−value)
Recombination rate (cM/Mb)
r2
0.8
0.6
0.4
0.2
GRM8
AGO2
PTK2
TLR4
Position on chr7 (Mb)
Position on chr8 (Mb)
Position on chr9 (Mb)

rs7895261

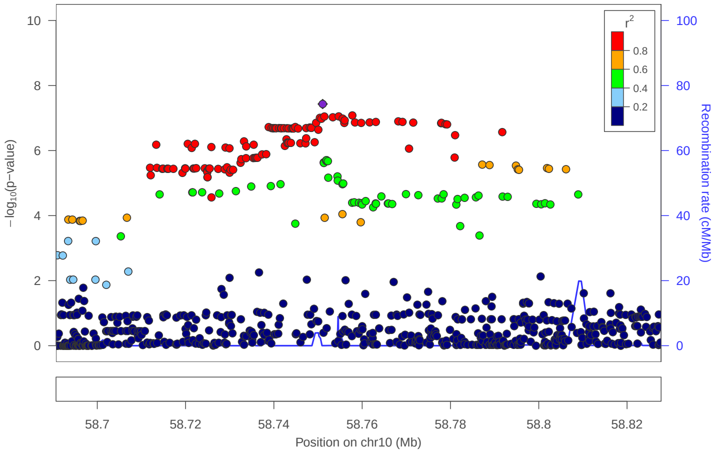

rs297346

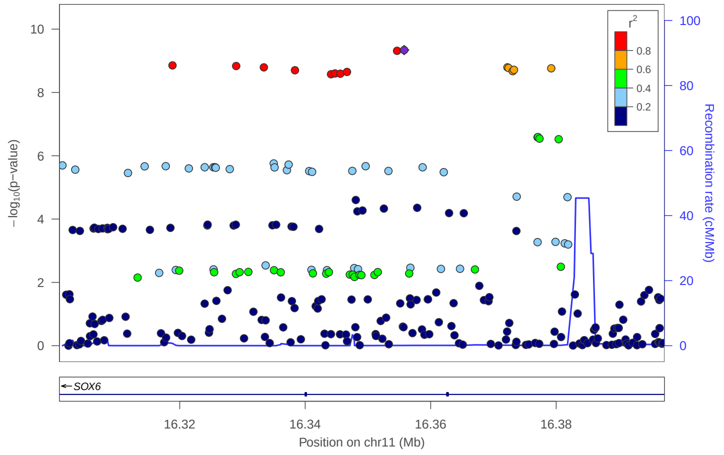

rs11039149

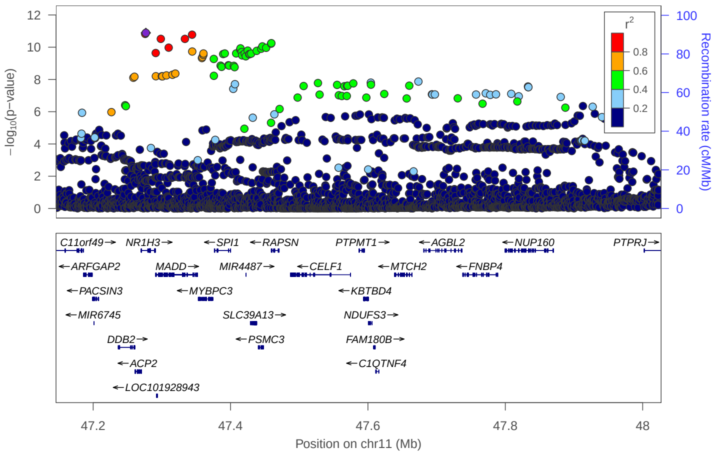

rs12420205

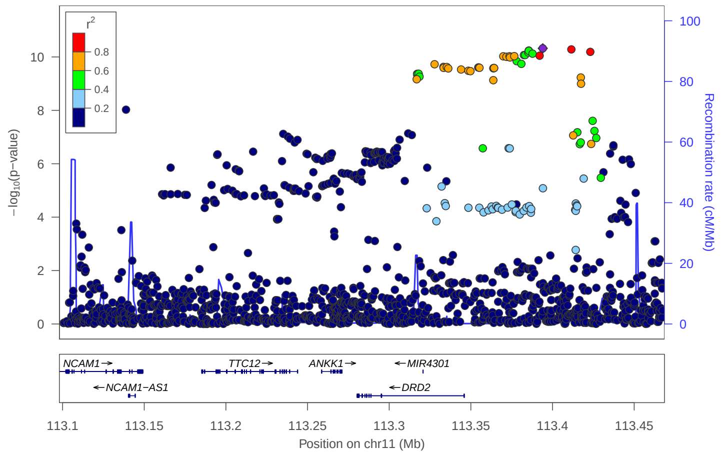

rs28655666

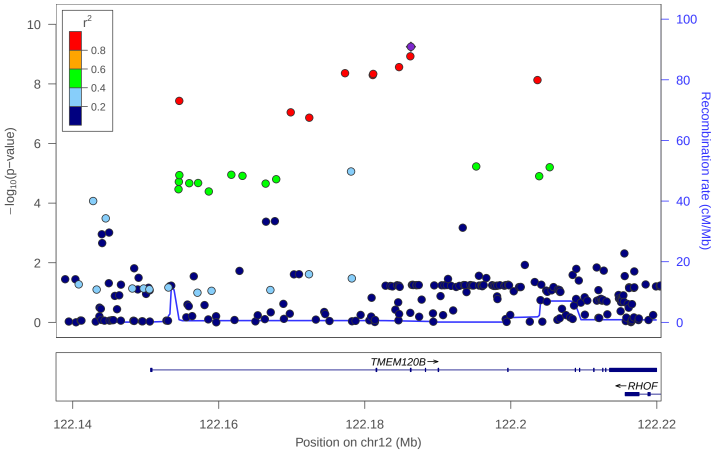

rs1373921

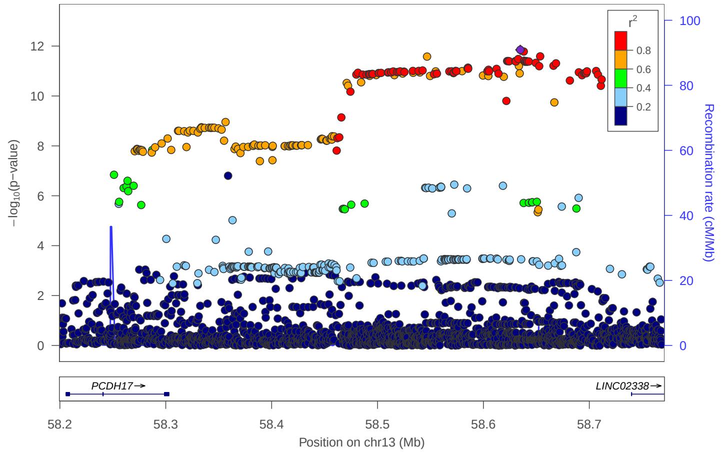

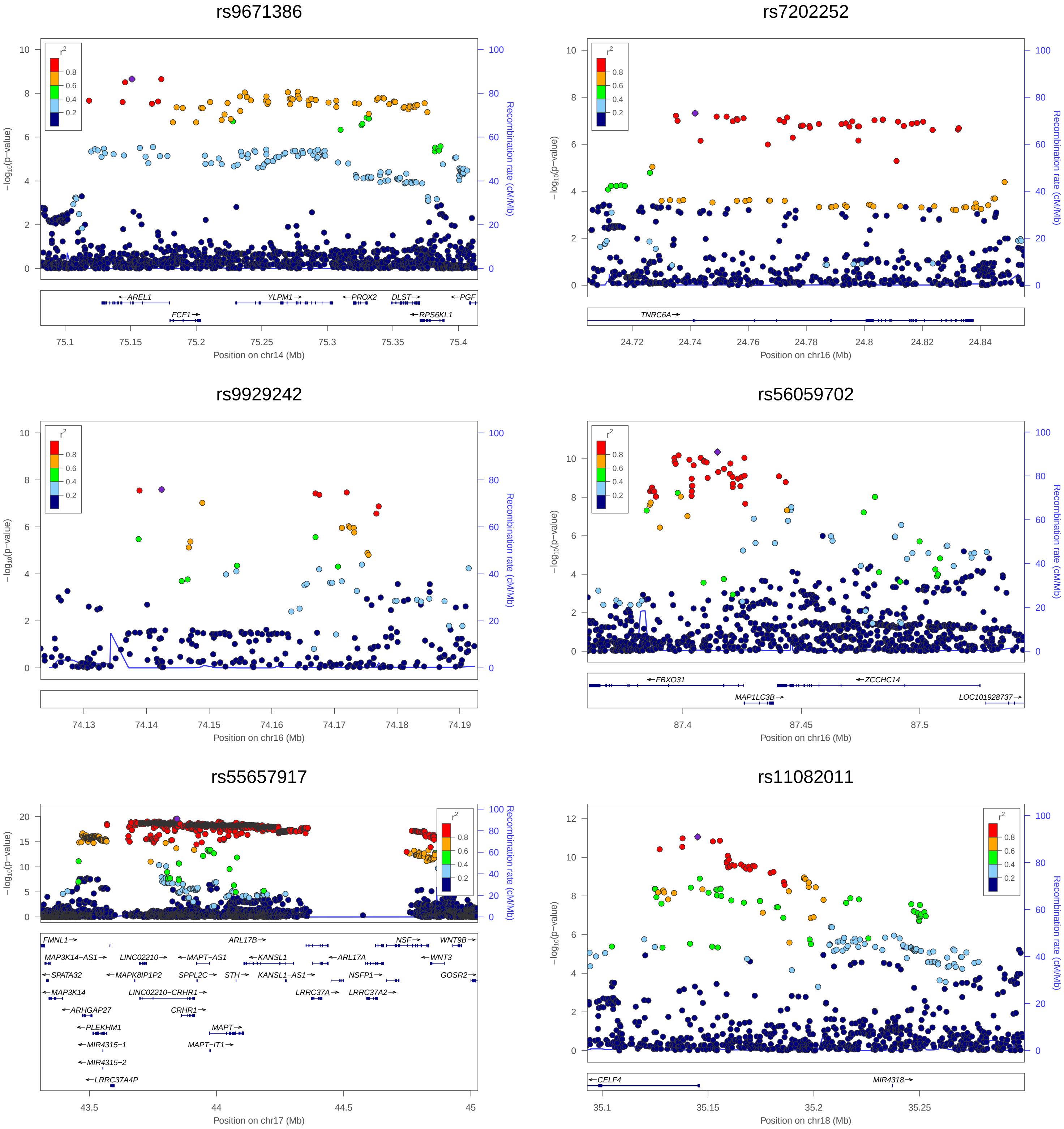
rs9671386
−log10(p−value)
Recombination rate (cM/Mb)
r2
AREL1
YLPM1
PROX2
DLST
PGF
FCF1
RPS6KL1
Position on chr14 (Mb)
rs7202252
TNRC6A
Position on chr16 (Mb)
rs9929242
Position on chr16 (Mb)
rs56059702
FBXO31
ZCCHC14
MAP1LC3B
LOC101928737
Position on chr16 (Mb)
rs55657917
FMNL1
ARL17B
NSF
WNT9B
MAP3K14−AS1
LINC02210
MAPT−AS1
KANSL1
ARL17A
WNT3
SPATA32
MAPK8IP1P2
SPPL2C
STH
KANSL1−AS1
NSFP1
GOSR2
MAP3K14
LINC02210−CRHR1
LRRC37A
LRRC37A2
ARHGAP27
CRHR1
PLEKHM1
MAPT
MIR4315−1
MAPT−IT1
MIR4315−2
LRRC37A4P
Position on chr17 (Mb)
rs11082011
CELF4
MIR4318
Position on chr18 (Mb)

rs11877496

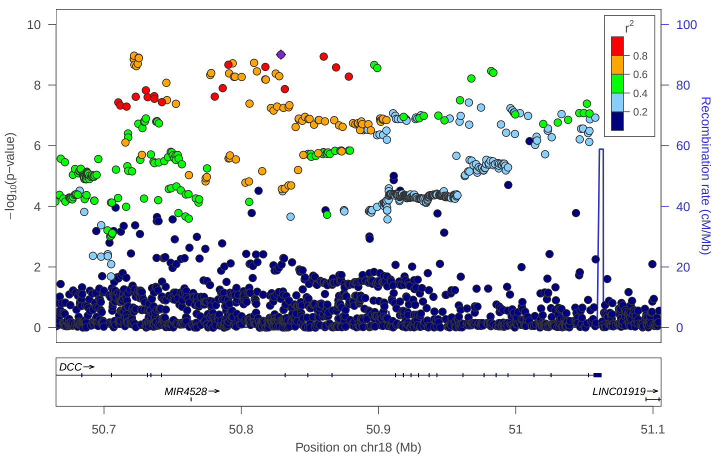

rs56403421

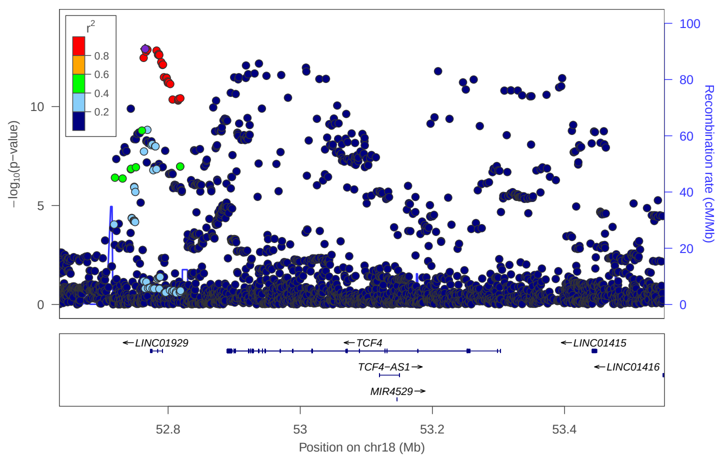

rs6103271

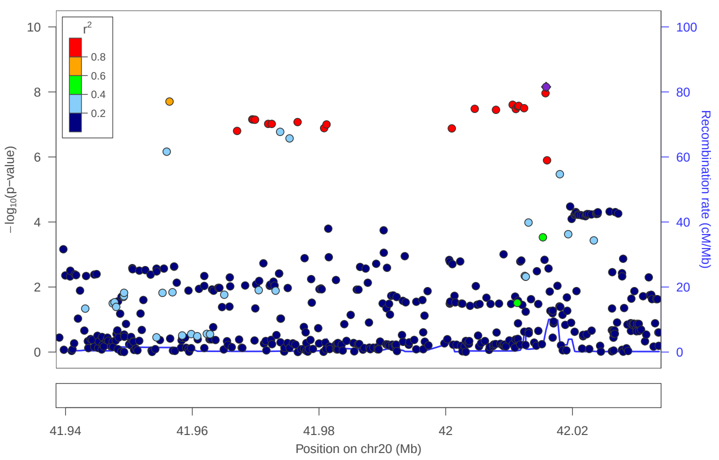

rs4578918

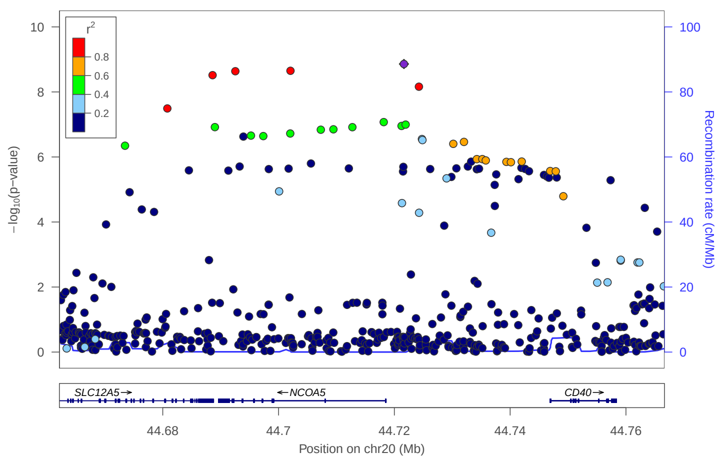

rs11090039

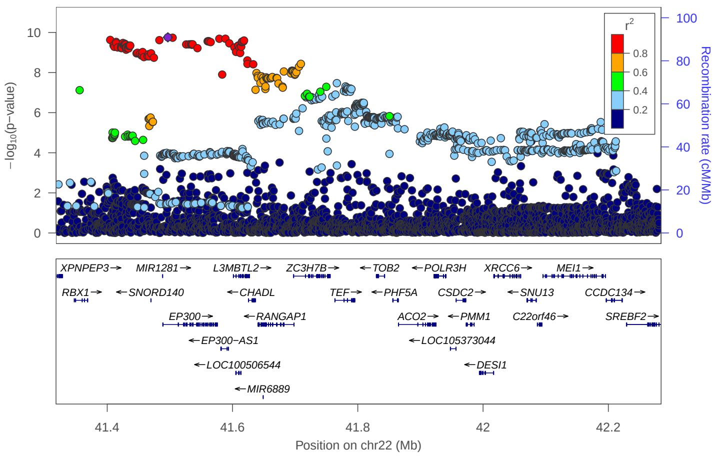

Supplementary Figure 8. Regional plots of GWA analyses of item MOOD.

The *y*-axis represents the negative logarithm (base 10) of the SNP *P* value and the *x*-axis the position on the chromosome, with the name and location of genes in the UCSC Genome Browser in the bottom panel. The SNP with the lowest *P* value in the region is marked by a purple diamond. The colors of the other SNPs indicate the r^2 of these SNPs with the lead SNP, with grey color indicating SNPs below the user-defined r^2. Plots are generated with LocusZoom. See **Supplementary Data 2** for an overview of all lead SNPs for which a regional plot is presented above.

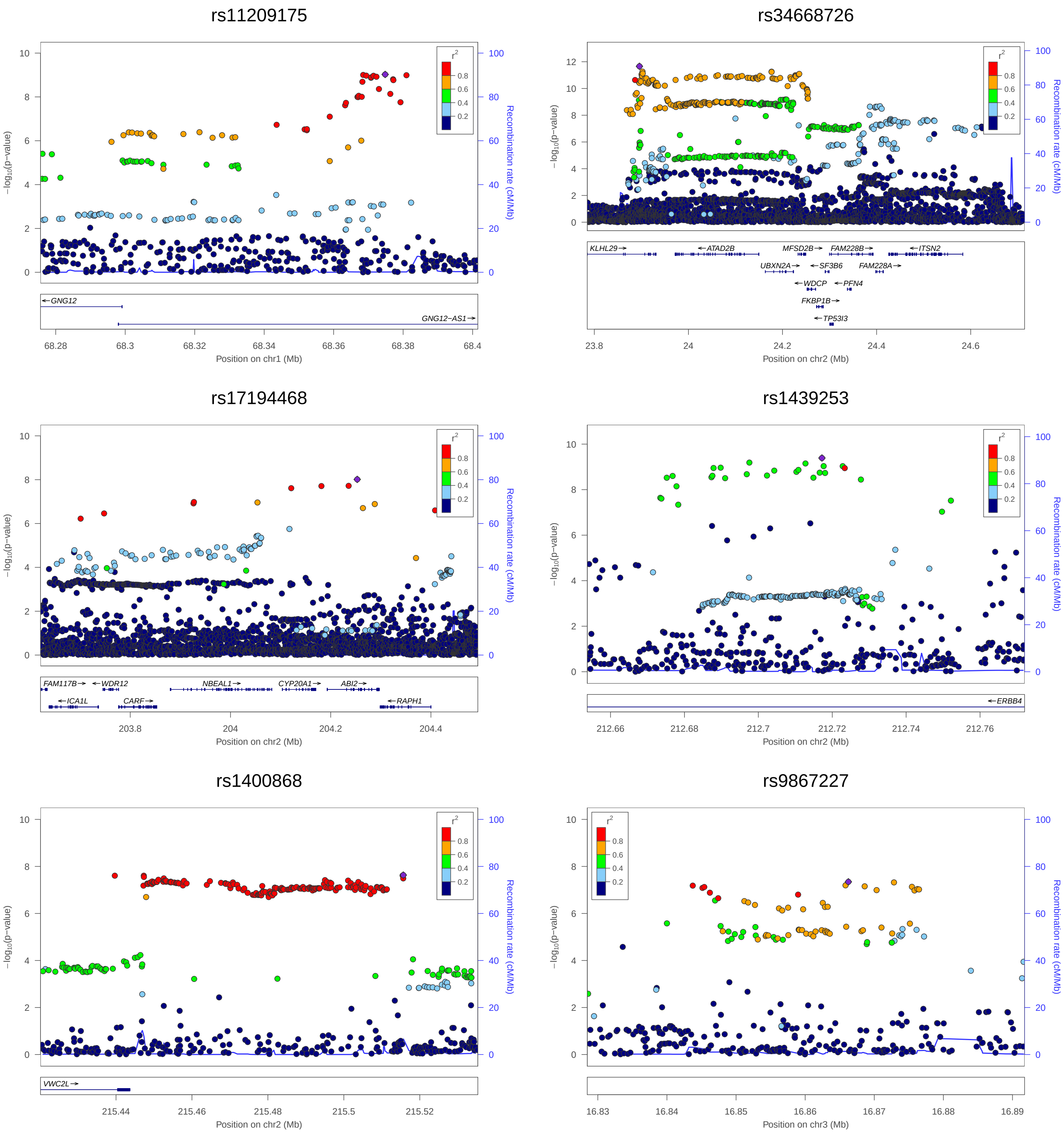
rs11209175
−log10(p−value)
Recombination rate (cM/Mb)
GNG12
GNG12−AS1
Position on chr1 (Mb)
rs34668726
KLHL29
ATAD2B
MFSD2B
FAM228B
ITSN2
UBXN2A
SF3B6
FAM228A
WDCP
PFN4
FKBP1B
TP53I3
Position on chr2 (Mb)
rs17194468
FAM117B
WDR12
NBEAL1
CYP20A1
ABI2
ICA1L
CARF
RAPH1
Position on chr2 (Mb)
rs1439253
ERBB4
Position on chr2 (Mb)
rs1400868
VWC2L
Position on chr2 (Mb)
rs9867227
Position on chr3 (Mb)

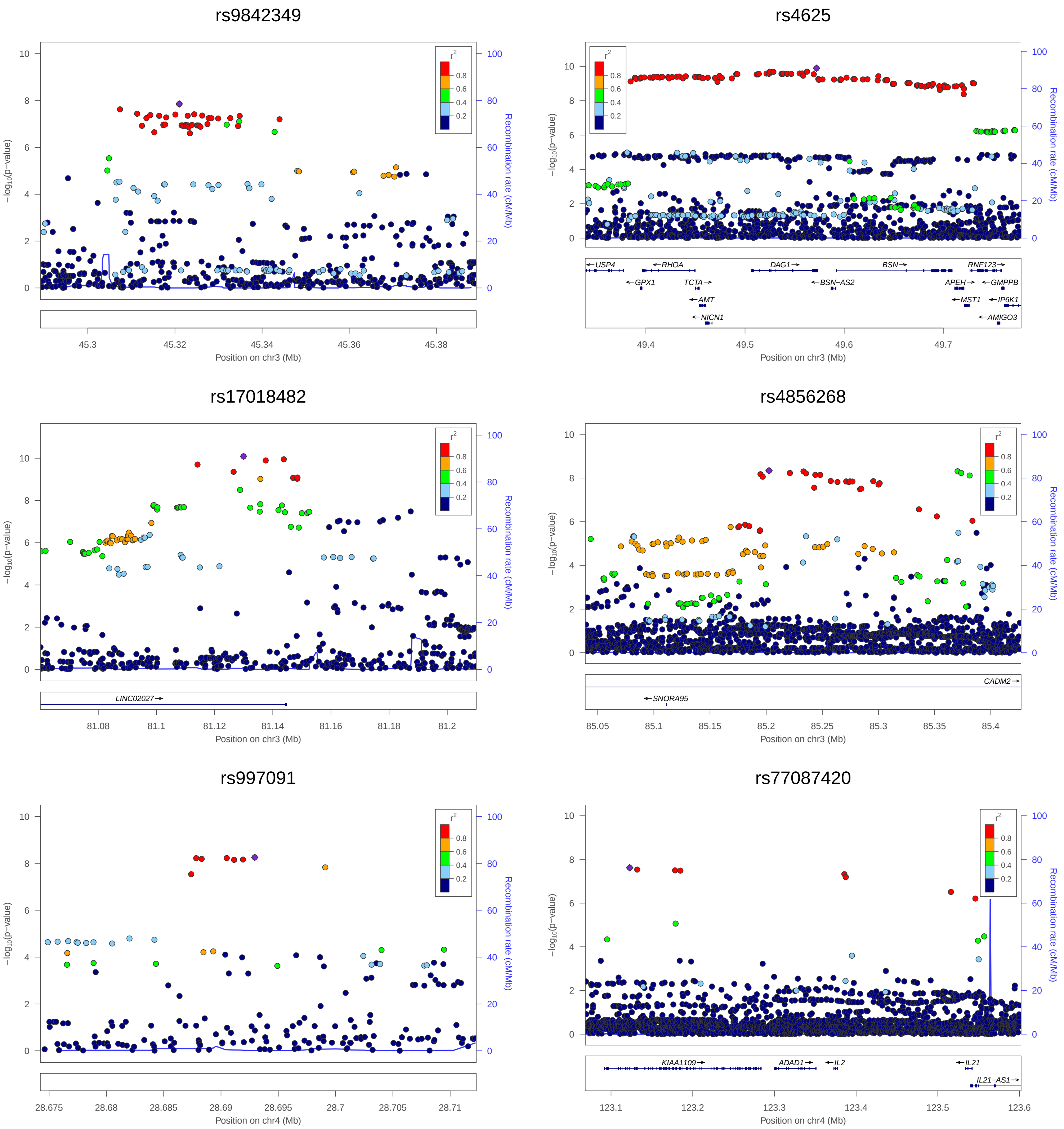
rs9842349
−log10(p−value)
Recombination rate (cM/Mb)
Position on chr3 (Mb)
45.3
45.32
45.34
45.36
45.38
rs4625
USP4
RHOA
GPX1
TCTA
AMT
NICN1
DAG1
BSN−AS2
BSN
RNF123
APEH
GMPPB
MST1
IP6K1
AMIGO3
Position on chr3 (Mb)
49.4
49.5
49.6
49.7
rs17018482
LINC02027
Position on chr3 (Mb)
81.08
81.1
81.12
81.14
81.16
81.18
81.2
rs4856268
CADM2
SNORA95
Position on chr3 (Mb)
85.05
85.1
85.15
85.2
85.25
85.3
85.35
85.4
rs997091
Position on chr4 (Mb)
28.675
28.68
28.685
28.69
28.695
28.7
28.705
28.71
rs77087420
KIAA1109
ADAD1
IL2
IL21
IL21−AS1
Position on chr4 (Mb)
123.1
123.2
123.3
123.4
123.5
123.6
r2
0.8
0.6
0.4
0.2

rs6862766

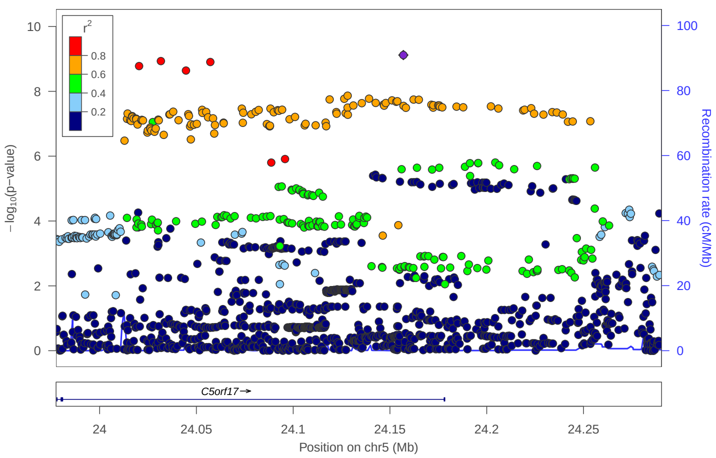

rs10044479

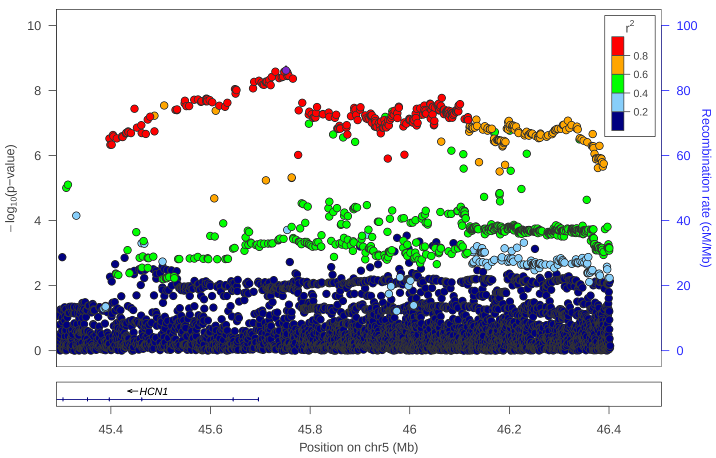

rs143668283

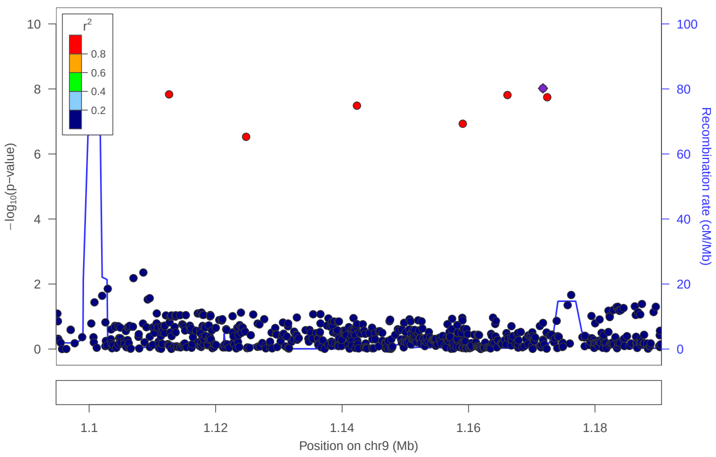

rs2149351

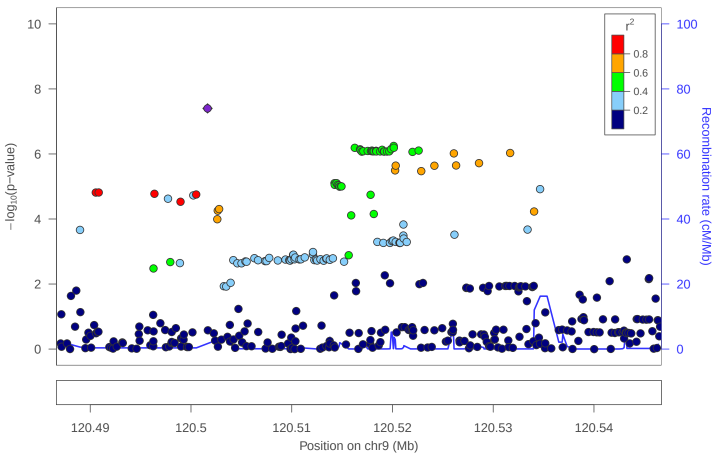

rs7912226

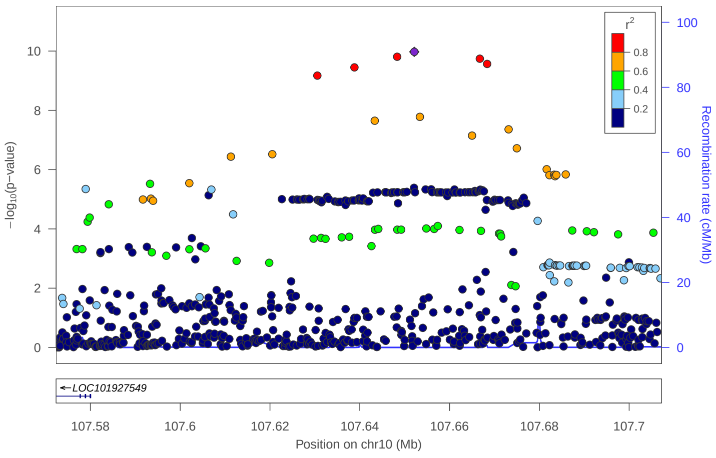

rs10838635

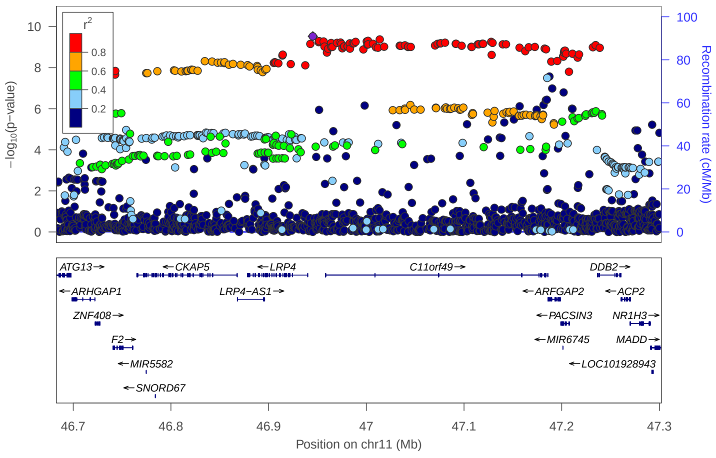

rs11532157

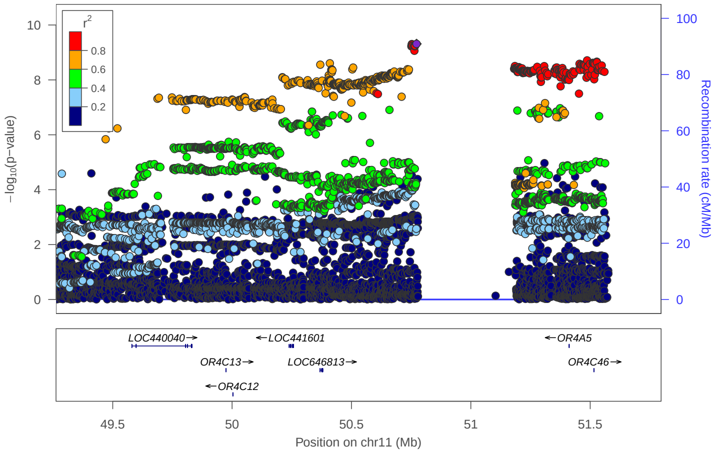

rs2262536

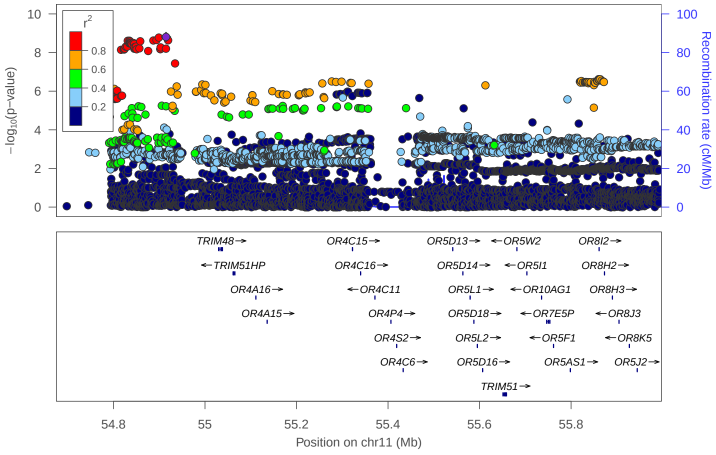

rs7334060

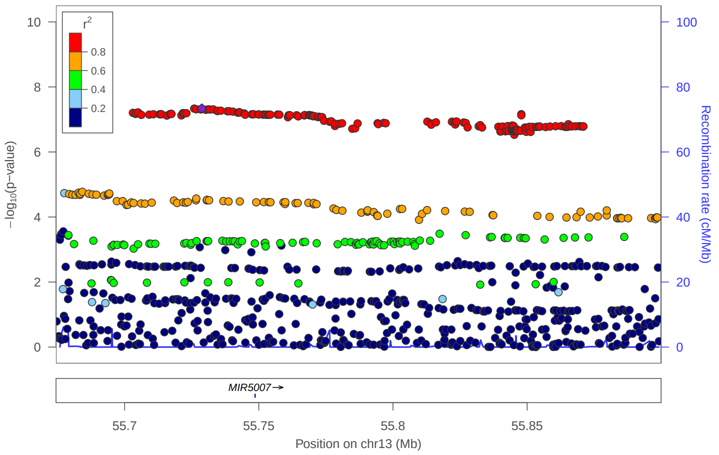

rs1275411

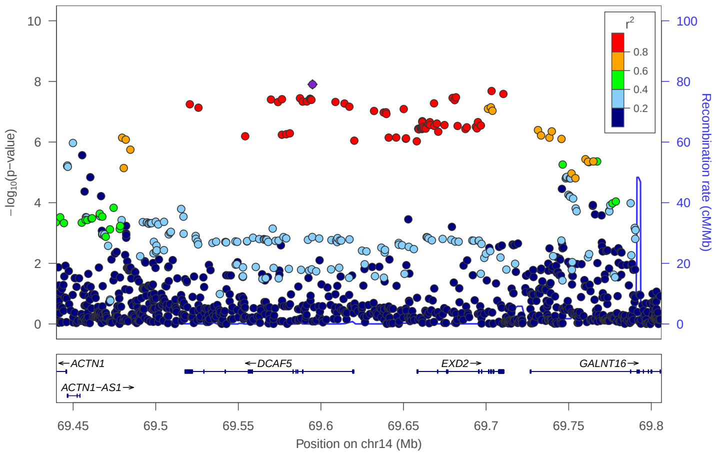

rs10144845

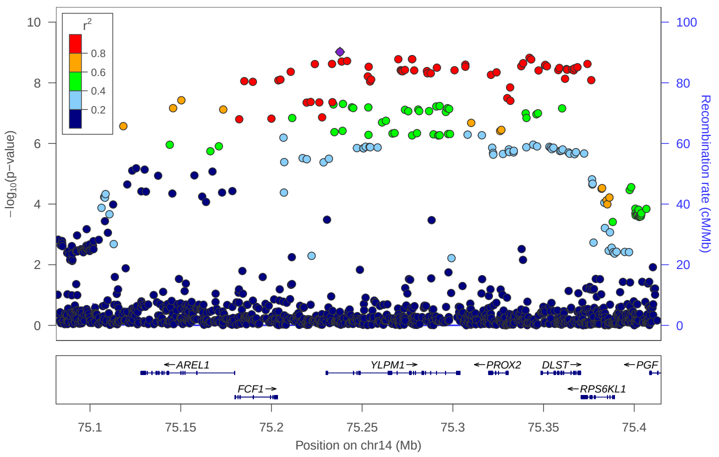

rs34641928

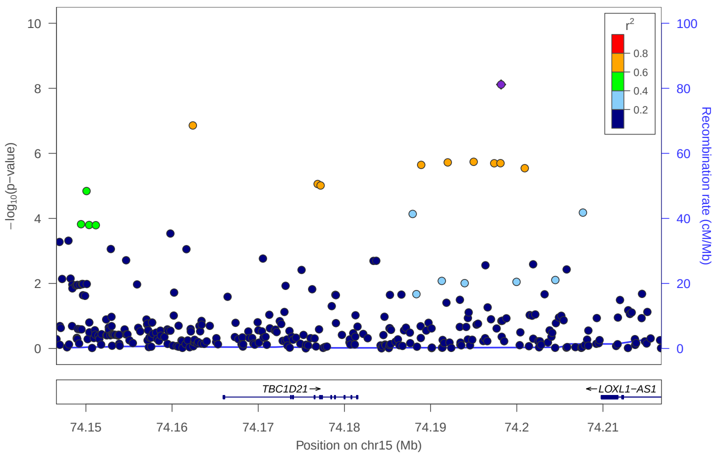

rs783537

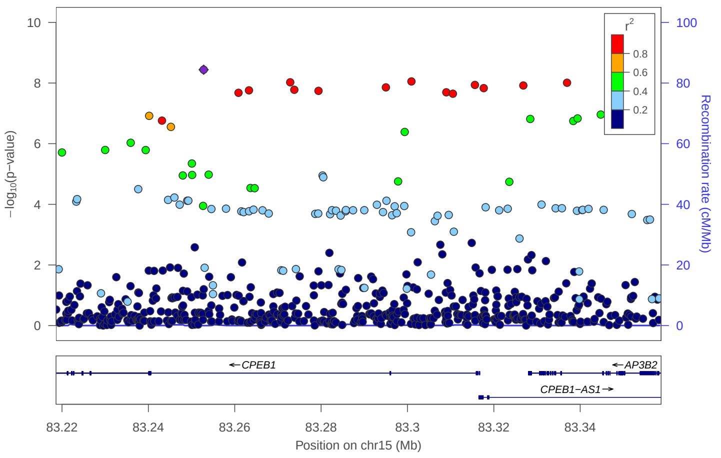

rs61744010

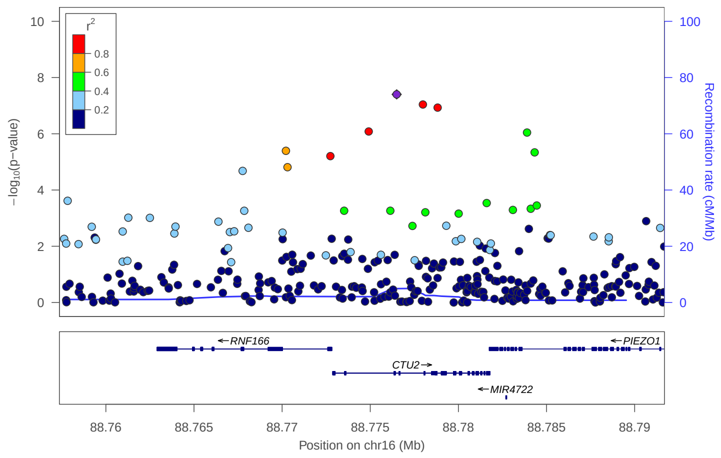

rs4630591

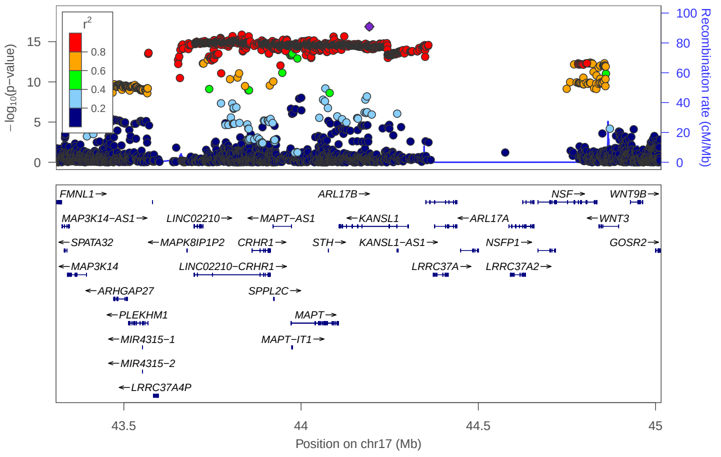

rs4799949

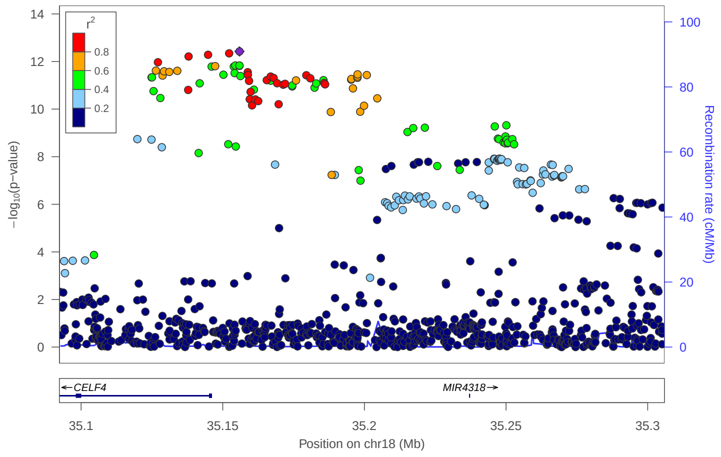

rs1875560

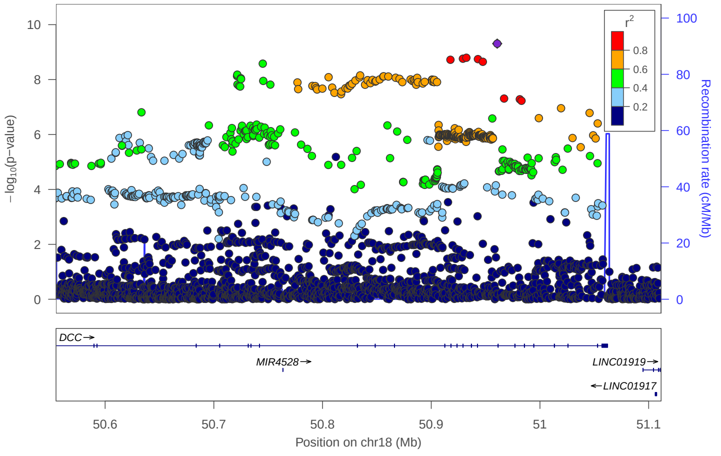

rs599550

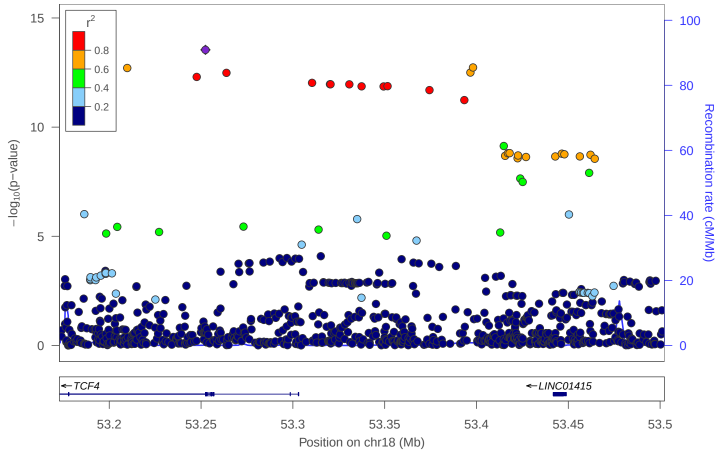

rs10409264

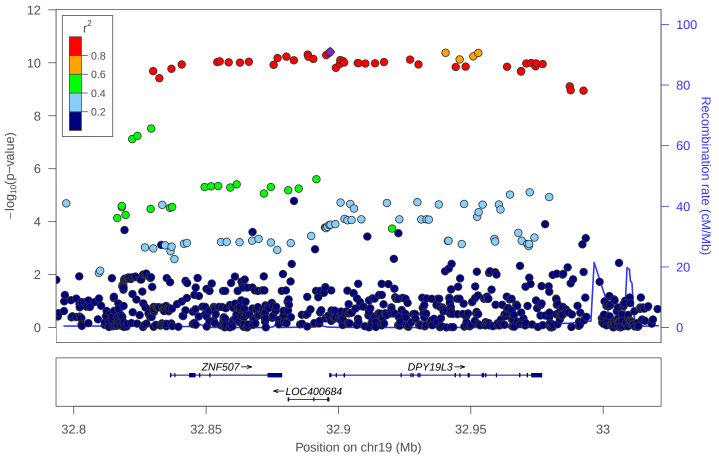

rs6130328

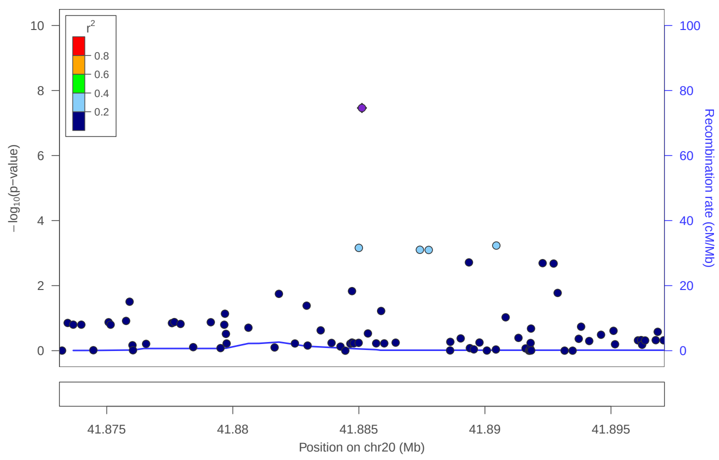

rs6031966

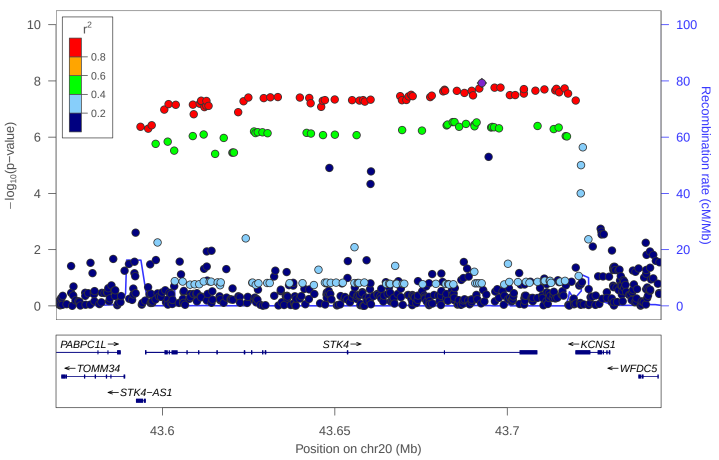

Supplementary Figure 9. Regional plots of GWA analyses of item FED-UP.

The *y*-axis represents the negative logarithm (base 10) of the SNP *P* value and the *x*-axis the position on the chromosome, with the name and location of genes in the UCSC Genome Browser in the bottom panel. The SNP with the lowest *P* value in the region is marked by a purple diamond. The colors of the other SNPs indicate the r^2 of these SNPs with the lead SNP, with grey color indicating SNPs below the user-defined r^2. Plots are generated with LocusZoom. See **Supplementary Data 2** for an overview of all lead SNPs for which a regional plot is presented above. For excluded loci (see **Methods**; **Supplementary Data 3-4**) the rsID's are marked yellow.

rs332828

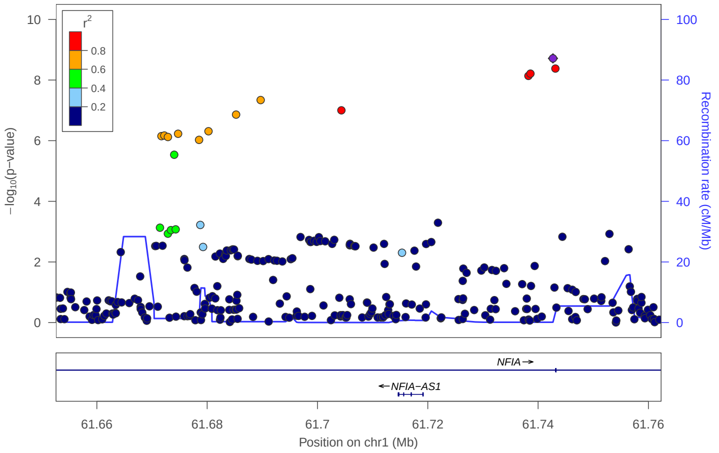

rs10747488

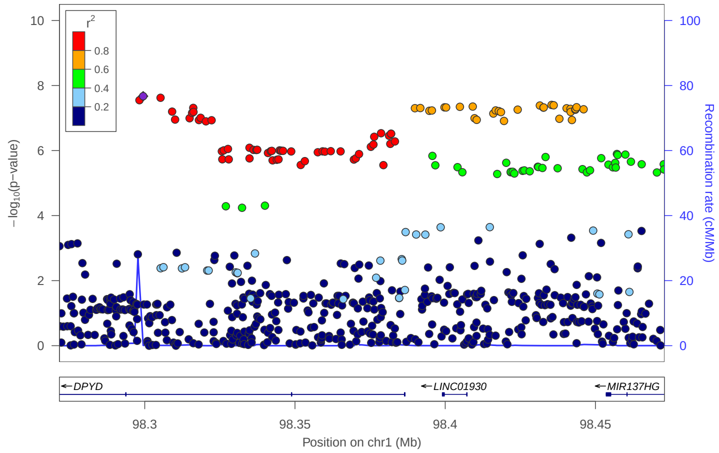

rs12729445

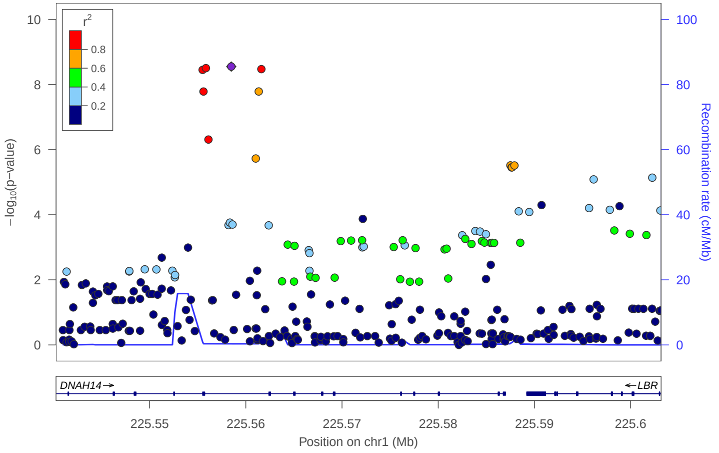

rs1368549

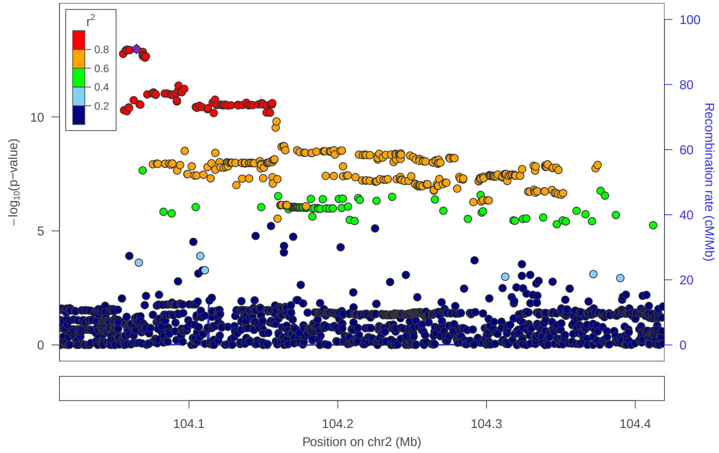

rs13395141

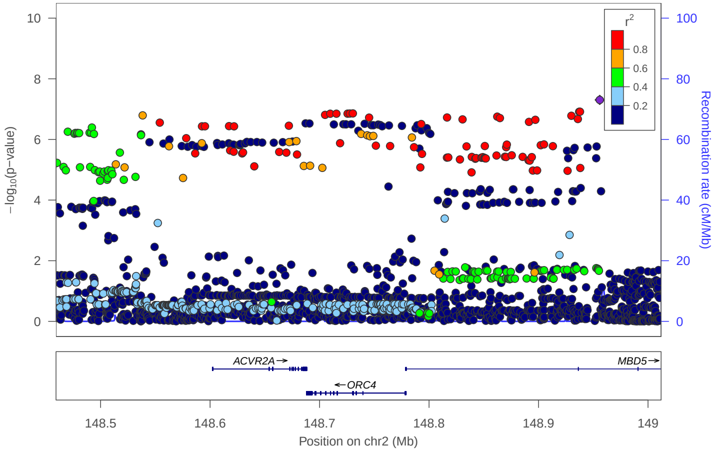

rs9879090

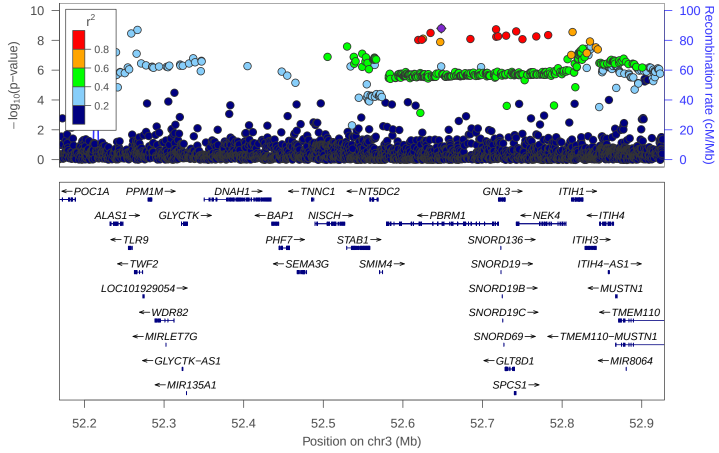

rs1375545

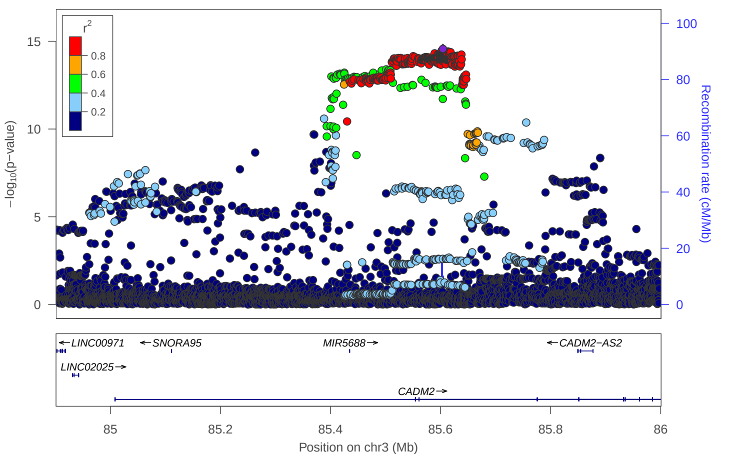

rs10935184

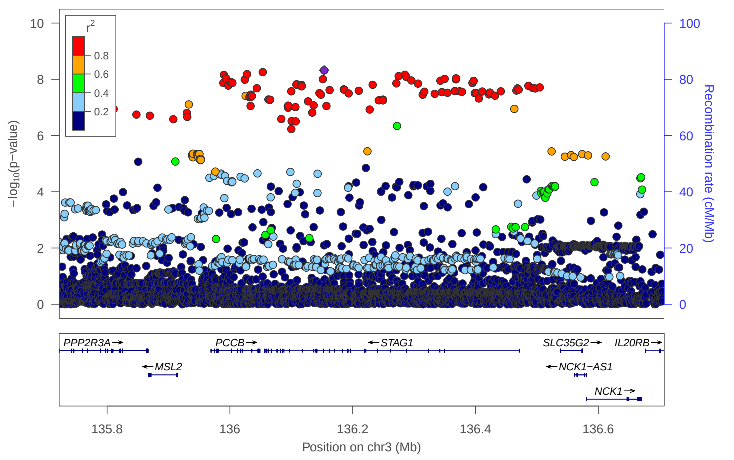

rs974711

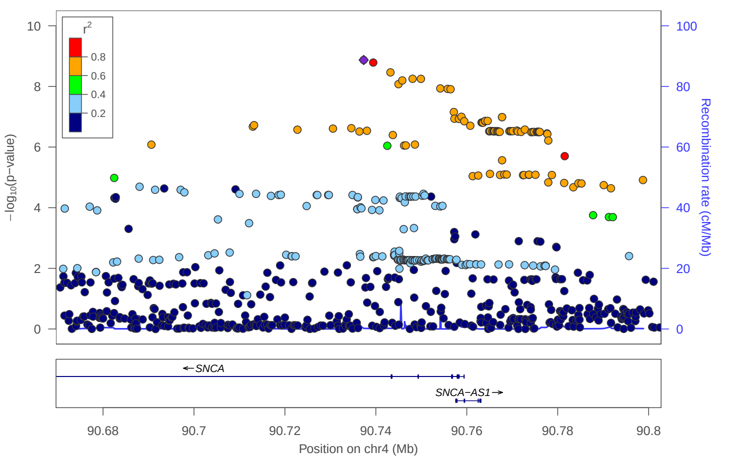

rs12203592

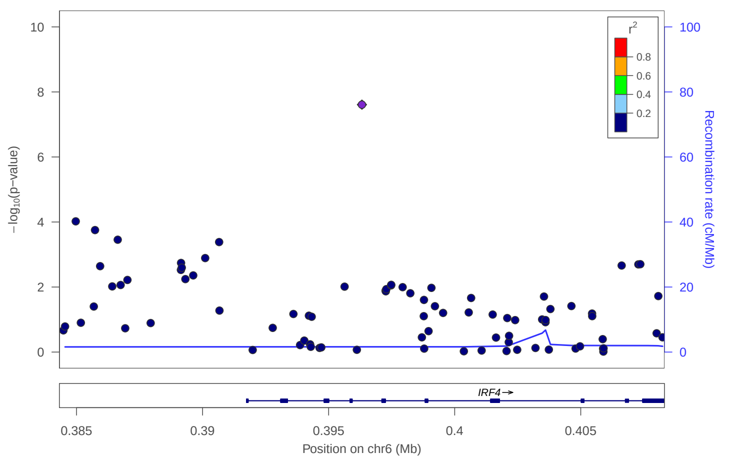

rs3117425

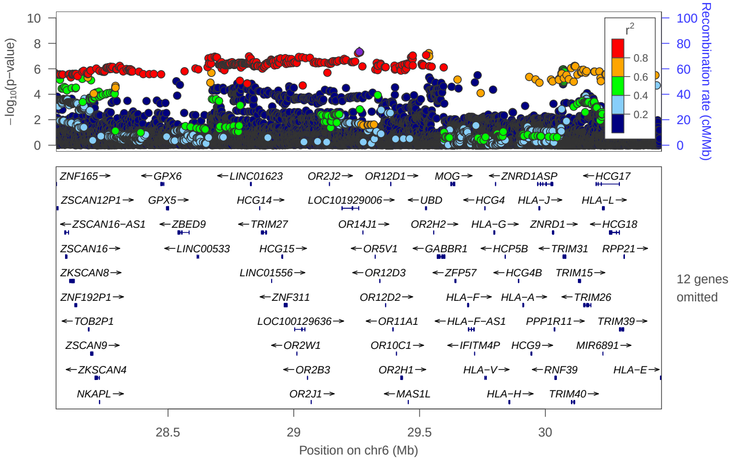

rs707916

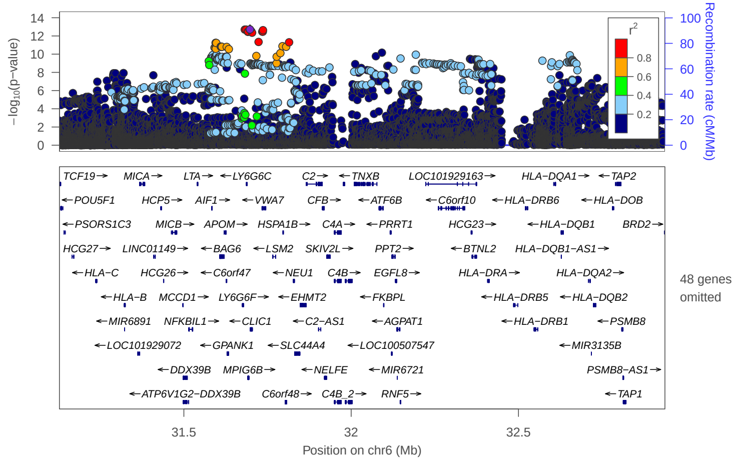

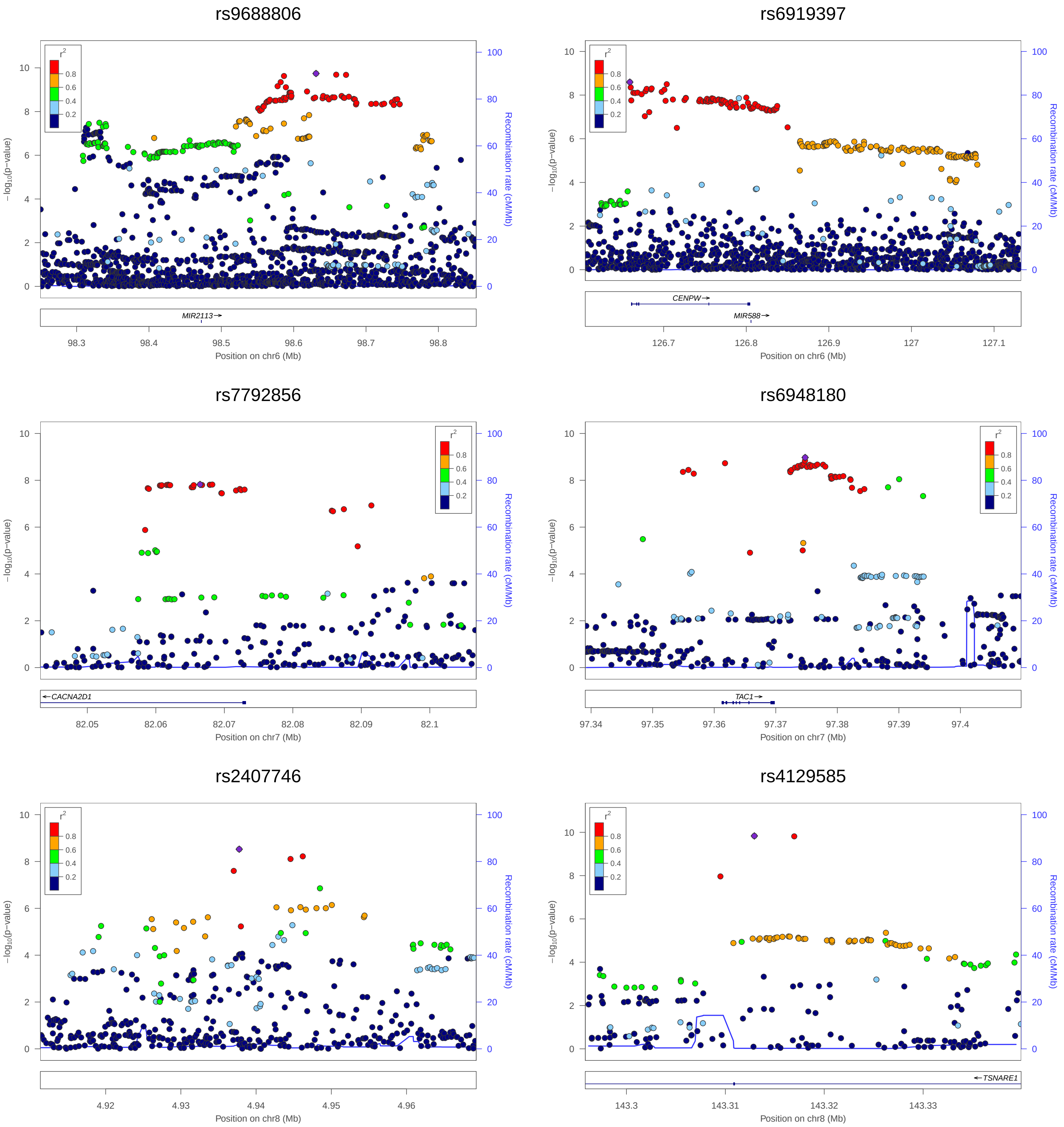
rs9688806
MIR2113
Position on chr6 (Mb)
rs6919397
CENPW
MIR588
Position on chr6 (Mb)
rs7792856
CACNA2D1
Position on chr7 (Mb)
rs6948180
TAC1
Position on chr7 (Mb)
rs2407746
Position on chr8 (Mb)
rs4129585
TSNARE1
Position on chr8 (Mb)
−log10(p−value)
Recombination rate (cM/Mb)
r2
0.8
0.6
0.4
0.2

rs7026674

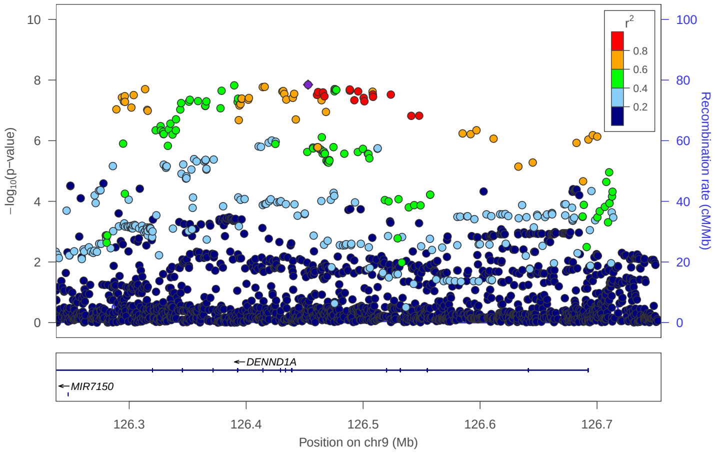

rs4919695

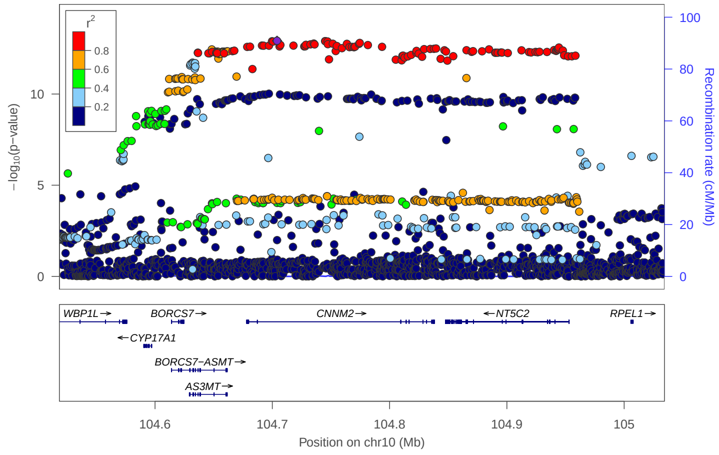

rs2930456

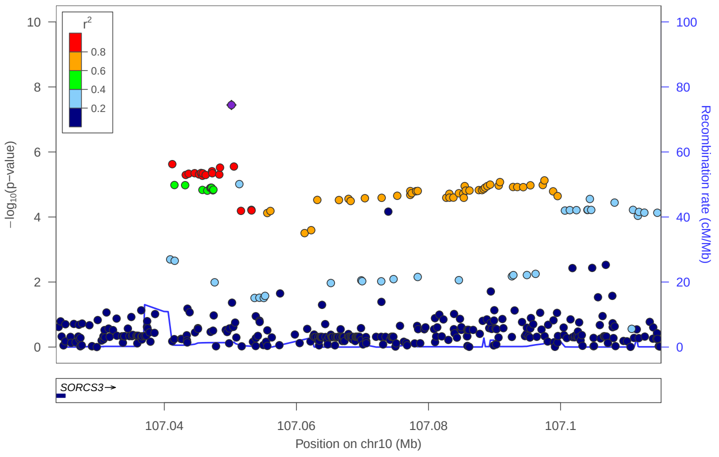

rs11030107

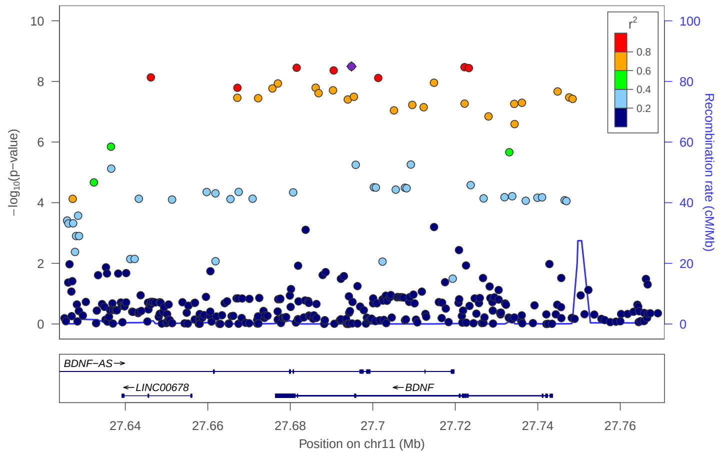

rs10501320

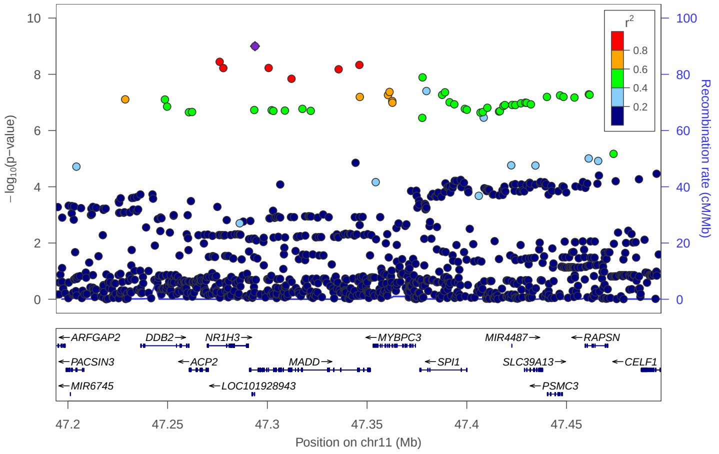

rs11619066

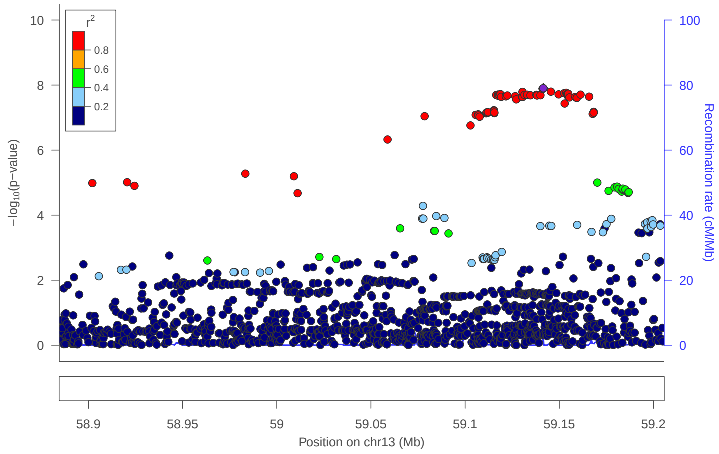

rs9571771

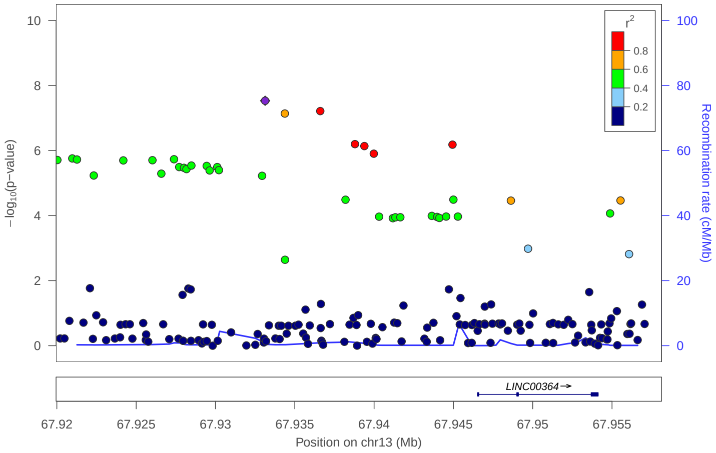

rs2853779

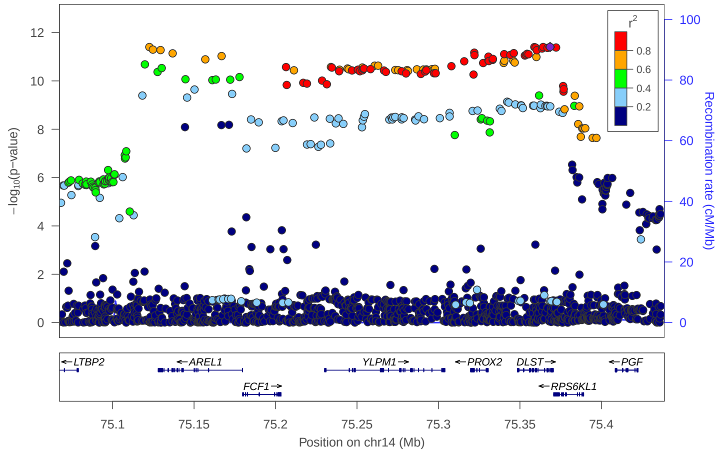

rs6574349

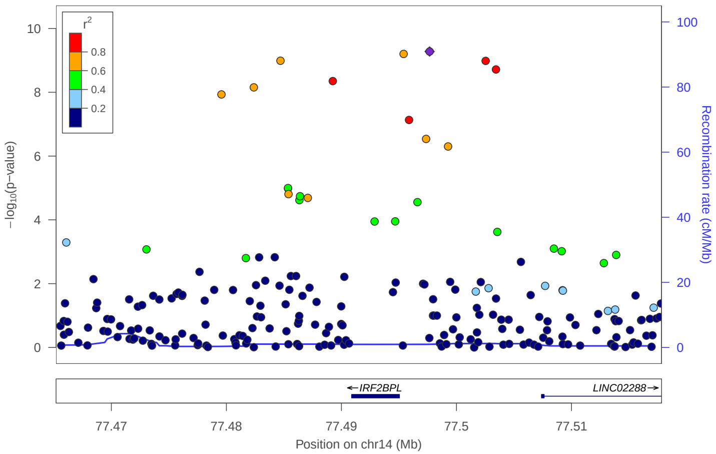

rs117618307

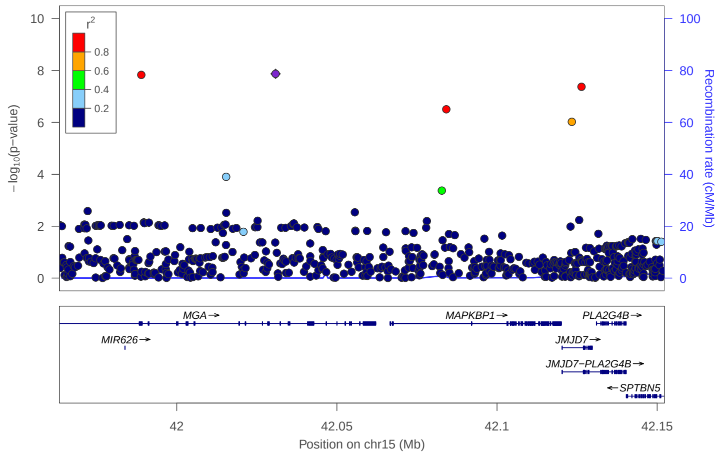

rs4787491

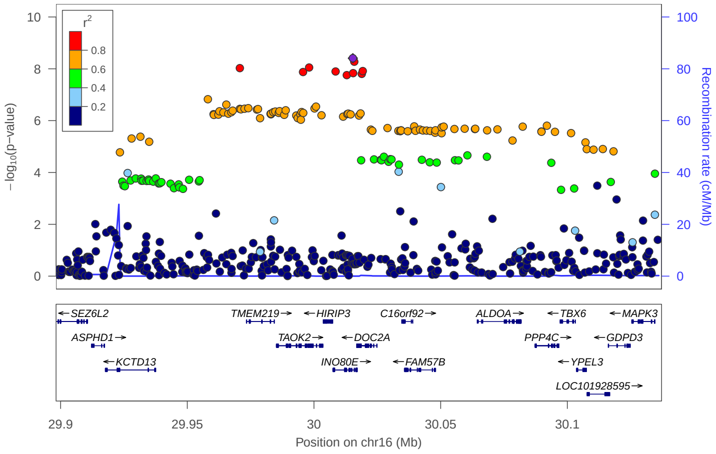

rs8050237

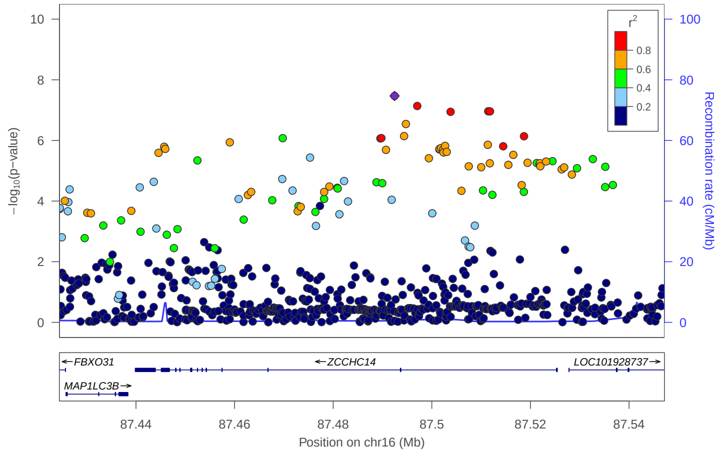

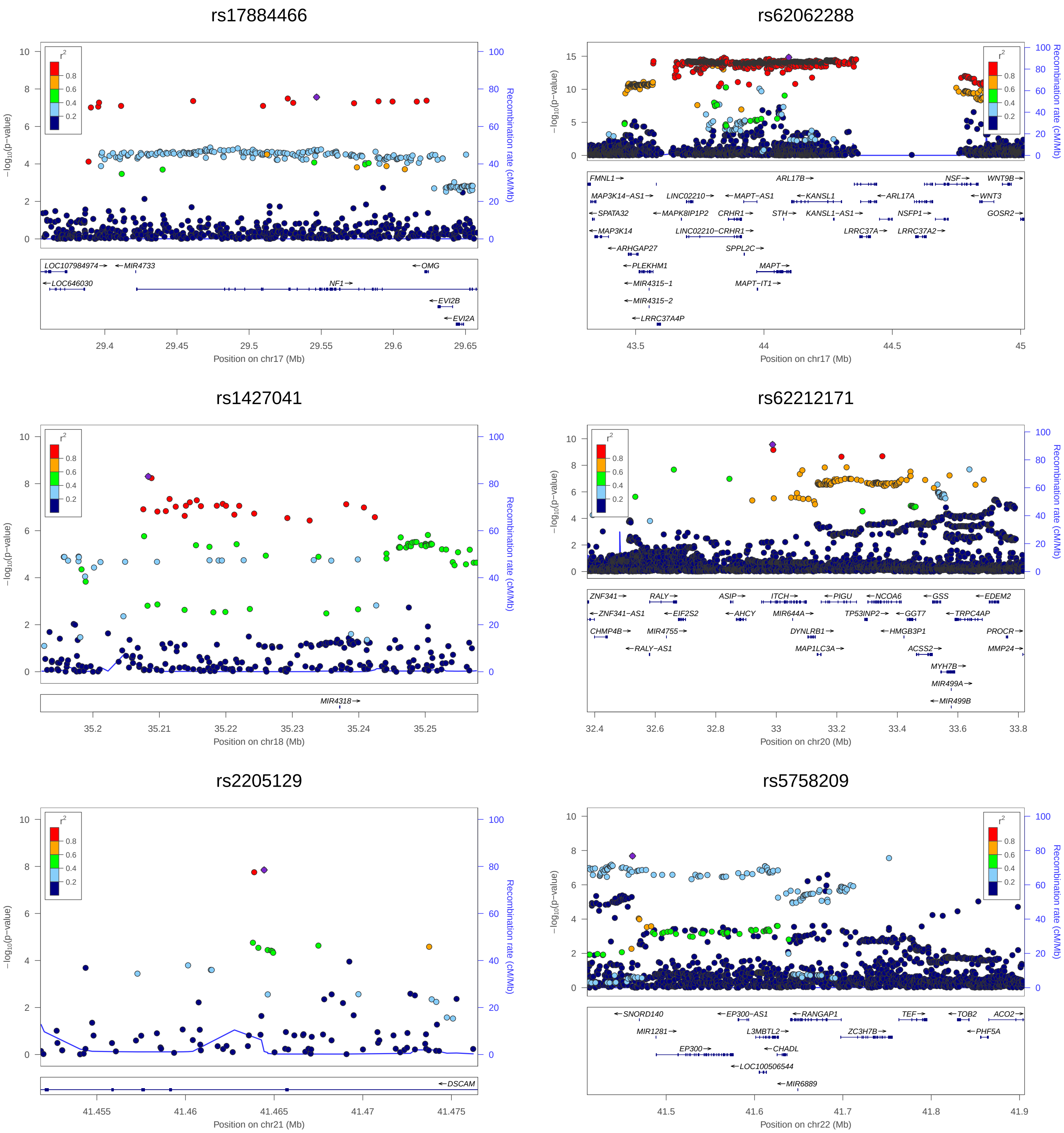
rs17884466
−log10(p−value)
Recombination rate (cM/Mb)
r2
LOC107984974→ ←MIR4733 ←OMG
←LOC646030 NF1→
←EVI2B
←EVI2A
Position on chr17 (Mb)
rs62062288
FMNL1→ ARL17B→ NSF→ WNT9B→
MAP3K14−AS1→ LINC02210→ ←MAPT−AS1 ←KANSL1 ←ARL17A ←WNT3
←SPATA32 ←MAPK8IP1P2 CRHR1→ STH→ KANSL1−AS1→ NSFP1→ GOSR2→
←MAP3K14 LINC02210−CRHR1→ LRRC37A→ LRRC37A2→
←ARHGAP27 SPPL2C→
←PLEKHM1 MAPT→
←MIR4315−1 MAPT−IT1→
←MIR4315−2
←LRRC37A4P
Position on chr17 (Mb)
rs1427041
MIR4318→
Position on chr18 (Mb)
rs62212171
ZNF341→ RALY→ ASIP→ ITCH→ ←PIGU ←NCOA6 ←GSS ←EDEM2
←ZNF341−AS1 ←EIF2S2 ←AHCY MIR644A→ TP53INP2→ ←GGT7 ←TRPC4AP
CHMP4B→ MIR4755→ DYNLRB1→ ←HMGB3P1 PROCR→
←RALY−AS1 MAP1LC3A→ ACSS2→ MMP24→
MYH7B→
MIR499A→
←MIR499B
Position on chr20 (Mb)
rs2205129
←DSCAM
Position on chr21 (Mb)
rs5758209
←SNORD140 ←EP300−AS1 ←RANGAP1 TEF→ ←TOB2 ACO2→
MIR1281→ L3MBTL2→ ZC3H7B→ ←PHF5A
EP300→ ←CHADL
←LOC100506544
←MIR6889
Position on chr22 (Mb)

Supplementary Figure 10. Regional plots of GWA analyses of item NERV-FEEL.

The *y*-axis represents the negative logarithm (base 10) of the SNP *P* value and the *x*-axis the position on the chromosome, with the name and location of genes in the UCSC Genome Browser in the bottom panel. The SNP with the lowest *P* value in the region is marked by a purple diamond. The colors of the other SNPs indicate the r^2 of these SNPs with the lead SNP, with grey color indicating SNPs below the user-defined r^2. Plots are generated with LocusZoom. See **Supplementary Data 2** for an overview of all lead SNPs for which a regional plot is presented above. For excluded loci (see **Methods**; **Supplementary Data 3-4**) the rsID's are marked yellow.

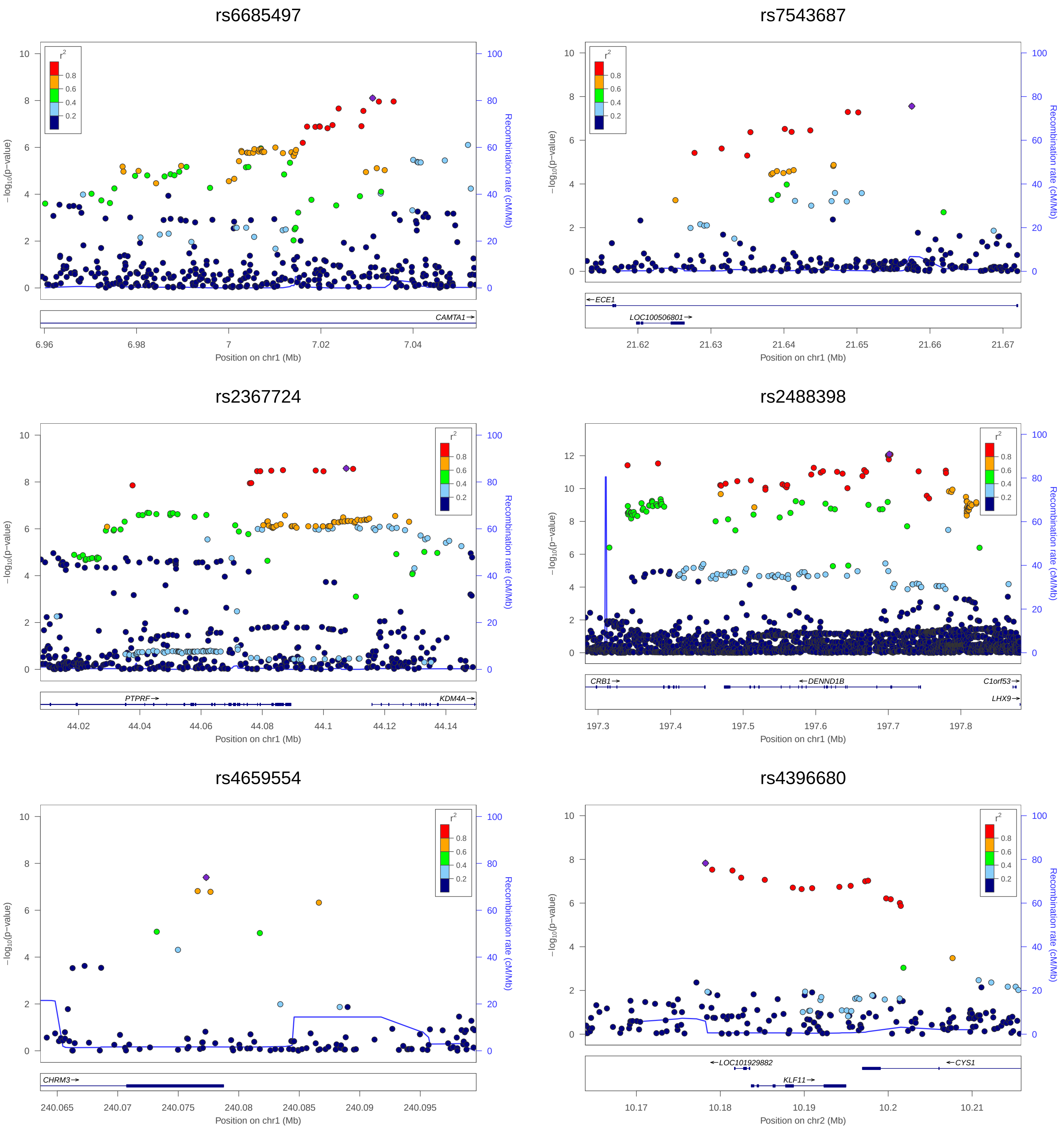
rs6685497
CAMTA1
Position on chr1 (Mb)
rs7543687
ECE1
LOC100506801
Position on chr1 (Mb)
rs2367724
PTPRF
KDM4A
Position on chr1 (Mb)
rs2488398
CRB1
DENND1B
C1orf53
LHX9
Position on chr1 (Mb)
rs4659554
CHRM3
Position on chr1 (Mb)
rs4396680
LOC101929882
CYS1
KLF11
Position on chr2 (Mb)
−log10(p−value)
Recombination rate (cM/Mb)

rs7567451

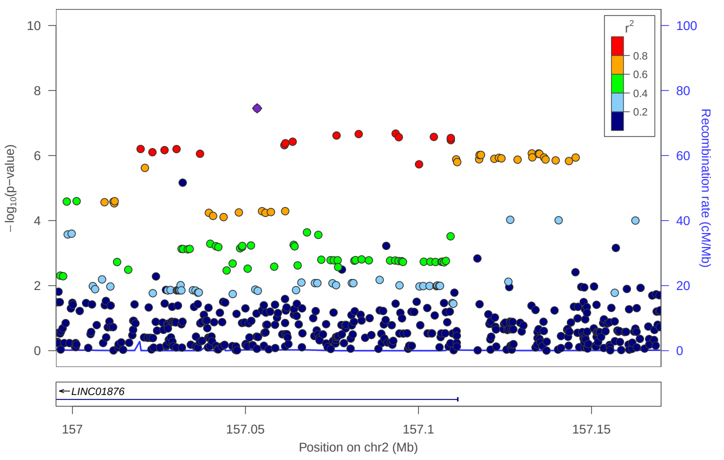

rs1375311

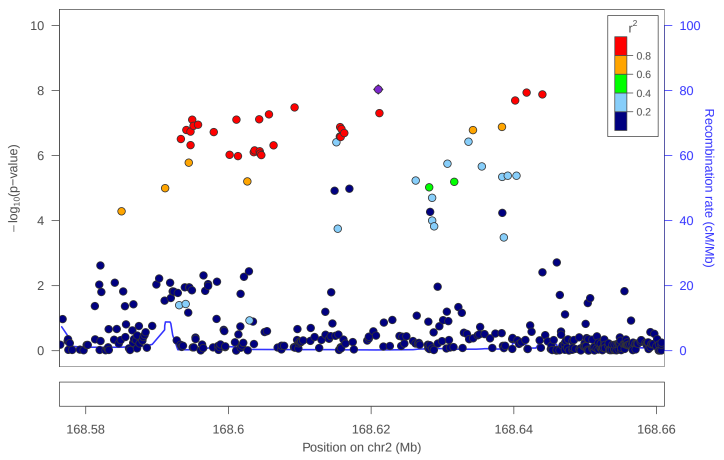

rs1542212

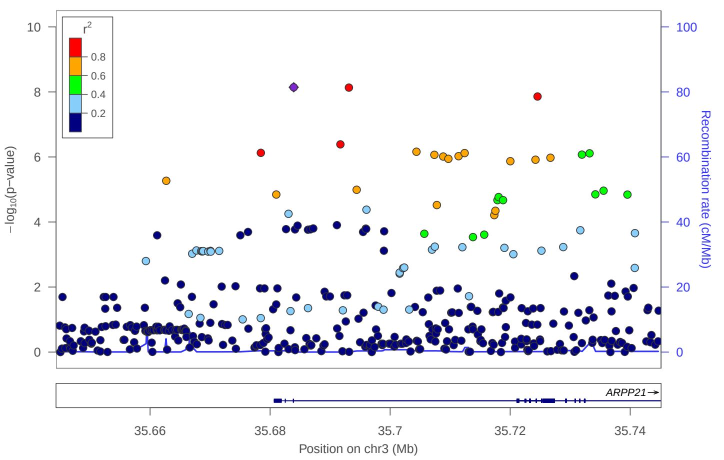

rs57462170

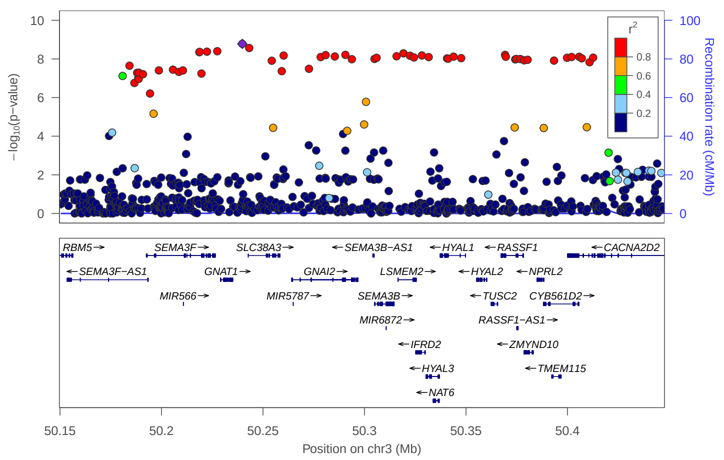

rs6798941

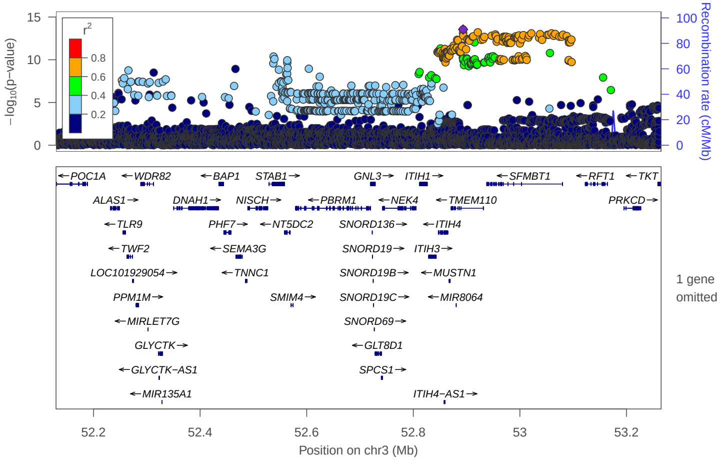

rs62250713

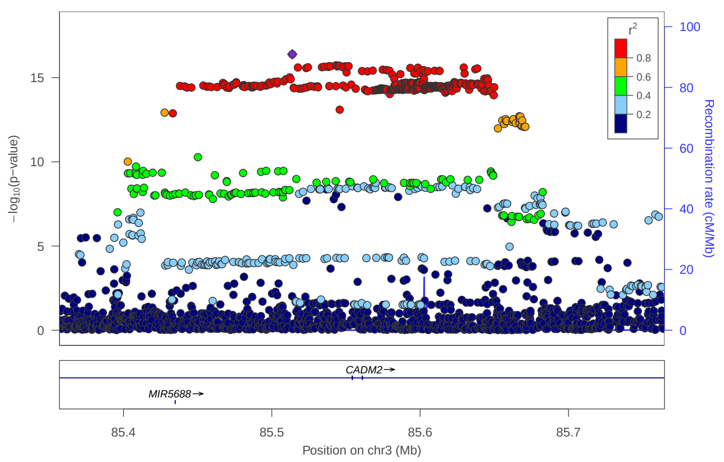

rs1697692

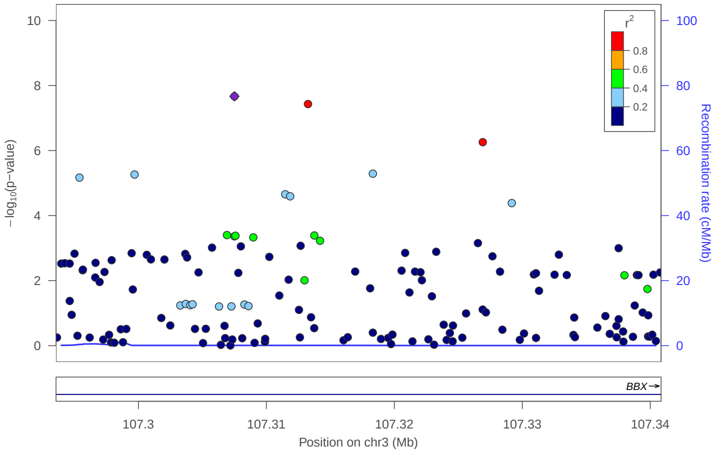

rs6791142

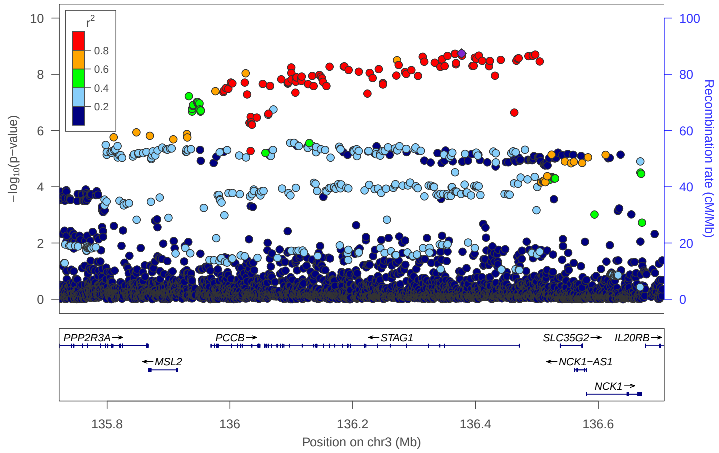

rs10034259

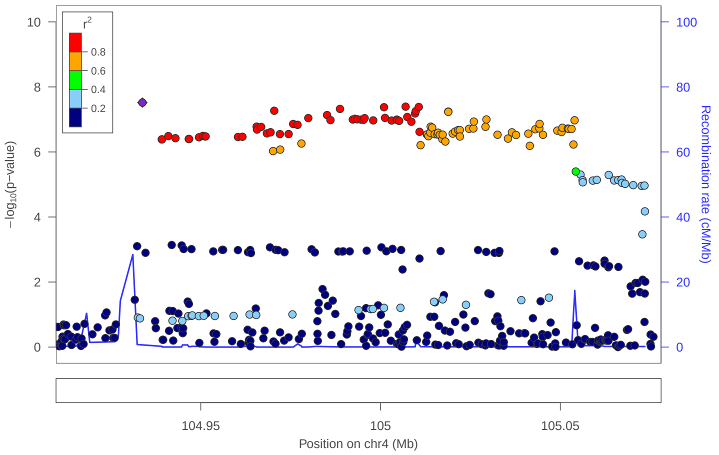

rs2389499

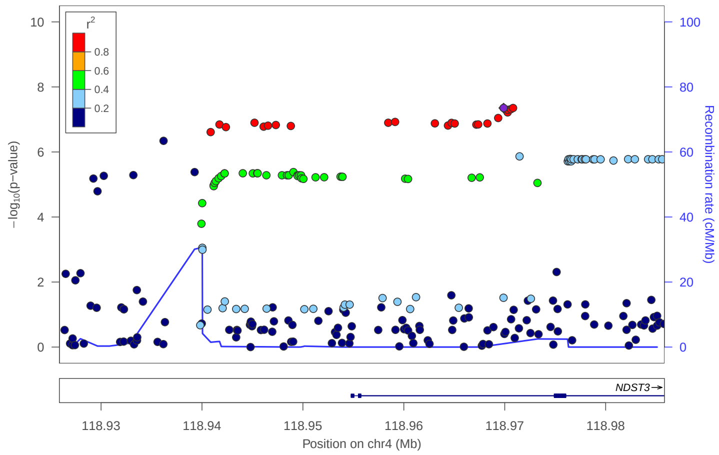

rs187580

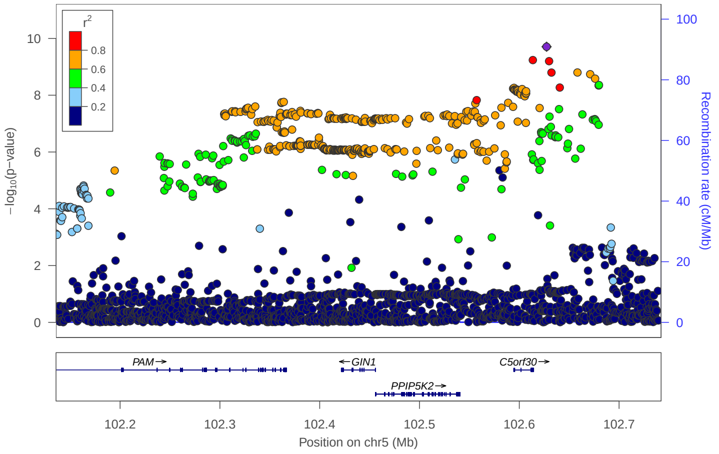

rs9462364

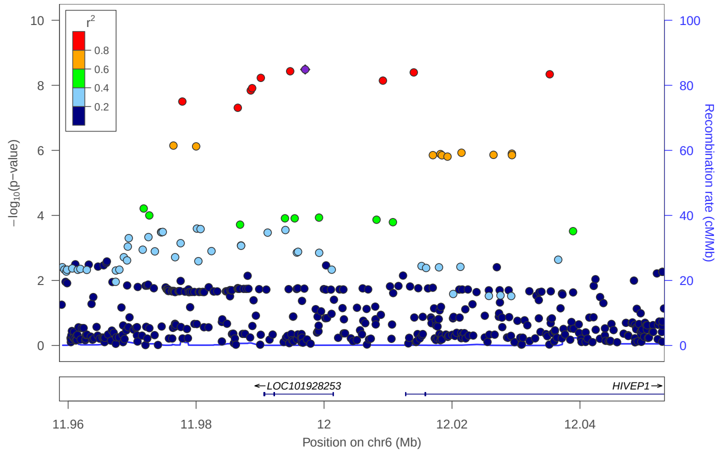

rs2269426

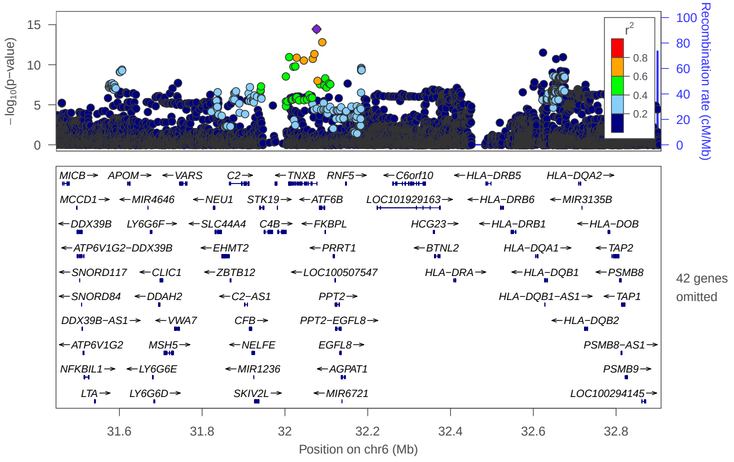

rs274632

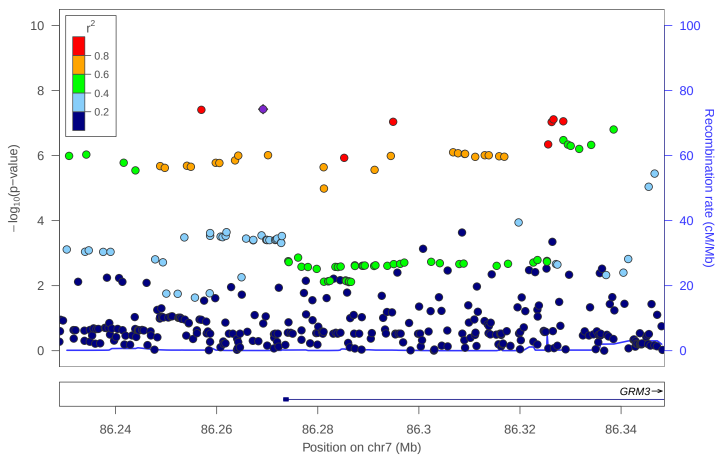

rs3808072

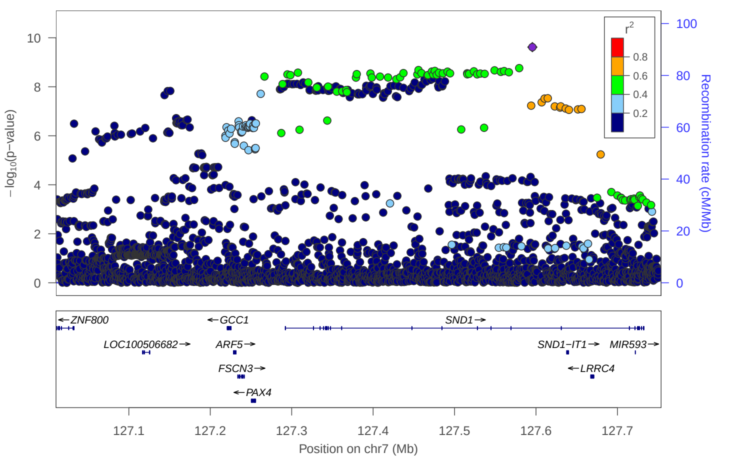

rs10096972

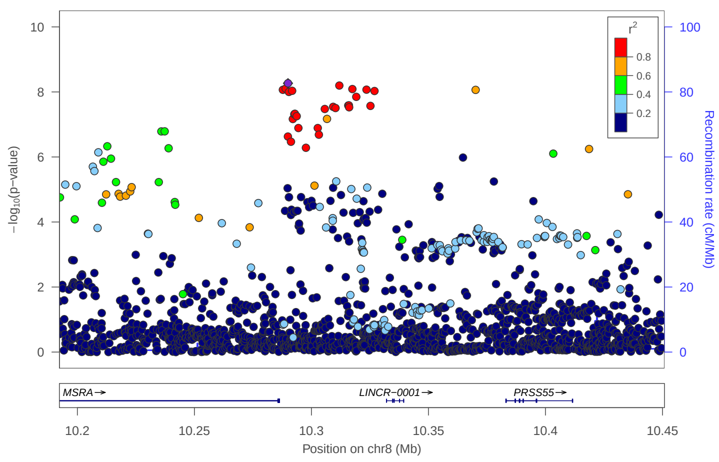

rs10959797

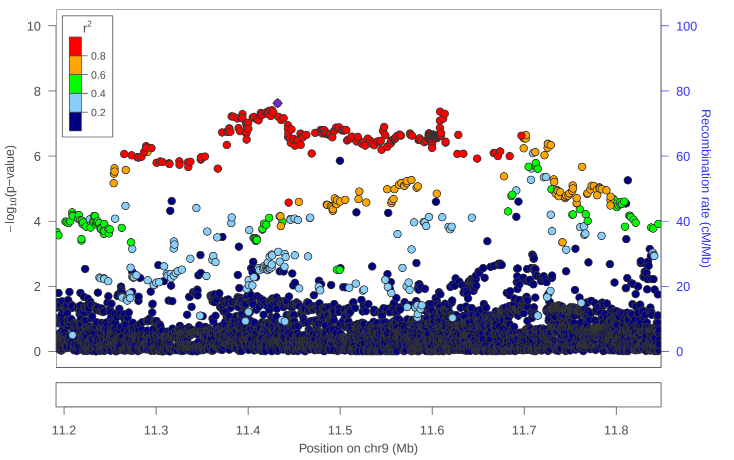

rs28510415

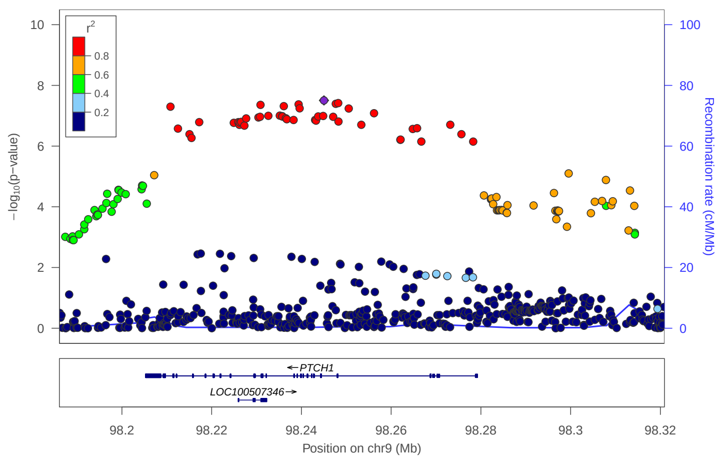

rs4919695

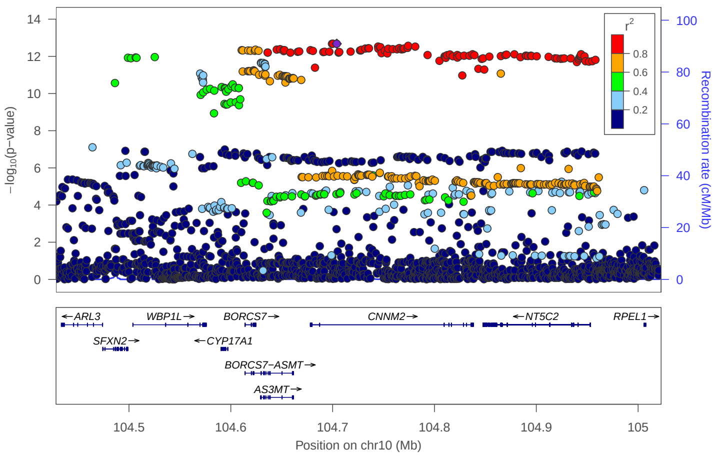

rs3026389

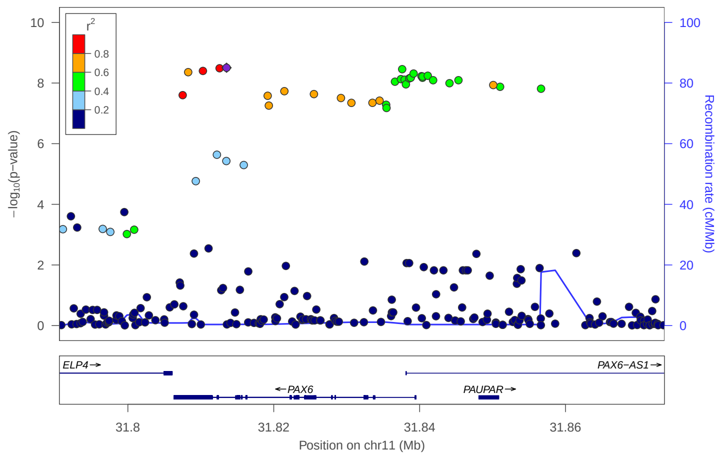

rs10765762

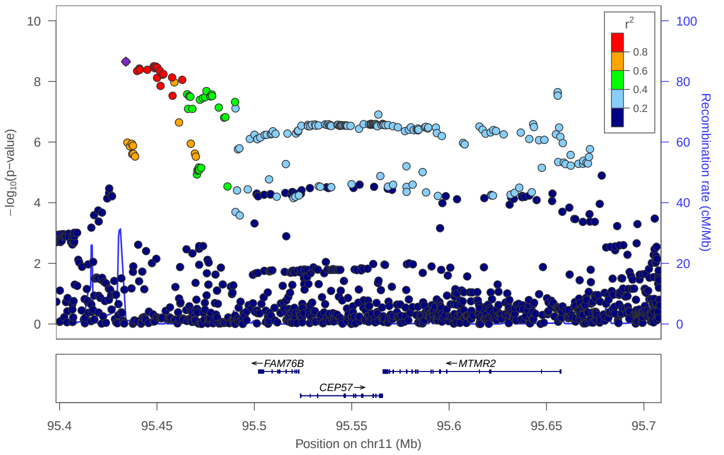

rs10750486

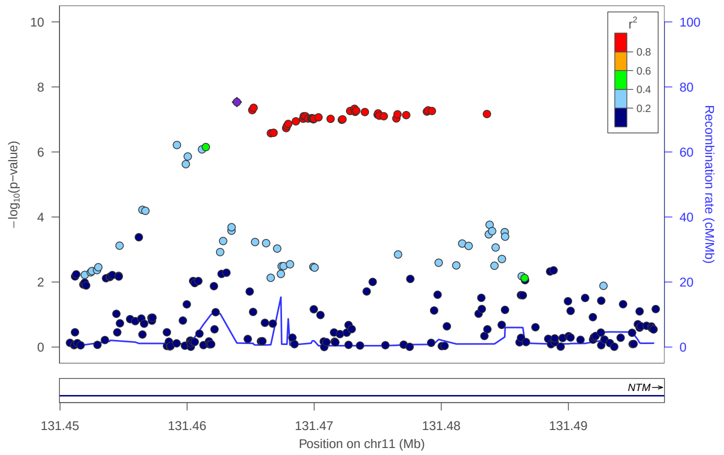

rs167915

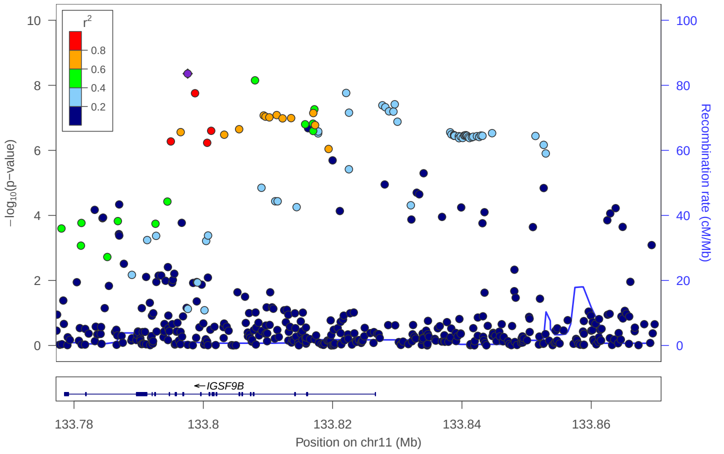

rs11067376

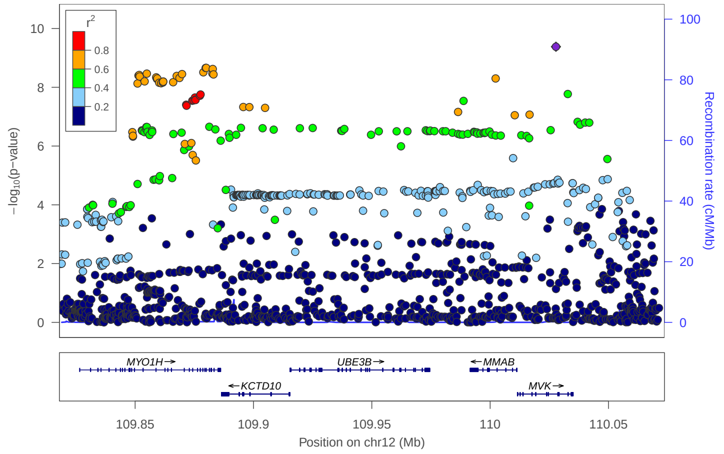

rs61957597

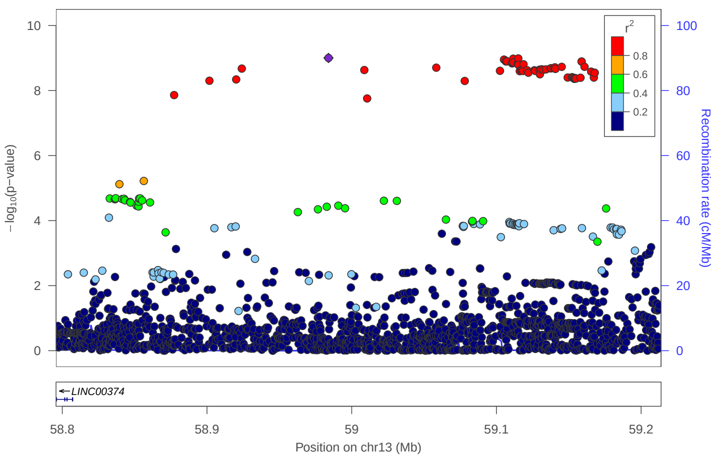

rs7152906

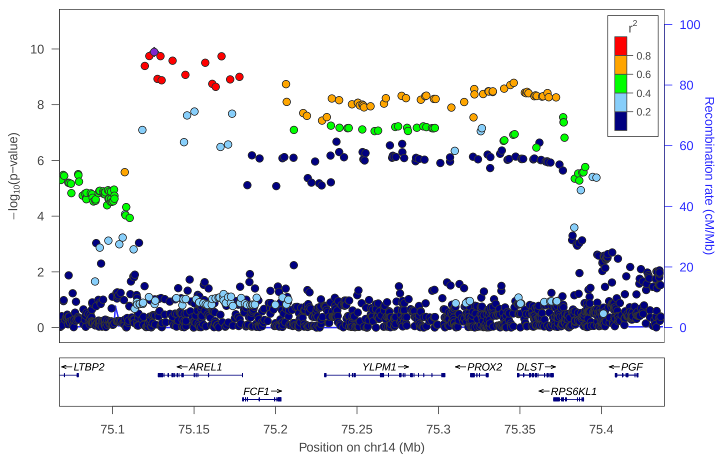

rs55997507

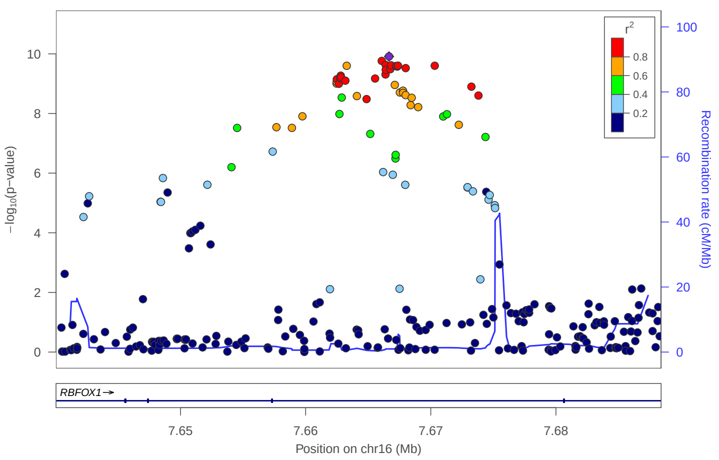

rs17688916

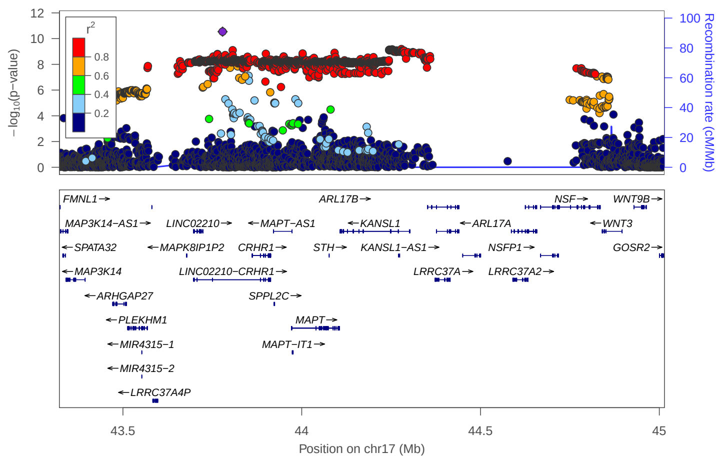

rs72893199

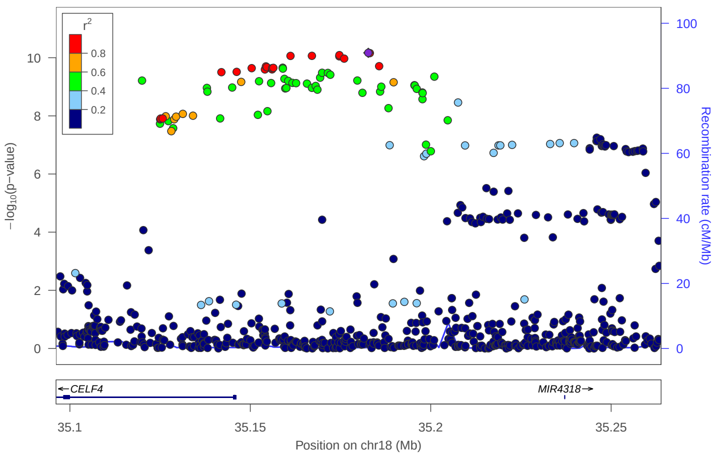

rs7228159

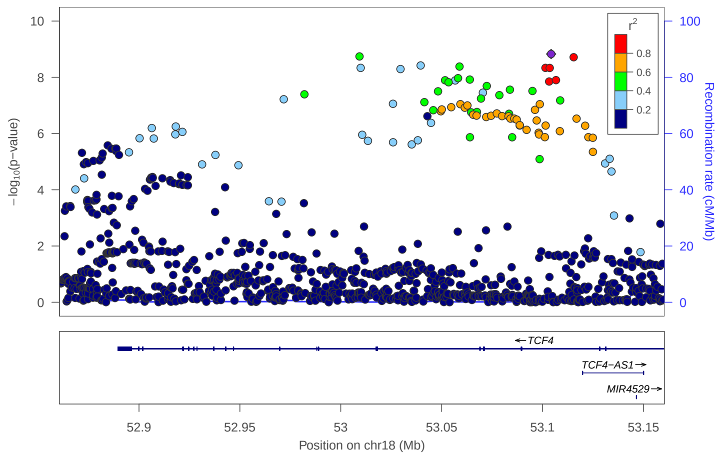

rs58084604

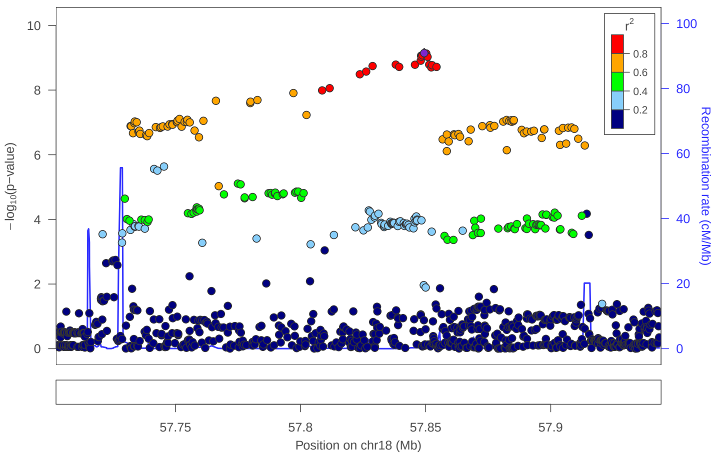

rs116962250

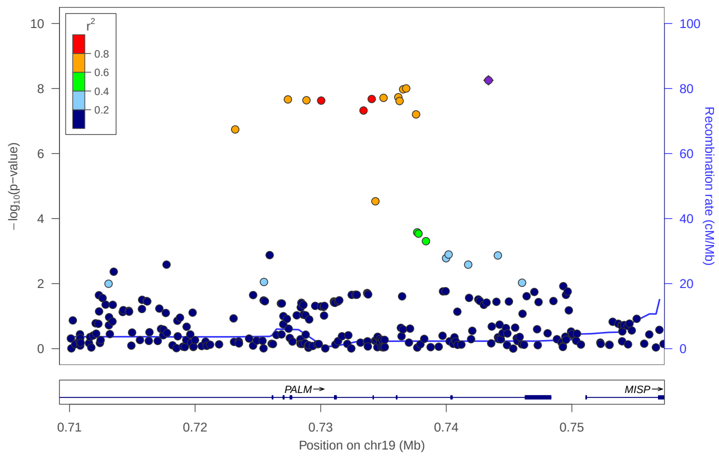

rs13054099

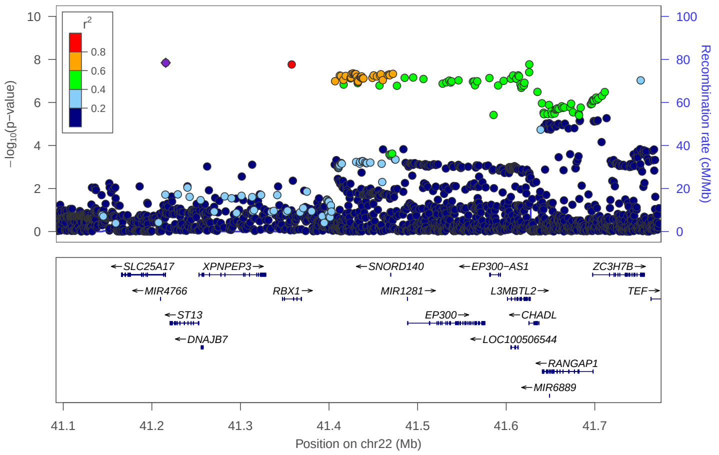

Supplementary Figure 11. Regional plots of GWA analyses of item WORRY.
The *y*-axis represents the negative logarithm (base 10) of the SNP *P* value and the *x*-axis the position on the chromosome, with the name and location of genes in the UCSC Genome Browser in the bottom panel. The SNP with the lowest *P* value in the region is marked by a purple diamond. The colors of the other SNPs indicate the r^2 of these SNPs with the lead SNP, with grey color indicating SNPs below the user-defined r^2. Plots are generated with LocusZoom. See **Supplementary Data 2** for an overview of all lead SNPs for which a regional plot is presented above.

rs2100888

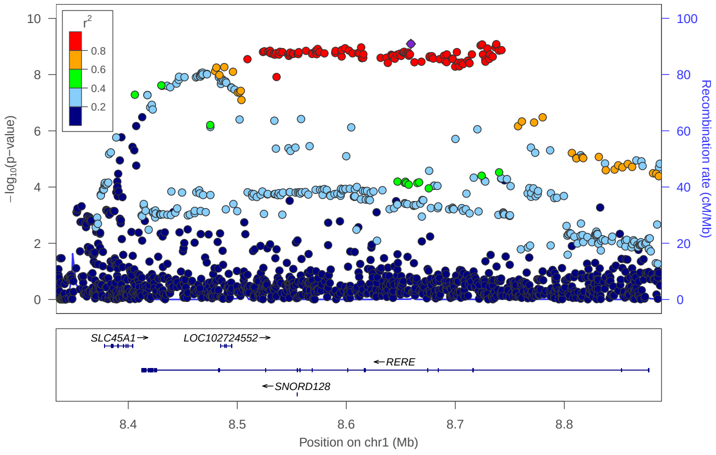

rs10218528

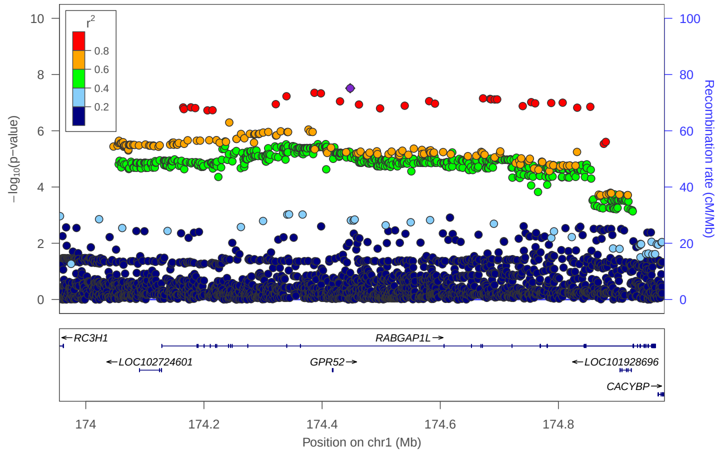

rs4671330

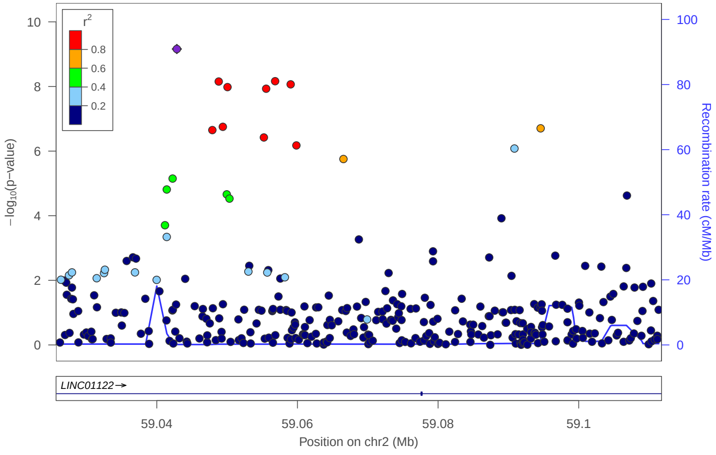

rs144851691

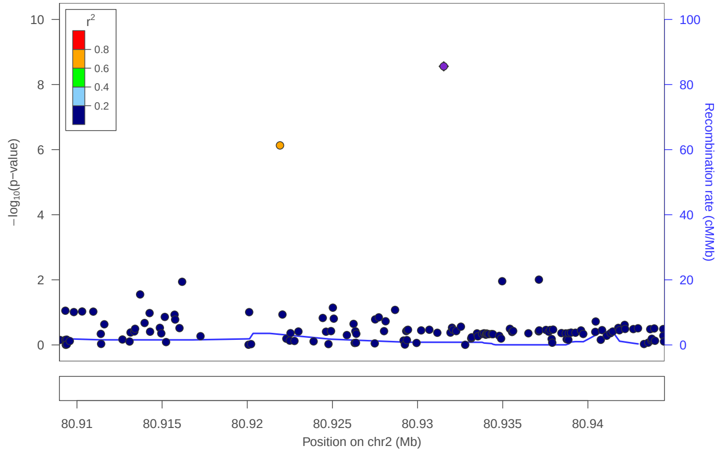

rs79861172

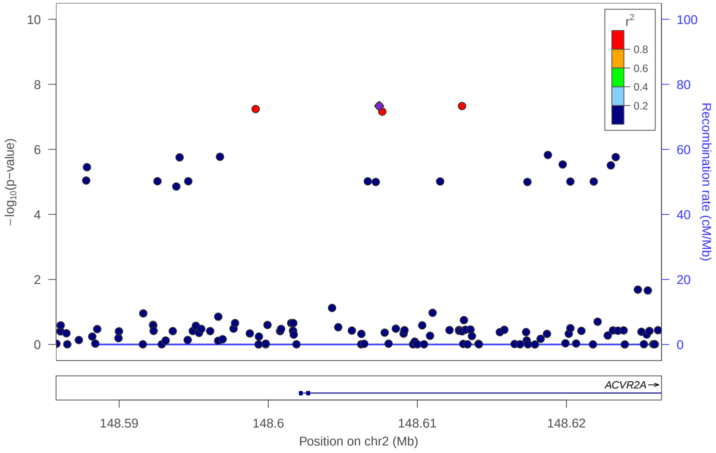

rs2097247

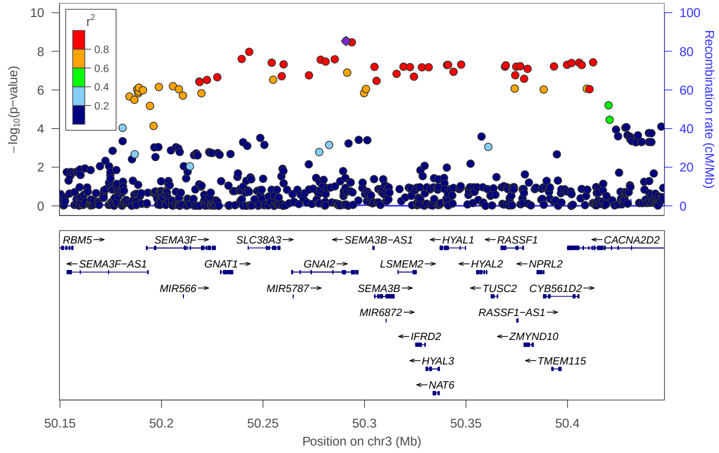

rs9811546

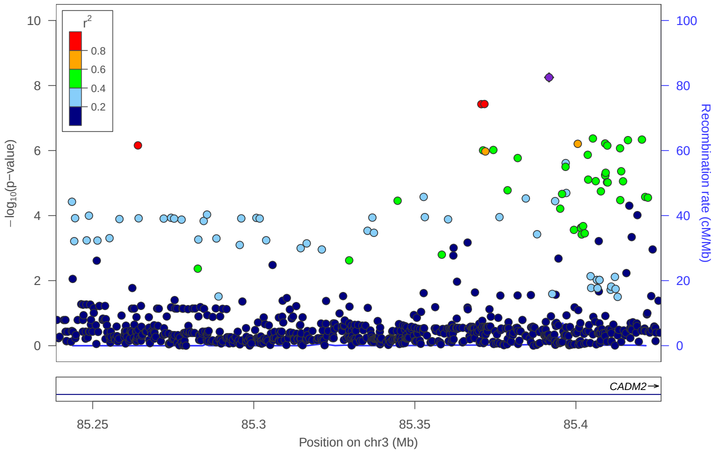

rs6891955

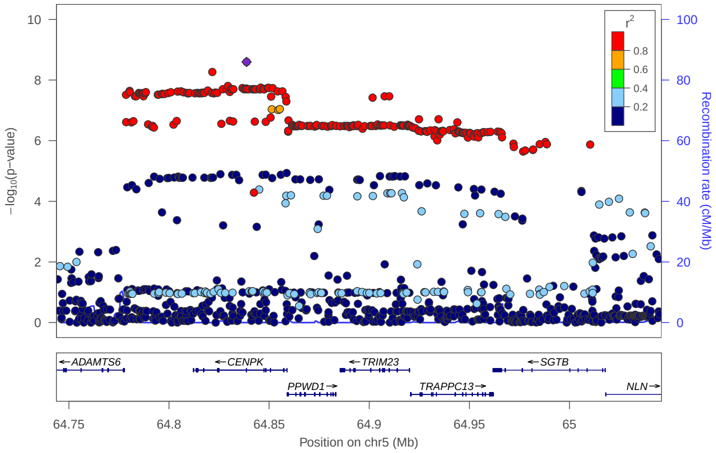

rs28738966

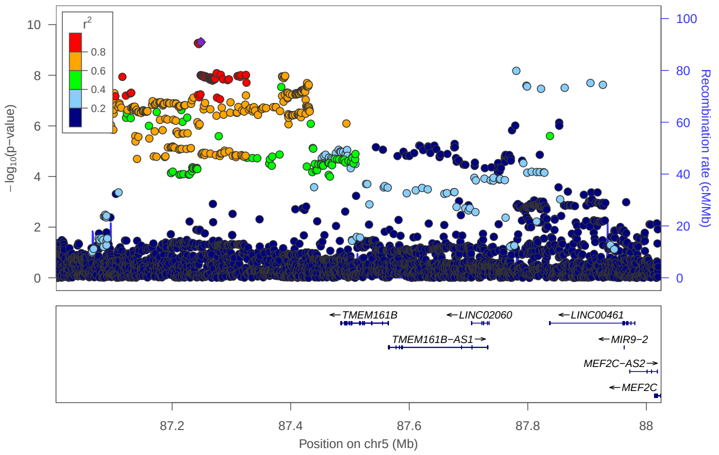

rs6883228

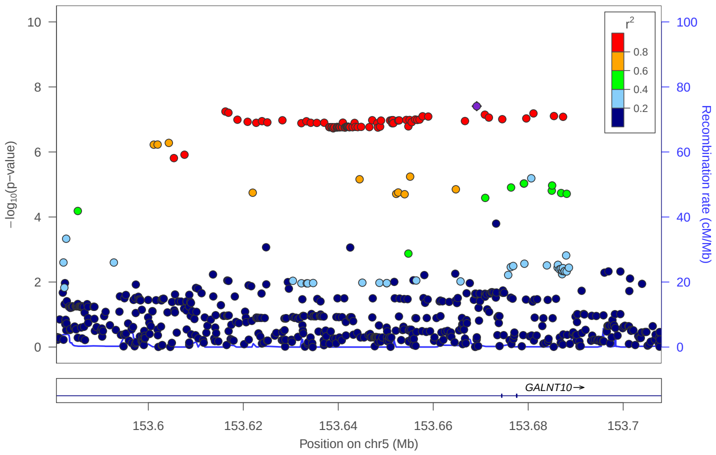

rs1147851

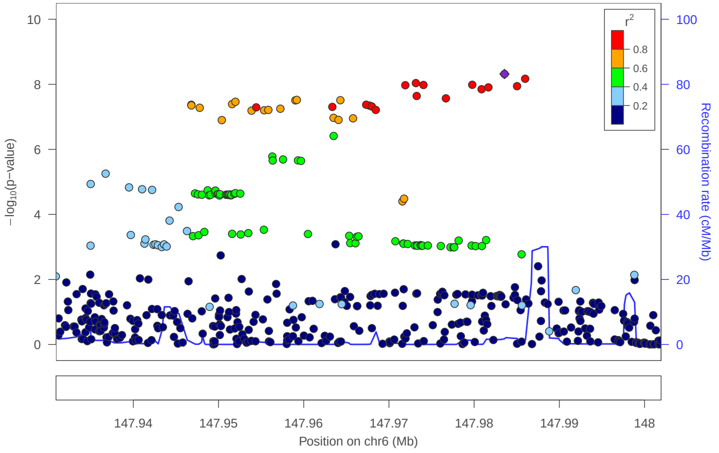

rs11509880

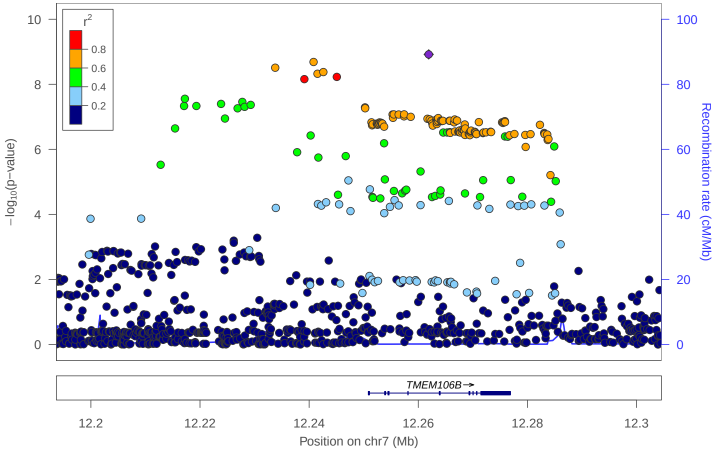

rs1450832

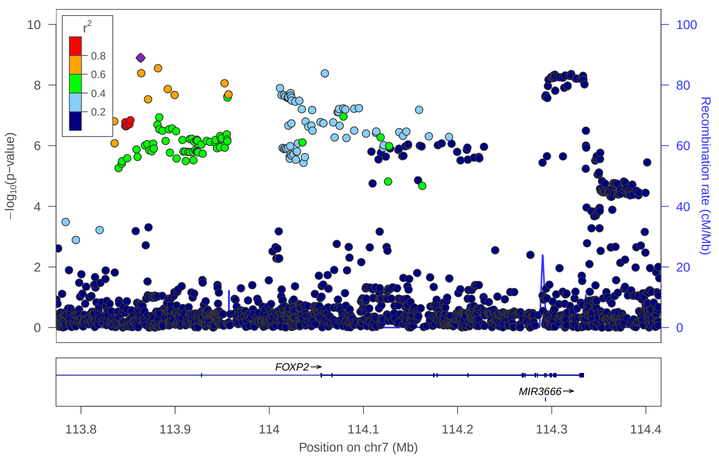

rs4129585

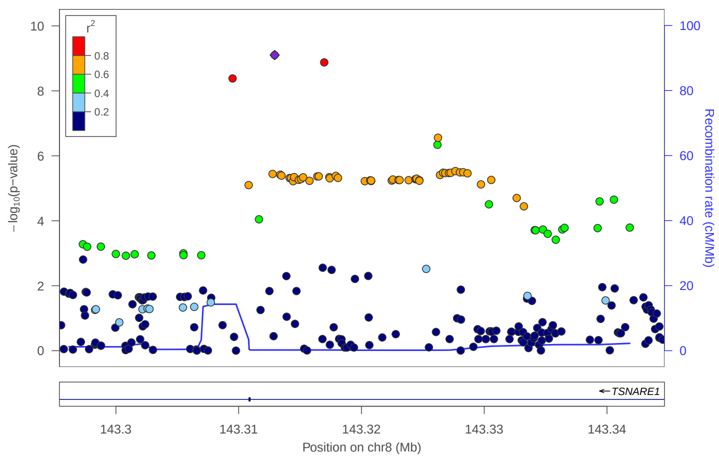

rs3740393

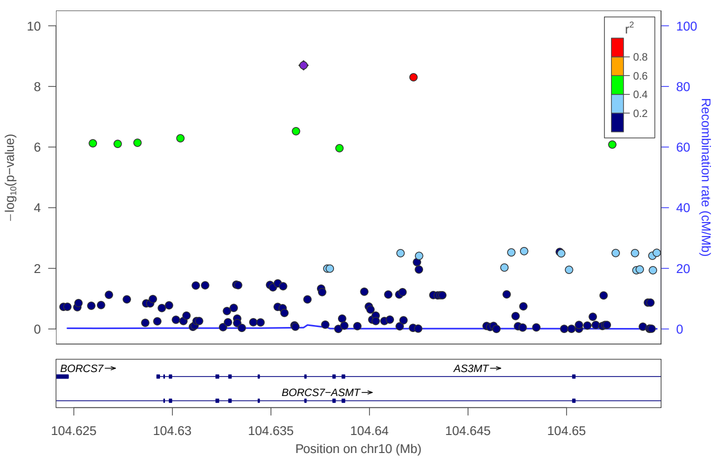

rs10767733

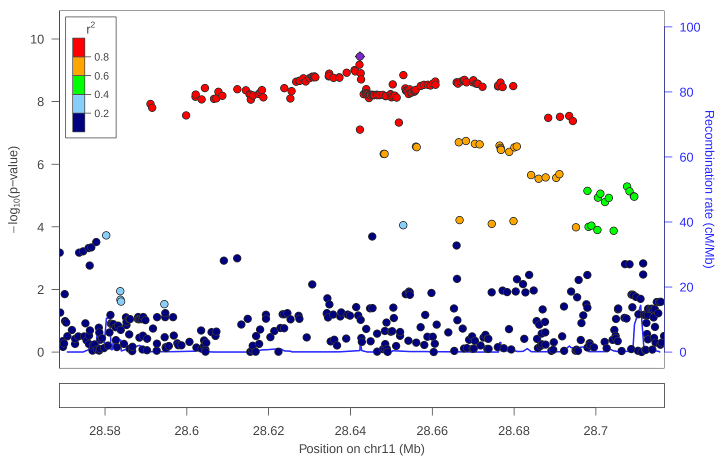

rs4937872

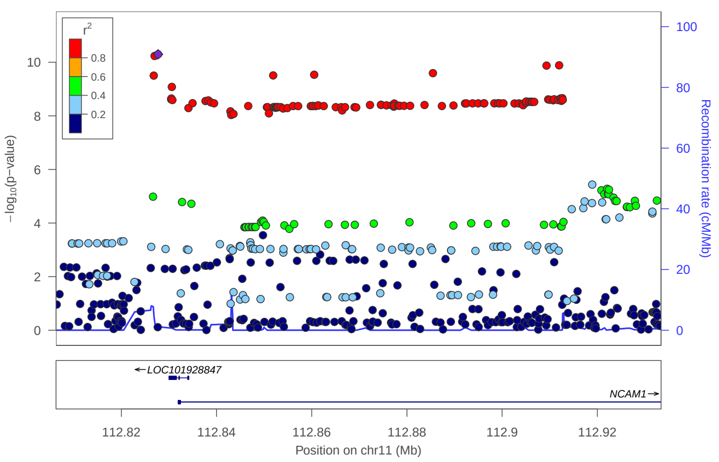

rs9527336

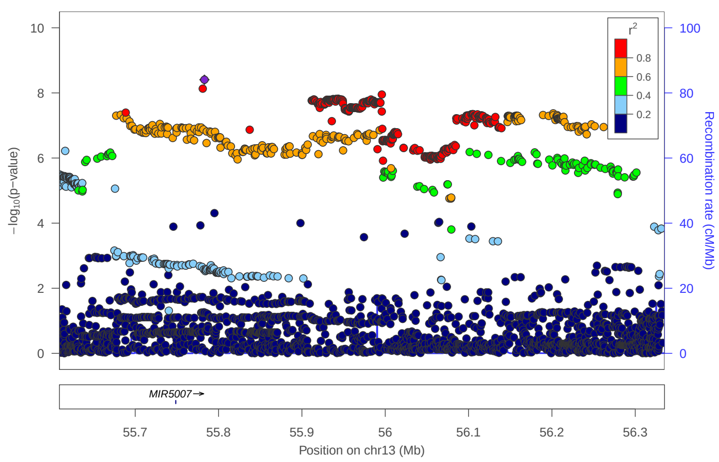

rs3751855

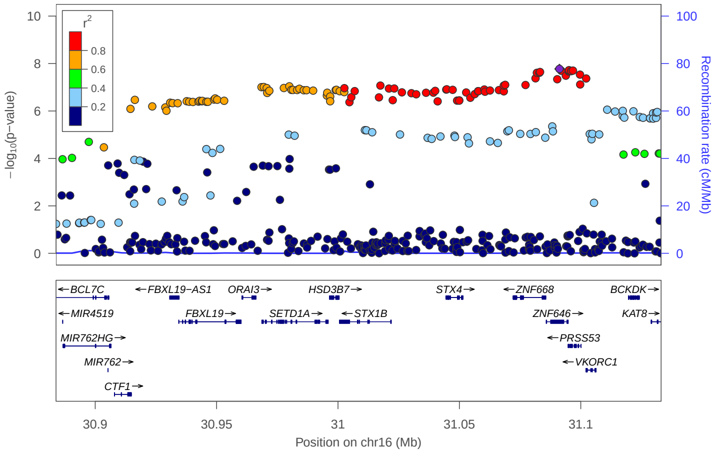

rs7194615

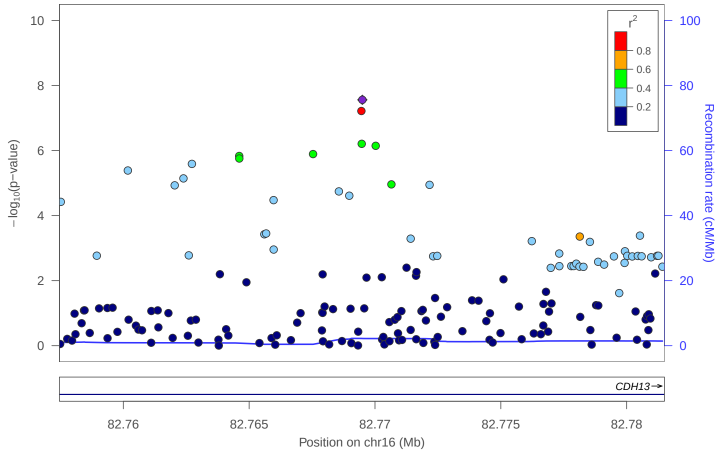

rs56084168

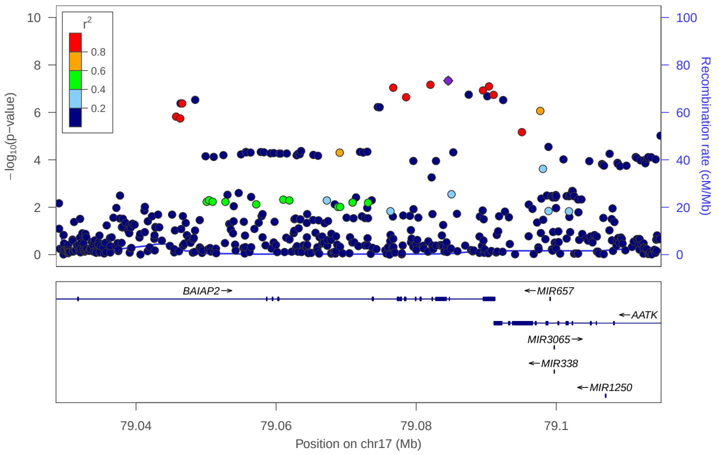

rs78379741

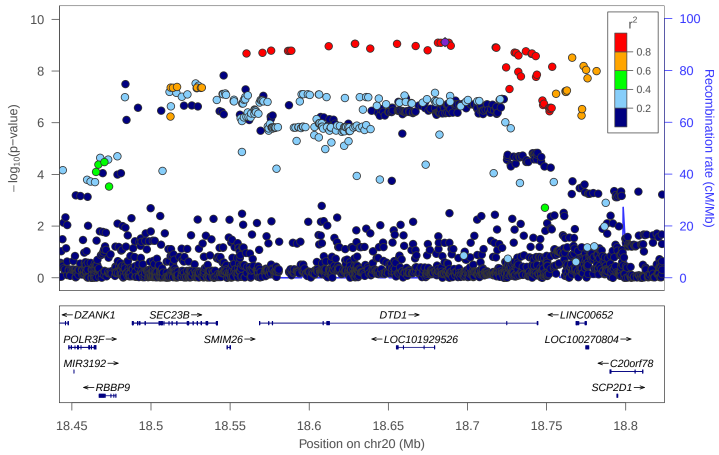

rs11090045

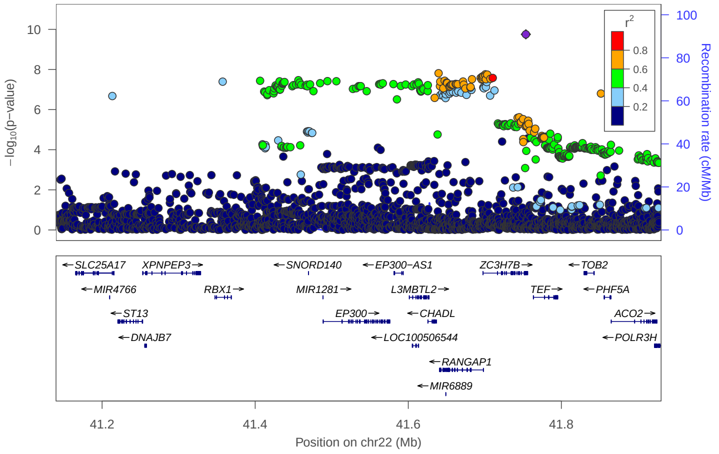

Supplementary Figure 12. Regional plots of GWA analyses of item TENSE.

The *y*-axis represents the negative logarithm (base 10) of the SNP *P* value and the *x*-axis the position on the chromosome, with the name and location of genes in the UCSC Genome Browser in the bottom panel. The SNP with the lowest *P* value in the region is marked by a purple diamond. The colors of the other SNPs indicate the r^2 of these SNPs with the lead SNP, with grey color indicating SNPs below the user-defined r^2. Plots are generated with LocusZoom. See **Supplementary Data 2** for an overview of all lead SNPs for which a regional plot is presented above. For excluded loci (see **Methods**; **Supplementary Data 3-4**) the rsID's are marked yellow.

rs9854869

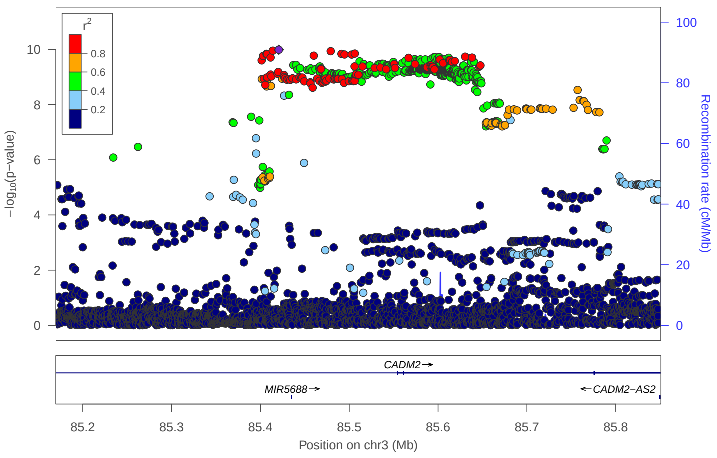

rs6478623

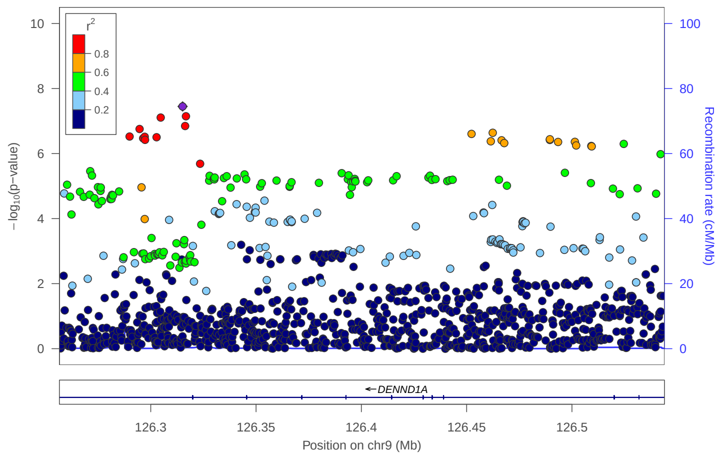

rs12787112

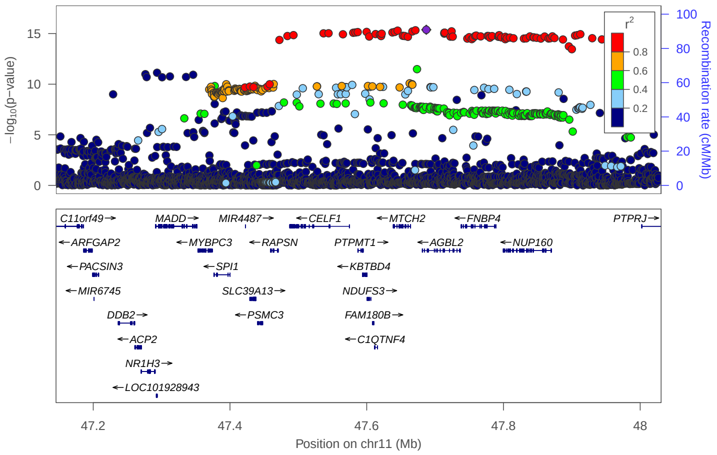

rs2193596

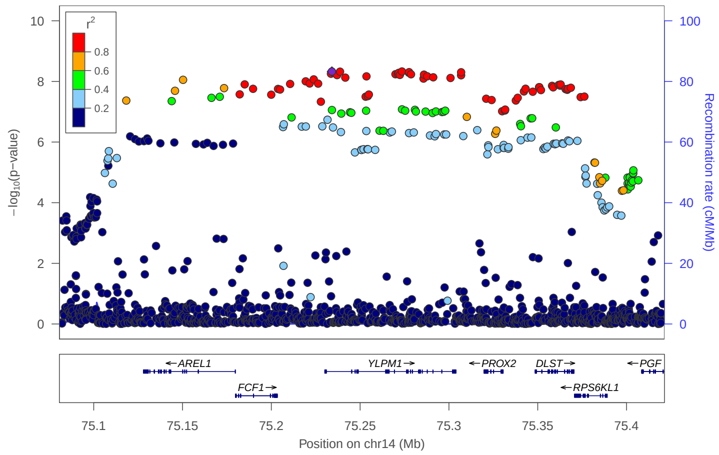

rs62081501

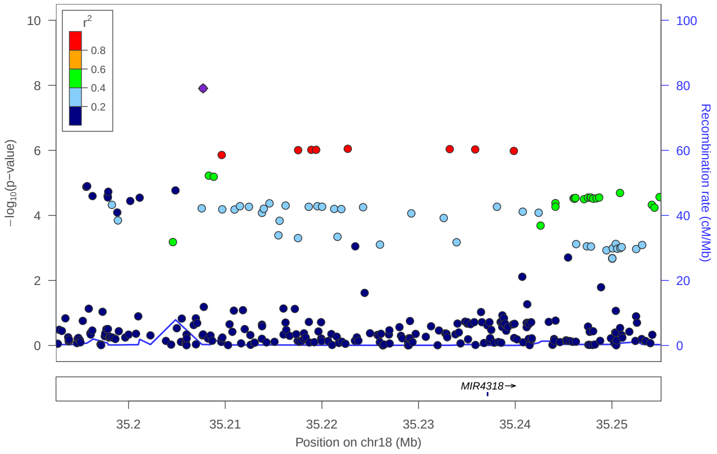

rs11665052

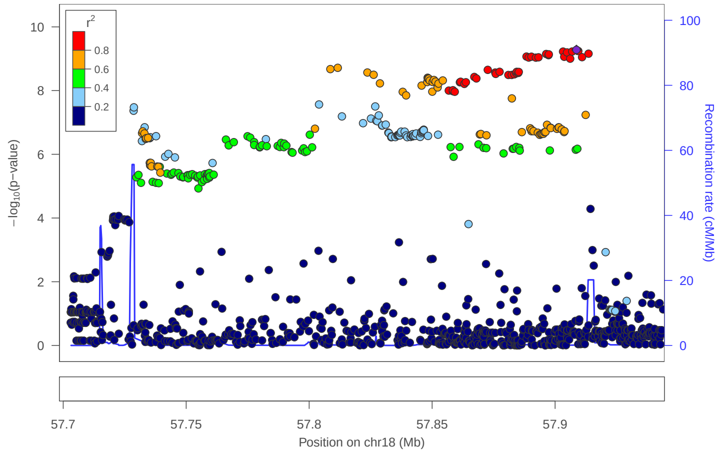

Supplementary Figure 13. Regional plots of GWA analyses of item SUF-NERV.
The *y*-axis represents the negative logarithm (base 10) of the SNP *P* value and the *x*-axis the position on the chromosome, with the name and location of genes in the UCSC Genome Browser in the bottom panel. The SNP with the lowest *P* value in the region is marked by a purple diamond. The colors of the other SNPs indicate the r^2 of these SNPs with the lead SNP, with grey color indicating SNPs below the user-defined r^2. Plots are generated with LocusZoom. See **Supplementary Data 2** for an overview of all lead SNPs for which a regional plot is presented above.

rs12028465

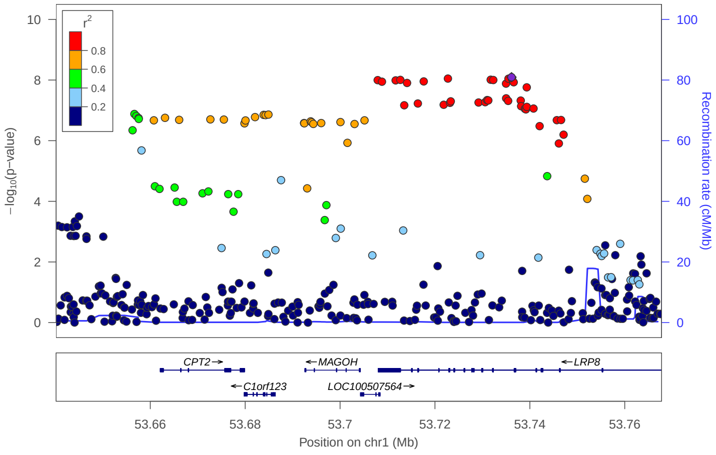

rs4652676

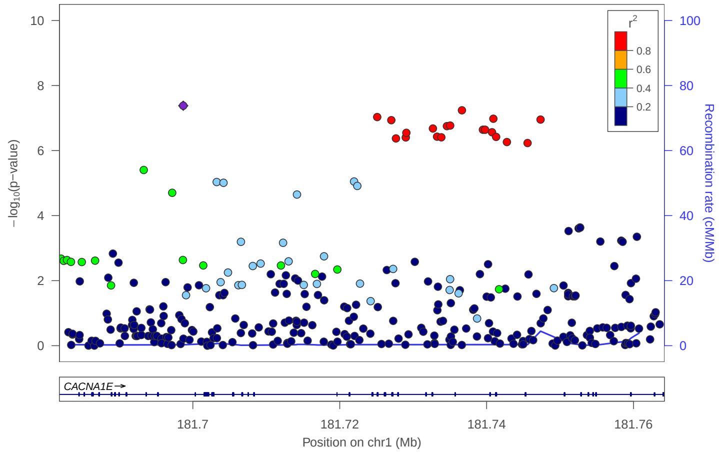

rs2488401

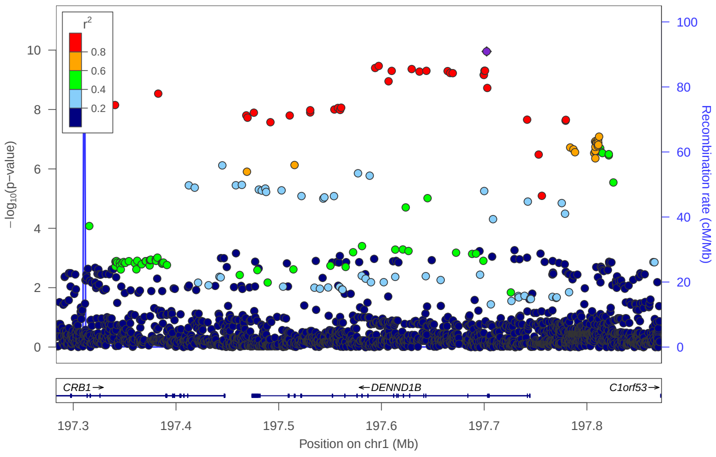

rs10210652

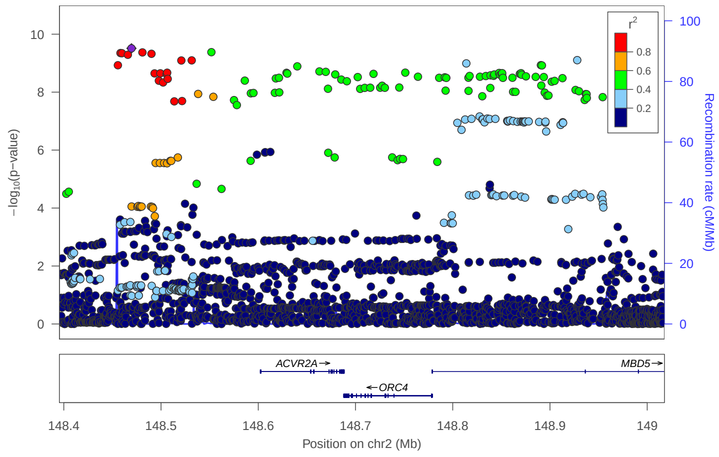

rs1978573

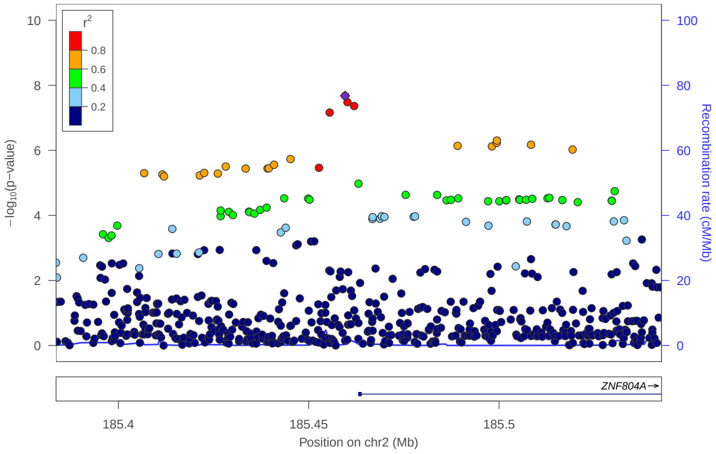

rs219226

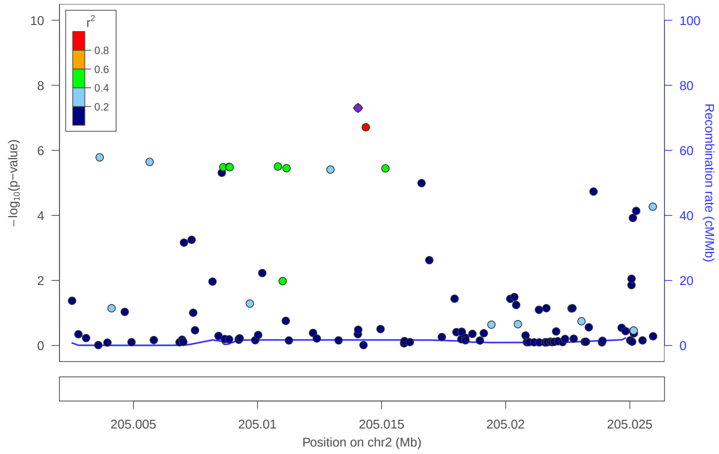

rs10511285

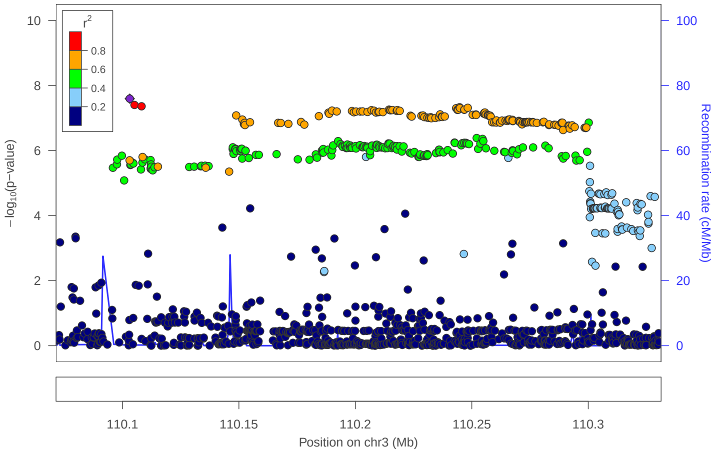

rs62268962

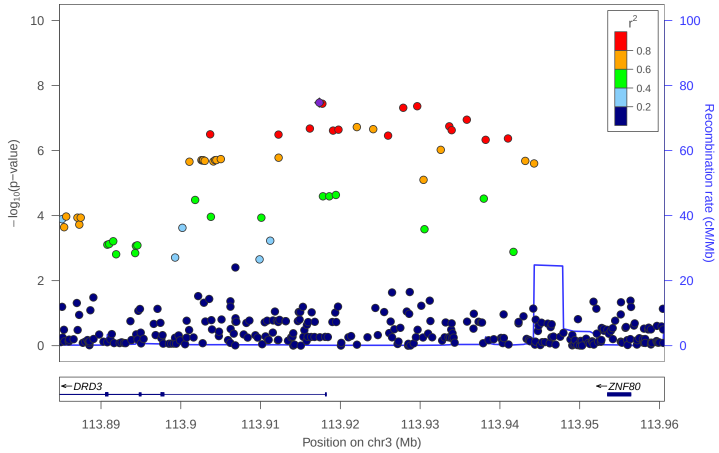

rs2027798

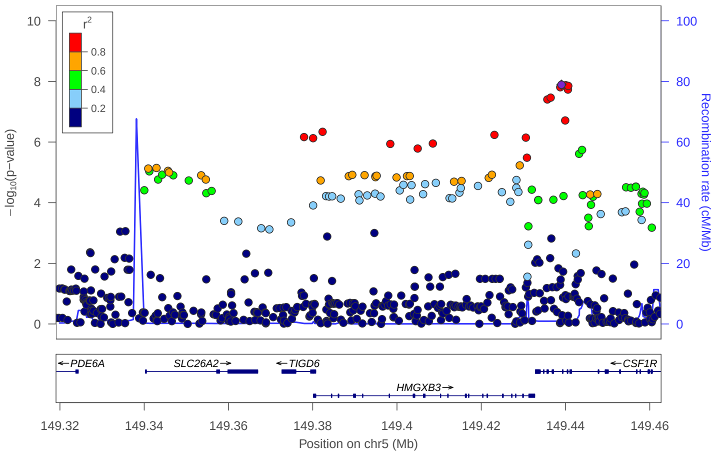

rs4868774

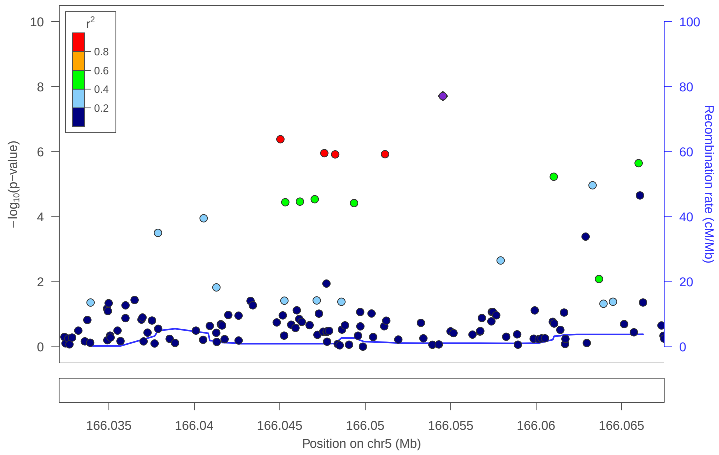

rs8111

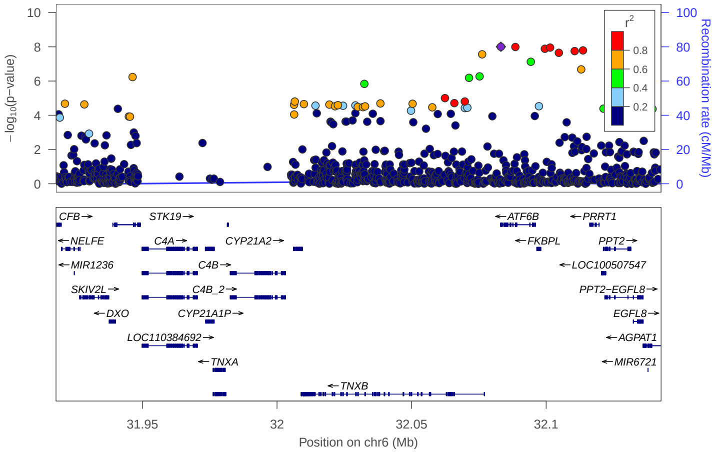

rs11767715

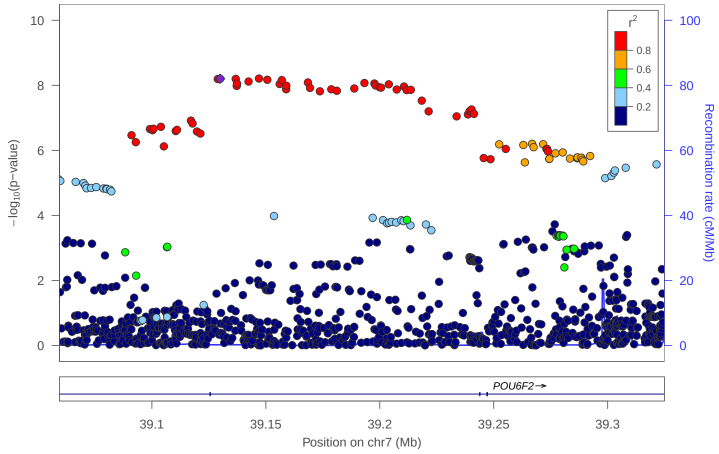

rs34548930

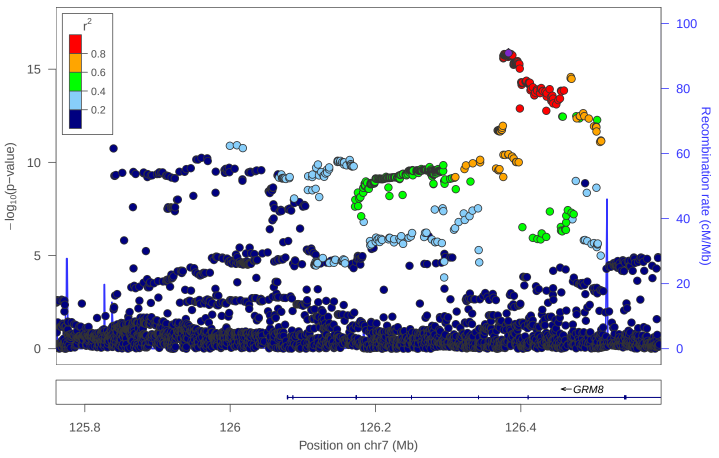

rs10117184

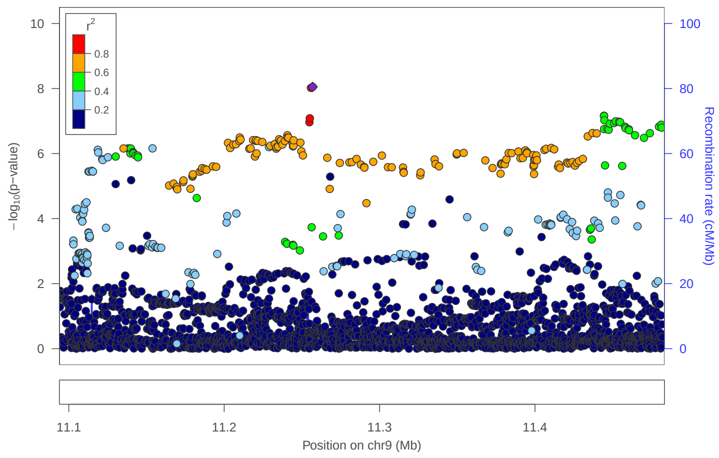

rs1231375

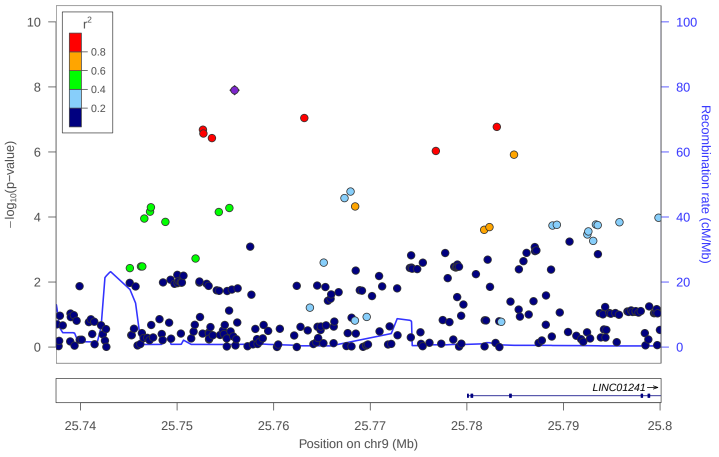

rs17532098

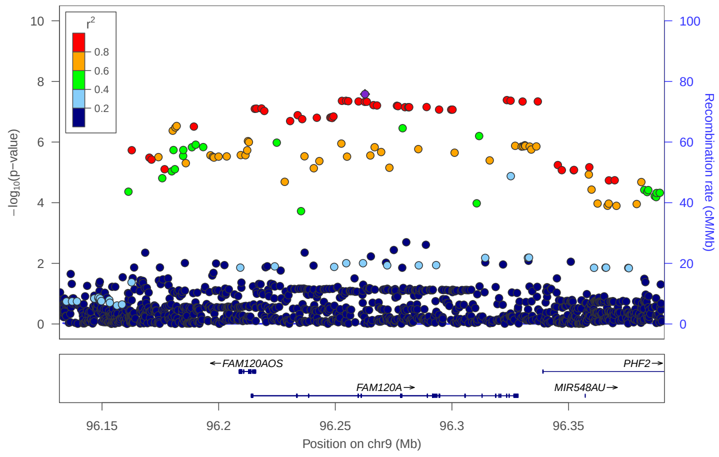

rs145965565

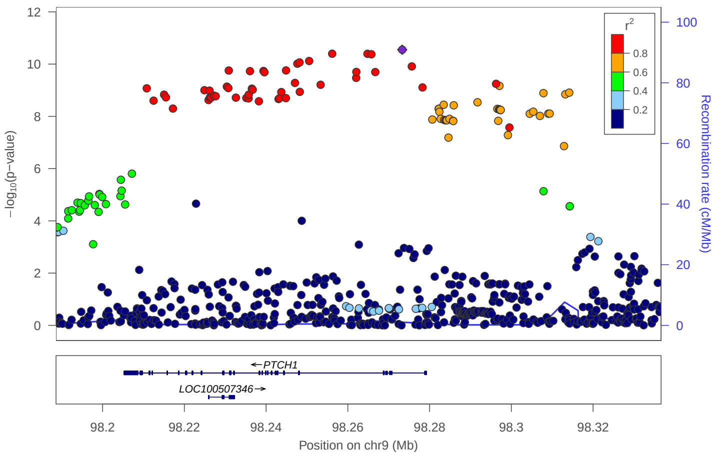

rs73480560

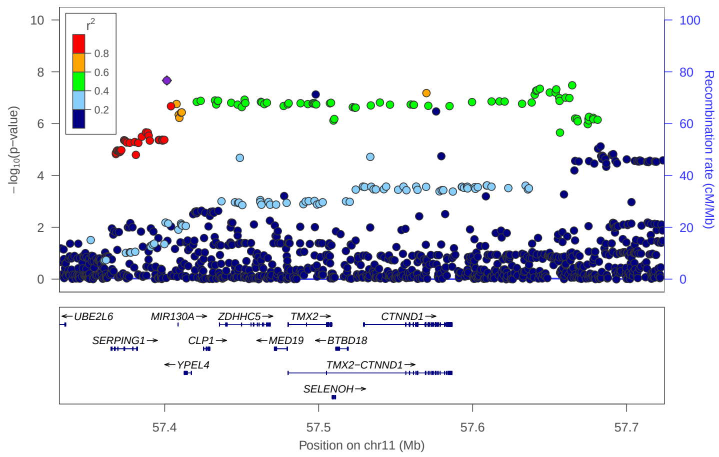

rs10891564

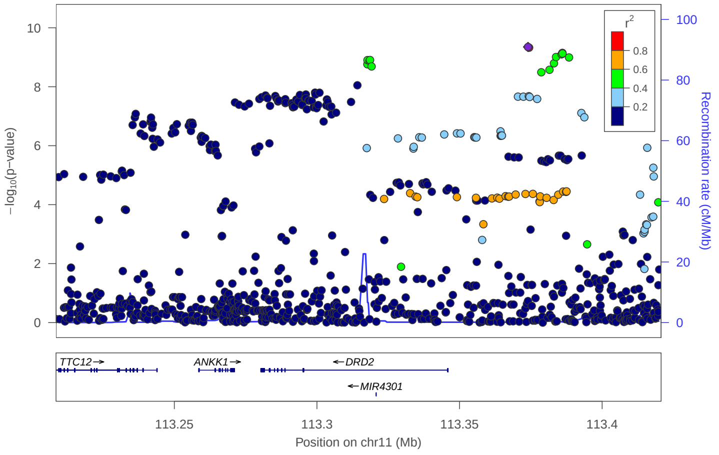

rs10850379

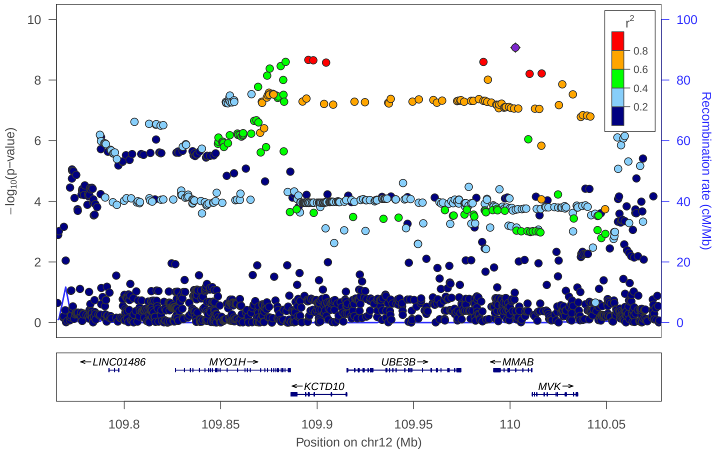

rs2102923

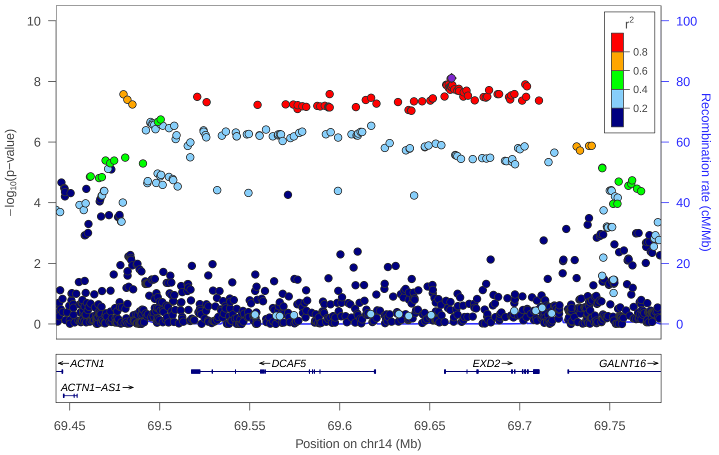

rs4702

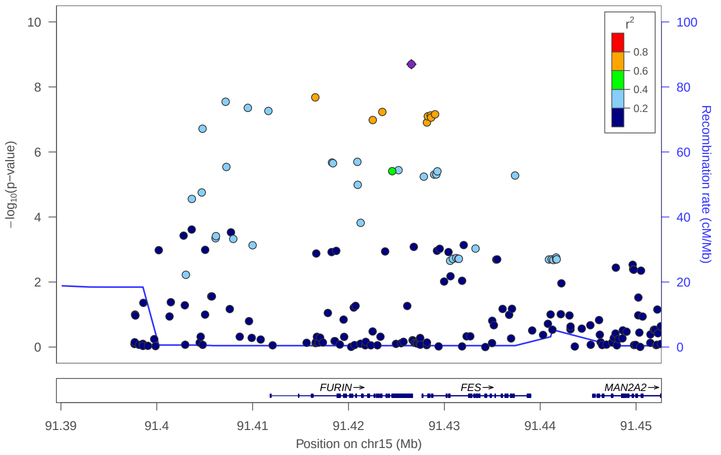

rs12933611

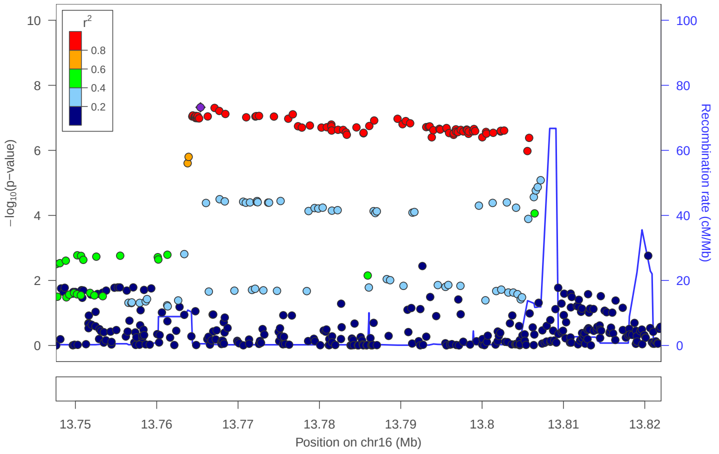

rs62035176

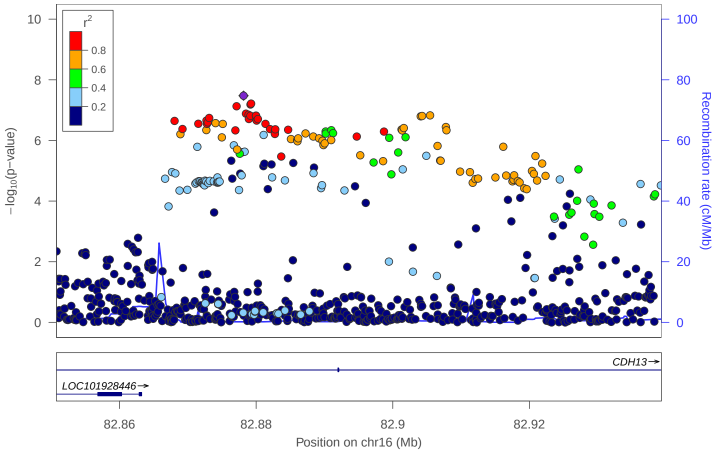

rs4791774

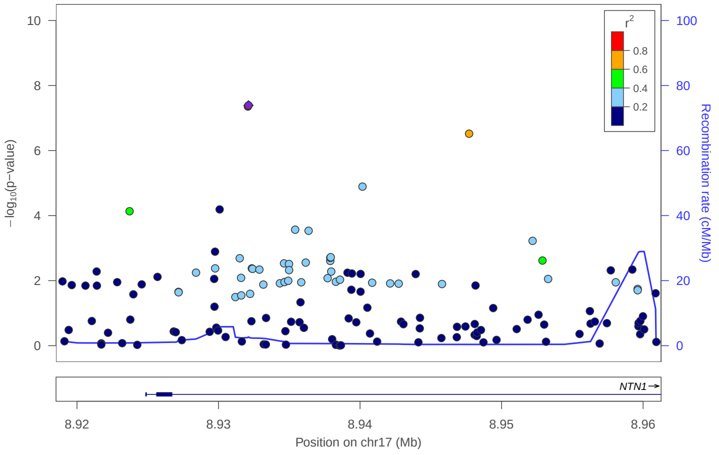

rs55657917

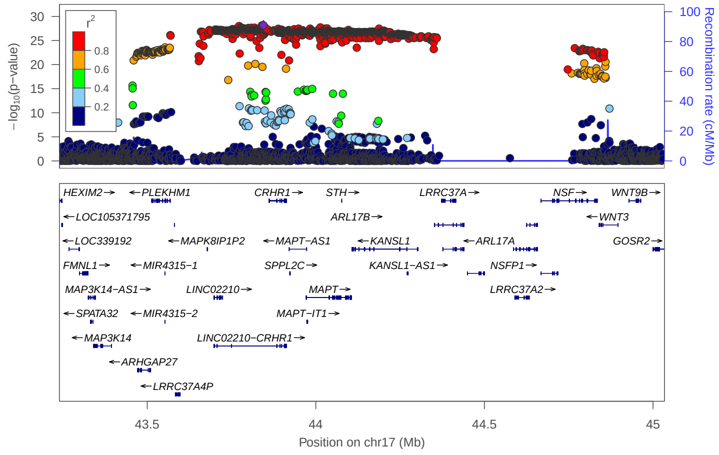

rs11663050

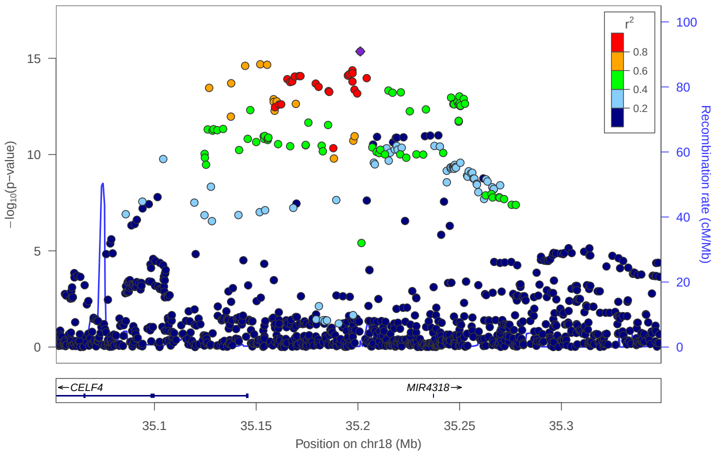

rs545853

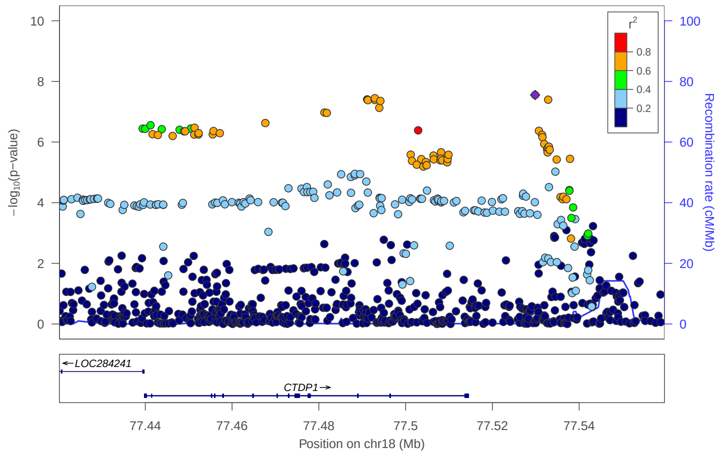

rs1389993

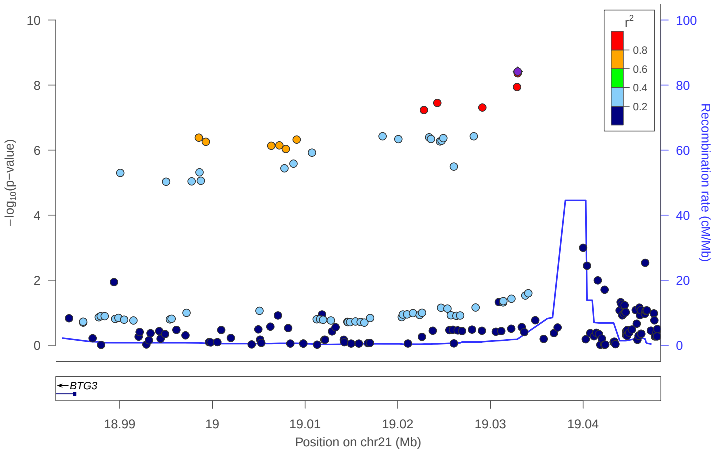

Supplementary Figure 14. Regional plots of GWA analyses of item HURT.

The *y*-axis represents the negative logarithm (base 10) of the SNP *P* value and the *x*-axis the position on the chromosome, with the name and location of genes in the UCSC Genome Browser in the bottom panel. The SNP with the lowest *P* value in the region is marked by a purple diamond. The colors of the other SNPs indicate the r^2 of these SNPs with the lead SNP, with grey color indicating SNPs below the user-defined r^2. Plots are generated with LocusZoom. See **Supplementary Data 2** for an overview of all lead SNPs for which a regional plot is presented above.

rs1983614

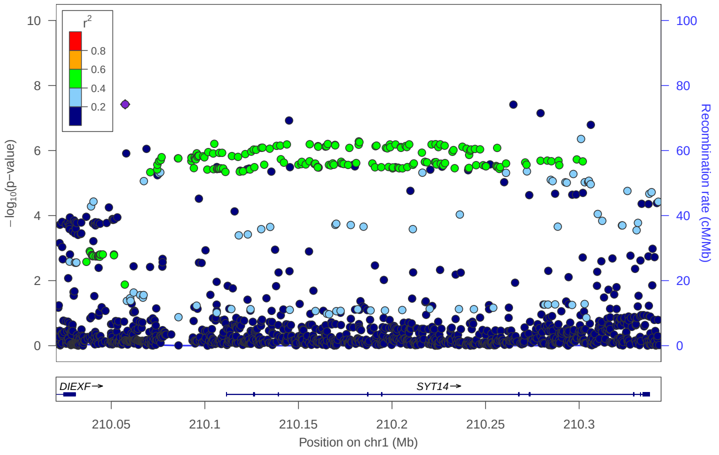

rs9811585

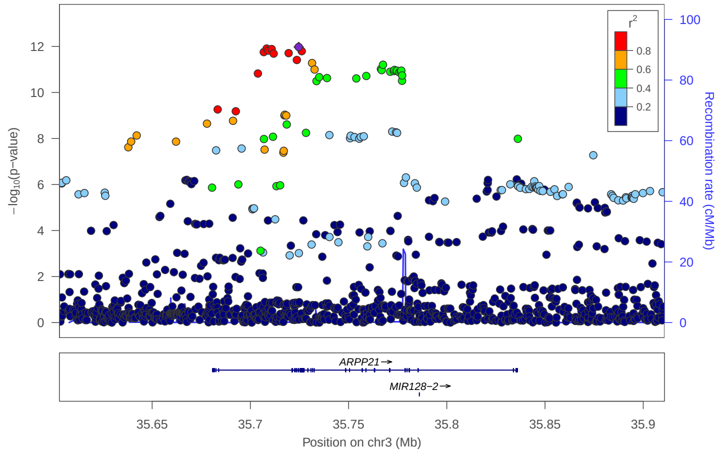

rs793545

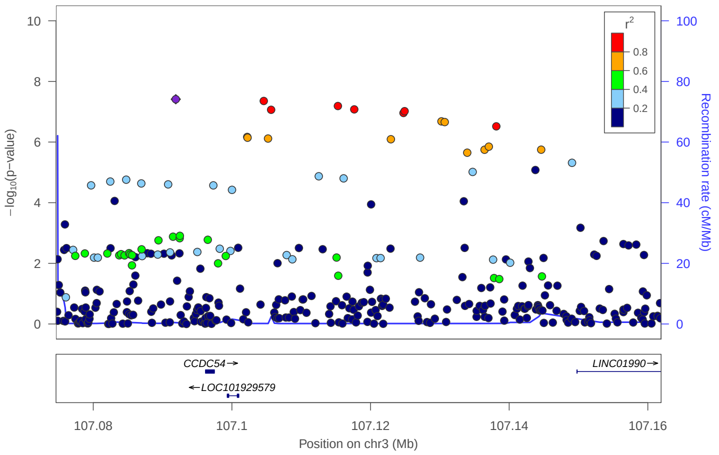

rs6439649

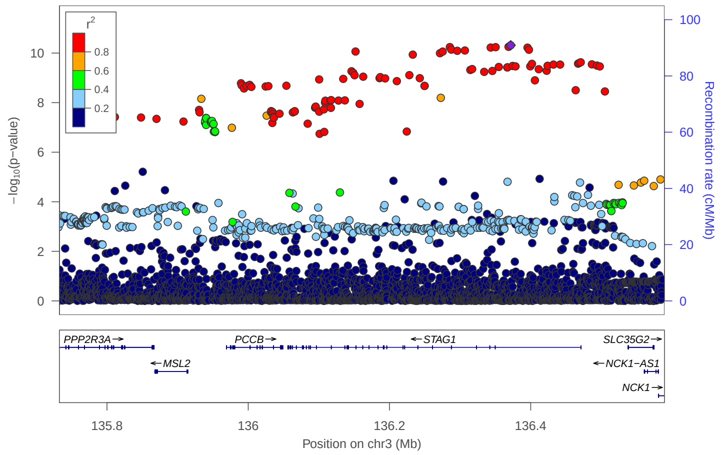

rs362307

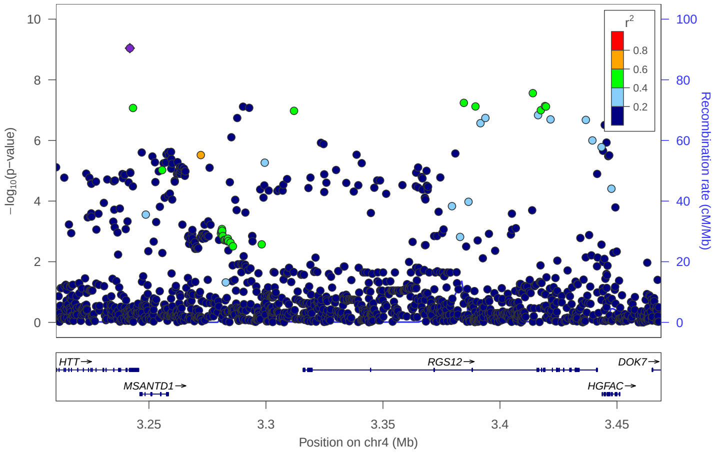

rs35267052

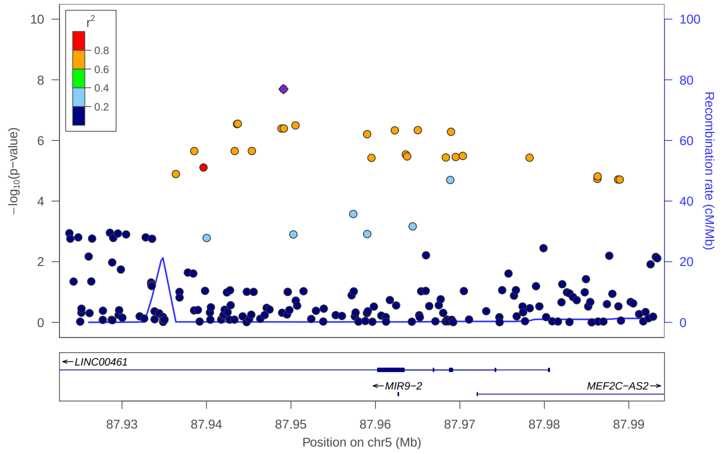

rs406204

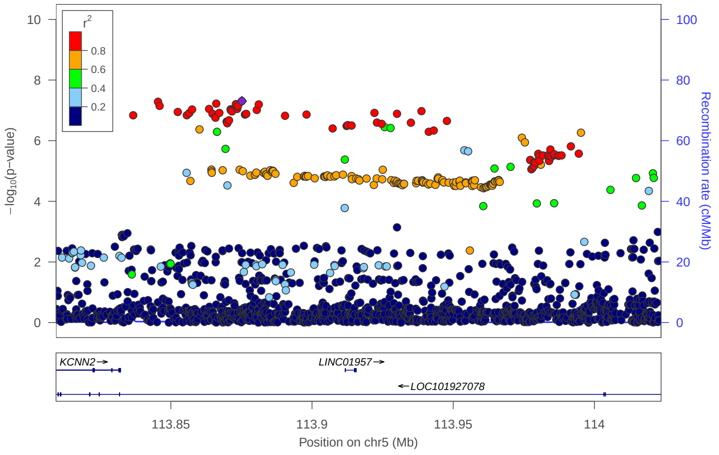

rs3777095

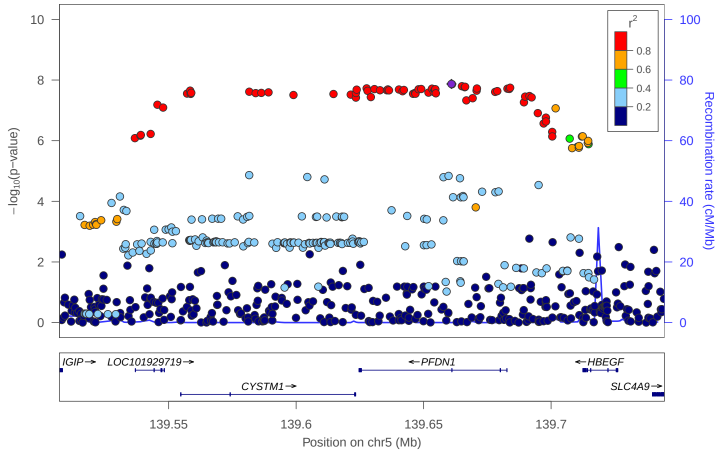

rs3999543

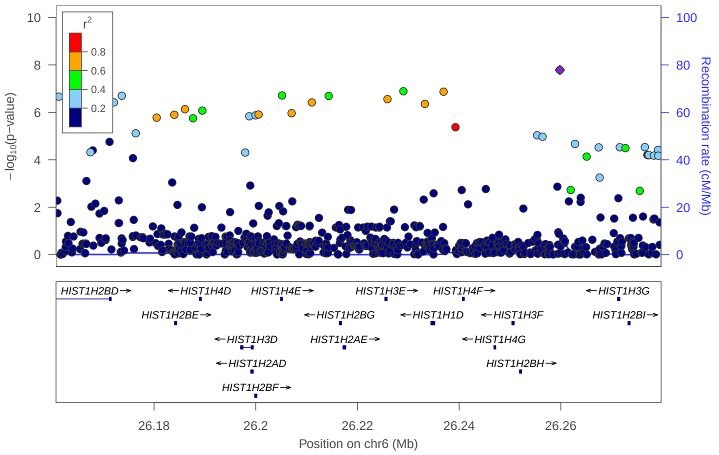

rs727903

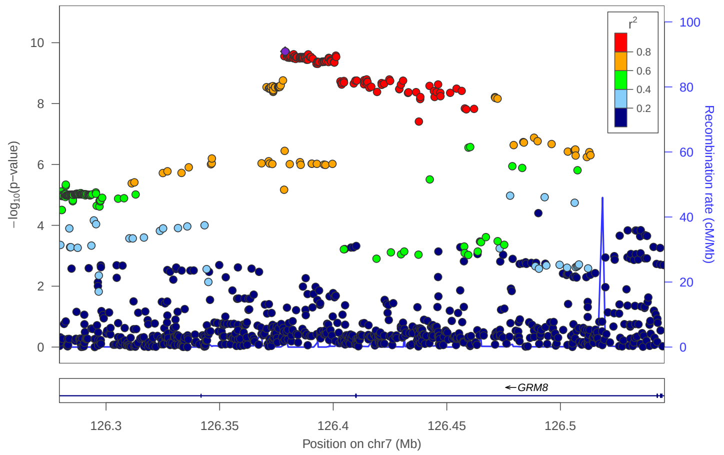

rs72765272

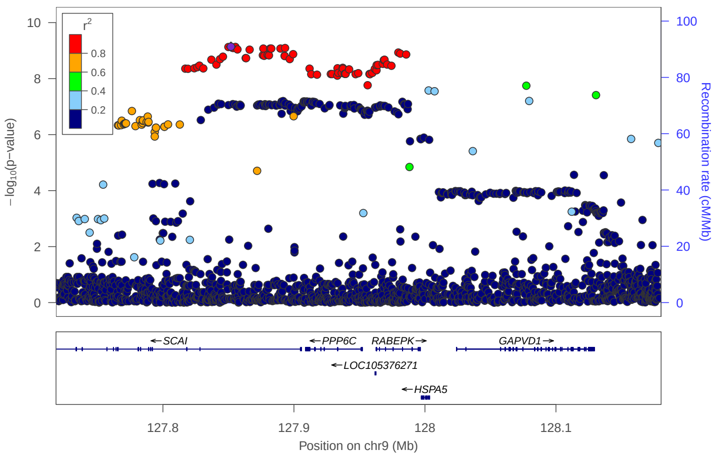

rs34588274

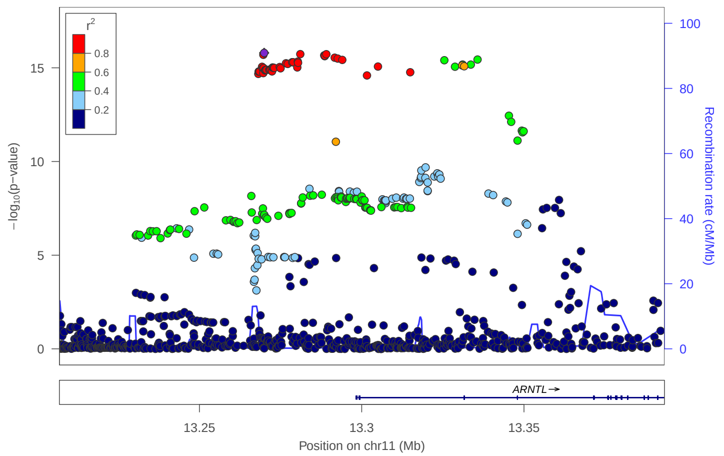

rs10750866

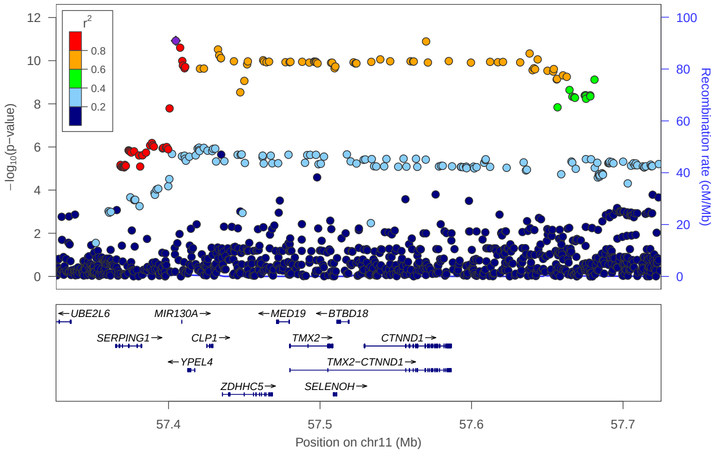

rs2734837

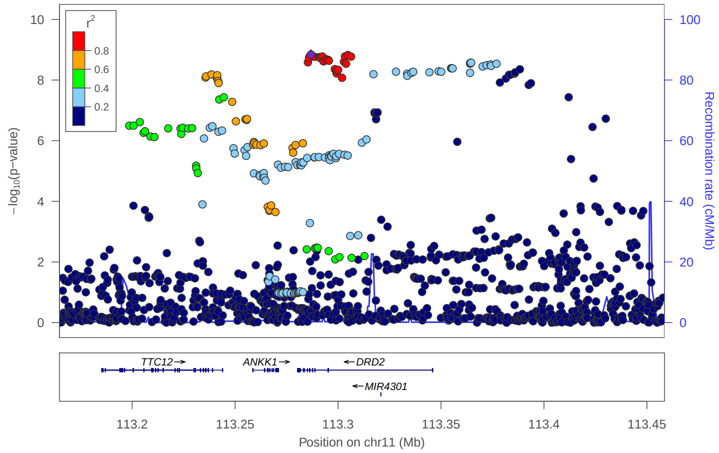

rs2160515

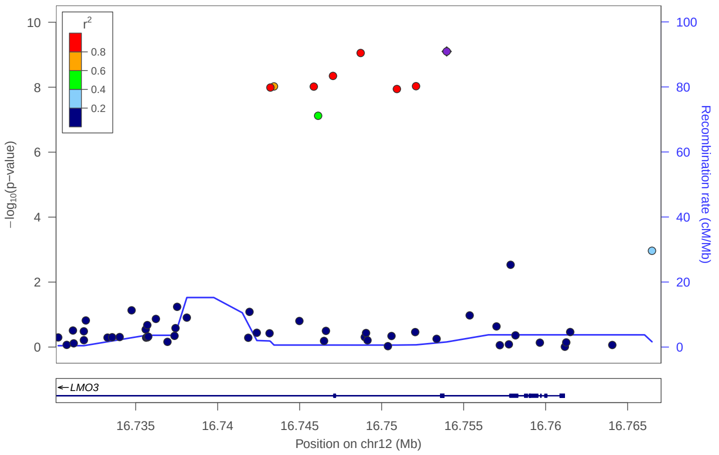

rs3742021

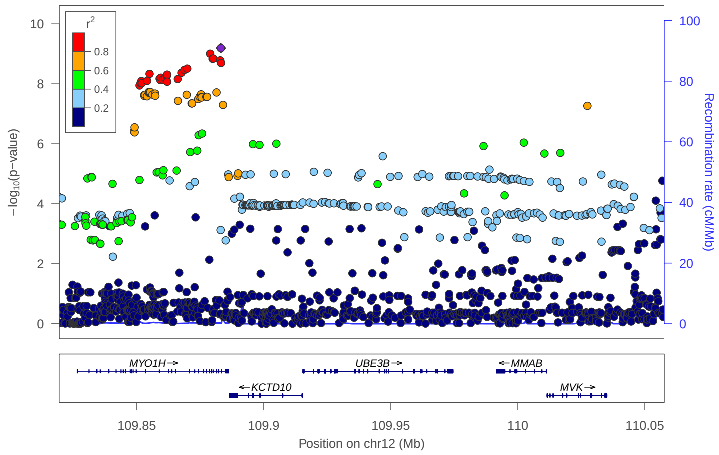

rs7987467

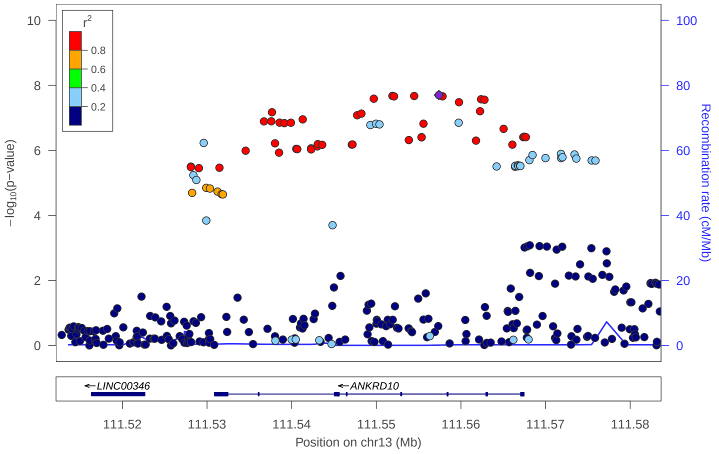

rs2191130

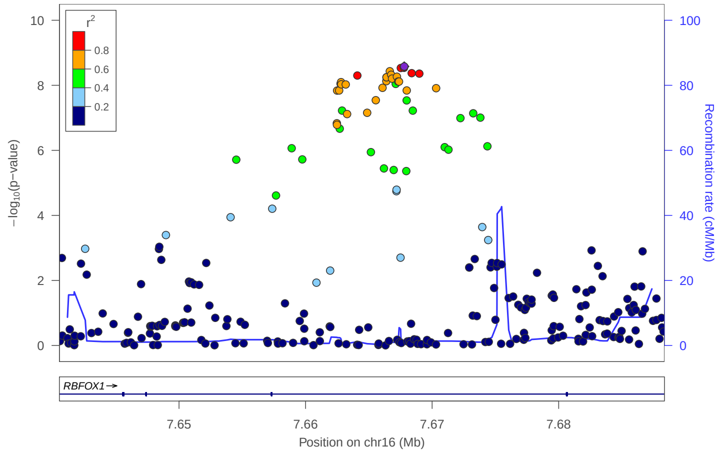

rs75022332

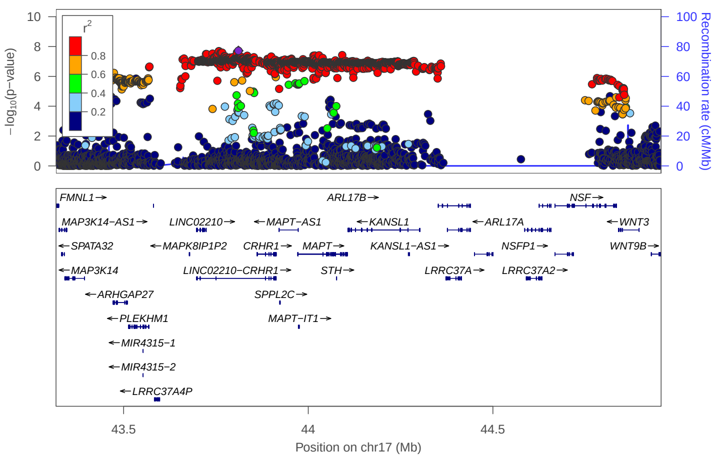

rs35327499

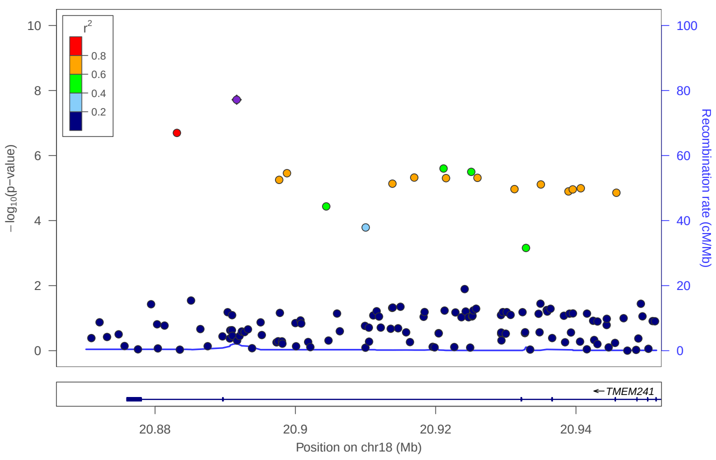

rs55731231

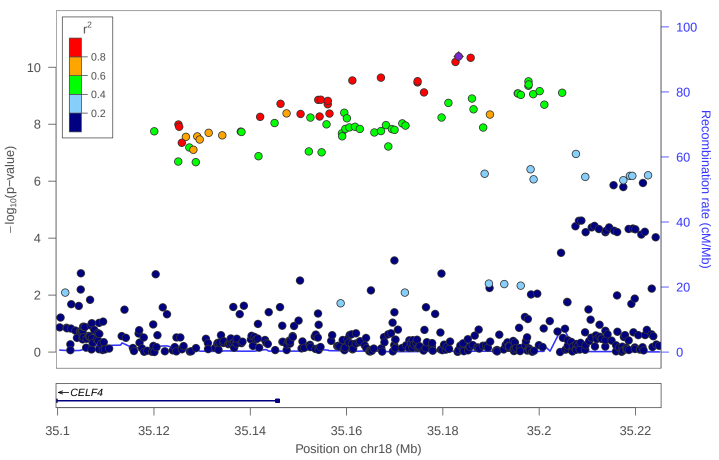

Supplementary Figure 15. Regional plots of GWA analyses of item WORR-EMB.
The *y*-axis represents the negative logarithm (base 10) of the SNP *P* value and the *x*-axis the position on the chromosome, with the name and location of genes in the UCSC Genome Browser in the bottom panel. The SNP with the lowest *P* value in the region is marked by a purple diamond. The colors of the other SNPs indicate the r^2 of these SNPs with the lead SNP, with grey color indicating SNPs below the user-defined r^2. Plots are generated with LocusZoom. See **Supplementary Data 2** for an overview of all lead SNPs for which a regional plot is presented above.

rs681875

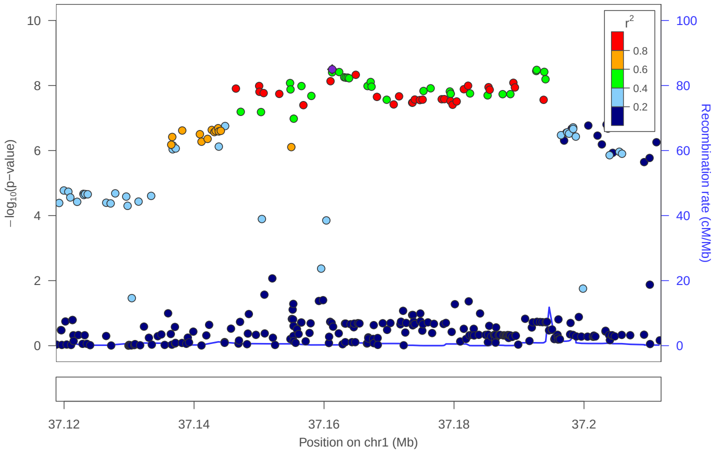

rs34657012

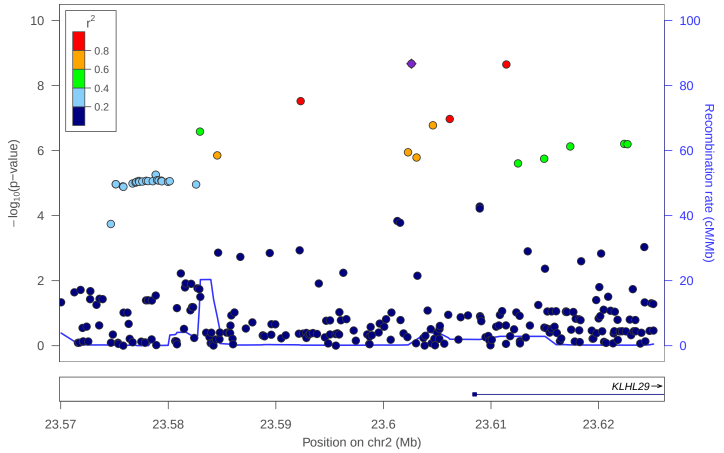

rs56343114

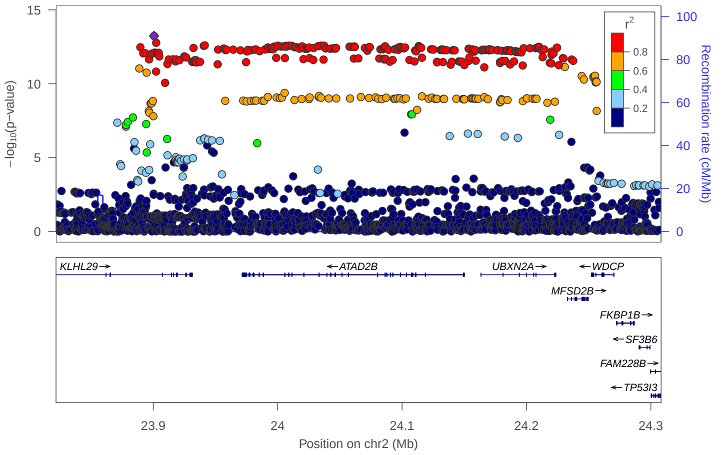

rs780024

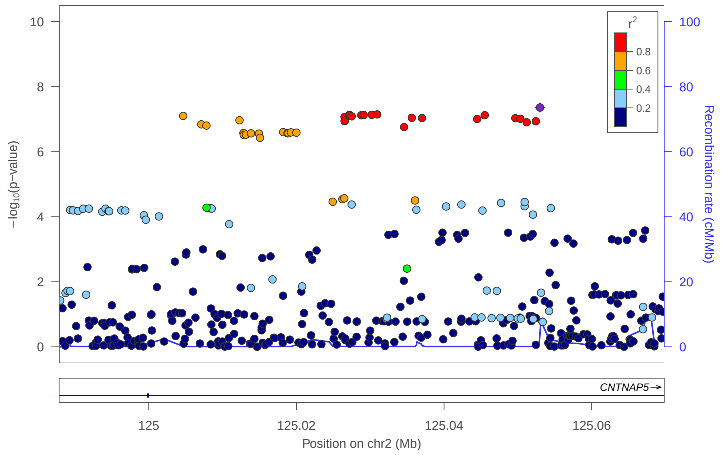

rs200988

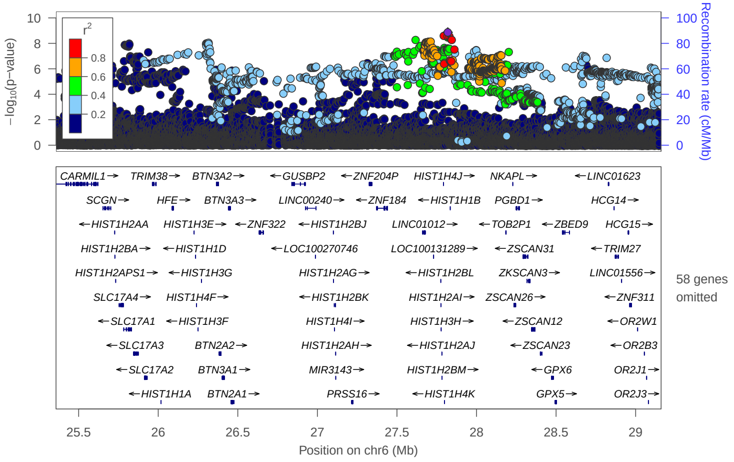

rs6986

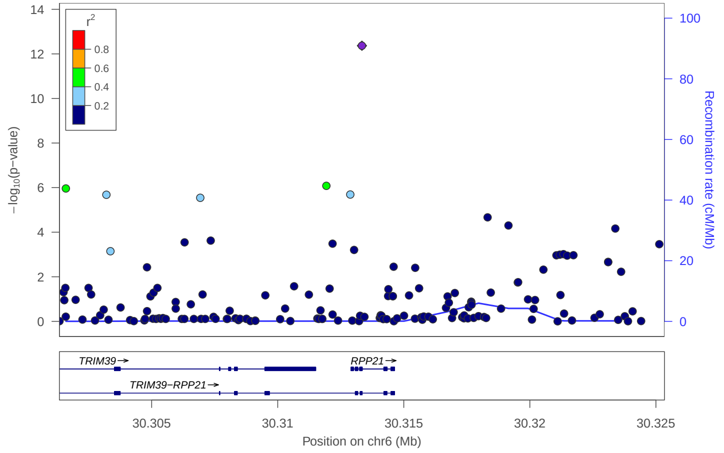

rs12528131

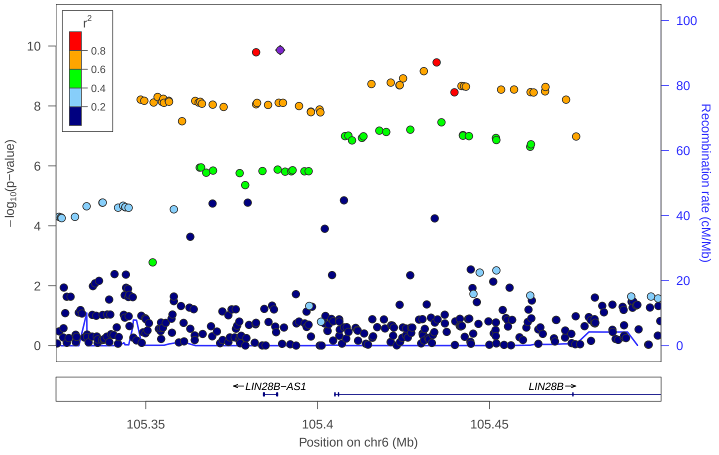

rs10119773

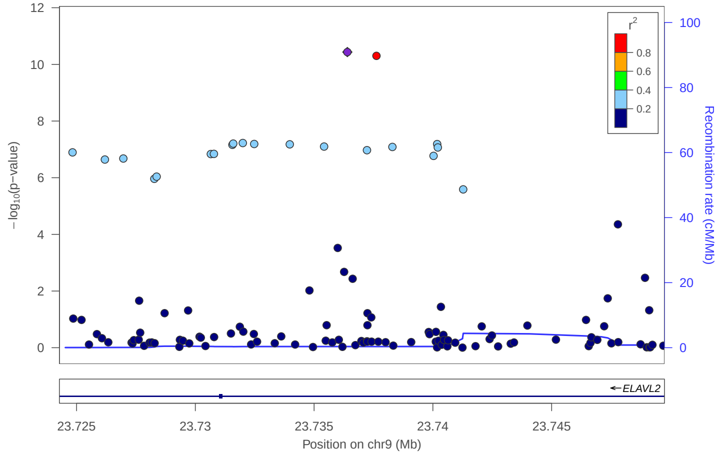

rs35623509

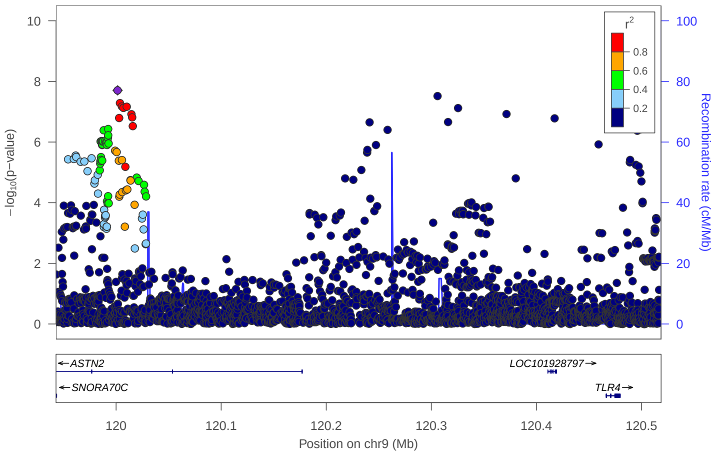

rs55769038

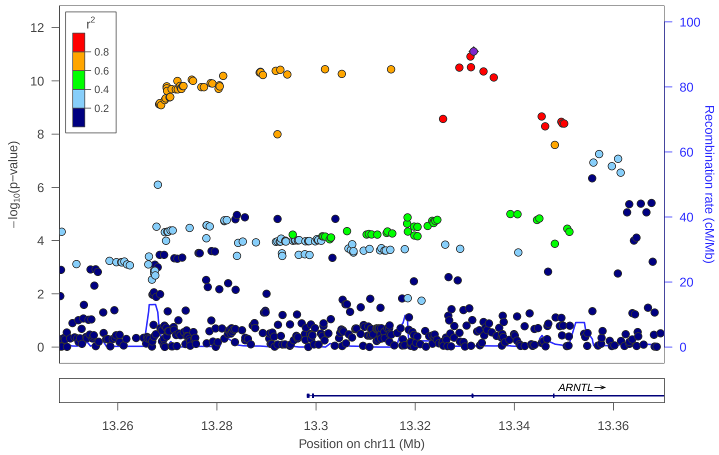

rs12420205

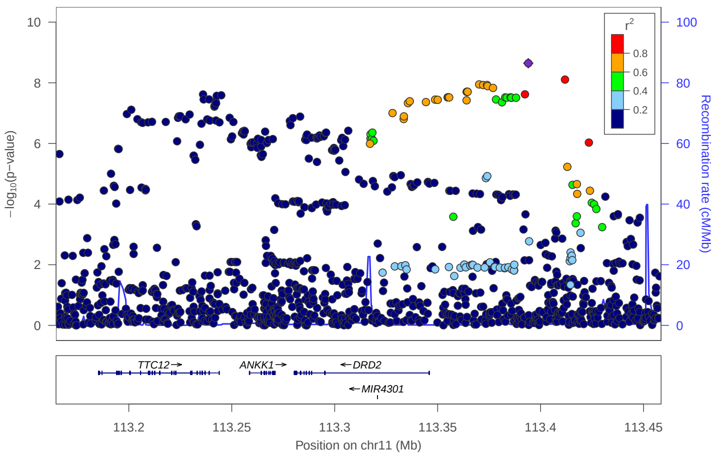

rs77804065

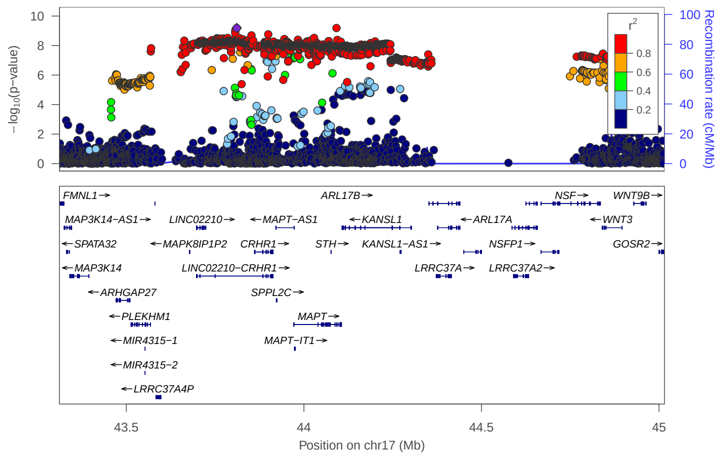

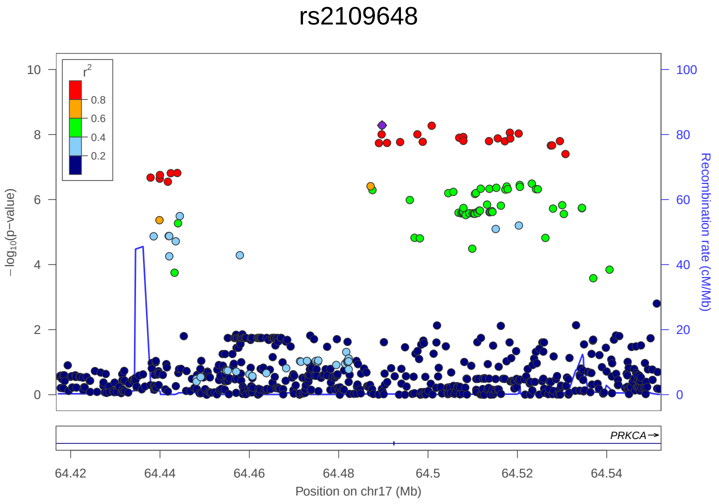
rs2109648
r²
0.8
0.6
0.4
0.2
10
8
6
4
2
0
−log10(p−value)
100
80
60
40
20
0
Recombination rate (cM/Mb)
PRKCA→
64.42
64.44
64.46
64.48
64.5
64.52
64.54
Position on chr17 (Mb)

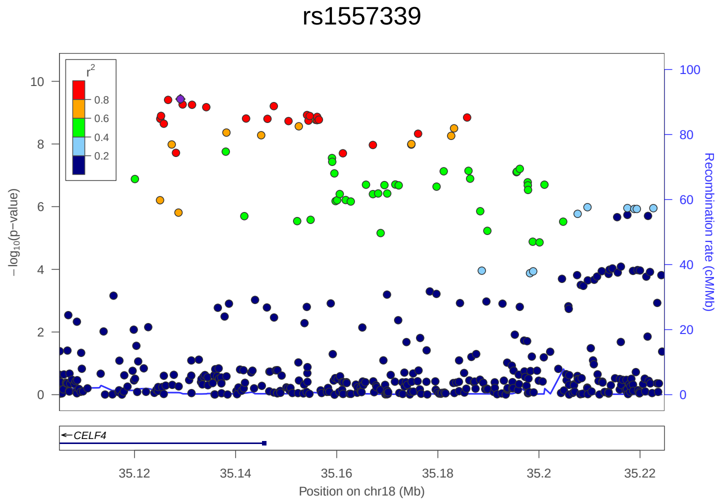
rs1557339
r²
0.8
0.6
0.4
0.2
10
8
6
4
2
0
−log10(p−value)
100
80
60
40
20
0
Recombination rate (cM/Mb)
←CELF4
35.12
35.14
35.16
35.18
35.2
35.22
Position on chr18 (Mb)

Supplementary Figure 16. Regional plots of GWA analyses of item GUILT.
The *y*-axis represents the negative logarithm (base 10) of the SNP *P* value and the *x*-axis the position on the chromosome, with the name and location of genes in the UCSC Genome Browser in the bottom panel. The SNP with the lowest *P* value in the region is marked by a purple diamond. The colors of the other SNPs indicate the r^2 of these SNPs with the lead SNP, with grey color indicating SNPs below the user-defined r^2. Plots are generated with LocusZoom. See **Supplementary Data 2** for an overview of all lead SNPs for which a regional plot is presented above.

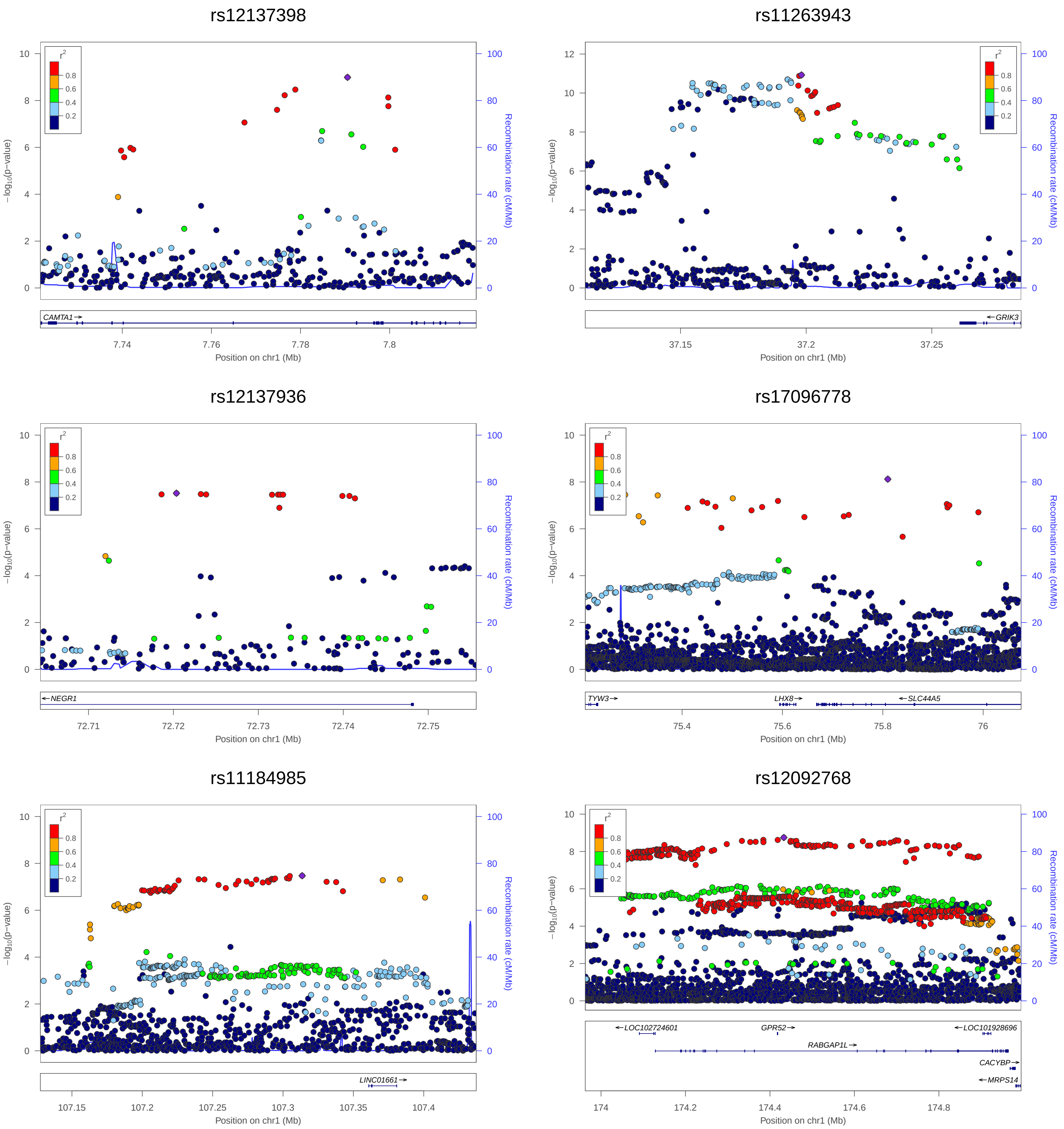
rs12137398
CAMTA1
Position on chr1 (Mb)
rs11263943
GRIK3
Position on chr1 (Mb)
rs12137936
NEGR1
Position on chr1 (Mb)
rs17096778
TYW3
LHX8
SLC44A5
Position on chr1 (Mb)
rs11184985
LINC01661
Position on chr1 (Mb)
rs12092768
LOC102724601
GPR52
LOC101928696
RABGAP1L
CACYBP
MRPS14
Position on chr1 (Mb)
−log10(p−value)
Recombination rate (cM/Mb)
r2

rs2488401

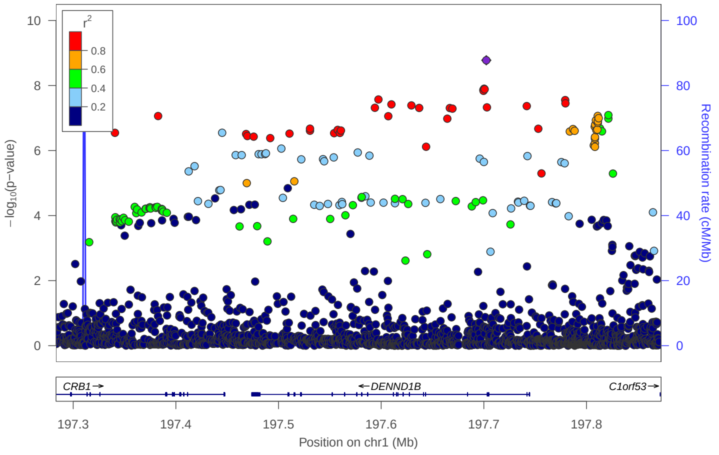

rs17432675

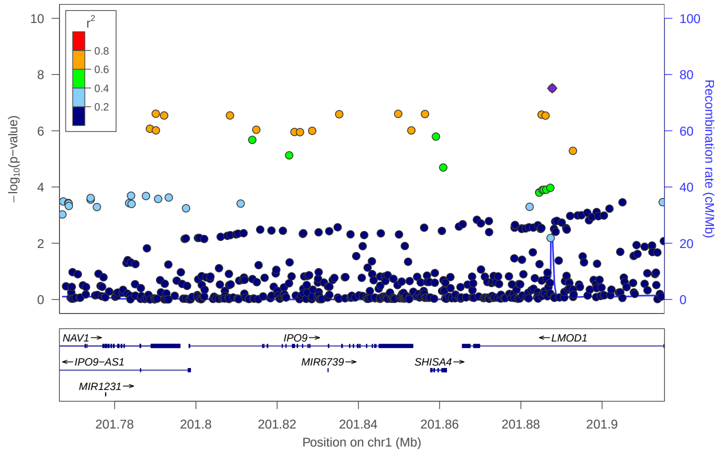

rs4396680

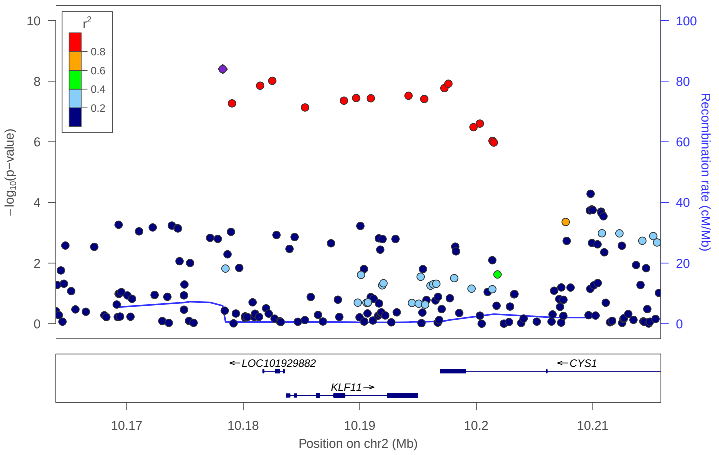

rs2717043

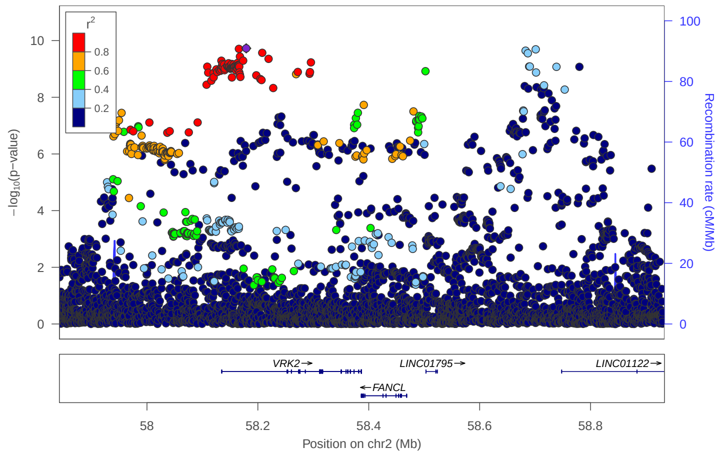

rs17619012

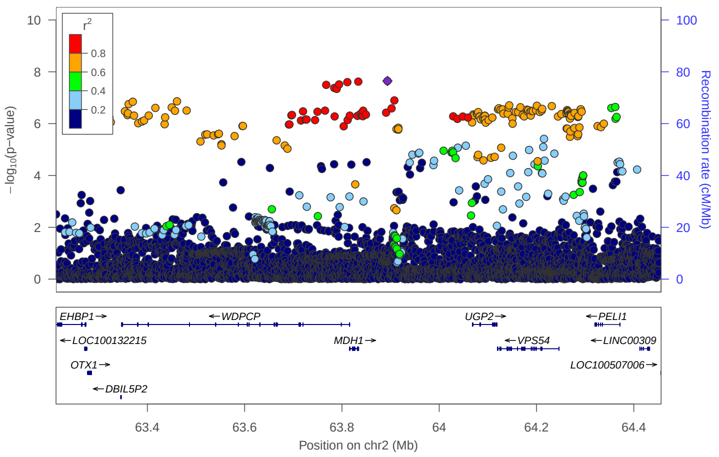

rs998884

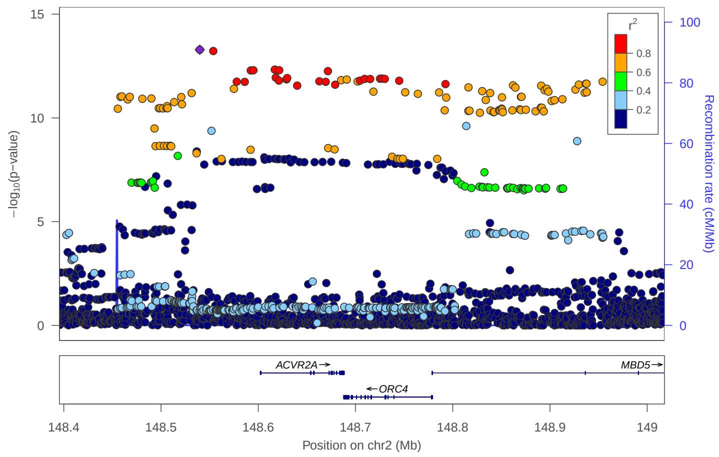

rs59491086

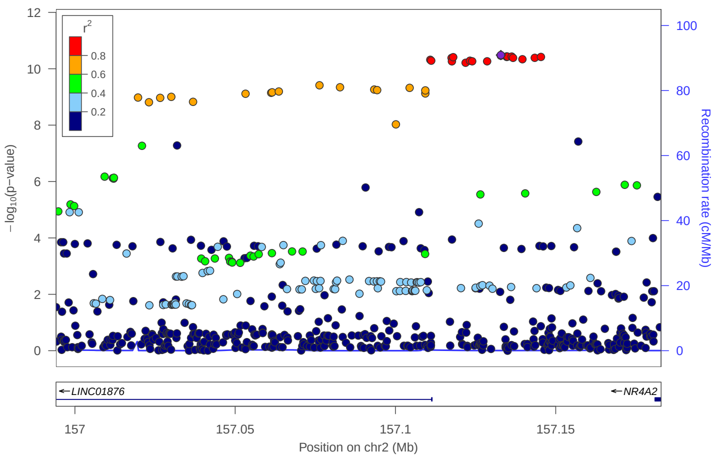

rs10497655

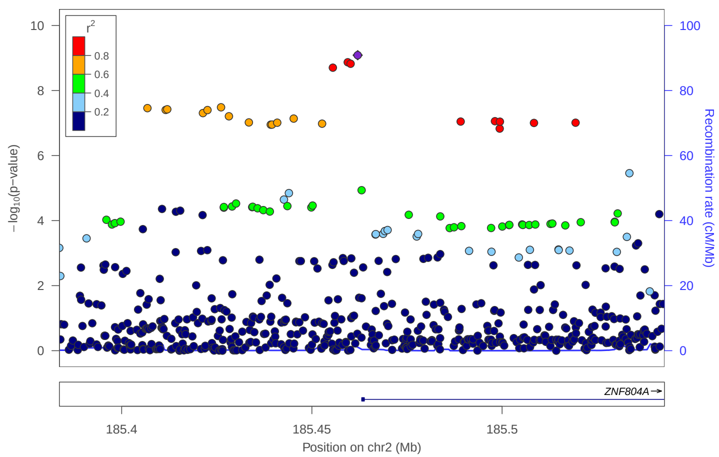

rs988867

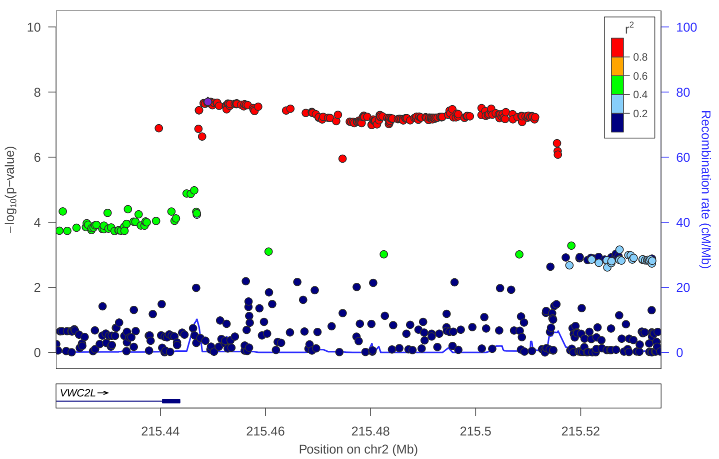

rs6737187

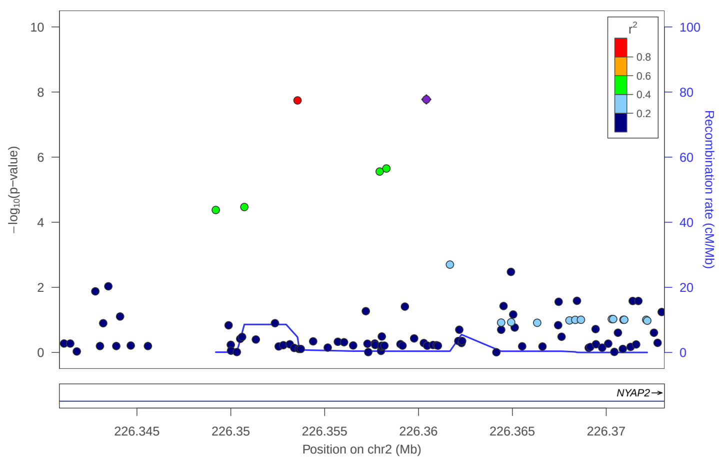

rs6773869

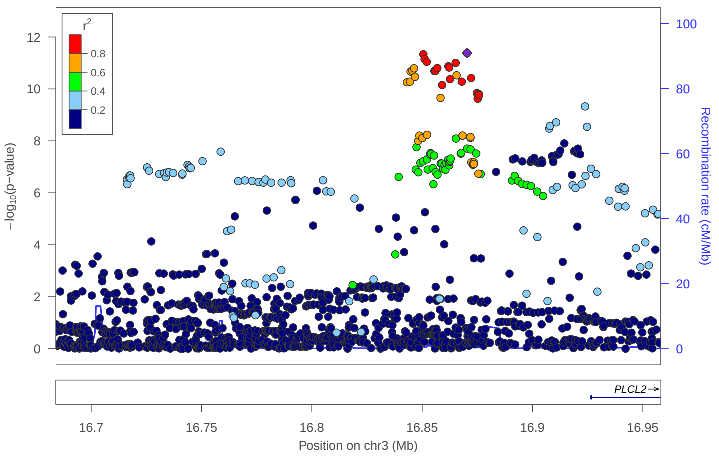

rs1542212

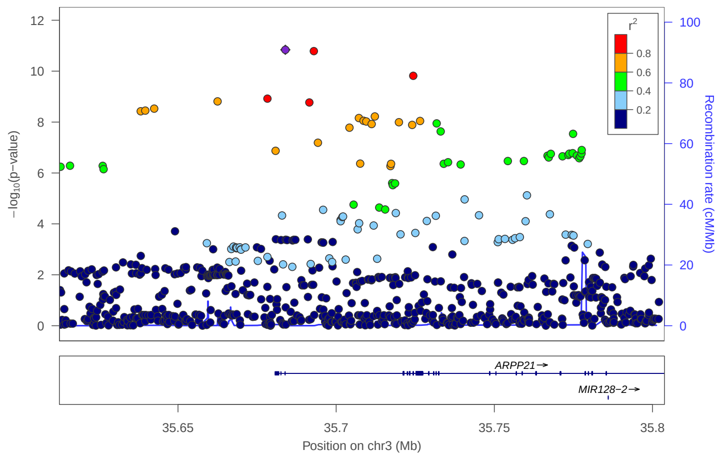

rs4405857

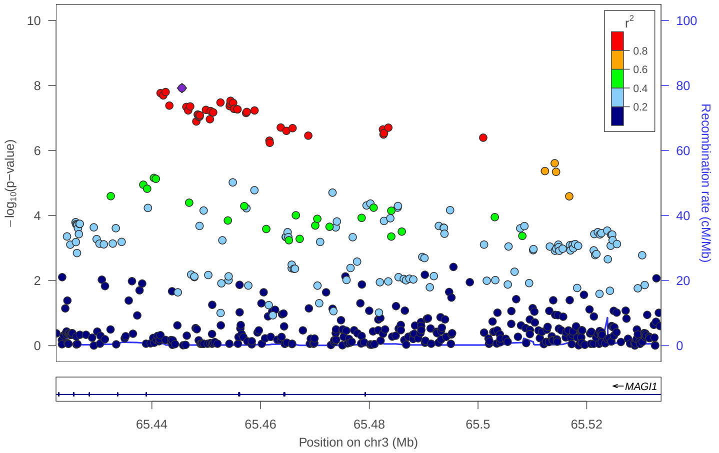

rs1282545

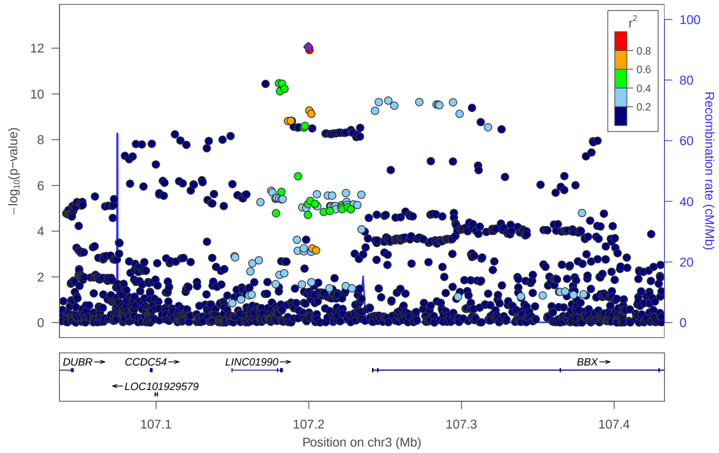

rs10935181

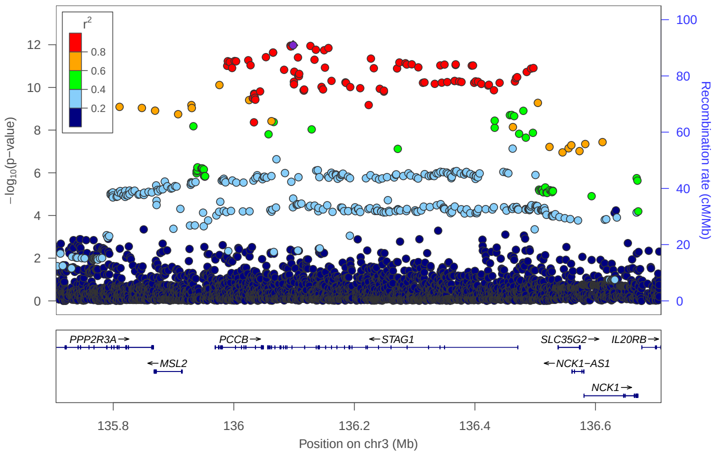

rs4585149

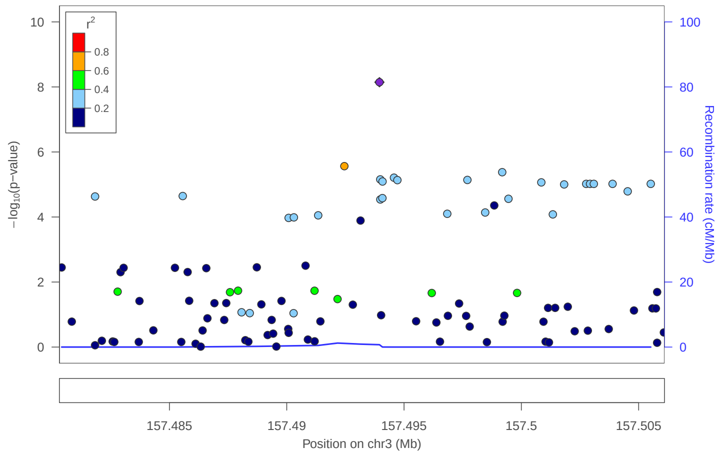

rs1714510

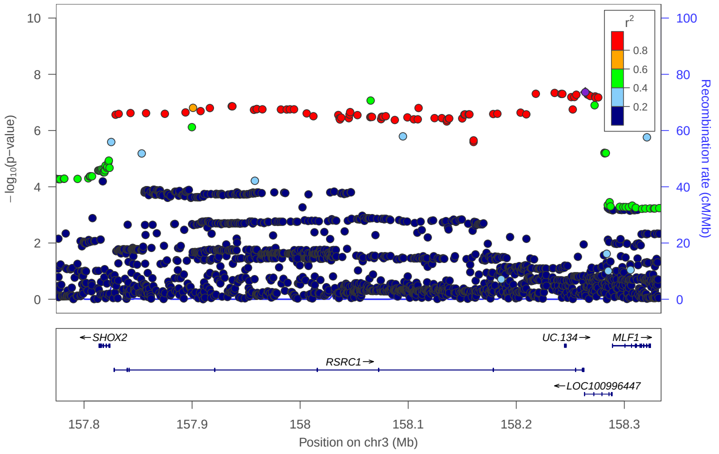

rs16854051

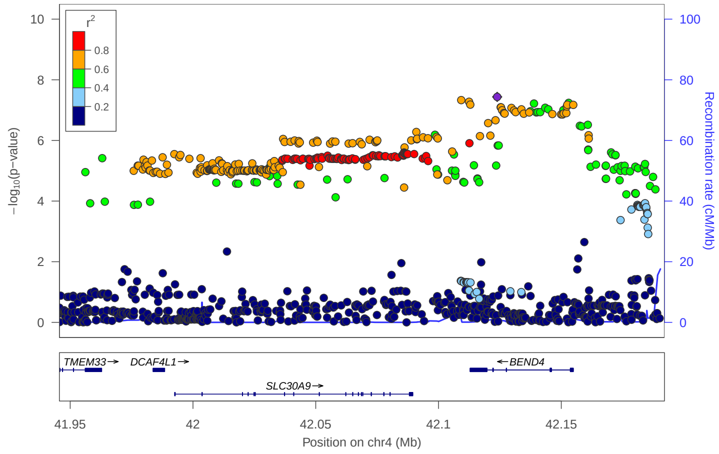

rs10005233

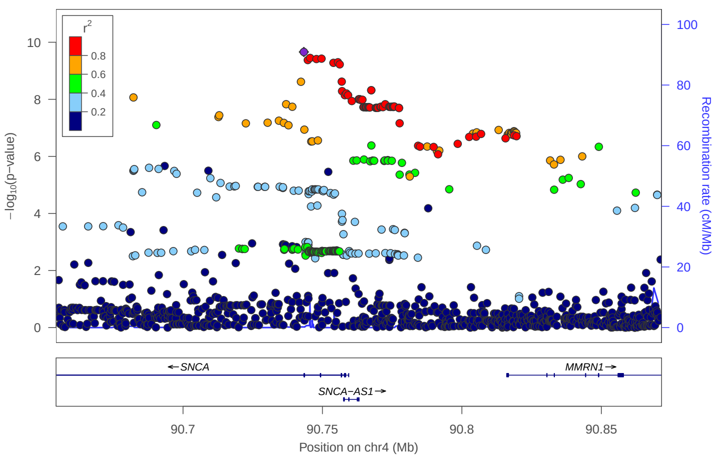

rs4530683

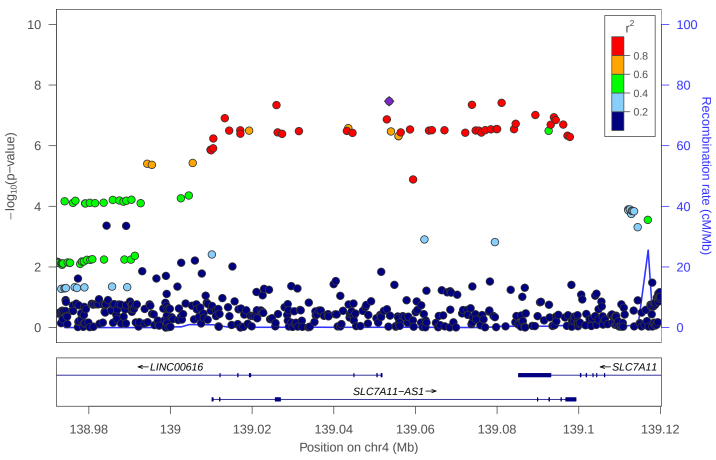

rs10054419

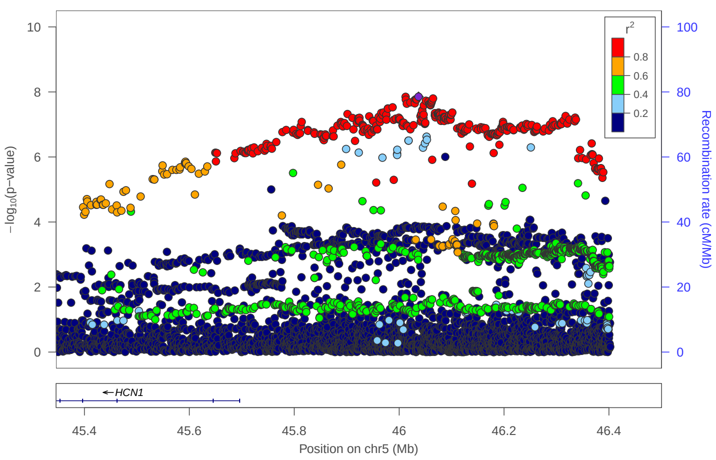

rs1422192

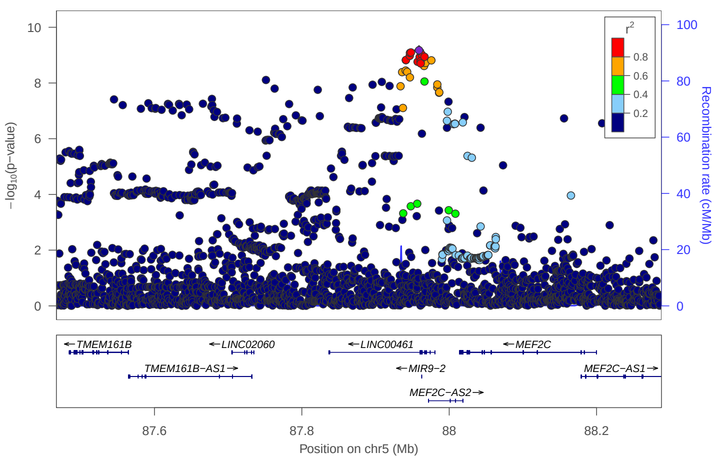

rs11948261

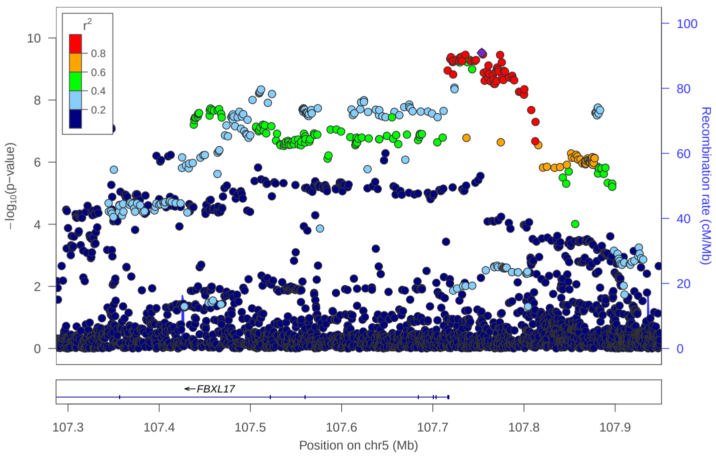

rs58007470

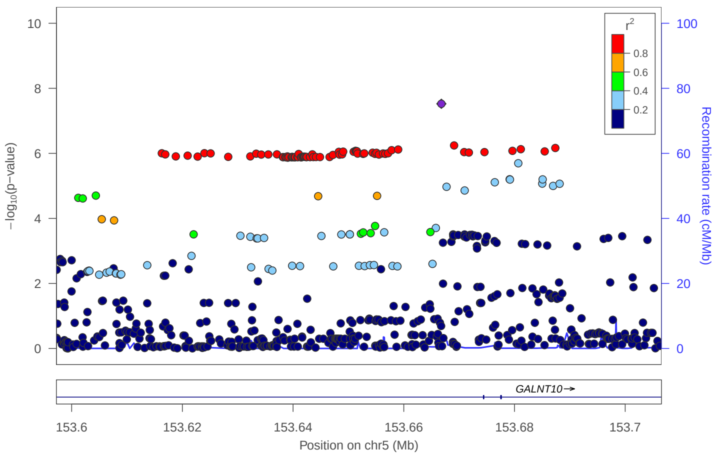

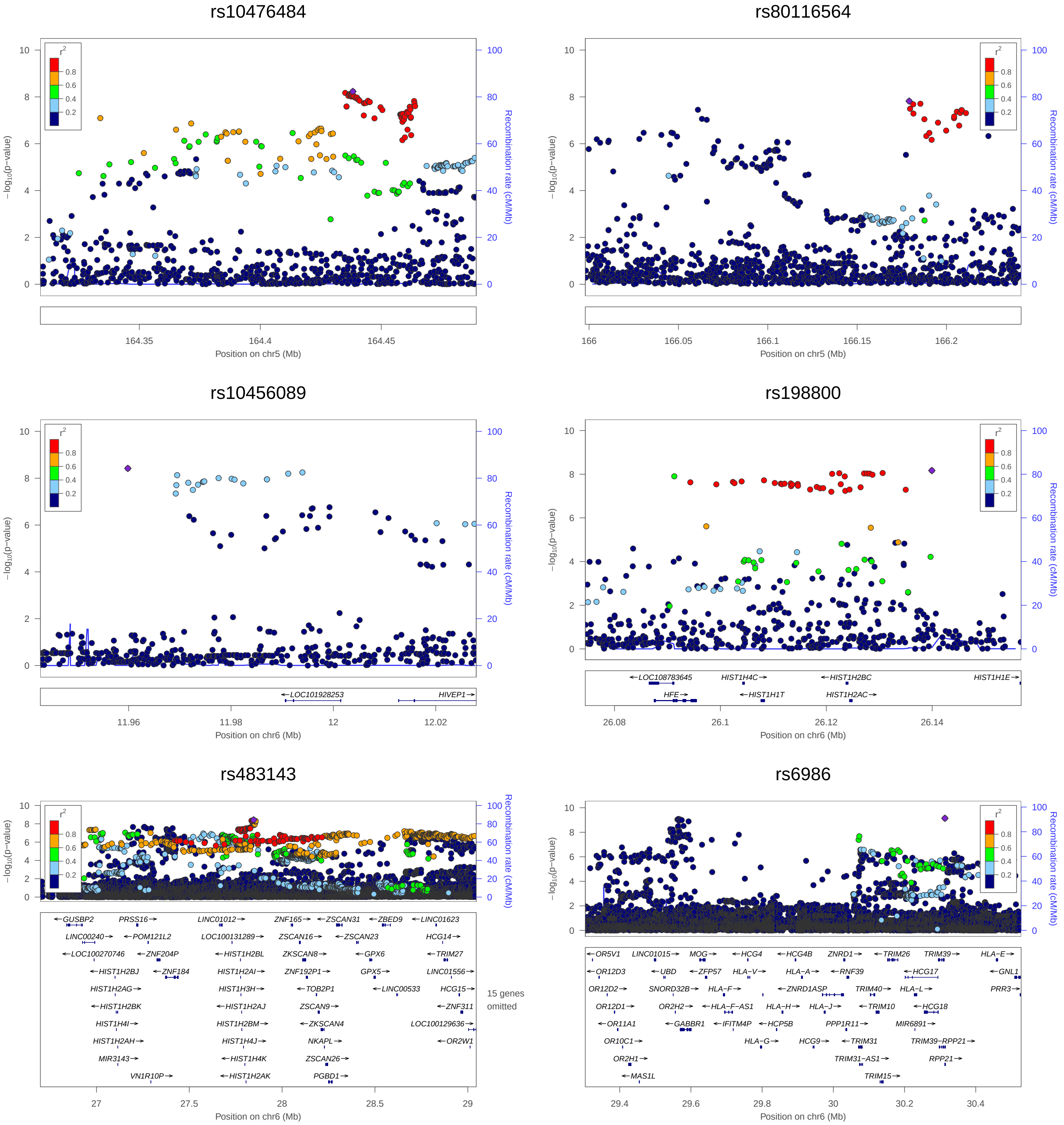

rs10476484
rs80116564
rs10456089
rs198800
rs483143
rs6986
−log10(p−value)
Recombination rate (cM/Mb)
r2
0.8
0.6
0.4
0.2
Position on chr5 (Mb)
Position on chr6 (Mb)
164.35
164.4
164.45
166
166.05
166.1
166.15
166.2
11.96
11.98
12
12.02
←LOC101928253
HIVEP1→
26.08
26.1
26.12
26.14
←LOC108783645
HIST1H4C→
←HIST1H2BC
HIST1H1E→
HFE→
←HIST1H1T
HIST1H2AC→
27
27.5
28
28.5
29
←GUSBP2
PRSS16→
LINC01012→
ZNF165→
←ZSCAN31
←ZBED9
←LINC01623
LINC00240→
←POM121L2
LOC100131289→
ZSCAN16→
←ZSCAN23
HCG14→
←LOC100270746
←ZNF204P
←HIST1H2BL
ZKSCAN8→
←GPX6
←TRIM27
←HIST1H2BJ
←ZNF184
HIST1H2AI→
ZNF192P1→
GPX5→
LINC01556→
HIST1H2AG→
HIST1H3H→
←TOB2P1
←LINC00533
HCG15→
←HIST1H2BK
←HIST1H2AJ
ZSCAN9→
←ZNF311
HIST1H4I→
HIST1H2BM→
←ZKSCAN4
LOC100129636→
HIST1H2AH→
HIST1H4J→
NKAPL→
←OR2W1
MIR3143→
←HIST1H4K
ZSCAN26→
VN1R10P→
←HIST1H2AK
PGBD1→
15 genes omitted
29.4
29.6
29.8
30
30.2
30.4
←OR5V1
LINC01015→
MOG→
←HCG4
←HCG4B
ZNRD1→
←TRIM26
TRIM39→
HLA−E→
←OR12D3
←UBD
←ZFP57
HLA−V→
HLA−A→
←RNF39
←HCG17
←GNL1
OR12D2→
SNORD32B→
HLA−F→
←ZNRD1ASP
TRIM40→
HLA−L→
PRR3→
OR12D1→
OR2H2→
←HLA−F−AS1
HLA−H→
HLA−J→
←TRIM10
←HCG18
←OR11A1
←GABBR1
←IFITM4P
←HCP5B
PPP1R11→
MIR6891→
OR10C1→
HLA−G→
HCG9→
←TRIM31
TRIM39−RPP21→
OR2H1→
TRIM31−AS1→
RPP21→
←MAS1L
TRIM15→

rs2269426

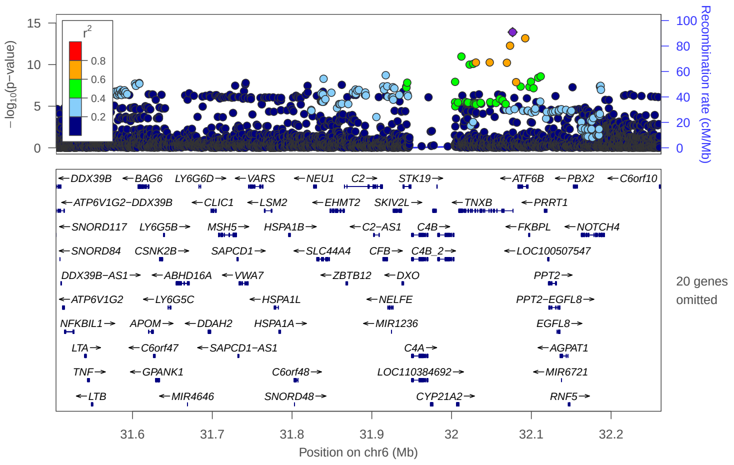

rs2503775

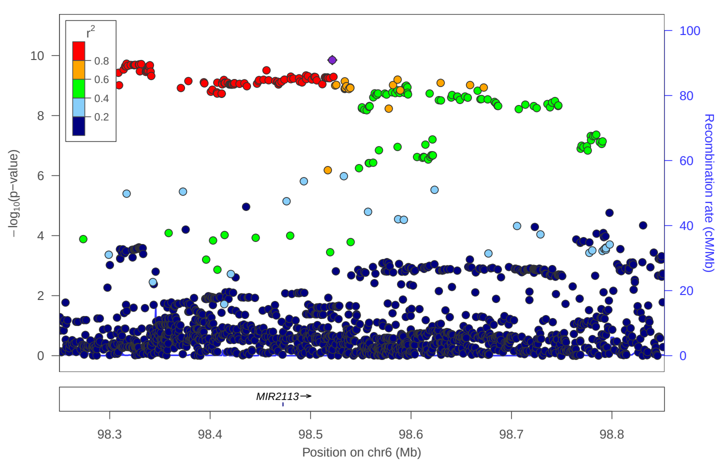

rs240769

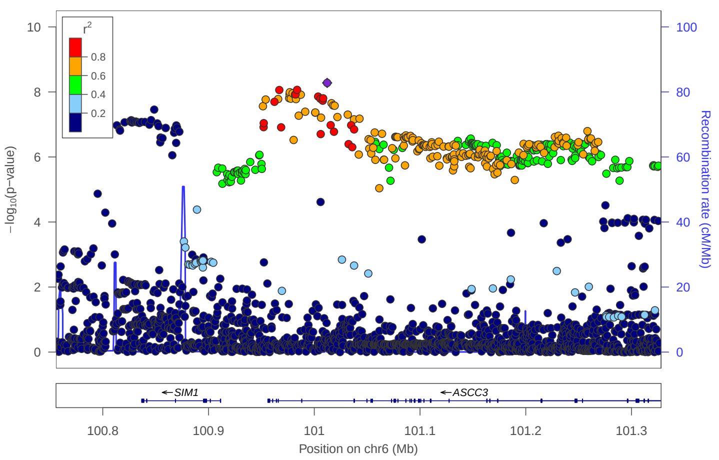

rs11759026

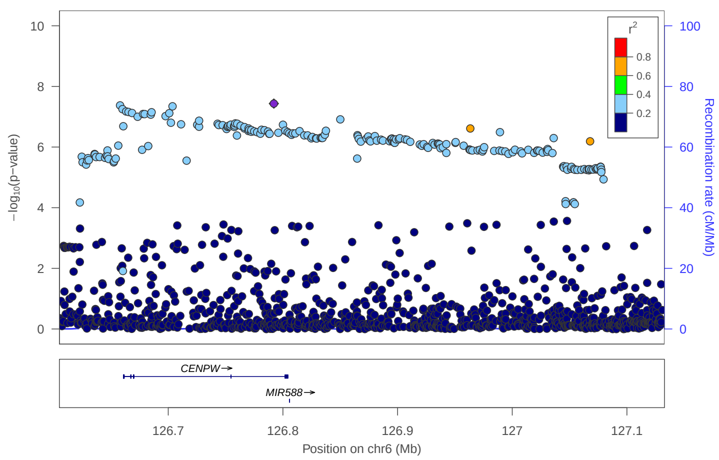

rs2056477

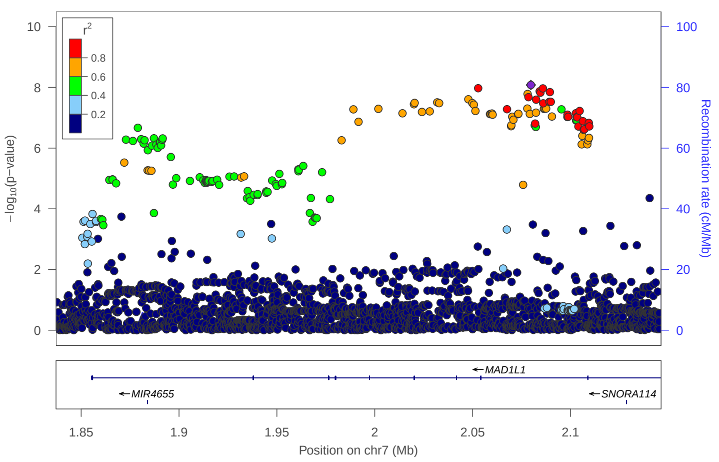

rs11509880

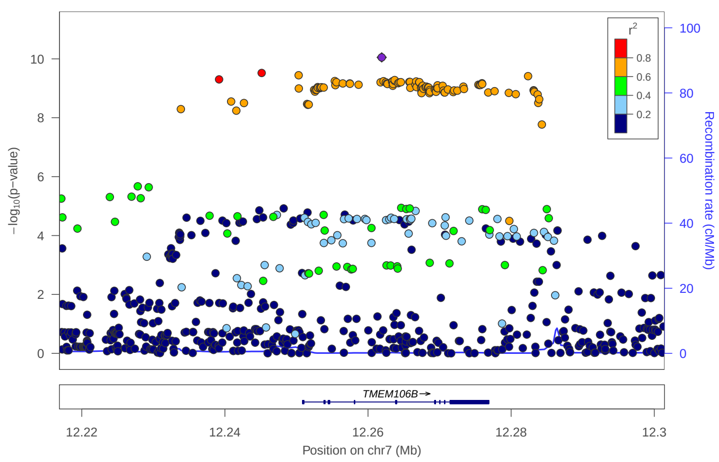

rs6970541

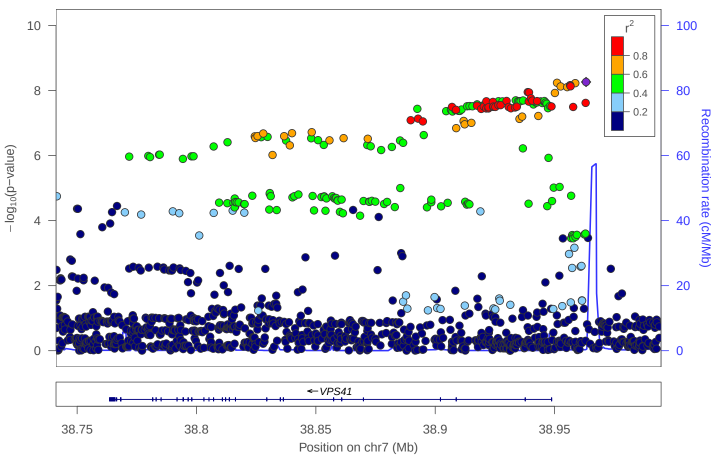

rs802425

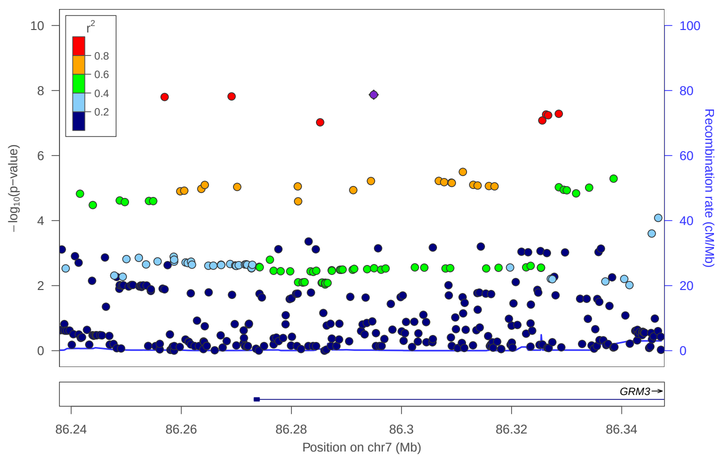

rs13226841

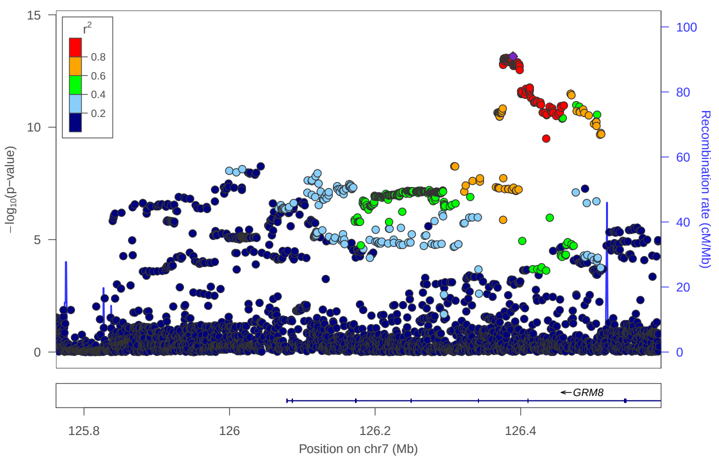

rs1731951

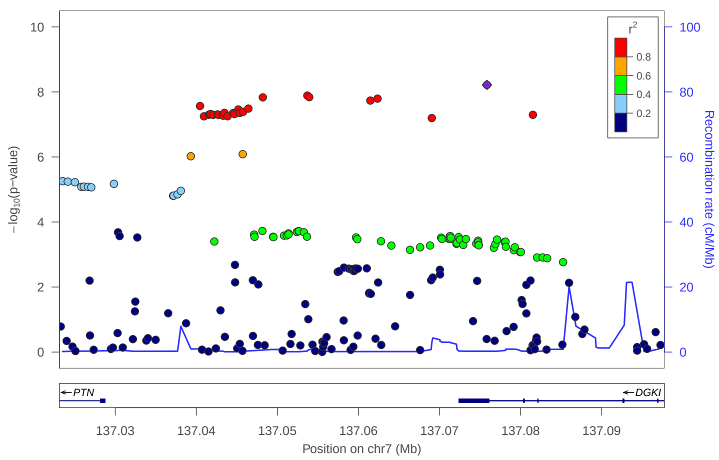

rs2407746

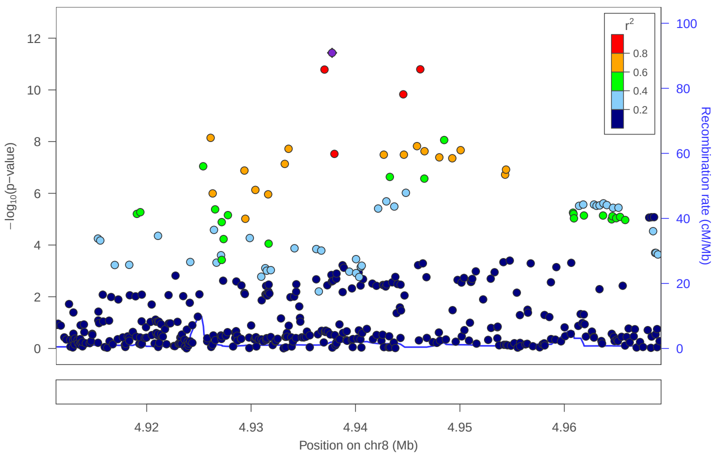

rs7818437

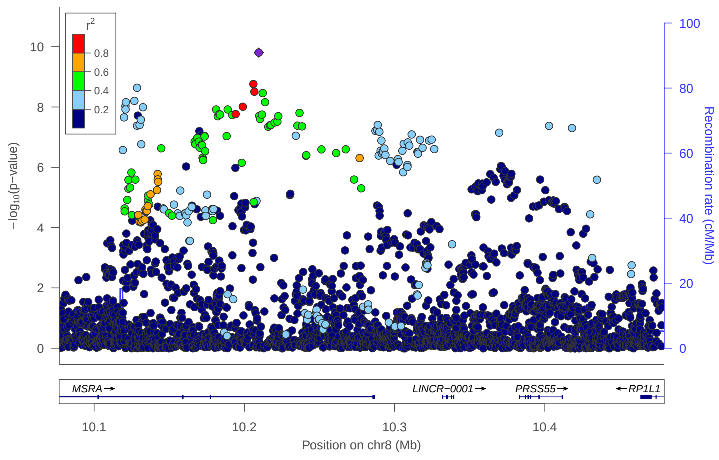

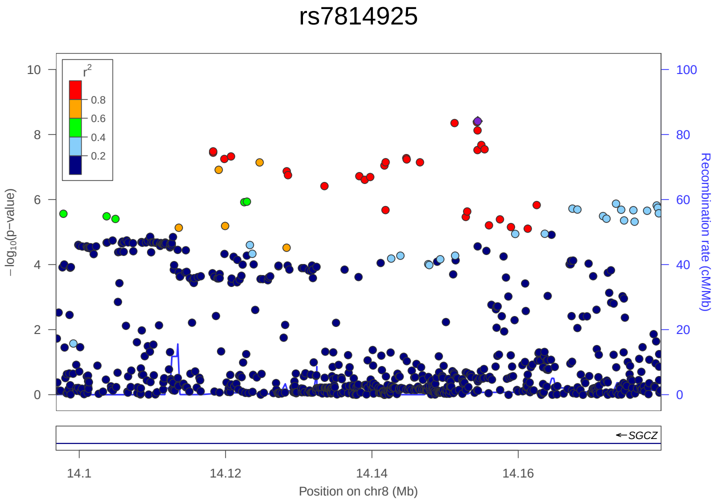
rs7814925
Position on chr8 (Mb)
−log10(p−value)
Recombination rate (cM/Mb)
SGCZ

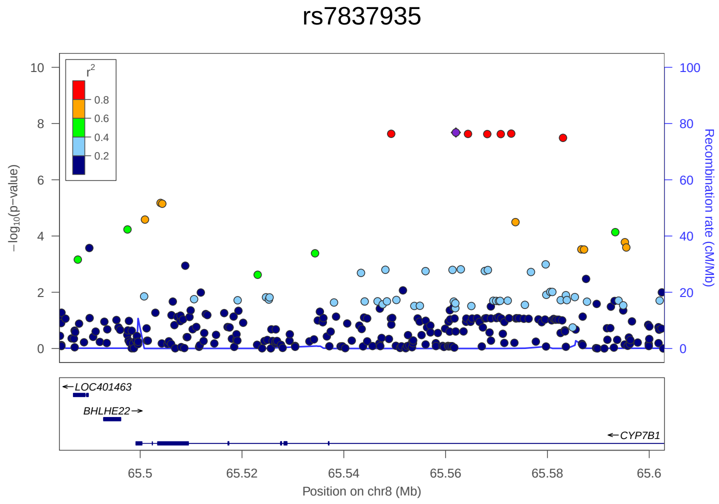
rs7837935
Position on chr8 (Mb)
−log10(p−value)
Recombination rate (cM/Mb)
LOC401463
BHLHE22
CYP7B1

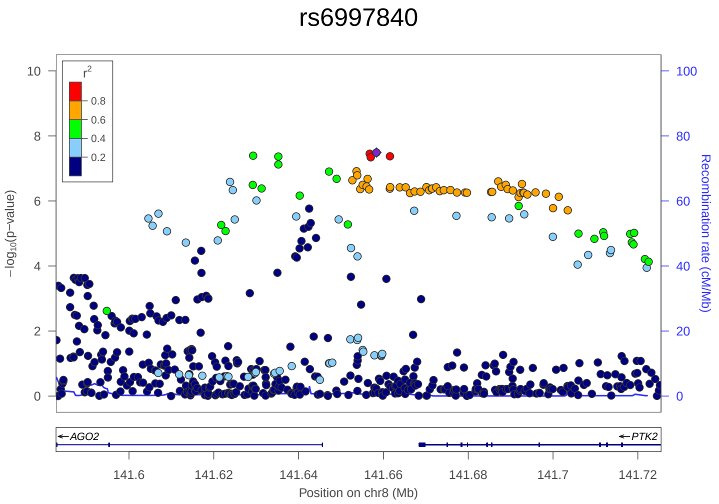
rs6997840
Position on chr8 (Mb)
−log10(p−value)
Recombination rate (cM/Mb)
AGO2
PTK2

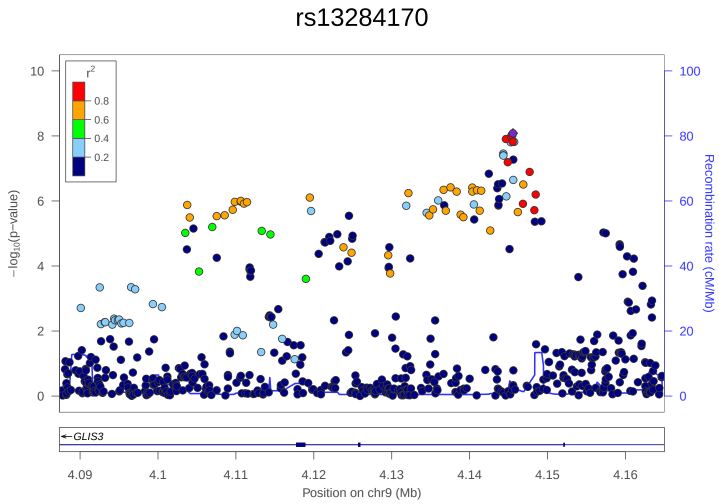
rs13284170
Position on chr9 (Mb)
−log10(p−value)
Recombination rate (cM/Mb)
GLIS3

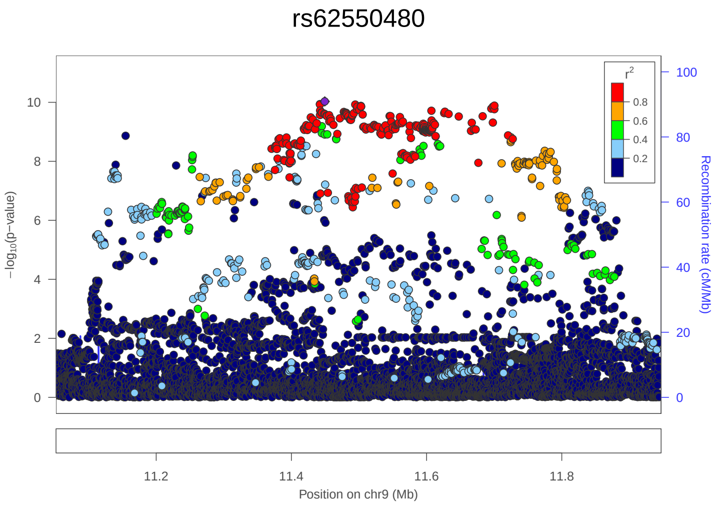
rs62550480
Position on chr9 (Mb)
−log10(p−value)
Recombination rate (cM/Mb)

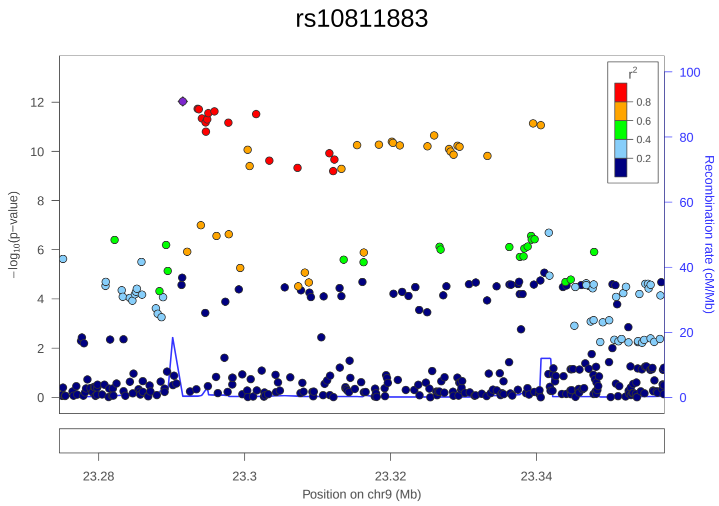
rs10811883
Position on chr9 (Mb)
−log10(p−value)
Recombination rate (cM/Mb)

rs10119773

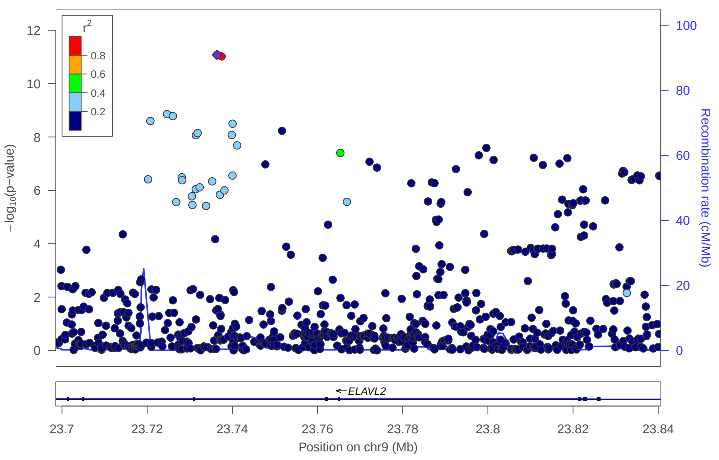

rs7033345

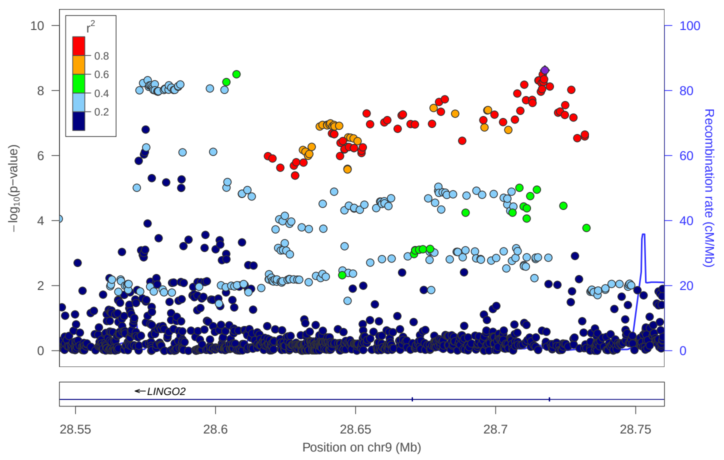

rs11975

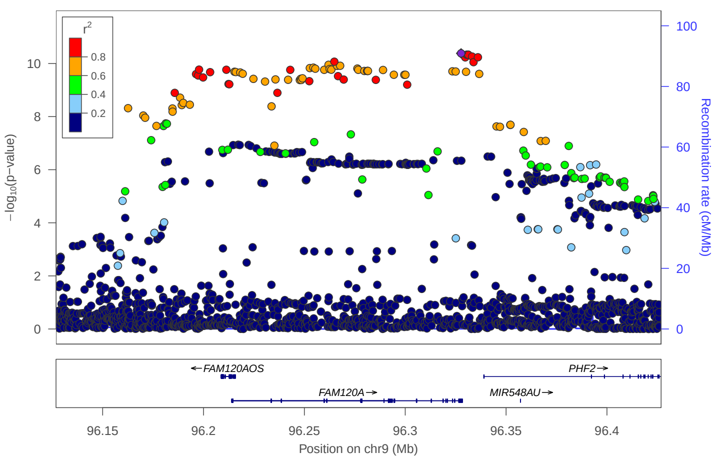

rs75614054

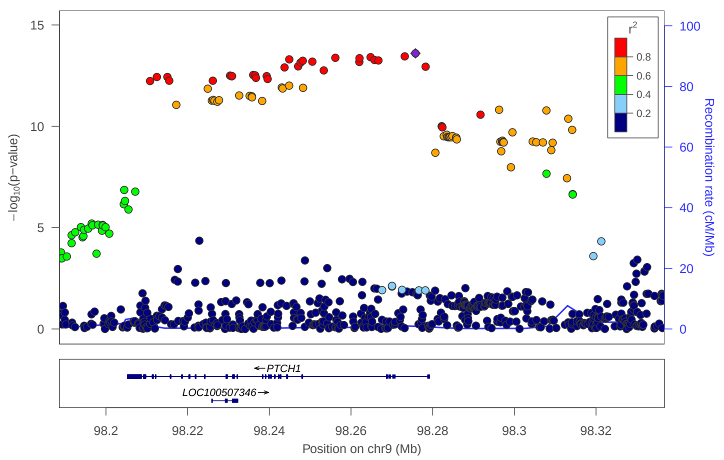

rs2149351

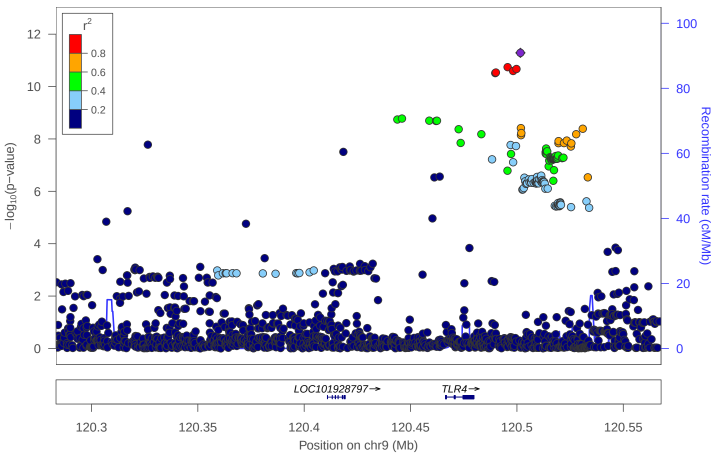

rs72759273

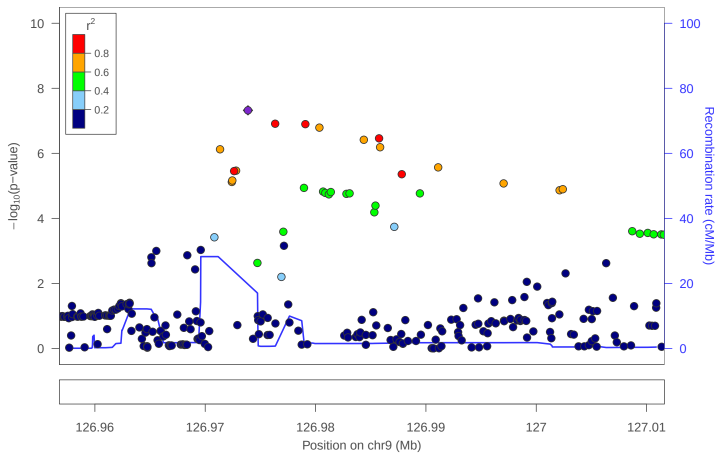

rs7895261

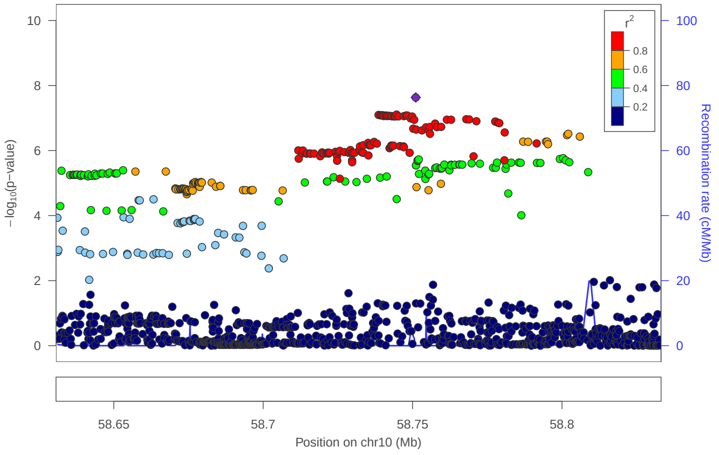

rs7912226

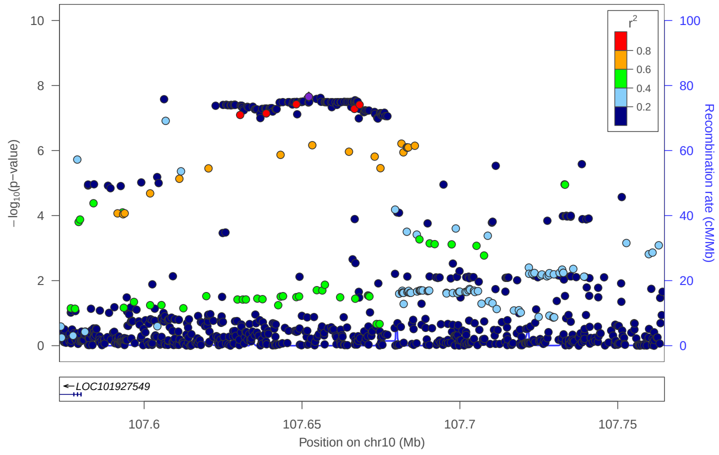

rs860626

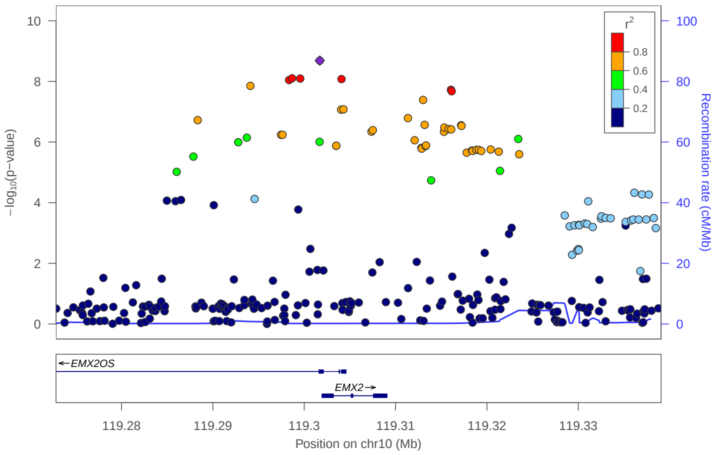

rs4757136

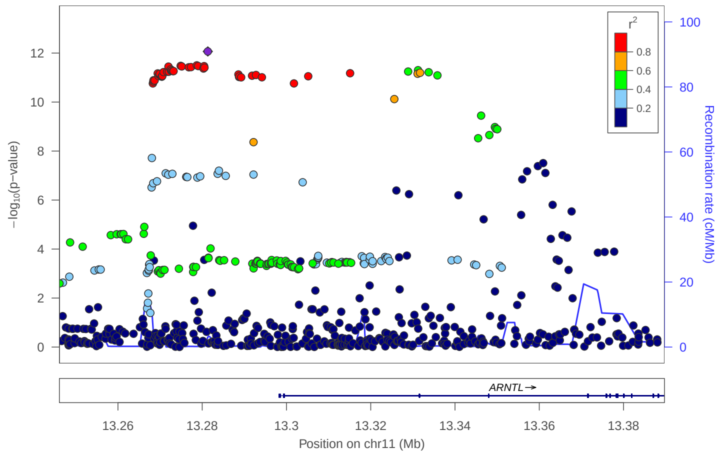

rs297346

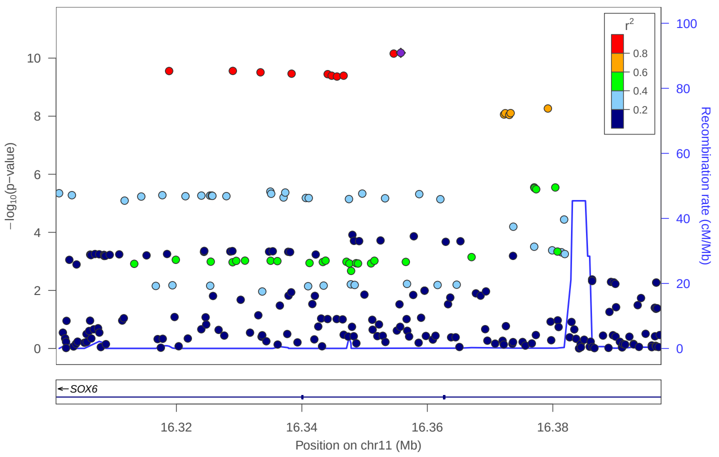

rs3026389

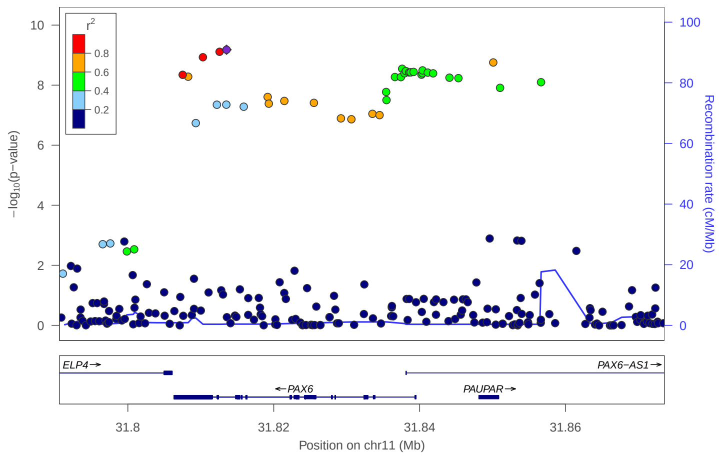

rs55702830

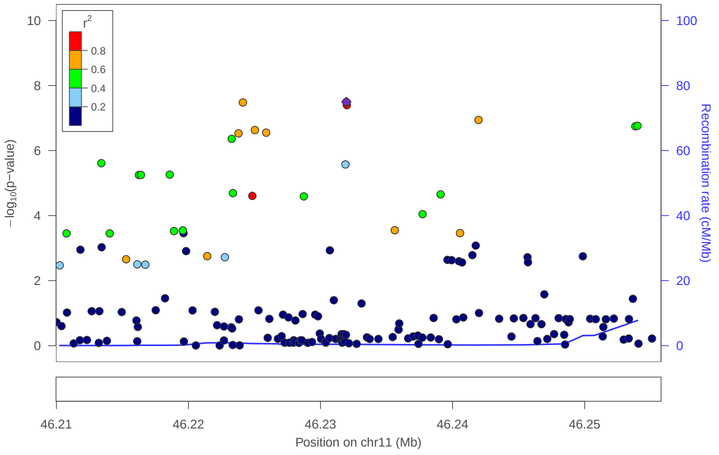

rs11039182

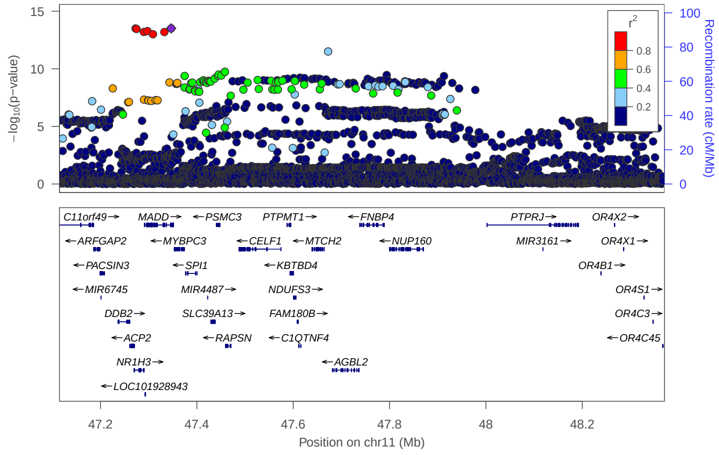

rs10896636

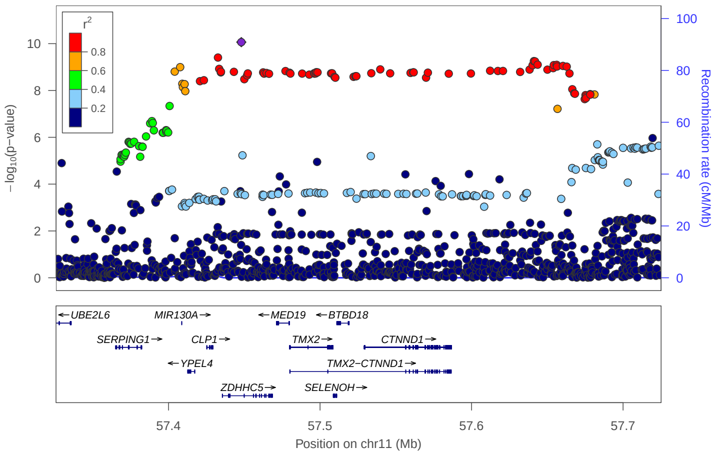

rs674437

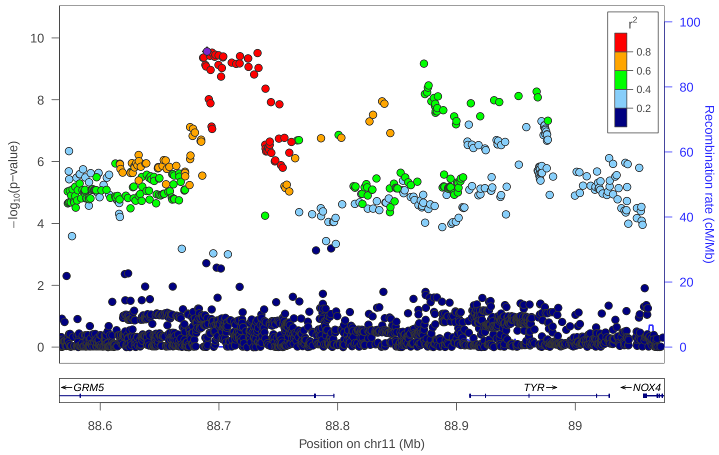

rs4245154

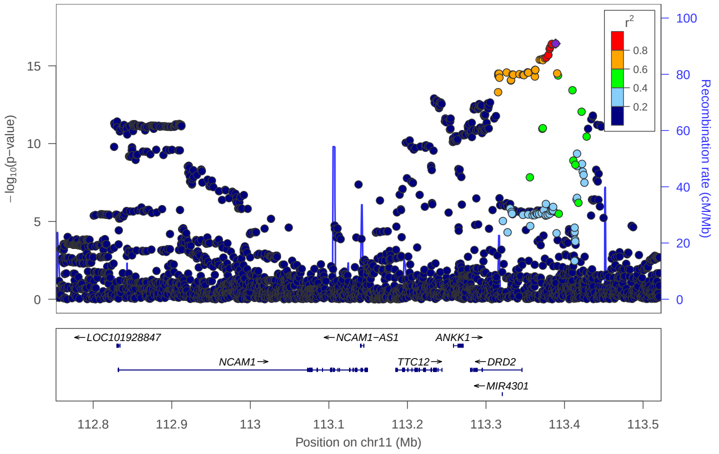

rs73034263

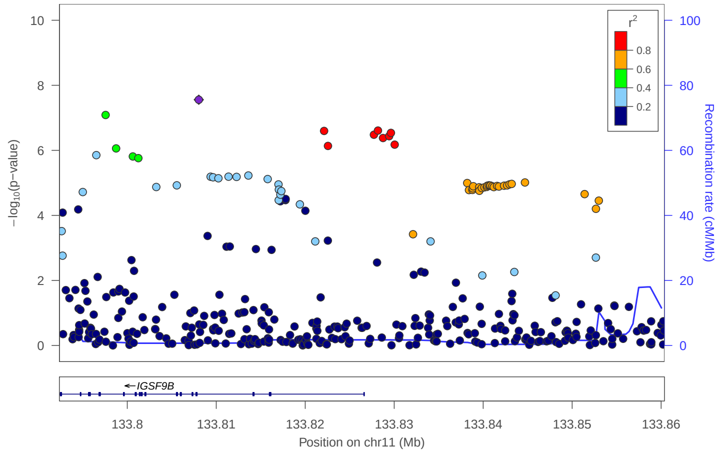

rs11608355

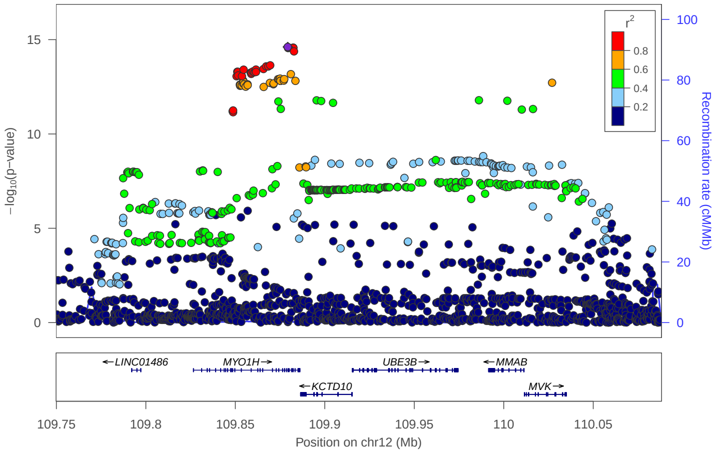

rs10507274

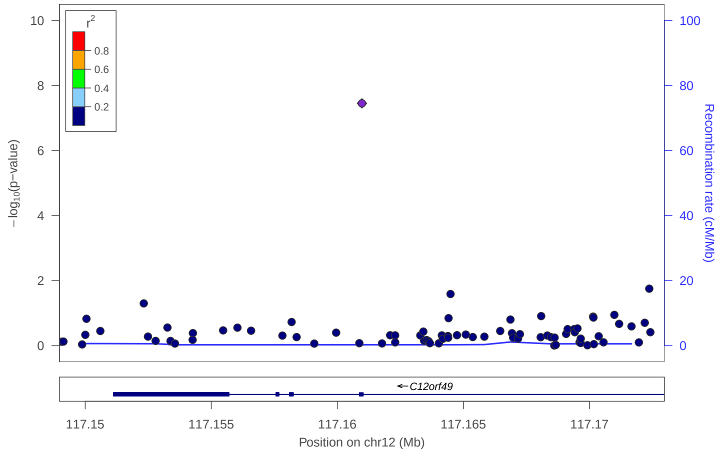

rs3741475

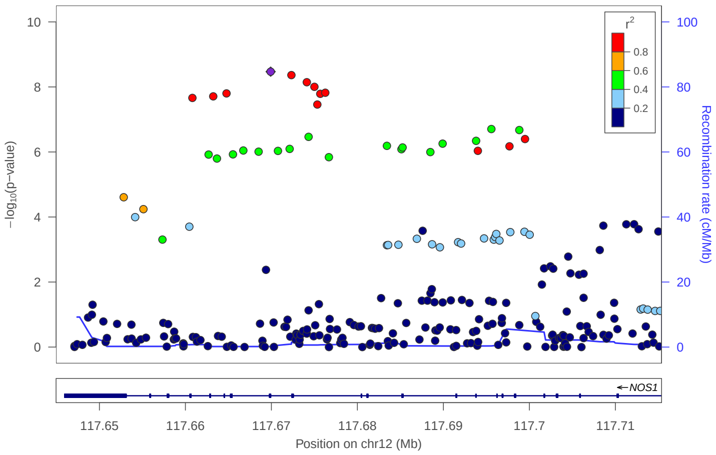

rs6490177

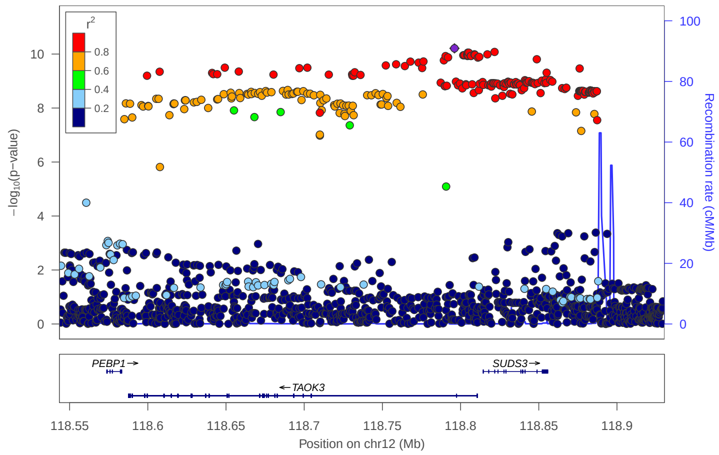

rs3124426

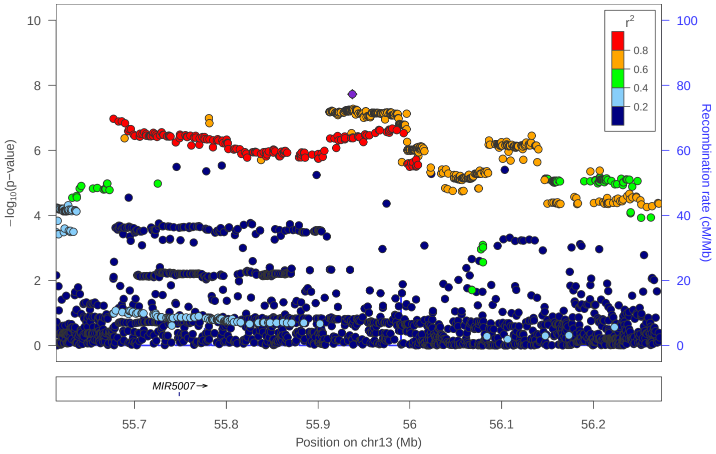

rs4444227

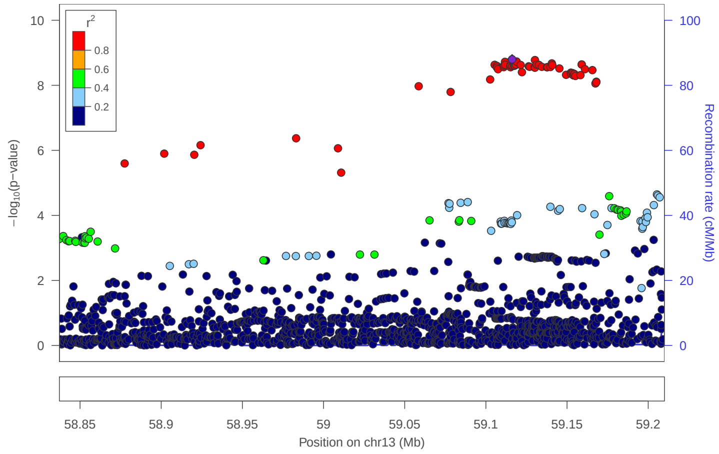

rs1892350

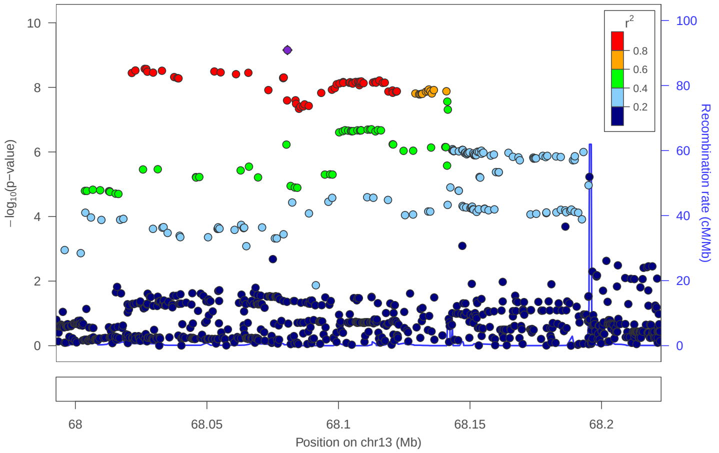

rs7338774

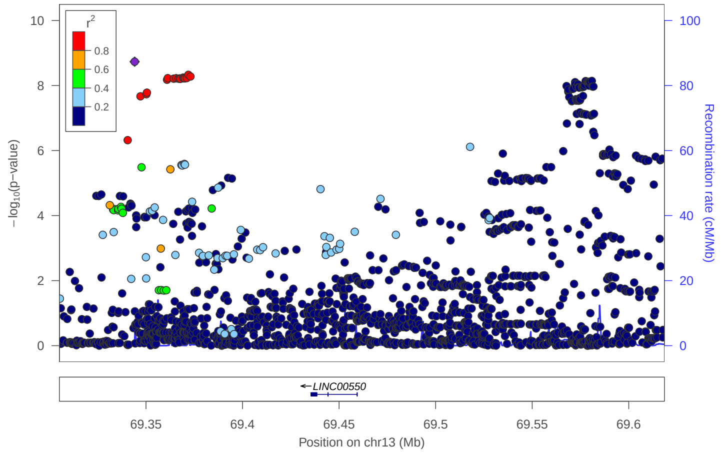

rs9516861

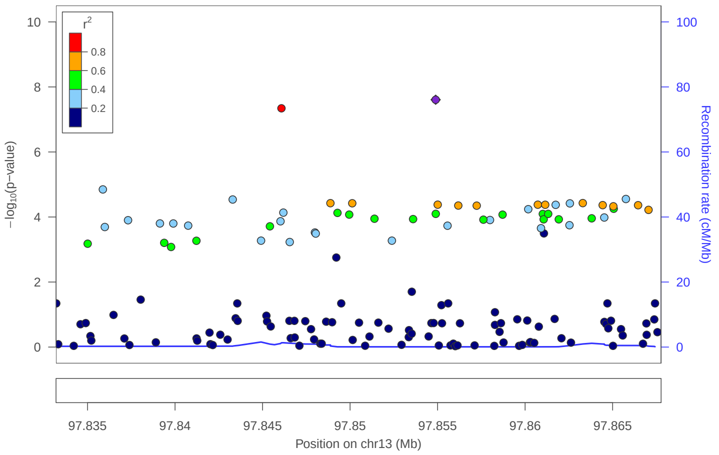

rs36006259

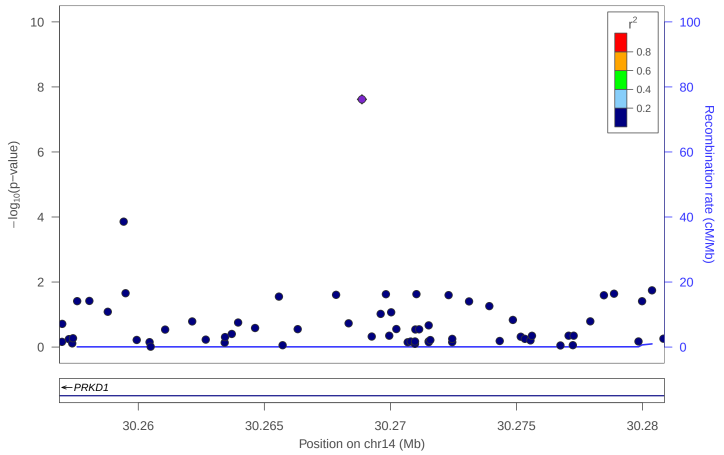

rs1778377

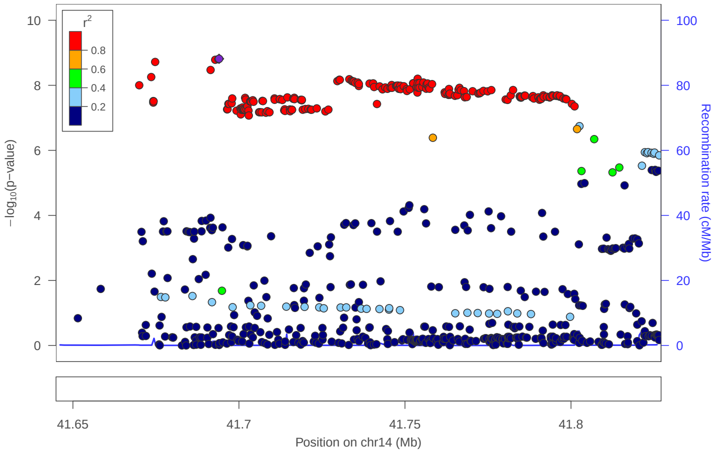

rs11627348

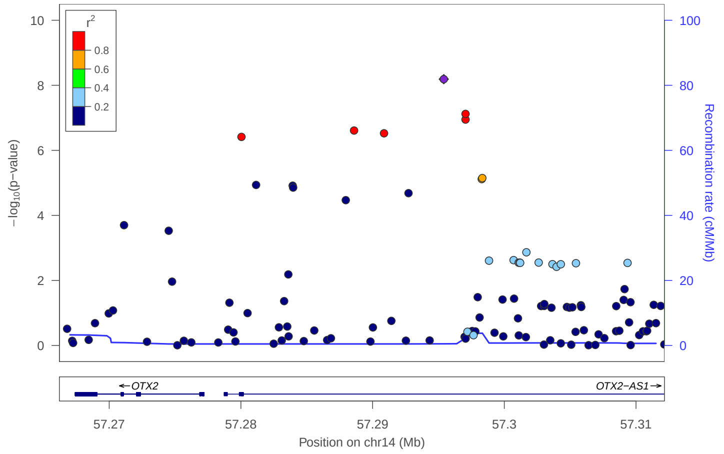

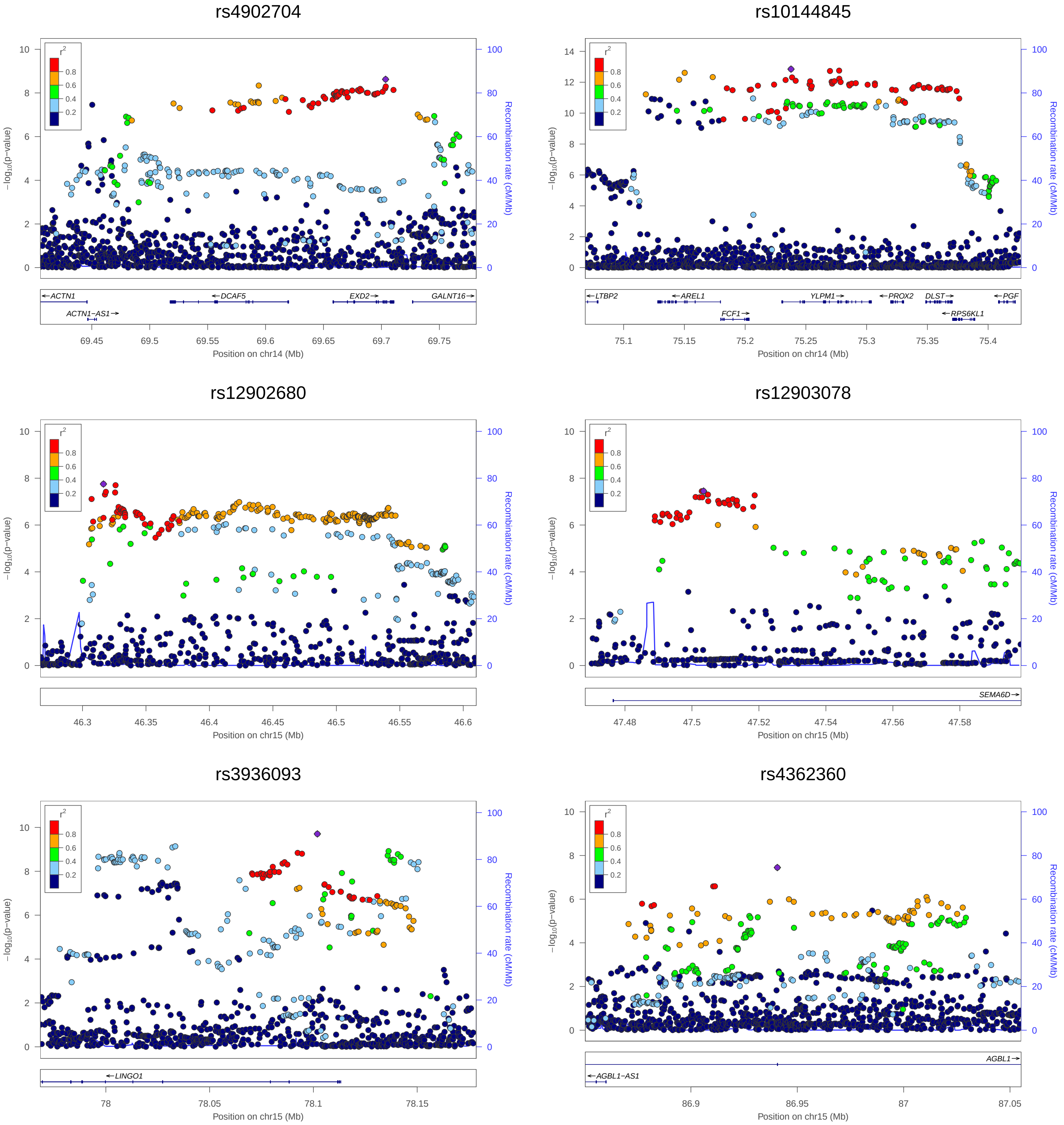
rs4902704
rs10144845
rs12902680
rs12903078
rs3936093
rs4362360
−log10(p−value)
Recombination rate (cM/Mb)
r2
0.8
0.6
0.4
0.2
ACTN1
ACTN1−AS1
DCAF5
EXD2
GALNT16
Position on chr14 (Mb)
LTBP2
AREL1
FCF1
YLPM1
PROX2
DLST
RPS6KL1
PGF
Position on chr15 (Mb)
SEMA6D
LINGO1
AGBL1
AGBL1−AS1

rs3785237

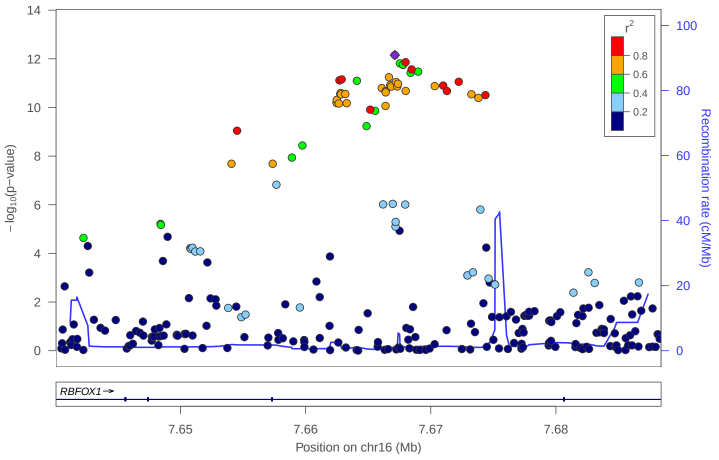

rs62055866

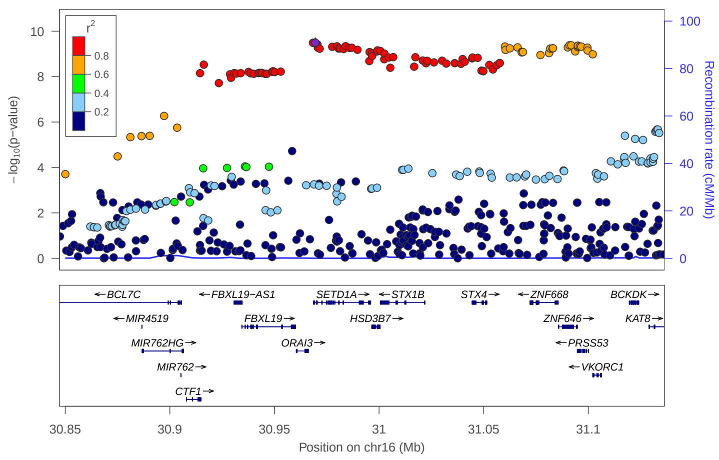

rs76082995

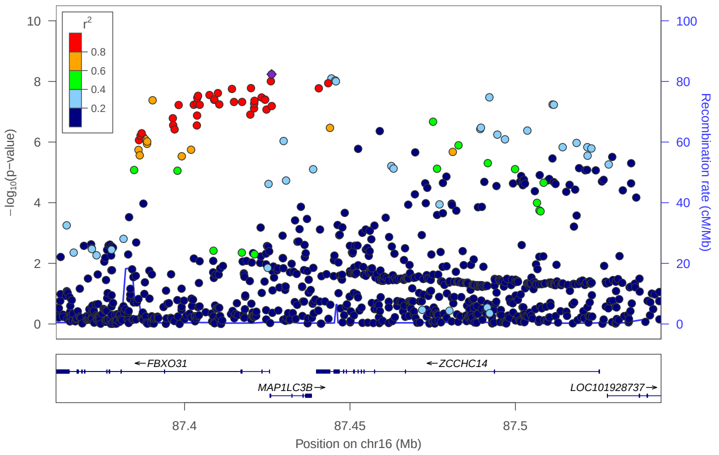

rs12938775

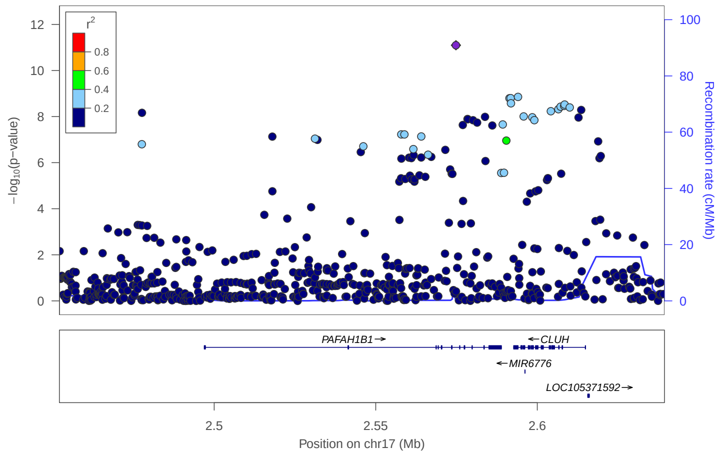

rs35982947

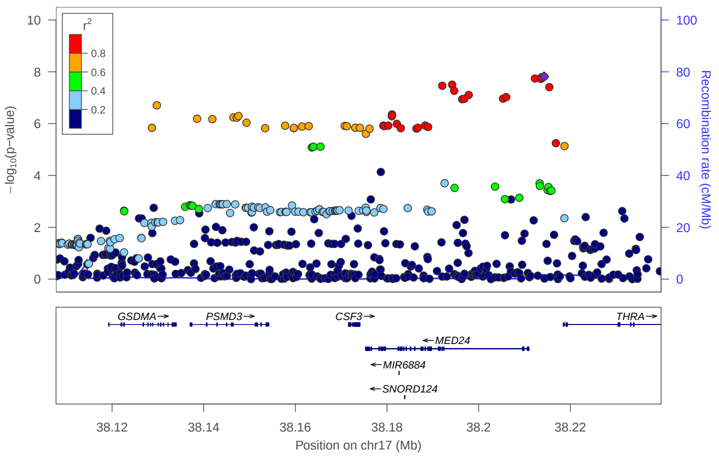

rs62062288

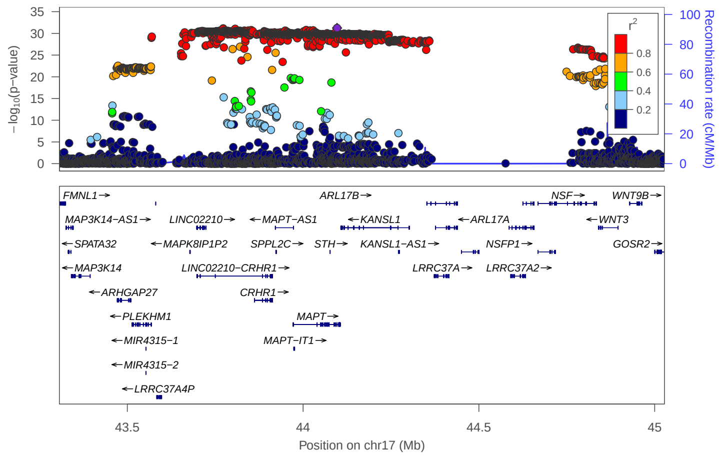

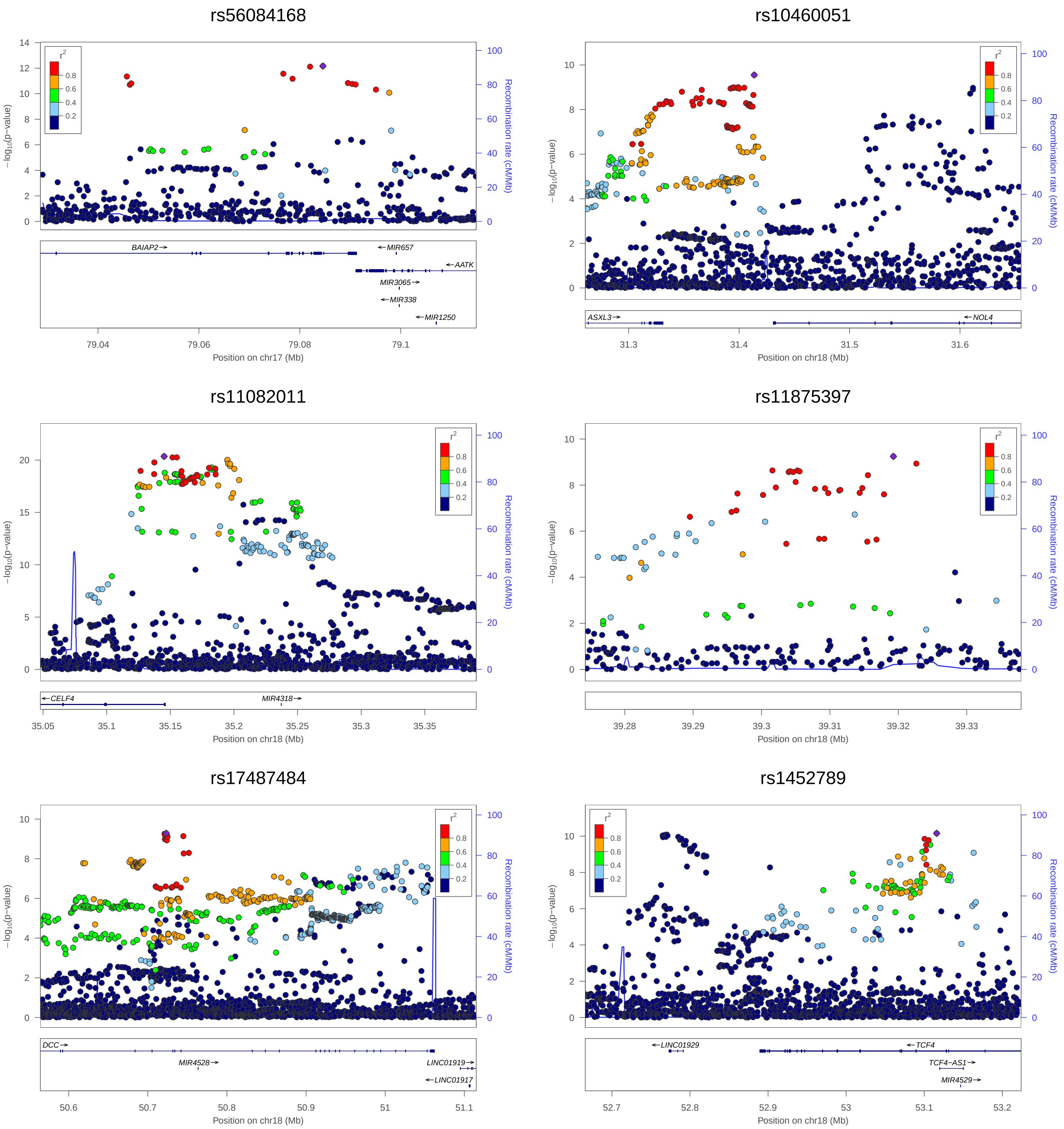
rs56084168
−log10(p−value)
Recombination rate (cM/Mb)
r²
BAIAP2→
←MIR657
←AATK
MIR3065→
←MIR338
←MIR1250
79.04
79.06
79.08
79.1
Position on chr17 (Mb)
rs10460051
ASXL3→
←NOL4
31.3
31.4
31.5
31.6
Position on chr18 (Mb)
rs11082011
←CELF4
MIR4318→
35.05
35.1
35.15
35.2
35.25
35.3
35.35
Position on chr18 (Mb)
rs11875397
39.28
39.29
39.3
39.31
39.32
39.33
Position on chr18 (Mb)
rs17487484
DCC→
MIR4528→
LINC01919→
←LINC01917
50.6
50.7
50.8
50.9
51
51.1
Position on chr18 (Mb)
rs1452789
←LINC01929
←TCF4
TCF4−AS1→
MIR4529→
52.7
52.8
52.9
53
53.1
53.2
Position on chr18 (Mb)

rs2295094

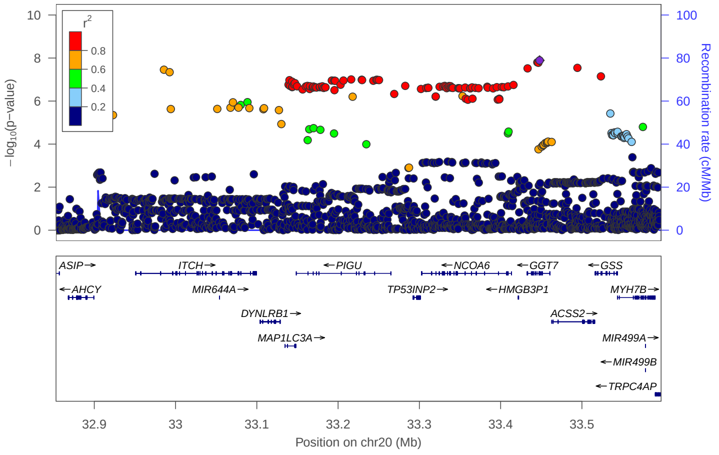

rs4578918

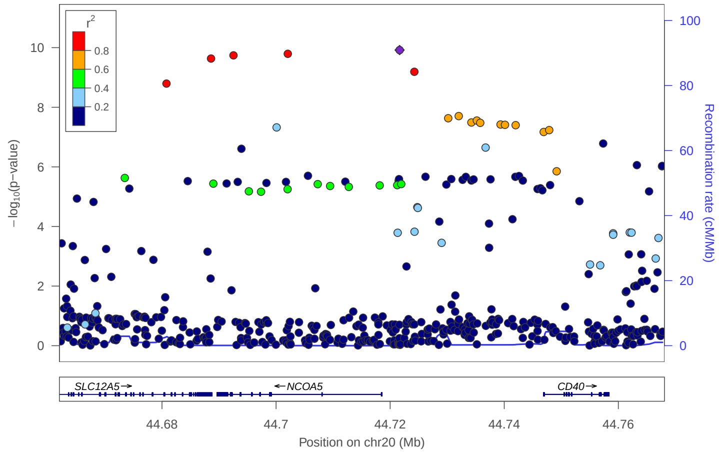

rs11090045

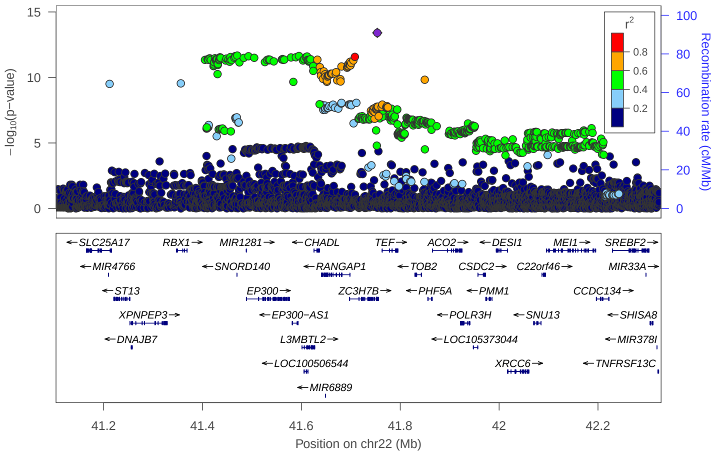

Supplementary Figure 17. Regional plots of GWA analyses of the neuroticism sum-score.

The *y*-axis represents the negative logarithm (base 10) of the SNP *P* value and the *x*-axis the position on the chromosome, with the name and location of genes in the UCSC Genome Browser in the bottom panel. The SNP with the lowest *P* value in the region is marked by a purple diamond. The colors of the other SNPs indicate the r^2 of these SNPs with the lead SNP, with grey color indicating SNPs below the user-defined r^2. Plots are generated with LocusZoom. See **Supplementary Data 2** for an overview of all lead SNPs for which a regional plot is presented above. For excluded loci (see **Methods**; **Supplementary Data 3-4**) the rsID's are marked yellow.

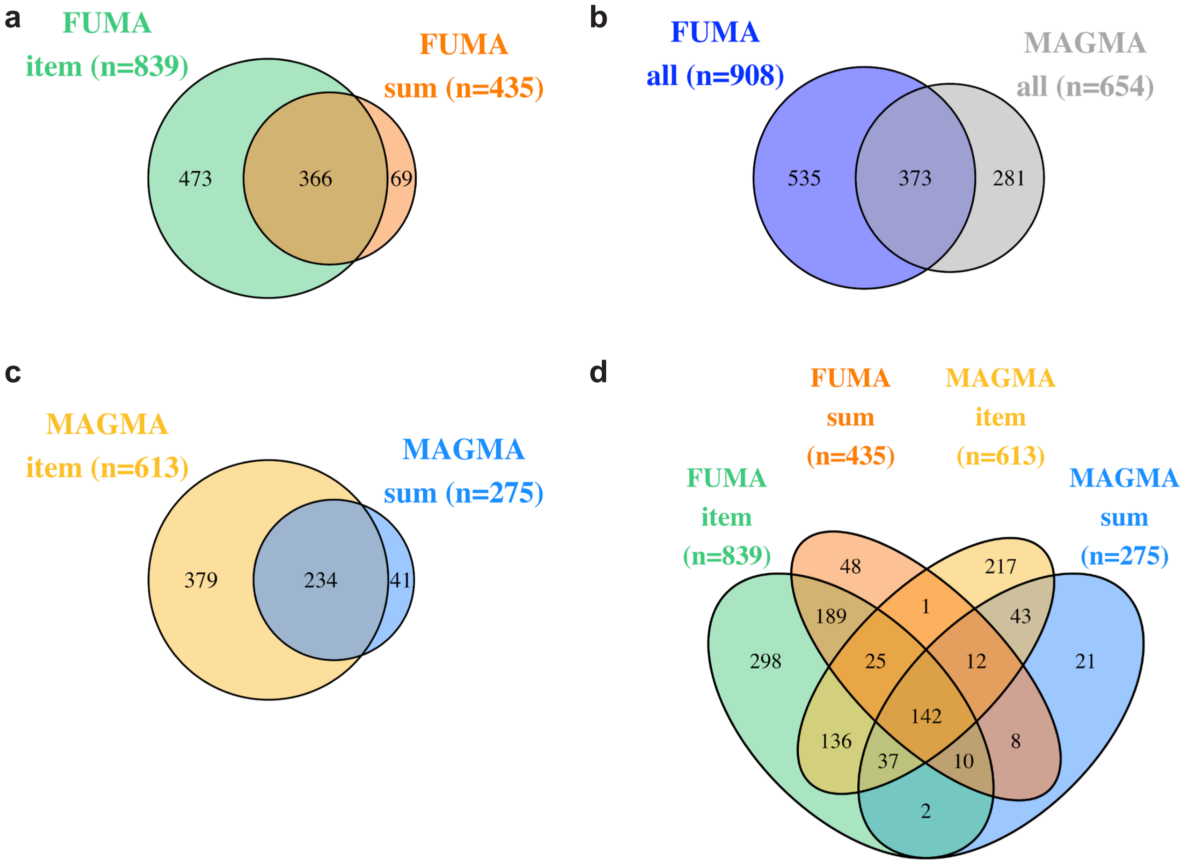

Supplementary Figure 18. Venn diagrams of overlap between genes implicated by SNP-based (FUMA) versus gene-based (MAGMA) analyses of the 12 neuroticism items and the sum-score.
(a) Overlap in number of genes implicated by FUMA in SNP-based analyses of the 12 neuroticism items and the sum-score. **(b)** Overlap in number of genes implicated by SNP-based analyses in FUMA versus gene-based analyses in MAGMA. **(c)** Overlap in number of genes identified in MAGMA's gene-based analyses of the 12 neuroticism items and the sum-score. **(d)** Overlap between genes implicated by FUMA and MAGMA for the 12 neuroticism items and the neuroticism sum-score.

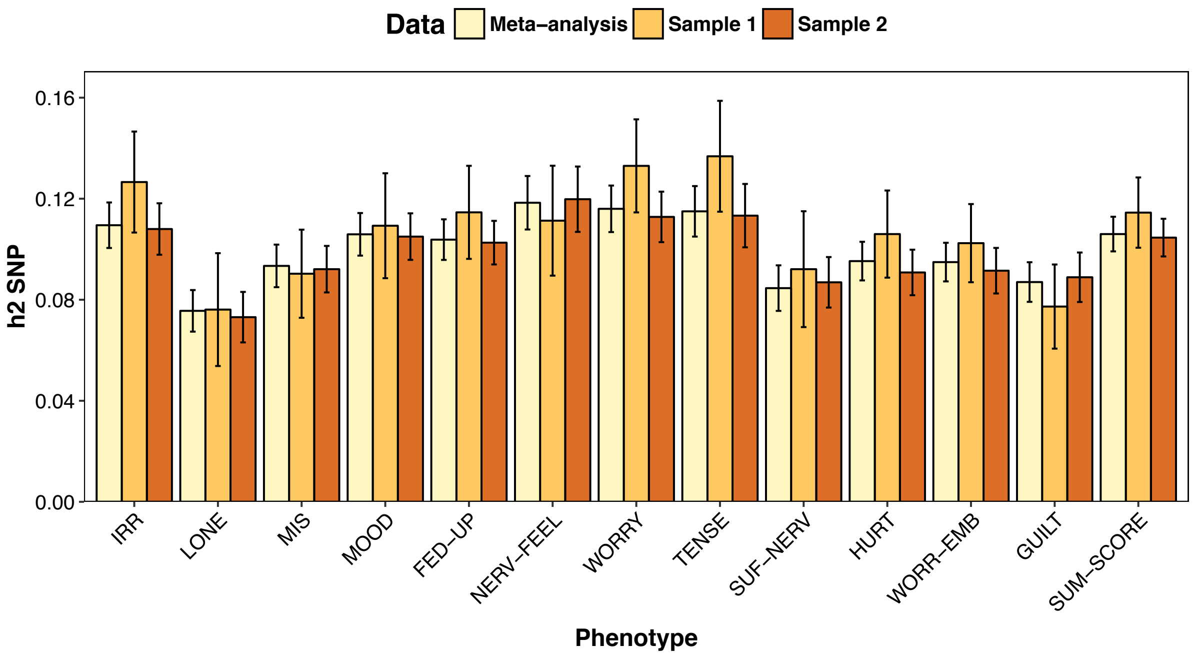

Supplementary Figure 19. SNP-based heritability for the 12 neuroticism items and the sum-score.

SNP-based heritability was computed on summary statistics of sample 1, sample 2, and the meta-analysis results of both samples, using LD score regression[6]. Error bars represent 95% confidence intervals.

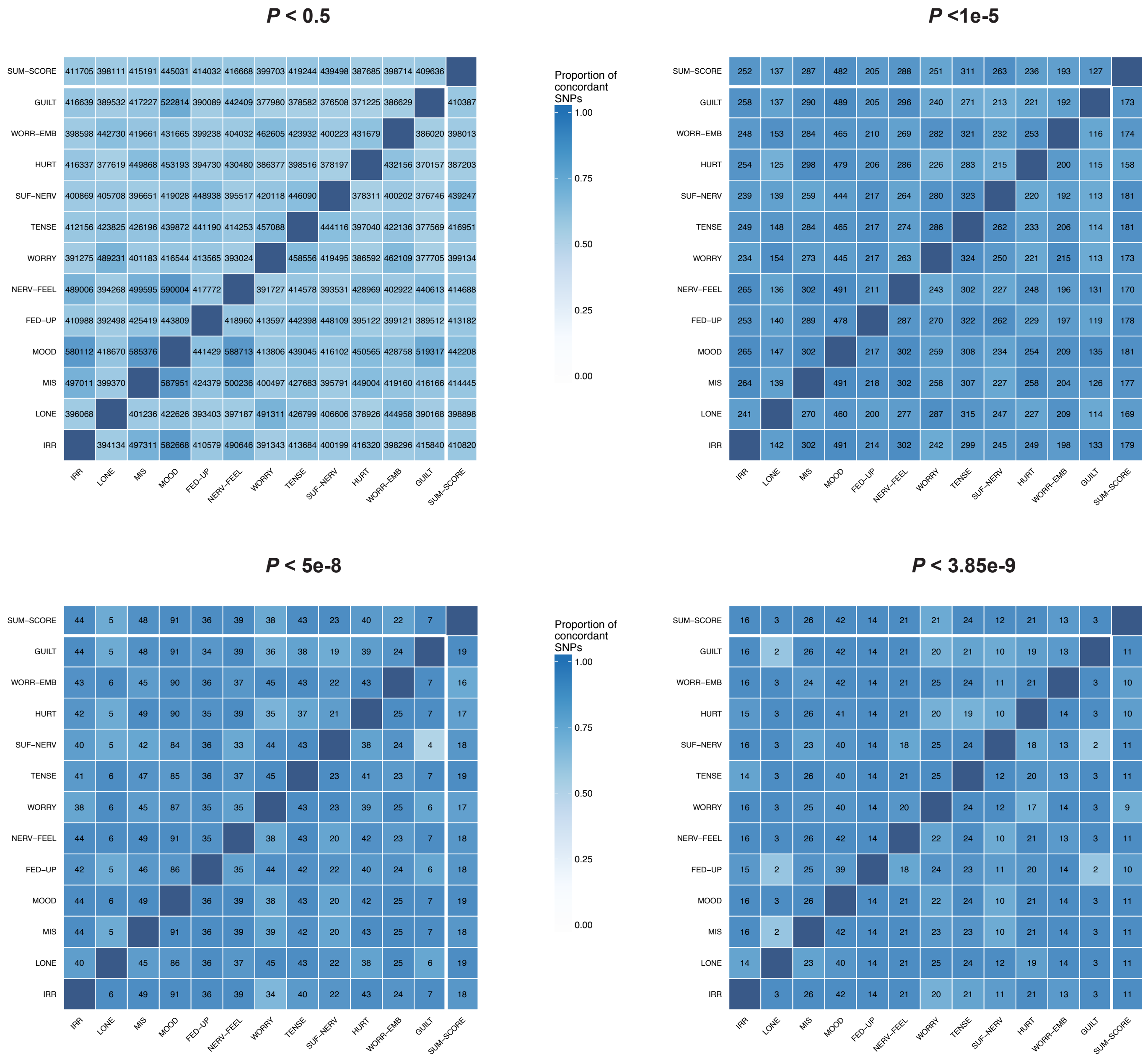

Digits in the cells indicate the number of SNPs for that specific combination of phenotypes that exceeded the given *P* value threshold. The depicted heatmaps are not symmetrical, as LD-based clumping was performed on both phenotypes (see **Methods** and **Supplementary Note 1**).

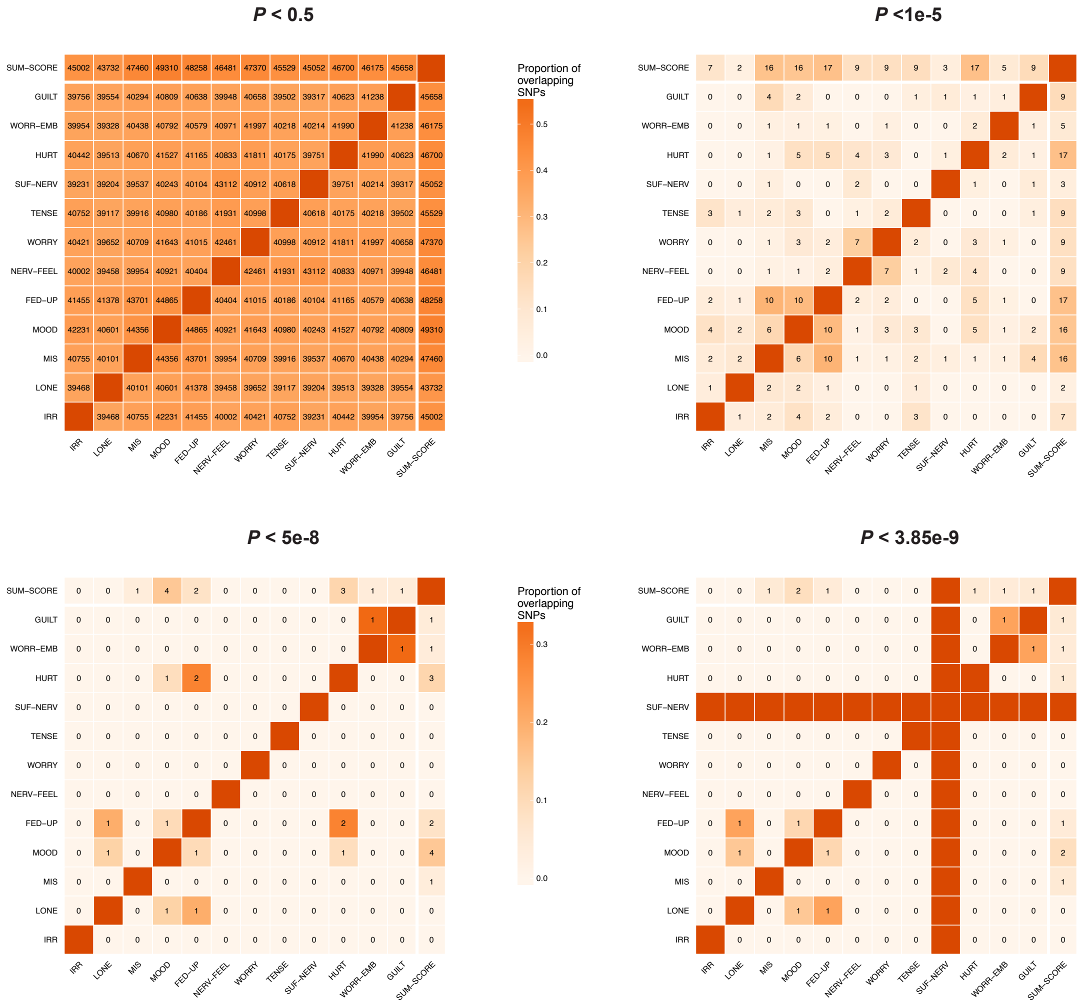

Supplementary Figure 21. Heatmaps showing the results from Fisher's exact test for overlap in the top SNPs (for different *P* value thresholds).

Digits in the cells indicate the number of SNPs for that specific combination of phenotypes that exceeded the given *P* value threshold (see **Methods** and **Supplementary Note 1**). If an off-diagonal cell contains no value this indicates that there were no SNPs with a *P* value lower than the given threshold for that phenotype. A value '0' indicates that although there were SNPs with *P* values lower than the threshold for both phenotypes, these were not the same SNPs. All heatmaps in this figure are symmetric.

IRR - Are you an irritable person?

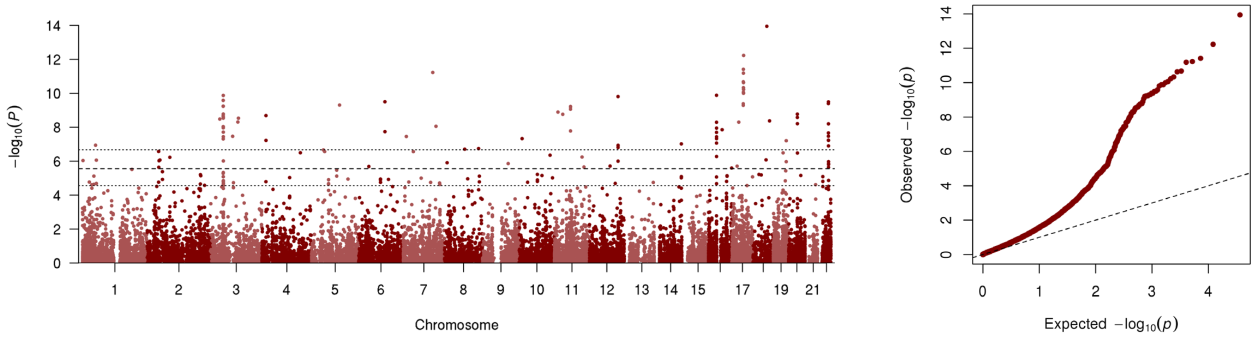

LONE - Do you often feel lonely?

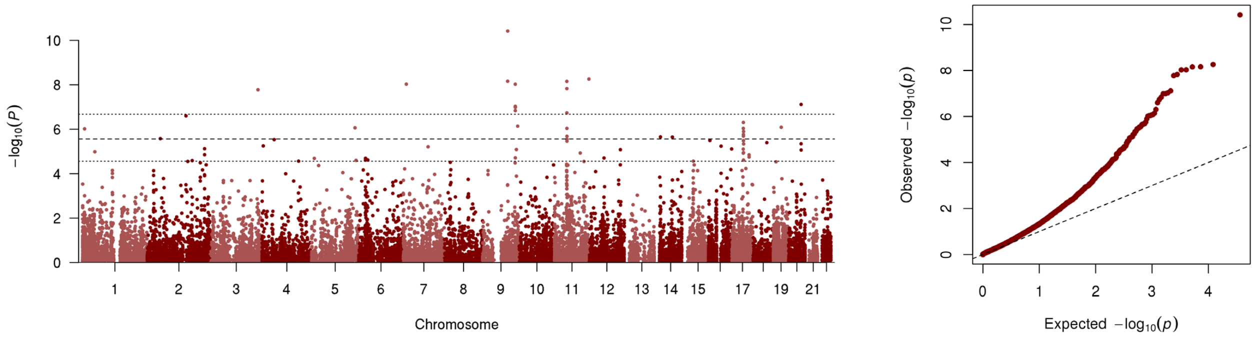

MIS - Do you ever feel ‘just miserable’ for no reason?

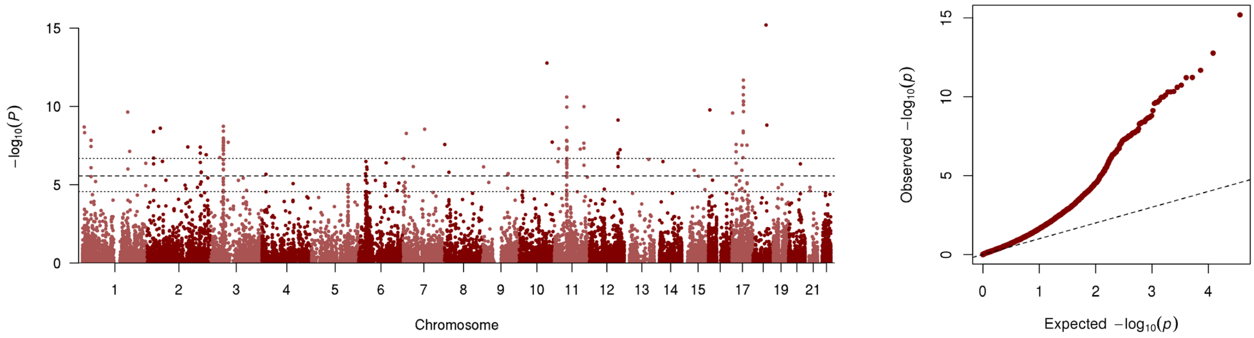

MOOD - Does your mood often go up and down?

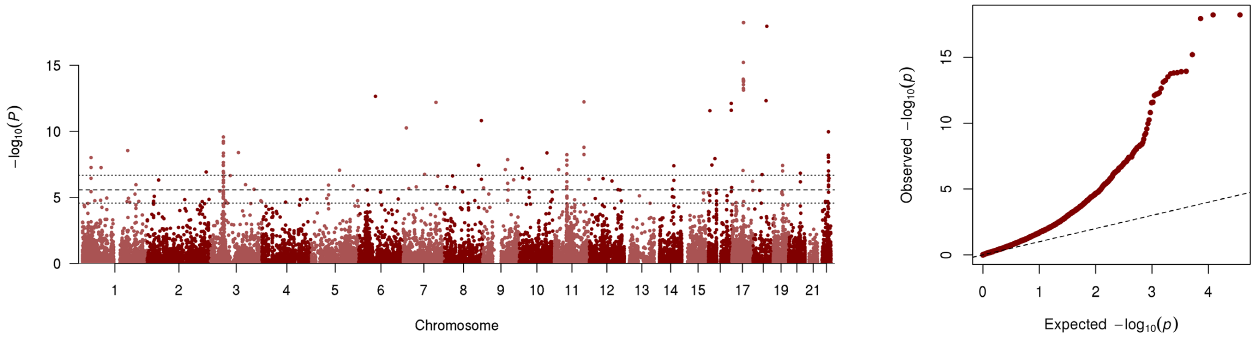

FED-UP - Do you often feel ‘fed-up’?

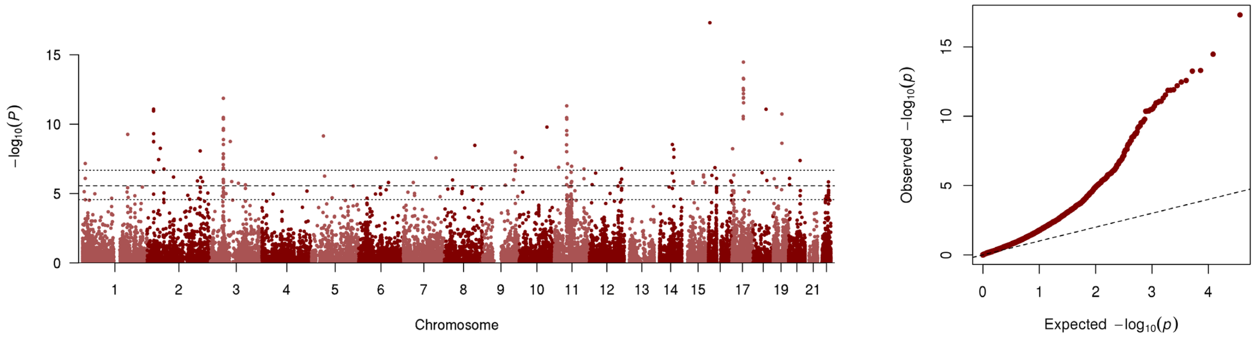

NERV-FEEL - Would you call yourself a nervous person?

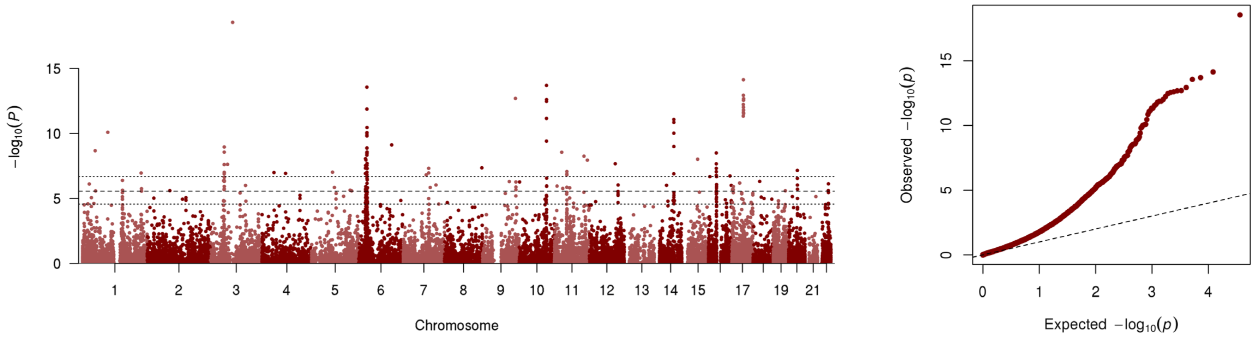

WORRY - Are you a worrier?

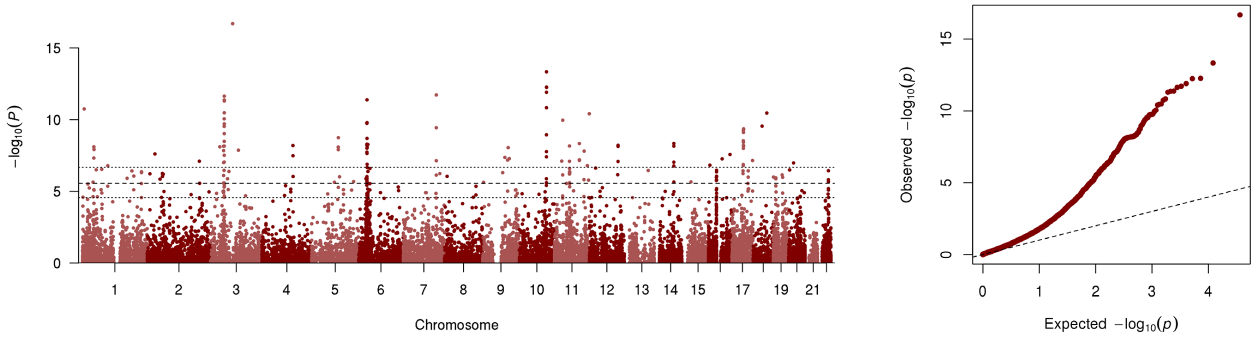

TENSE - Would you call yourself tense or ‘highly strung’?

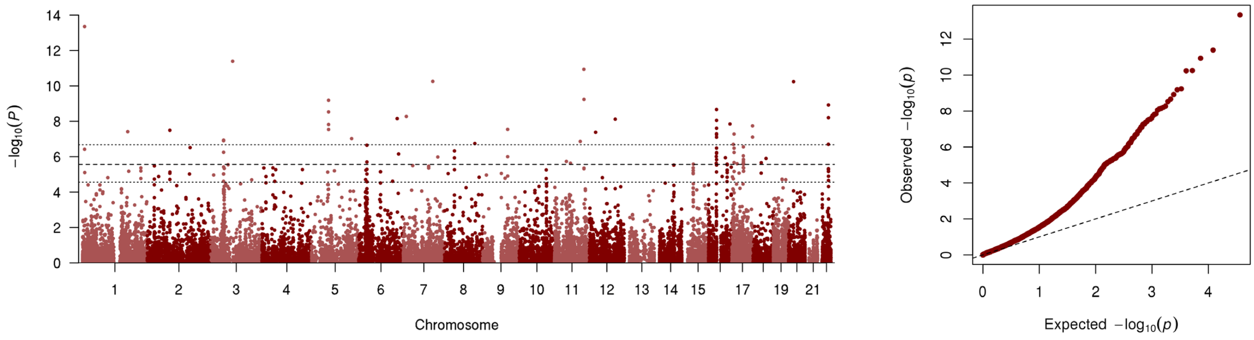

SUF-NERV - Do you suffer from ‘nerves’?

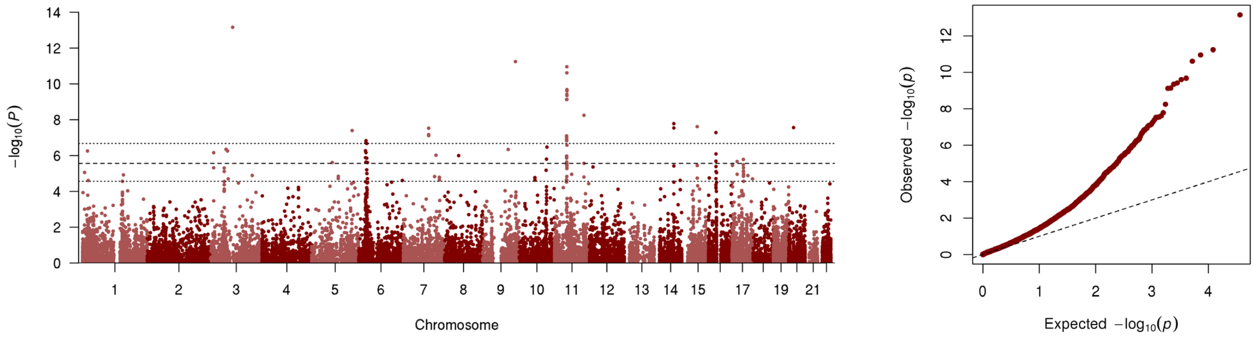

HURT - Are your feelings easily hurt?

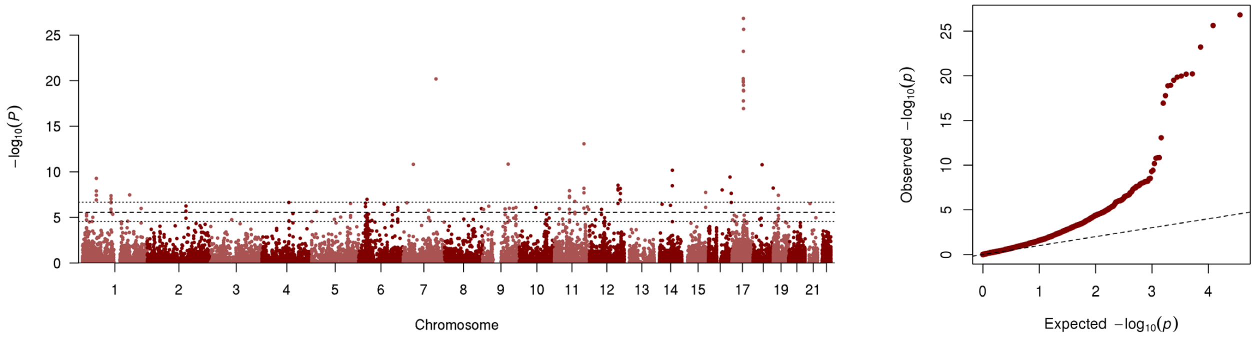

WORR-EMB - Do you worry too long after an embarrassing experience?

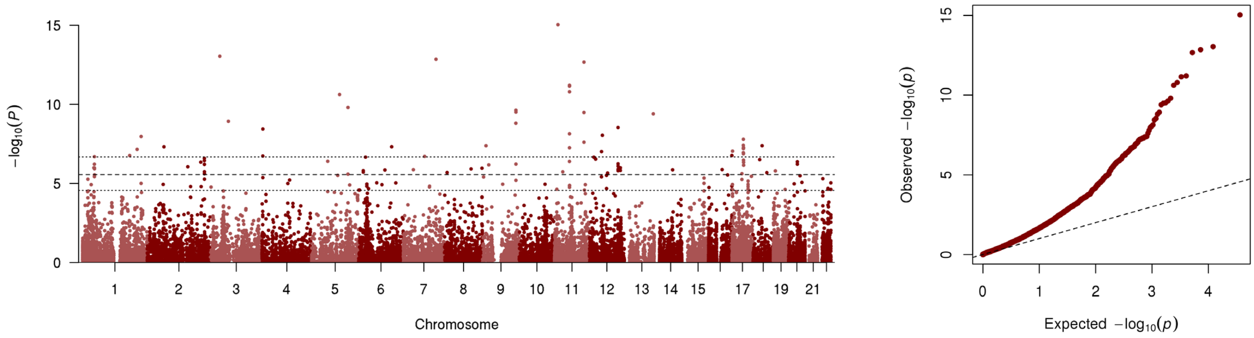

GUILT - Are you often troubled by feelings of guilt?

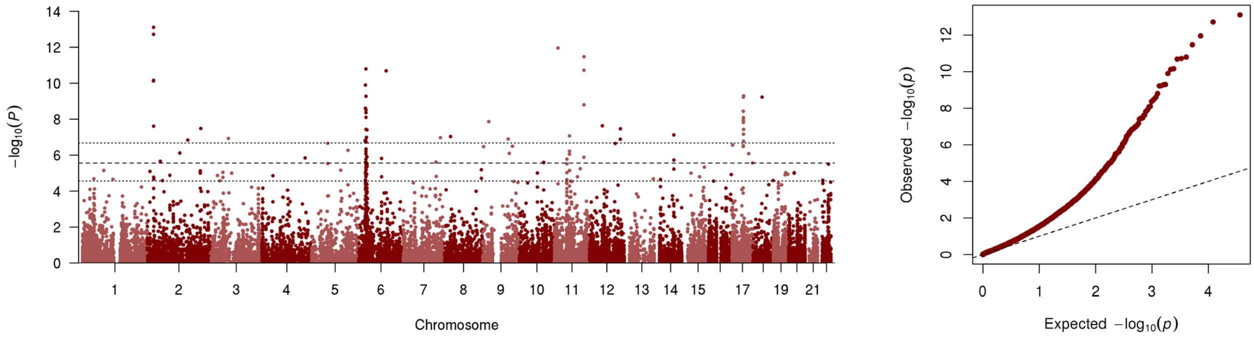

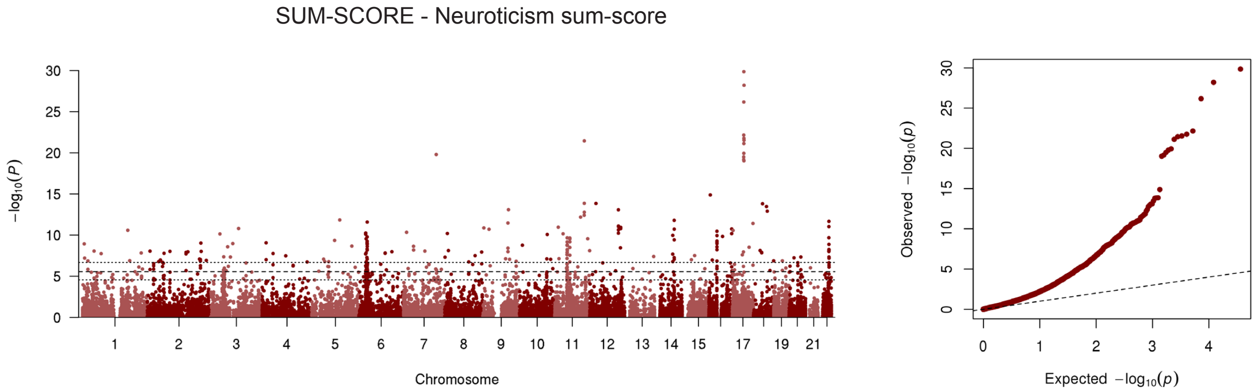

Supplementary Figure 22. Manhattan and quantile-quantile (Q-Q) plots of the gene-based association results for the individual items and the neuroticism sum-score.

Gene association results from the MAGMA gene-based tests, which are based on the summary statistics of the meta-analyses of the 12 neuroticism items and the sum-score. Each dot represents a gene. The *y*-axis shows the -log10 *P* values and the *x*-axis the chromosomal position of a given gene. Dashed lines indicate genome-wide significance ($P < 2.75\times10^{-6}$). The upper dotted line indicates a more stringent Bonferroni-corrected significance threshold, where we corrected for testing 13 phenotypes; $P < 2.12\times10^{-7}$ ($2.75\times10^{-6}/13$) whereas the lower dotted line indicates the 'suggestive' significance threshold ($P < 2.75\times10^{-5}$). See **Supplementary Table 1** for an explanation of item labels.

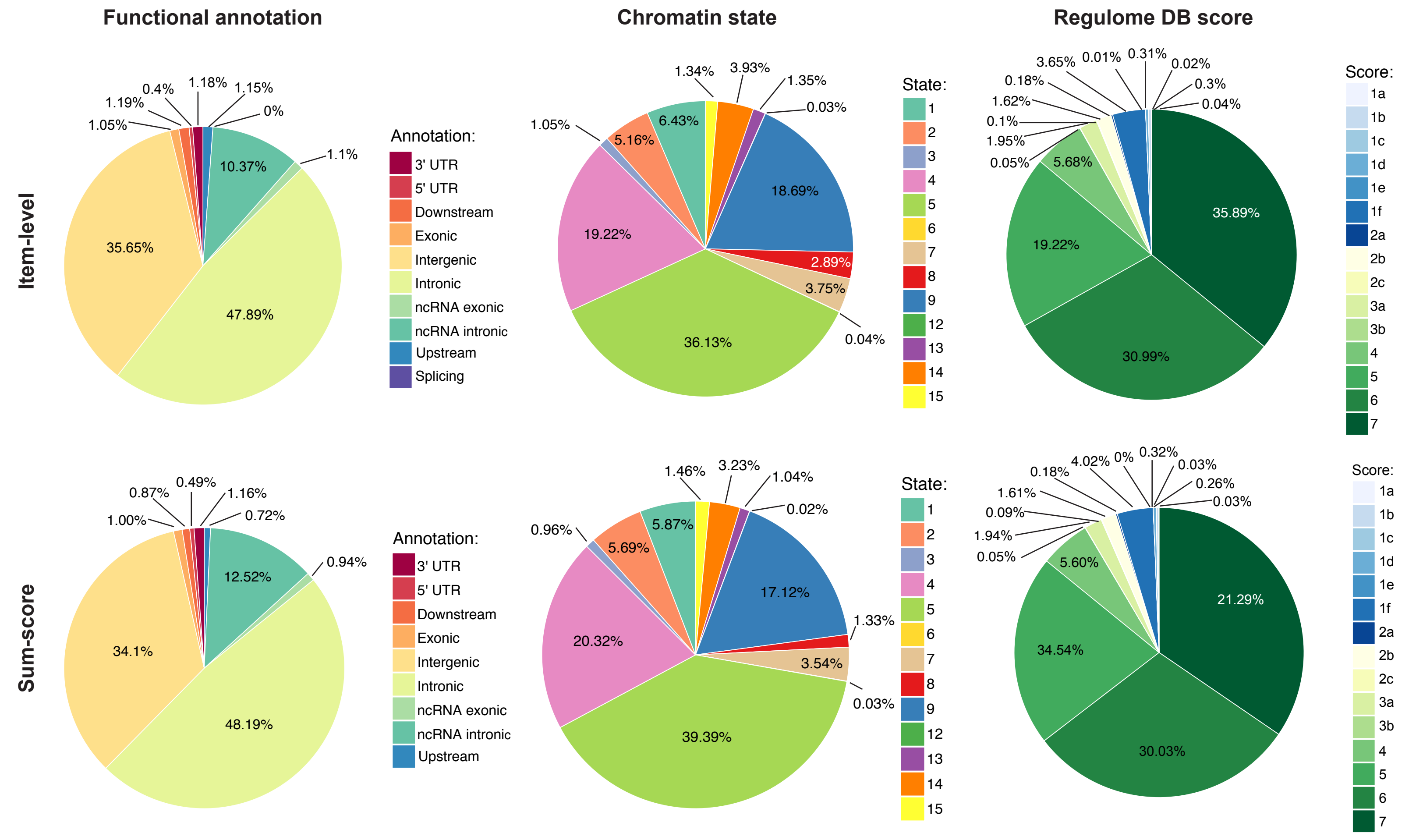
Functional annotation
Chromatin state
Regulome DB score
Item-level
Sum-score
Annotation:
3' UTR
5' UTR
Downstream
Exonic
Intergenic
Intronic
ncRNA exonic
ncRNA intronic
Upstream
Splicing
1.05%
1.19%
0.4%
1.18%
1.15%
0%
1.1%
10.37%
35.65%
47.89%
State:
1
2
3
4
5
6
7
8
9
12
13
14
15
1.34%
3.93%
1.35%
0.03%
1.05%
5.16%
6.43%
18.69%
19.22%
2.89%
3.75%
0.04%
36.13%
Score:
1a
1b
1c
1d
1e
1f
2a
2b
2c
3a
3b
4
5
6
7
3.65%
0.01%
0.31%
0.02%
0.18%
0.3%
0.04%
1.62%
0.1%
1.95%
0.05%
5.68%
35.89%
19.22%
30.99%
0.87%
0.49%
1.16%
1.00%
0.72%
0.94%
12.52%
34.1%
48.19%
1.46%
3.23%
1.04%
0.02%
0.96%
5.69%
5.87%
17.12%
20.32%
1.33%
3.54%
0.03%
39.39%
0.18%
4.02%
0%
0.32%
0.03%
1.61%
0.26%
0.03%
0.09%
1.94%
0.05%
5.60%
21.29%
34.54%
30.03%

Supplementary Figure 23. Functional categories, chromatin state and regulome DB score for SNPs that were GWS in item-level analyses versus analysis of the neuroticism sum-score.

The functional annotation indicates the functional consequences on the gene to which a given SNP was annotated (**Supplementary Table 6**). The chromatin state refers to the minimum (i.e., most active) chromatin state across 127 tissues for all genome-wide significant SNPs**.** The lower the regulome DB (database) score, the more likely it is that the SNP has a regulatory function. Coding of the chromatin states and regulome DB scores is presented in **Supplementary Tables 7-8**. Annotation was performed in FUMA[7].

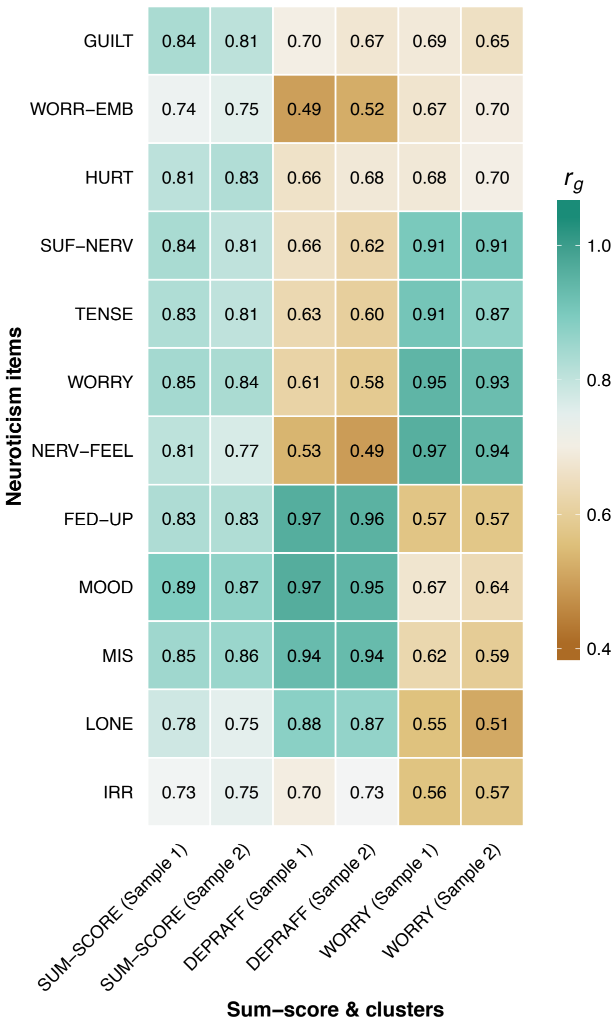

Supplementary Figure 24. Genetic correlations between the 12 neuroticism items and the sum- and cluster-scores in samples 1 and 2.

Genetic correlations between the 12 neuroticism items and the sum- and cluster-scores in both samples. The genetic correlations of the same items measured in both samples are presented on the diagonal. All reported genetic correlations, computed with bivariate LD score regression, are significantly different from zero after Bonferroni correction ($P < 2.22\times10^{-4}$).

See **Supplementary Table 1** for a description of the item labels.

Supplementary References

1. Eysenck, B. G., Eysenck, H. J. & Barrett, P. A revised version of the psychoticism scale. *Pers. Individ. Dif.* **6,** 21–29 (1985).
2. Beck, A. T., Ward, C. H., Mendelson, M., Mock, J. & Erbaugh, J. An Inventory for Measuring Depression. *Arch. Gen. Psychiatry* **4,** 561–571 (1961).
3. Rush, A. J., Gullion, C. M., Basco, M. R., Jarrett, R. B. & Trivedi, M. H. The Inventory of Depressive Symptomatology (IDS): psychometric properties. *Psychol. Med.* **26,** 477–486 (1996).
4. Hamilton, M. . Hamilton Depression Rating Scale (HDRS). *J Neurol Neurosurg Psychiatry* **23,** 56–62 (1960).
5. Achenbach, T. *Manual for the Child Behavior Checklist/4-18 and 1991 Profile.* (Burlington, VT: University of Vermont, Department of Psychiatry, 1991).
6. Bulik-Sullivan, B. K. *et al.* LD Score regression distinguishes confounding from polygenicity in genome-wide association studies. *Nat. Genet.* **47,** 291–295 (2015).
7. Watanabe, K., Taskesen, E., Bochoven, A. van & Posthuma, D. FUMA: Functional mapping and annotation of genetic associations. *Nat. Commun.* **8,** (2017).
8. American Psychiatric Association. *Diagnostic and statistical manual of mental disorders (5th ed.)*. (Author, 2013). doi:http://dx.doi.org/10.1176/appi.books.9780890425596
